# Comparing organization-focused and state-focused financing strategies on provider-level reach of a youth substance use treatment model: a mixed-method study

**DOI:** 10.1186/s13012-023-01305-z

**Published:** 2023-10-12

**Authors:** Alex R. Dopp, Sarah B. Hunter, Mark D. Godley, Isabelle González, Michelle Bongard, Bing Han, Jonathan Cantor, Grace Hindmarch, Kerry Lindquist, Blanche Wright, Danielle Schlang, Lora L. Passetti, Kelli L. Wright, Beau Kilmer, Gregory A. Aarons, Jonathan Purtle

**Affiliations:** 1 RAND Corporation, 1776 Main Street, Santa Monica, CA 90401 USA; 2 Chestnut Health Systems, 448 Wylie Drive, Normal, IL 61761 USA; 3 Department of Research and Evaluation, Division of Biostatistics Research, Kaiser Permanente Southern California, 100 South Los Robles Avenue 2nd Floor, Pasadena, CA 91101 USA; 4 Department of Health Policy and Management, University of California Los Angeles, 650 Charles Young Dr. S., 31-269 CHS Box 951772, Los Angeles, CA 90095 USA; 5 Department of Psychiatry and Altman Clinical and Translational Research Institute Dissemination and Implementation Science Center, University of California San Diego, 9500 Gilman Dr. (0812), La Jolla, San Diego, CA 92093 USA; 6 Department of Public Health Policy & Management and Global Center for Implementation Science, New York University School of Global Public Health, 708 Broadway, New York, NY 10003 USA

**Keywords:** Youth substance use, Substance use disorder treatment, Evidence-based practices, A-CRA, Behavioral health service systems, Financing strategies, Implementation, Reach, Public finance, Policy

## Abstract

**Background:**

Financial barriers in substance use disorder service systems have limited the widespread adoption—i.e., provider-level reach—of evidence-based practices (EBPs) for youth substance use disorders. Reach is essential to maximizing the population-level impact of EBPs. One promising, but rarely studied, type of implementation strategy for overcoming barriers to EBP reach is financing strategies, which direct financial resources in various ways to support implementation. We evaluated financing strategies for the Adolescent Community Reinforcement Approach (A-CRA) EBP by comparing two US federal grant mechanisms, organization-focused and state-focused grants, on organization-level A-CRA reach outcomes.

**Method:**

A-CRA implementation took place through organization-focused and state-focused grantee cohorts from 2006 to 2021. We used a quasi-experimental, mixed-method design to compare reach between treatment organizations funded by organization-focused versus state-focused grants (164 organizations, 35 states). Using administrative training records, we calculated reach as the per-organization proportion of trained individuals who received certification in A-CRA clinical delivery and/or supervision by the end of grant funding. We tested differences in certification rate by grant type using multivariable linear regression models that controlled for key covariates (e.g., time), and tested threats to internal validity from our quasi-experimental design through a series of sensitivity analyses. We also drew on interviews and surveys collected from the treatment organizations and (when relevant) interviews with state administrators to identify factors that influenced reach.

**Results:**

The overall certification rates were 27 percentage points lower in state-focused versus organization-focused grants (*p* = .01). Sensitivity analyses suggested these findings were not explained by confounding temporal trends nor by organizational or state characteristics. We did not identify significant quantitative moderators of reach outcomes, but qualitative findings suggested certain facilitating factors were more influential for organization-focused grants (e.g., strategic planning) and certain barrier factors were more impactful for state-focused grants (e.g., states finding it difficult to execute grant activities).

**Discussion:**

As the first published comparison of EBP reach outcomes between financing strategies, our findings can help guide state and federal policy related to financing strategies for implementing EBPs that reduce youth substance use. Future work should explore contextual conditions under which different financing strategies can support the widespread implementation of EBPs for substance use disorder treatment.

**Supplementary Information:**

The online version contains supplementary material available at 10.1186/s13012-023-01305-z.

Contributions to the literature

- Organization-focused grants were robustly associated with higher average organization-level certification rates in the Adolescent Community Reinforcement Approach, revealing a notable strength of that financing strategy relative to state-focused grants.
- Qualitative findings revealed key differences in facilitators and barriers by grant type, which help identify conditions under which each grant type could be most useful for maximizing provider adoption of evidence-based practices.
- These findings offer evidence to inform state and federal investments into policy efforts to implement evidence-based practices.

## Background

Research has identified several evidence-based practices (EBPs) for the treatment of adolescent and young adult substance use disorders (i.e., youth SUD) [1]. One exemplar is the Adolescent Community Reinforcement Approach (A-CRA [2]), a 12–14-week behavioral treatment for youth ages 12–25 that improved substance use, mental health, and social outcomes in randomized trials (as summarized in a recent systematic review [1]). A-CRA has been widely implemented in the USA, where it was developed, and several other countries (Canada, Brazil, Spain, and the Netherlands). It embodies many common EBP features for youth SUD, such as the use of strategies to modify factors related to substance use, procedures to address family and community influences, and community-based delivery options [1].

Despite the strong evidence for EBPs like A-CRA, low rates of SUD service delivery (7.6% ages 12–17 and 4.4% ages 18–25) [3] suggest that an even smaller portion of the ~ 10 million youth with SUD receive effective services. Behavioral health administrators and policymakers need research-based guidance on how to increase the availability of EBPs for youth SUD through targeted implementation efforts [1, 4–7]. To produce generalizable knowledge that can guide administrators and policymakers, researchers may examine the implementation strategies that help systems, organizations, and/or individual providers (e.g., clinicians, supervisors) successfully implement an EBP [8–10].

A recent review highlighted the importance of financing strategies [11], a type of implementation strategy that helps systems and organizations secure financial resources to cover implementation-related expenses. Grants and contract funding (e.g., from government or philanthropic funders) were commonly reported examples, although the review identified 23 financing strategies in total. Financing strategies are essential because EBP implementation is often a complex, long-term, and costly process [12–16]. For A-CRA specifically, previous research found that lack of stable funding was a major barrier to its implementation, despite favorable provider perceptions of the model [17–19].

Federal agencies have the influence and resources to potentially increase EBP delivery nationwide through financing strategies [6, 20]. Unfortunately, little is known about how federal financing strategies should be structured to maximize implementation outcomes for any EBP, behavioral health setting, or type of financing strategy [11]. For example, the US Substance Abuse and Mental Health Services Administration (SAMHSA) issued several rounds of grant funding that supported A-CRA implementation; first, “organization-focused” grants issued directly to treatment organizations, followed by “state-focused” grants issued to state substance use agencies. Treatment organizations provide direct clinical services and thus are the service delivery context for A-CRA implementation, whereas substance use agencies are state-level entities that support the publicly funded services delivered by treatment organizations. As one of the largest investments in EBP implementation to date, SAMHSA’s initiatives offer a natural experiment to compare the outcomes of organization-focused versus state-focused grants on a large scale, but so far, no such evaluation is publicly available.

The present study evaluated how well the two aforementioned SAMHSA grant strategies promoted A-CRA provider-level reach, one of the several key outcomes for evaluating the impact of implementation strategies [21, 22]. Reach is defined as the extent to which an EBP is used within a service system or community [23]. Many studies conceptualize reach at the service recipient or client level, but it is equally important to understand provider-level reach—i.e., the proportion of eligible clinicians and/or supervisors who start using an EBP (sometimes called “penetration” [22]), since implementation strategies often target organizations or providers (e.g., training). We defined provider-level reach as rates of A-CRA certification (which designates whether each provider adopted A-CRA and delivered it with adequate fidelity), in recognition that those other implementation outcomes (adoption and fidelity [21]) are preconditions for reach to be meaningful.

## Method

We describe this study using the Standards for Reporting Implementation Studies [24] (see Additional file 1). Relevant details are summarized here, but also see our published research protocol for more details [25]. All procedures were approved by the RAND Corporation IRB (Protocol #2020-N0887), including the use of data previously collected from organization-focused grantees [18, 19, 26] and new data collection from state-focused grantees.

### Study overview

The current study used a longitudinal, mixed-method [27] design to compare A-CRA reach between state-focused and organization-focused grant recipients. Leveraging SAMHSA grant initiatives that all targeted the same EBP (i.e., A-CRA) and provided the same implementation support (e.g., training and consultation) allowed for a natural experiment to examine the impact of the two financing strategies: organization-focused versus state-focused grants. Our research questions, detailed in Table 1, were as follows: (1) How did A-CRA reach rates differ by grant type? and (2) What factors influenced A-CRA reach outcomes?

**Table 1:** Mixed-method design for A-CRA provider-level reach outcomes study by research question

Design features	Research questions	
	(1) How did organization-level A-CRA reach rates differ by grant type?	(2) What factors influenced A-CRA reach outcomes?
Focus	Primary outcome (any A-CRA certification) Secondary outcomes (specific certification types) Descriptive variables for the certification process	Barriers and facilitators Moderators
Structure	QUAN	QUAL + QUAN
Function of mixed methods	n/a	Complementarity-elaboration: QUAL data provide depth of understanding to QUAN reach rates and moderator analyses
Hypothesis	State-focused > organization-focused for reach rates	Association between grant type and reach moderated by external support and internal leadership

*A-CRA* Adolescent Community Reinforcement Approach, *QUAN* quantitative, *QUAL* qualitative (all capital letters indicate the method is primary or co-primary in the design)

Given that the state-focused financing strategy was designed by SAMHSA to improve implementation outcomes beyond the organization-focused initiative, for research question 1, we tested SAMHSA’s hypothesis that reach would be higher among organizations participating in the state-focused grants relative to the organization-focused grants. Our design used a natural experiment, without random assignment of treatment organizations to the different grant types, and we prioritized producing externally valid evidence on the impact of each grant strategy in large-scale initiatives. This design does not fully isolate the effects of grant type on outcomes; however, we also tested the robustness of our findings against major internal validity threats with a series of sensitivity analyses.

We hypothesized for research question 2 that A-CRA reach outcomes would be moderated by two key factors shown to influence implementation: [28, 29] external financial support for services [13, 30] and internal leadership support [31, 32]. We used qualitative interview data to identify the barriers and facilitators of A-CRA implementation, providing a complementary qualitative data source on quantitatively measured reach rates in order to extend our understanding of how reach outcomes functioned in practice (a mixed-method complementarity-elaboration function [27]).

### Project context

The project design and hypotheses are grounded in the Exploration, Preparation, Implementation, Sustainment (EPIS) framework [28, 29], which describes how public service organizations implement EBPs by navigating a multi-phase process influenced by contextual factors across multilevel domains (innovation, inner context, outer context, bridging factors). In the exploration phase, the SAMHSA Center for Substance Abuse Treatment [33] selected A-CRA for implementation; other EBPs were also included in some initiatives, but we focused on A-CRA to minimize variation in outcomes due to innovation (i.e., EBP) characteristics. Briefly, A-CRA [2] is a psychosocial treatment model that uses cognitive-behavioral and family therapy techniques (e.g., functional analysis, communication skills) to replace factors supporting substance use with alternative activities and behaviors; it can be delivered in individual or group formats, with caregivers attending some sessions alone or with the youth. Chestnut Health Systems (CHS), the organization that developed A-CRA and conducts A-CRA training and research, created a certification protocol that supports high-fidelity delivery across providers through the use of a treatment manual, initial training (with behavioral role-plays), observed practice in delivering A-CRA, and consultation support and feedback until certification is achieved [34].

Through its grant-funded initiatives, SAMHSA provided funding, oversight, and leadership for A-CRA implementation to (i) four cohorts of organization-focused grantees across 26 states, awarded between 2006 and 2010, followed by (ii) four cohorts of state-focused grantees across 22 states, awarded between 2012 and 2017. During the preparation and implementation phases, organizations or states applied for, received, and executed SAMHSA grants. All grants targeted inner context (intra-organizational) factors at treatment organizations through training and certification for A-CRA clinicians and supervisors; leadership from treatment organizations selected which providers were available and interested to participate in the training. State-focused grants further targeted outer context (extra-organizational) factors at the state level and bridging factors that connected state substance use agencies and treatment organizations; with these changes, SAMHSA aimed to maximize the number of providers offering A-CRA in their states and, thus, enhance reach outcomes relative to organization-focused grants. Reach outcomes are the culmination of the implementation phase, i.e., grant funding period, after which the sustainment phase begins. Next, we describe the two grant types, and Table 2 details their characteristics.

**Table 2:** Characteristics of organization-focused and state-focused grant strategies

Characteristic	Grant type	
	Organization-focused	State-focused
SAMHSA grant initiatives included	Assertive Adolescent Family Treatment (AAFT), Juvenile Drug Court (JDC), Juvenile Drug Treatment Court (JDTC), Offender Re-entry Program (ORP), Targeted Capacity Expansion (TCE)	State Adolescent Treatment Enhancement and Dissemination (SAT-ED), State Youth Treatment (SYT), State Youth Treatment-Implementation (SYT-I), Youth Treatment-Implementation (YT-I), State Targeted Response (STR)
Grant recipient	Treatment organizations delivering substance use services, which applied directly for funding	State substance use agencies (sometimes referred to as departments) and treatment organizations designated by the state agency to receive funding
A-CRA training and certification purveyor	Chestnut Health Systems (contracted by SAMHSA)	Chestnut Health Systems and/or certified A-CRA supervisors acting as statewide trainers (contracted by state agencies)
Funding awarded to state agency	Not applicable	~ $3–4 million USD over 3–6 years
Funding awarded to treatment organization	~ $900,000 USD over 3 years	Similar to organization-focused grantees for demonstration site treatment organizations; provided by the state agency
Requirements to develop A-CRA infrastructure (e.g., funding, training)	Not applicable	Up to one-third of grant funds could be used for this purpose; expected activities varied by grant mechanism
EPIS domains and phases targeted	Inner context Implementation	Inner and outer Contexts, bridging Implementation and sustainment

*A-CRA* Adolescent Community Reinforcement Approach, *EPIS* Exploration, Preparation, Implementation, and Sustainment framework, *SAMHSA* US Substance Abuse and Mental Health Services Administration

#### Organization-focused grants

SAMHSA awarded ~ $900,000 USD (across a 3-year period) to each treatment organization. These grants supported A-CRA implementation by paying for A-CRA delivery, A-CRA supervision, and other related activities. Our prior work showed that grantees commonly experienced initial success in implementing A-CRA with fidelity and reducing youth substance use, but difficulty sustaining the model post-funding [17–19].

#### State-focused grants

SAMHSA awarded ~ $3–4 million USD each (across a 3- to 4-year period; sometimes extended to 6 years) to state agencies that administered publicly funded SUD services. One-third of the grant funds paid for states to develop EBP-focused infrastructure, such as funding and training. Some grants required that state agencies propose “demonstration site” organizations, which established partnerships with the state agency and implemented A-CRA first before the state began broad dissemination to other organizations.

### Participants

#### Treatment organizations

We used CHS certification records, conducted semi-structured interviews, and collected survey data from clinicians and supervisors at treatment organizations that implemented A-CRA (inner context). For interviews and surveys, we sampled organizations within the 5-year period following the completion of grant funding. Individuals who were currently or recently employed as a clinician or clinical supervisor responsible for youth SUD treatment were eligible to participate; those knowledgeable about A-CRA implementation were preferred.

For organization-focused grantees, all 82 eligible organizations (in 27 states) were invited for interviews. Organizations that participated in state-focused grants were more numerous, so we created a comparable sample of 82 organizations (in 18 states) by randomly selecting up to five organizations per state, including up to three “demonstration sites.” To ensure a comparable sample size for state-focused grants, we re-selected 17 organizations across nine states (because five had closed, 11 no longer provided youth treatment, and one had merged with a site that was already part of our sample); CHS records showed that selected and not-selected organizations did not differ on A-CRA certification rates (*χ*^2^_(1)_ = 1.45, *p* = .23) or turnover rates (*χ*^2^_(1)_ = 0.82, *p* = .36), even after replacement.

The interviewed sample included 154 organizations (94% of the 164 selected), with 249 provider participants (39% clinicians, 33% supervisor-clinicians, 28% supervisors). For CHS records, data from all trained providers (566 organization-focused, 417 state-focused) at the selected organizations were included. For 11 organizations that had providers trained under both grant types, we used the first (organization-focused) observation in our primary analyses, but we explored other ways of handling those organizations in sensitivity analyses (described later).

#### State substance use agencies

We also interviewed administrators from the state agencies (outer context) that received SAMHSA state-focused grants. Participants were currently or recently employed in a relevant leadership position and were eligible within the 5-year period after funding ended. Thirty-two administrators from 100% of the 18 eligible states participated; we conducted group interviews when multiple individuals from the same state agency participated.

### Data sources and collection procedures

Table 3 summarizes the data collection activities and specific measures used to evaluate reach outcomes. CHS maintains a database of SAMHSA grantees, from which we extracted administrative records used to contact eligible clinicians, supervisors, and state administrators for interviews and surveys. Recruitment was done via email, phone, and/or mail, and data were collected via telephone or Microsoft Teams for interviews and Confirmit for surveys. Informed consent was obtained for each activity, no personally identifiable information was collected, and we de-identified participants’ data upon collection. We offered $25 USD for each interview and survey, although some participants (mostly state agency administrators) considered participation part of their job and declined compensation.

**Table 3:** Measures used to compare state-focused versus organization-focused grants on their A-CRA provider-level reach outcomes

Activity	Measures	Participants	Time	Payment	Research Questions^a^
Administrative data from CHS	Proportion certified (any, first-level, full, supervisor, TAY first-level/full) Descriptive data about fidelity scores	Providers^b^(164 organizations in 35 states, *n* = 983)	n/a	n/a	RQs 1, 2 RQ 1
Semi-structured interviews (wave 1)			45–60 min	$25 USD	RQ 2
	Organization-reported barriers/facilitators of A-CRA implementation	Providers^b^ (154 organizations, *n* = 249)			
	State agency-reported barriers/facilitators of A-CRA implementation	State substance use agency administrators^c^ (18 states, *n* = 32)			
Web survey (wave 1)			~25 min	$25 USD	RQ 2
	PSAT funding stability [35, 36]	Providers^b^ (138 organizations, *n* = 202)			
	PSAT organizational capacity [35, 36]﻿				

*A-CRA* Adolescent Community Reinforcement Approach, *CHS* Chestnut Health Systems, *PSAT* Program Sustainability Assessment Tool, *RQ* research question, *TAY* transition-age youth

^a^See Table 1 for the details of the research questions

^b^Includes clinicians and/or supervisors and includes data collected in the previous project from organization-focused grantees as well as newly collected data from state-focused grantees

^c^Only collected for state-focused grants

#### A-CRA certification records

CHS created a database of certification outcomes for all sampled treatment organizations, which specified any certifications achieved by each clinician or supervisor trained in A-CRA during the grant period as well as relevant descriptive information.

#### Semi-structured interviews

Interview protocols used a combination of open-ended questions and standardized probes [37]. Protocols were tailored to each participants’ role and, for treatment organizations, whether the organization was still delivering A-CRA (see Additional file 2 for protocols). The interviews explored state- and/or organization-level approaches to implementing A-CRA, how A-CRA implementation was supported during the funding period, sustainability planning, and (state administrators only) state infrastructure developed through their grant, as well as sustainment-focused questions for future analysis. Interviews were audio-recorded and transcribed (with transcripts de-identified) and lasted approximately 45–60 min.

#### Provider surveys

Following each provider interview, respondents were sent a web-based survey that collected standardized measures (including potential moderating factors) and other descriptive information (see Additional file 3 for all survey items), which again include sustainment-related questions not analyzed here. The surveys took approximately 25 min to complete. Of the treatment organizations interviewed, 90% had at least one survey completed.

### Measures

#### Grant characteristics

Participants’ involvement in SAMHSA grants was determined from CHS records and later verified during the consent process and initial interview questions. Characteristics recorded included grant type, start and end years, and length.

#### Reach outcomes

We used CHS administrative records to define reach [23] as the proportion of providers in a treatment organization that were certified in A-CRA by the end of the grant period, out of all individuals who were eligible for certification (i.e., completed the initial training). A-CRA clinician certification is based on proficient demonstration of A-CRA procedures (i.e., clinical techniques or activities). “First-level” certification indicates proficiency in nine core procedures and is required for independent delivery of A-CRA; optional “full” certification indicates proficiency in an additional 10 procedures (19 total) [2]. Additional optional certifications are available to indicate proficiency with transition-age youth (TAY; ages 18–25), which adds two other procedures to the first-level and full certifications, and in A-CRA supervision, which is based on demonstrated ability to supervise within the A-CRA model and rate A-CRA procedures and session fidelity. We constructed six reach variables representing the proportion of participants who achieved any certification—which was the primary outcome of interest—as well as first-level, full, TAY (first-level or full), or supervisor certifications.

We also examined descriptive variables including time to certification, whether the participant left the organization (i.e., turnover status), and fidelity scores for rated sessions resulting in passing scores. It was important to verify that each certified individual achieved fidelity (i.e., adherence to the A-CRA model with adequate competence or skill), since fidelity is associated with client SUD outcomes [34, 38–43]. An average rating of ≥ 3 out of 5—based on a review of recorded sessions—is required across the relevant procedures for a given A-CRA certification [34, 38–43].

#### Moderators

We proposed two prespecified moderators of A-CRA reach outcomes [25], but the survey measures for these moderators could not be analyzed due to difficulties with scoring responses (“external support” measure) or high missingness at the organization level (leadership measure, which was only administered to clinicians). Instead, we used similar subscales from the Program Sustainability Assessment Tool, a measure of eight EBP sustainment capacity domains [35, 36]; this measure was administered to all participants, so all organizations with survey responses had data for the analysis (*n* = 138). We used the funding stability subscale to capture external financial support for A-CRA and the organizational capacity subscale for internal leadership support of A-CRA.

#### Descriptive measures of A-CRA barriers and facilitators

We collected other interview and survey data that measured constructs from EPIS. Innovation measures captured participants’ perceptions of and attitudes toward the A-CRA model. Inner context measures described the treatment organizations delivering A-CRA, outer context measures described extra-organizational factors that affected A-CRA reach, and bridging factors described factors that linked inner and outer contexts (including grant-funded activities). We asked about the impacts of the ongoing COVID-19 pandemic in the state-focused sample, but those data were only applicable to reach outcomes in three states with active state-focused grants in 2020.

### Analysis plan

#### Quantitative data analysis

To compare reach rates between grant types, we fit a series of multivariable linear regression models with reach rate as the organization-level dependent variables. Each regression included grant type and several covariates: turnover rate, number of individuals trained, number of supervisors who pursued certification, grant end date, and quarters of grant funding. These covariates helped address threats to interpreting the “grant type” variable (e.g., grant end date controls for time trends) and account for important factors identified in our qualitative analyses (e.g., turnover). Moderator variables (funding stability, organizational capacity) were entered at the organization level, along with an interaction term with grant type. Moderator variables were averaged for all ratings at that organization; if moderator items were missing, we imputed their mean value from other organizations with the same A-CRA sustainment status.

The initial examination of reach rates revealed that both TAY outcomes had sample sizes too small for an adequately powered analysis (*n*s = 34 for first-level TAY, 29 for full TAY), per our initial power calculations [25]. Therefore, we report only descriptive statistics for those outcomes and focused our modeling on the following four outcomes: any, first-level, full, and supervisor certification. Furthermore, any certification was the primary outcome (it subsumes the other certifications), so we used the threshold *p* < .05 for statistical significance rather than controlling the experiment-wide error rate.

#### Supplemental and sensitivity analyses

We tested alternate model specifications such as beta regression, robust standard errors, and state-level fixed effects (with and without clustering standard errors), but the findings changed minimally so we report the basic models. We also conducted a series of sensitivity analyses that characterized threats to internal validity in our design. Specifically, we examined (a) observed secular trends using a non-equivalent dependent variable [44, 45], delivery of buprenorphine, and an evidence-based medication for opioid use disorder and (b) patterns of findings across sub-samples that lent insight into the impacts of grant type (for example, we examined outcomes for demonstration sites vs. other state-focused grantees). All details of these analyses and the findings are reported in Additional file 4.

#### Qualitative analysis of interviews

We analyzed interviews using conventional content analysis [46] to identify the barriers and facilitators to A-CRA reach during grant funding periods. As a starting point for our analyses, we used a codebook from a prior published analysis of interviews with organization-focused grantees [17]; the second author of this study led that work.

To analyze the state-focused interview transcripts, we developed a codebook in Microsoft Excel with code definitions and exemplars [47], organized by the four EPIS domains (outer and inner contexts, bridging factors, and innovation characteristics) and further classified by determinant type of barrier and/or facilitator. We then used the NVivo qualitative software program to organize and analyze transcripts, iteratively adding newly identified codes to the codebook. Two research assistants coded most transcripts, but two interviewers also contributed. The lead author provided feedback on the initial coding and reviewed specific passages on request throughout; the coding team also met biweekly to discuss the progress and refine the coding as needed.

Finally, we developed written summaries of the content of codes (with exemplar quotes), focusing on implementation-specific barriers and facilitators. The lead author and second author (who led the previous analysis [17]) incorporated details about the similarities and differences between the barriers and facilitators that participants described for each grant type. All co-authors reviewed and helped further revise the summaries to ensure credibility and completeness.

## Results

### Reach outcomes

Table 4 reports reach rates for each A-CRA certification; for example, the average percentage of eligible individuals who received any certification was 72% for treatment organizations under organization-focused grants versus 36% for organizations under state-focused grants. The table also reports descriptive statistics for covariate, moderator, and certification process variables. Note that the average fidelity scores for certified providers ranged between 3.5 and 4.1, with all values ≥ 3 (i.e., adequate fidelity).

**Table 4:** Descriptive statistics for organizations that implemented A-CRA through organization-focused and state-focused grants

Domain	Variable	Descriptive statistics (*M* and *SD*) by grant type	
		Organization-focused (*n* = 82 in 27 states^a^)	State-focused (*n* = 82 in 18 states^a^)
A-CRA provider-level reach rates (i.e., percentage certified)	Any certification	72% (21%) *n =* 82	36% (33%) *n =* 82
	First-level certification	62% (20%) *n =* 82	36% (33%) *n =* 82
	Full certification	29% (25%) *n =* 82	30% (33%) *n =* 82
	Supervisor certification	66% (34%) *n =* 81	40% (41%) *n =* 68
	TAY first-level certification	100% (0%) *n =* 7	43% (43%) *n =* 27
	TAY full certification	100% (0%) *n =* 2	41% (43%) *n =* 27
Covariates (for full sample)	# of providers trained (clinicians, supervisors)	6.9 (3.9)	5.1 (4.3)
	# of supervisors who pursued certification	2.2 (1.3)	1.7 (1.5)
	Turnover rate	46% (25)	32% (32)
	Grant end date (in quarters since 2000)	50.3 (5.3) (approx. 2012)	74.7 (5.5) (approx. 2018)
	Length of grant funding (in quarters)	13.8 (5.0) (3.45 years)	17.6 (5.4) (4.40 years)
	# of organizations that trained under both grant types	11 (13.4% of sample)	N/A^b^
	# of demonstration sites	N/A	43 (52% of sample)
Moderators	PSAT funding stability^c^	3.4 (1.3) *n =* 68	3.4 (1.5) *n =* 60
	PSAT organizational capacity^c^	5.1 (1.3) *n =* 68	3.9 (1.4) *n =* 60
Certification process—clinician	Fidelity score^d^	3.8 (0.14) *n =* 58	4.1 (0.14) *n =* 52
	Number of first-level sessions rated	21.1 (6.0) *n =* 81	19.2 (5.4) *n =* 55
	Number of full sessions rated	33.2 (8.4) *n =* 58	24.3 (5.6) *n =* 52
	Time to first-level certification (in weeks)	32.7 (9.9) *n =* 81	50.2 (22.4) *n =* 55
	Time to full certification (in weeks)	61.3 (23.0) *n =* 58	62.9 (22.9) *n =* 52
Certification process—supervisor	Fidelity score^d^	3.8 (0.36) *n =* 72	3.5 (0.35) *n =* 38
	Number of sessions rated	3.2 (1.7) *n =* 72	2.5 (1.7) *n =* 38
	Time to supervisor certification (in weeks)	32 (16) *n =* 72	54 (33) *n =* 38

*A-CRA* Adolescent Community Reinforcement Approach, *TAY* transition-age youth, *PSAT* Program Sustainability Assessment Tool

^a^There were 10 states that were represented in both the organization- and state-focused samples, 17 states only represented in the organization-focused sample, and 8 states only represented in the state-focused sample, for a total of 35 states represented

^b^When organizations trained providers under both grant types, we included the first (organization-focused) observation in the primary analysis. See Additional file 3: Table S1 for the sensitivity analyses that handled these observations differently

^c^PSAT scores range from 1 to 7, with higher scores indicating higher sustainment capacity

^d^Fidelity scores range from 1 to 5, with scores ≥ 3 indicating competency in A-CRA

Table 5 presents estimates from regression models which examined whether grant type (organization-focused vs. state-focused) was associated with reach outcomes. Controlling for covariates, the rates of any A-CRA certification in organization-focused grants were 27 percentage points higher (*p* = .01), and the rates of first-level certification were 23 percentage points higher (*p* = .04), compared to state-focused grants. These models explained a large portion of the variance in outcomes (*R*^2^ = .33 and .22). There were no significant differences in full or supervisor certification rates.

**Table 5:** Regression models for association of grant type and A-CRA provider-level reach outcomes

Variable	Estimated association with reach outcome (*β*, *SE*)			
	Any (*n* = 164)	First-level (*n* = 164)	Full (*n* = 164)	Supervisor (*n* = 149)
State-focused grant (vs. organization-focused)	− 0.27 (.11) *p =* .01^*^	− 0.23 (.11) *p =* .04*	− 0.15 (.11) *p =* .18	− 0.06 (.15) *p =* .69
# of providers trained	0.01 (.01) *p =* .39	0.01 (.01) *p =* .26	0.00 (.01) *p =* .95	0.02 (.01) *p =* .02*
# of supervisors who pursued certification	− 0.02 (.02) *p =* .31	− 0.02 (.02) *p =* .25	− 0.02 (.02) *p =* .44	− 0.09 (.03) *p* < .01**
Turnover rate	− 0.2 (.08) *p =* .01^*^	− 0.17 (.08) *p =* .03^*^	− 0.15 (.09) *p =* .08	− 0.02 (.13) *p =* .85
Grant end date	0.00 (.00) *p =* .33	0.00 (.00) *p =* .61	0.01 (.00) *p =* .16	− 0.01 (.01) *p =* .31
Length of grant period	0.00 (.00) *p =* .62	0.00 (.00) *p =* .72	0.00 (.00) *p =* .69	− 0.01 (.01) *p =* .29
Constant	1.05 (.22) *p* < .01^**^	0.82 (.22) *p* < .01^**^	0.12 (.23) *p =* .62	1.10 (.32) *p* < .01^**^
*R*^2^	.33	.22	.05	.17

*A-CRA* Adolescent Community Reinforcement Approach

^*^Significant at *p* < .05 level

^**^Significant at *p* < .01 level

### Sensitivity analyses

The detailed results of the sensitivity analysis regression models are reported in Additional file 4. Briefly, we found that observed differences in reach outcomes did not result from confounding temporal trends or the characteristics of states or organizations but instead were strongly associated with grant type. For the non-equivalent dependent variable, grant type was not associated with the likelihood of an organization delivering buprenorphine. For the sub-sample analyses, the pattern of results was similar to the main analysis across sub-samples.

### Moderator analyses

Neither tested moderator showed significant effects for the sub-sample of organizations (84%) with survey responses. Coefficients for the main effects on any certification were as follows: funding stability, 0.03 (*p* = .18), and organizational capacity, 0.00 (*p* = .91). Interaction effects with grant type were as follows: funding stability, 0.01 (*p* = .88), and organizational capacity, 0.07 (*p* = .07).

### Barriers and facilitators identified in qualitative interviews

Table 6 reports the demographic characteristics of the providers who completed the interviews. For state administrators, we only collected information about professional experience, with the primary respondent(s) in the interview generally providing the information (21 of 32 administrators [66%] responded): experience with administering substance use treatment services ranged from 1 to 30 years (*M* = 14.2, *SD* = 8.3), and when relevant, experience providing substance use treatment services ranged from 1 to 25 years (*n* = 14; *M* = 11.7, *SD* = 6.4).

**Table 6:** Demographic characteristics of provider interview samples by grant type

Characteristic	Organization-focused (78 organizations in 27 states)	State-focused (76 organizations in 18 states)
Role (*n*, %)	*n* = 134	*n* = 115
Clinician	55 (41%)	42 (37%)
Supervisor-clinician	44 (33%)	38 (33%)
Supervisor	35 (26%)	35 (30%)
Age (*M*, *SD*)	41 (11.79)	45 (10.83)
Not reported (*n*, %)	25 (19%)	21 (18%)
Gender (*n*, %)		
Female	81 (61%)	69 (60%)
Male	35 (26%)	26 (23%)
Not reported	18 (13%)	20 (17%)
Race (*n*, %)		
White	81 (61%)	77 (67%)
Black	14 (10%)	11 (10%)
Asian or Pacific Islander	2 (1%)	1 (1%)
American Indian or Alaska Native	6 (5%)	1 (1%)
Others (includes multiracial)	11 (8%)	4 (3%)
Not reported	20 (15%)	23 (20%)
Ethnicity (*n*, %)		
Hispanic or Latino/a/x	37 (28%)	10 (9%)
Not Hispanic or Latino/a/x	78 (58%)	85 (74%)
Not reported	19 (14%)	20 (17%)
Education (*n*, %)		
Some college, associate's, or bachelor’s degree	33 (25%)	11 (10%)
Graduate degree (master’s or doctoral)	83 (62%)	84 (73%)
Not reported	18 (13%)	20 (17%)

From our coding of those interviews, we summarized the key barriers and facilitators to A-CRA reach. Findings are presented by EPIS domain (innovation, inner context, outer context, bridging factors). The written summaries give an overview of all findings but emphasize the key differences between contextual influences on organization-focused and state-focused grants. Table 7 lists each of those key differences in further detail, highlighting how each finding contributed to our interpretation of quantitative reach outcomes. Finally, we provide an expanded version of that table in Additional file 5, which includes summaries of all identified codes (with illustrative quotes) and details of which participants reported each code by grant type (organization-focused, state-focused) and role (provider, state administrator); most codes were reported by all grant types and roles.

**Table 7:** Summary of key barriers and facilitators to A-CRA provider-level reach outcomes from qualitative interviews

EPIS domain	Determinant code^a^	Brief description and exemplar quote^b^	Contribution to interpreting reach outcomes within mixed-method design
Innovation	Lack of fit with population (B)	Some providers noted A-CRA was not culturally appropriate for their population, often due to disparities in access to resources (e.g., low-income or minoritized racial/ethnic groups). Others noted fit problems due to the clinical population, such as not working as well with for youth in residential care or with severe mental health problems.	Participants from state-focused grants reported more frequent and serious barriers around fit of A-CRA with their client population compared to participants from organization-focused grantees. This suggested there was a greater likelihood of fit issues when implementing treatment organizations were encouraged to implement by state agencies rather than making the decision to implement themselves, which may have decreased certification rates under state-focused grants.
		*Exemplar quote for contribution*: “One of the difficulties with A-CRA … is that there is a big component of getting kids involved in more pro-social activities. There’s a lot of barriers to that culturally and otherwise and financially for folks…. so there aren’t a lot of resources for things for young people to do, especially in the after school hours, or the money to do a whole lot.” (SF, P)	
Inner context	Strategic planning (F)	Treatment organizations described engaging in strategic planning to promote adoption and use of A-CRA, with an emphasis on fully successful implementation and preparing for sustainment after the SAMHSA funding ended.	Engagement in strategic planning to promote adoption and use of A-CRA was uniquely describe by organization-focused grantees as a facilitator; state-focused grantees described minimal strategic planning. This may have been a natural extension of the strategic planning process that went into organization-focused grantees applying for and securing their grants through a competitive application process.
		*Exemplar quote for contribution*: “Part of planning was also to work with state legislature, [Department of Children and Families], and Court Services Division to let them know about [A-CRA] and seek longer-term state-based funding for these services. We have also done some work with [Department of Social Service]… to advocate for this model as being paid for in full.” (OF, P)	
Outer context	State leadership support (F/B)	Having state agency staff dedicated to working with treatment organizations on A-CRA helped facilitate project administration, encouraged buy-in at the organizational level, and helped move providers through the certification process, whereas participants in other states noted their state agency did not encourage use of A-CRA and the model was not marketed well in those states.	State leadership support was only relevant under state-focused grants. Some respondents described state substance use agency administrators as helpful in guiding the implementation process, which encouraged organizational buy-in; however, state administrators also encountered challenges to fulfilling their role, with the specific challenges varying from state to state (e.g., limited support from state leaders, decision-making power lying with local authorities, turnover of state agency staff). These challenges helped explain why state-focused grant activities were challenging to execute and resulted in lower average certification rates, despite the strengths of some state agency leadership.
		*Exemplar quote for contribution*: “We kept in really good contact with the people from the state … I remember the guy who was from the state department that I talked to about [A-CRA], we talked to each other at least, I would say every other week, at a minimum. So from the state department all the way down, I think everyone involved in the [state-focused grant] program was pretty supportive.” (SF, P)	
	Community beliefs about substance use (B)	Substance use issues were seen as a less serious problem in some communities—including among caregivers, partner organizations, and providers—decreasing youth referrals and engagement.	Such attitudes (community beliefs about substance use, adolescents not a priority group) were uniquely described by participants from the more recent state-focused grants. Some participants attributed changing attitudes to increased legalization of cannabis in the USA. Such trends may indeed have been occurring in US society, although we did not find strong evidence that they influenced A-CRA reach rates (e.g., the grant end date covariate in the models did not have a significant effect for any outcome). This finding may also reflect differences in the type of organization that implemented A-CRA through organization-focused versus state-focused grants, similar to the “lack of fit with population” code above.
	Adolescents not a priority group (B)	In some communities, adolescents were not viewed as a priority treatment group. Thus, state and federal funding was focused on adult populations instead of youth, limiting resources available for youth treatments like A-CRA.	
		*Exemplar quote for contribution*: “There’s been tremendous reduction in referrals to substance use treatment. And most of those, that reduction, is really due to the decline of children and teenagers, families requesting treatment for marijuana. I mean, it’s really significant. It’s a dramatic drop.” (SF, A)	
Bridging factors	State funding (F)	The money states provided to treatment organizations in state-focused grants was integral to funding training, certification, supervision, and the resources needed to fully understand and deliver A-CRA with clients.	These activities (state-led training activities and comprehensive funding for A-CRA) were identified as major facilitators for state-focused grants, because they allowed for statewide coordination of activities. Despite the facilitators not leading to higher certification rates, they provided insight into the potential that SAMHSA and state agencies saw in pursuing state-focused grants as a financing strategy. However, it was also the case that organization-focused grants provided similar benefits as state-focused grants (i.e., training and comprehensive funding) through a more direct mechanism, albeit only to individual organizations.
	State-led Training Activities (F)	The partnership between states and Chestnut Health Systems to provide initial A-CRA training was viewed as informative to providers and state administrators, and helped them implement the model.	
		*Exemplar quote for contribution*: “One of the most important things that [state agency] can provide is accessibility to training and evidence-based models of treatment. My [organization] is a nonprofit. We serve an underserved community. When SAMHSA, through the states, is able to offer us access to training, consultation, and support, that’s an important resource.” (SF, P)	
Bridging factors, continued	Intensive support for organization-focused grants (F)	Many participants found the support provided by SAMHSA organization-focused grants to be of good quality.	Organization-focused grantees’ experiences differed in that they worked with SAMHSA directly, a collaboration often described as helpful but also as intensive and overwhelming. Combined with these organizations tending to exhibit more strategic planning and better fit between A-CRA and their climates and communities, the direct relationship with SAMHSA created by organization-focused grants could have lent greater support for increasing certification rates than state agencies were able to provide. These organizations did report more burden due to assessment and reporting requirements of the organization-focused grants, but those factors were not closely related to the A-CRA certification process—consistent with the higher certification rates under organization-focused grants.
	Assessment tool was cumbersome (B)	The GAIN (Global Assessment of Individual Needs), an assessment SAMHSA required organization-focused grantees to collect and report, was frequently seen as overly complex and burdensome.	
	Too much reporting/paperwork (B)	Reporting and paperwork related to administration of federal grants were seen as a barrier to using organization-focused grants	
		*Exemplar quotes for contribution*: “I think SAMHSA provided a lot of good training with the [A-CRA provider organizations] involved… I felt like there was a lot of support. It helped us get through what we needed to do.” (OF, P) “It was a very intensive-labor process, and it was monitored closely by SAMHSA … you would not be able to sustain that level of involvement per client. You would have to cut down on all the reporting.” (OF, P)	

See Additional file 5 for the details of all determinants identified, including information about which participant types contributed that determinant and additional exemplar quotes

*A-CRA* Adolescent Community Reinforcement Approach, *EPIS* Exploration, Preparation, Implementation, and Sustainment framework, *GAIN* Global Assessment of Individual Needs, *SAMHSA* US Substance Abuse and Mental Health Services Administration

^a^Key for determinant types: B, barrier; F, facilitator; F/B, facilitator or barrier (depending on the circumstances)

^b^Key for participant type who provided exemplar quote: OF, organization-focused grants; SF, state-focused grants; P, providers (clinicians and/or supervisors); A, state administrators

#### Innovation: characteristics of A-CRA

Respondents described numerous innovation factors as strengths of A-CRA, such as its content and procedures, structured format, and evidence-based status, and the training and certification support offered by CHS. These facilitators were often endorsed regardless of an individual’s certification status, though some respondents who stopped using A-CRA described its being overly structured and inflexible as a barrier. Respondents also broadly described the A-CRA certification process as complex and burdensome (whether they completed it or not), which was a major barrier to reach. A key difference between grant types was that state-focused grantees reported more frequent and serious barriers around fit of A-CRA with their client population compared to organization-focused grantees; across grant types, A-CRA being a good fit for the youth and families served was considered an important facilitator. Overall, providers had more detailed responses about A-CRA characteristics, though state administrators reported similar perceptions based on their discussions with providers.

#### Inner context: treatment organization factors

Respondents across roles identified organizational characteristics that facilitated reach of A-CRA, including leadership, supervisors, and clinicians who supported the model; “champions” who helped provide resources and encouraged certification; and organizational policies that aligned with A-CRA’s use (e.g., availability of client incentives). Conversely, the lack of support from key individuals (often related to the negative perceptions described under the innovation domain) and misalignment with organizational policies (e.g., strict time requirements for direct service delivery) created barriers to A-CRA reach. Turnover of providers and leaders was another major barrier, especially when it disrupted support for A-CRA or required the replacement of certified providers. Finally, one prominent facilitator uniquely described by organization-focused grantees was an engagement in strategic planning to promote the adoption and use of A-CRA. State-focused grantees described minimal strategic planning around A-CRA, so this facilitator was rarely present for them.

#### Outer context: community, state, and national factors

State administrators provided rich perspectives on outer context factors that influenced A-CRA reach, though providers commented on similar factors. Across grant types, states’ policy contexts influenced A-CRA reach; for example, policies that encouraged the provision of EBPs facilitated A-CRA use, whereas a common barrier was limited reimbursement for non-service components of A-CRA (e.g., certification and supervision activities). Among state-focused grantees, some respondents described state substance use agency administrators as helpful in guiding the implementation process, which also encouraged organizational buy-in. However, state administrators also described challenges to fulfilling that role, with the specific challenges varying from state to state (e.g., limited support from state leaders, decision-making power lying with local authorities, turnover of state agency staff). Finally, some state-focused grantees also described attitudes among caregivers, community members, and government agencies that increasingly de-prioritized addressing youth substance use (perhaps related to increased legalization of cannabis)—which decreased youth referrals and engagement in A-CRA.

#### Bridging factors: treatment organization links to external resources

The most salient bridging factor for many respondents was their SAMHSA funding. For state-focused grants, state-led training activities and comprehensive funding for A-CRA training, certification, supervision, and delivery were identified as major facilitators of reach, bridging between outer context (state-focused grant) and inner context (enactment of grant activities at treatment organizations) through statewide coordination of activities. However, providers noted that some state administrators did not encourage the use of A-CRA or help identify referral sources, creating barriers to reach. A few states were able to extend efforts beyond training and funding into policy development (e.g., developing new funding mechanisms to support A-CRA) and broader implementation supports (e.g., statewide learning collaboratives); such activities promoted reach but were rare and challenging to execute due to aforementioned state policy barriers. Organization-focused grants provided similar benefits as state-focused grants (i.e., training and comprehensive funding) through a more direct mechanism, albeit to individual organizations only. Organization-focused grantees’ experiences differed in that they worked with SAMHSA directly, a collaboration often described as helpful but also as intensive and overwhelming (e.g., due to grant-related assessment and reporting requirements). Treatment organizations involved in state-focused grants typically had fewer requirements and less interaction with grant administrators (state or SAMHSA). Finally, providers considered their partnerships with other organizations for referrals and on-site service delivery (e.g., schools, juvenile justice) as important facilitators (or barriers, when ineffective) across grant types.

#### COVID-19 impacts

For the three states where the COVID-19 pandemic overlapped with their state-focused grant, participants described several impacts on A-CRA implementation. Effects on A-CRA delivery (innovation domain) were mixed; despite the benefit of increased use of telehealth as an accessible treatment modality, providers noted that some clients experienced connectivity and equipment issues, making it harder to engage them in A-CRA via telehealth. Respondents also described how the pandemic exacerbated existing barriers like staff turnover (inner context) and difficulties working with partners for A-CRA referrals (bridging factors).

## Discussion

Effective financing strategies are needed to support EBP implementation in SUD services. We compared two financing strategies from the SAMHSA on the implementation outcome of A-CRA provider-level reach (i.e., certification rates). The shift from organization-focused to state-focused grants was associated with a 27 percentage point decrease in provider certification rates at treatment organizations, counter to our hypothesis. This finding appeared to be driven by differences in required first-level certification, with rates of the optional full certification < 1/3 in both grant types. We used a rigorous non-experimental design, with extensive sensitivity analyses supporting our interpretation that grant type was the major contributor to variation in reach, and confounding temporal, state, or organizational factors did not explain the findings.

Interviews with providers and state administrators suggested that achieving A-CRA certification was challenging and resource-intensive regardless of the type of financing strategy. Barriers and facilitators spanned all EPIS domains, as well as the construct of interactions and relationships during the implementation process (e.g., need for alignment among individuals in different roles within the inner context, partnerships with outer context organizations), which has rarely been reported in studies using EPIS [28, 29]. Importantly, organization-focused grantees benefitted more from certain facilitators, such as strategic planning around A-CRA and more intensive involvement with SAMHSA, whereas state-focused grantees encountered certain barriers more frequently, such as difficulties for state administrators in executing implementation support and engaging with treatment organizations or A-CRA being perceived as a poor fit for the organization’s client population. These qualitative findings helped lend valuable insight into why reach rates were lower under state-focused grants—especially since we could not identify quantitative moderators.

We designed this mixed-methods study to provide an in-depth understanding of financing strategies and identify the practical implications for policymakers. The findings contradicted our hypothesis, which makes it even more imperative to use this research evidence to inform EBP financing strategy design. Indeed, SAMHSA created the state-focused grant mechanisms in response to concerns about organization-focused grants, which in our earlier work [17–19] had declining rates of sustainment—57% of organizations discontinued A-CRA by 3 years post-grant. Therefore, policymakers need to consider the strengths and limitations of both types of financing strategies when deciding which to use or which warrants scalability—as well as consider other financing strategies not tested here. We plan to conduct focus group discussions with state and federal policymakers to help identify implications of our findings; for example, we will ask policymakers what strategic planning strategies could help state agencies best navigate systemic challenges (e.g., state policies, budget priorities) when scaling up EBP initiatives.

State-focused grants might have additional benefits not identified in our organization-level analysis. Reach at the state level (i.e., number of certified A-CRA providers relative to population of youth with SUD) could be higher in state-focused grants, which trained a much larger number of organizations than received organization-focused grants. Another important future direction is our planned state-level case study analyses [48, 49], in which we will incorporate additional data sources (e.g., state-level reach rates, documents provided by state administrators) to compare and contrast states’ state-focused grant activities and outcomes [25].﻿ The effectiveness of state-focused grants likely depends on each state’s policy context—which can vary dramatically [50]. This conclusion is supported by our findings that certain states were successful at developing infrastructure to support ongoing A-CRA implementation (e.g., new funding mechanisms).

It is also possible that state-focused grants produced higher A-CRA sustainment rates, as SAMHSA intended, among certified providers—though this seems unlikely given that successful initial implementation (e.g., reach) provides the necessary foundation for later sustainment [51]. Responding to calls to better articulate strategies for EBP sustainment [52], we are now measuring A-CRA sustainment among the state-focused versus organization-focused grant-funded programs. Ultimately, we expect that the financing strategies with the greatest policy impact will be those that produce both widespread and long-term implementation of EBPs, maximizing public health impact [53].

Our study builds on a small but emerging area of research on provider-level reach rates as an implementation outcome. Two previous studies examined provider reach for SUD-specific treatments—contingency management at an opioid treatment program [54] and alcohol and opioid use disorder treatments in a collaborative care service delivery model [55]—but examined only one or two implementation sites. Other studies examined system-level reach rates of youth EBPs including multidimensional family therapy for SUD and behavior problems [56], parent-child interaction therapy for behavior problems in young children [57], and six different mental health EBPs fiscally mandated for adoption in Los Angeles County [58, 59]. None of these studies compared reach rates between alternate implementation strategies, but all of the system-level studies identified financing strategies as important for sustaining EBPs in public behavioral health systems. The Los Angeles County study is a rare example of research examining implementation outcomes (including reach) from financing strategies [58]. The current study went beyond that county-level analysis by examining reach outcomes from different federal-level financing strategies (grants), for an SUD-focused EBP, and across various states and treatment organizations.

Researchers hoping to conceptualize their own studies of financing strategies in behavioral health, medicine, public health, and/or prevention may find it instructive to consider recently published recommendations from a policy adaptation of the EPIS framework [60]. Those recommendations were to specify the function and forms of the policies of interest (e.g., what resources state-focused and organization-focused grants provided, how those resources related to A-CRA reach outcomes); describe phases of policy implementation across inner and outer contexts (e.g., EPIS guided our data collection, analysis, and interpretation) and bridging factors [61] (of which financing strategies are one example [62]); and identify the temporal roles of policy actors such as policymakers and administrators (e.g., we distinguished barriers, facilitators, and outcomes in implementation vs. sustainment phases). We have not yet addressed the sixth recommendation, considering outer and inner context adaptations, but we intend to assess how contexts could be adapted to support A-CRA reach in the policymaker focus groups.

Despite its strengths, this study had several limitations. First, this work cannot directly inform efforts to improve the feasibility of the A-CRA certification process (e.g., identifying pragmatic fidelity measures [63]), which could make it easier to finance A-CRA as well as achieve full or supervisor certification. Second, we were not able to evaluate clinical outcomes of A-CRA; we confirmed that providers had adequate fidelity scores, which are associated with clinical outcomes [38, 39], but monitoring clinical impacts remains a major challenge for most EBP implementation initiatives. Third, we focused on provider-level A-CRA reach, but client-level reach must be separately considered; e.g., a recent analysis of the fiscally mandated EBP adoption in Los Angeles County found only 17% of youth in need of EBPs received them, with further disparities for minoritized ethnic groups and immigrants [64]. Fourth, we did not assess adherence to required grant activities, which could have introduced unmeasured variability within each grant type; our case studies will consider this issue to the extent possible. Finally, some aspects of our study design made the planned moderator analyses challenging, most notably missing data and interpretability issues (described under the “ Method” section) and survey responses being collected post-implementation, which may not represent how a factor operated during implementation. To balance that limitation, we maximized the explanatory power of qualitative data to identify influences and contextualize the experiences of our participants.

## Conclusions

This study is the first published comparison of reach outcomes between alternate financing strategies to support EBP implementation. This work fills a critical gap in how implementation research findings can guide administrators’ and policymakers’ investments in EBPs for youth SUD. Having identified critical issues in A-CRA reach and sustainment outcomes for state-focused and organization-focused grant financing strategies, we are poised to begin collaboratively exploring policy solutions. We hope that this work will encourage researchers, policymakers, and system/organization leaders to collaboratively study optimal ways to implement diverse EBPs in financially sustainable strategies, including the use of novel analytic approaches (e.g., non-equivalent dependent variables). Continued efforts will be essential to understand how these strategies operate with service and administrative systems and organizations to ultimately improve the effectiveness of providers and the lives of clients.

### Supplementary Information

**Additional file 1.** StaRI reporting checklist.

**Additional file 2.** All Wave 1 Interview Guides.

**Additional file 3.** All Wave 1 Survey Items.

**Additional file 4.** Supplemental Analyses for Quantitative Analysis of Provider-Level Reach Outcomes.

**Additional file 5.** Expanded Summary of All Barriers and Facilitators to A-CRA Provider-Level Reach Outcomes.

## Data Availability

The datasets analyzed for this study are available from the corresponding author upon reasonable request. Depending on the nature of the request, institutional data-sharing agreements may or may not be required.

## Abbreviations

A-CRA: Adolescent Community Reinforcement Approach
CHS: Chestnut Health Systems
EBP: Evidence-based practice
EPIS: Exploration Preparation Implementation Sustainment framework
GAIN: Global Assessment of Individual Needs
N-SSATS: National Survey of Substance Abuse Treatment Services
PSAT: Program Sustainability Assessment Tool
SAMHSA: Substance Abuse and Mental Health Services Administration
SUD: Substance use disorder
TAY: Transition-age youth
