# Supplementary material for: Comparing organization-focused and state-focused financing strategies on provider-level reach of a youth substance use treatment model: a mixed-method study
Source: Implement Sci. 2023 Oct 12;18:50. doi: 10.1186/s13012-023-01305-z (PMC10571404; doi:10.1186/s13012-023-01305-z)
Supplement: Supplementary file 2 — Additional file 2. All Wave 1 Interview Guides. [file 13012_2023_1305_MOESM2_ESM.docx]

# A-CRA Financing Project: STATE ADMIN INTERVIEW COVER SHEET

RESPONDENT ID(s):

RESPONDENT NAME(s):

STATE ID:

RESPONDANT STATE:

STATE AGENCY NAME:

DATE OF INTERVIEW: ______ / ______ /_______

TIME OF INTERVIEW (INTERVIEWEE TIME): _______ PT/MT/CT/ET

INTERVIEWER:

PHONE NUMBER FOR INTERVIEW:

E-MAIL:

A-CRA GRANTS RECEIVED:

SAT-ED START DATE: ______ / ______ /_______ END DATE: ______ / ______ /_______

SYT START DATE: ______ / ______ /_______ END DATE: ______ / ______ /_______

SYT-I START DATE: ______ / ______ /_______ END DATE: ______ / ______ /_______

STR START DATE: ______ / ______ /_______ END DATE: ______ / ______ /_______

YT-I START DATE: ______ / ______ /_______ END DATE: ______ / ______ /_______

other START DATE: ______ / ______ /_______ END DATE: ______ / ______ /_______

(if other, describe: ___________________ )

OTHER NAMES FOR THE SAMHSA/CSAT-FUNDED A-CRA IMPLEMENTATION PROJECT IN THIS STATE:

RELEVANT CASE NOTES:

***STATE ADMIN INTERVIEW VERBAL CONSENT***

(read prior to beginning interview/recording)

Hi [STATE ADMIN NAME],

This is [INTERVIEWER NAME] calling from RAND to speak with you about [STATE AGENCY NAME]’s experiences with implementing the Adolescent Community Reinforcement Approach (A-CRA) treatment model in [STATE]. Is this still a good time to complete your interview? To ensure confidentiality, make sure you are in a private, secure location for completing the interview.

>IF NO, reschedule.

>IF YES, great.

We would like a representative from [STATE AGENCY NAME] to answer these questions. Ideally, it should be someone knowledgeable about your A-CRA implementation efforts. Are you the right person at your agency to participate in this opportunity?

>IF NO: May I get the contact information of the best person to speak with about the state’s youth/adolescent substance use treatment programs (especially A-CRA implementation)? [RECORD CONTACT INFORMATION TO FOLLOW-UP]

>IF YES, thanks for confirming.

Before we begin, let me assure you that your responses to these questions will be held in strict confidence. In collaboration with Chestnut Health Systems, we are requesting interviews with state agency representatives in nearly 20 states that received funding from the SAMHSA Centers for Substance Abuse Treatment (CSAT) to deliver A-CRA. We will use information from the interviews to understand how different CSAT funding models influence the implementation and sustainability of A-CRA. We will also examine information provided by treatment organizations that implemented A-CRA as part of CSAT grants like yours through surveys and interviews. We will not attribute comments to specific individuals or programs in any of our reports or publications. Your responses will not be shared with your agency or with SAMHSA.

Today’s interview will last about 60 minutes. You will receive a $25 Amazon e-gift card as a thank you for your participation.

Your participation in this discussion is entirely voluntary. We would like to have your responses to all of the questions. However, if you’re uncomfortable with any question we can skip it, and you can stop the interview at any time. There are no right or wrong answers – we are interested in your perspectives and experiences. After the interview, we may follow up with you about sharing documentation related to your CSAT grant that would be useful for us to review. It will always be your decision whether to share. Finally, we would like to audio-record the interview to ensure that we capture everything that is said. We will destroy the recording once we confirm we have captured everything in our de-identified notes and transcripts. However, you can still participate in the interview even if you do not give permission to audio-record.

If you have any questions or want to discuss the project further at any time, you may always contact us at [PHONE] or at [EMAIL]. Furthermore, if you have questions about your rights as a research participant or need to report a research-related injury or concern, you can contact RAND's Human Subjects Protection Committee toll-free at (866) 697-5620 or email [hspcinfo@​rand.org](mailto:hspcinfo@​rand.org)​. If you contact the Committee, please reference Study #2020-N0887.

- **Do you have any questions?**
- **Are you willing to take part in this discussion?**

>IF NO: That is not a problem, thank you for your time.

>IF YES, great.

- **Is it ok with you if we audiotape this discussion?**

>IF NO: That is not a problem. I can take notes while we’re talking so I don’t miss anything important, though that means we might proceed through the interview more slowly than usual. I could also arrange for a colleague to take notes during the interview. [RESCHEDULE IF NEEDED]

>IF YES, perfect, let’s get started.

**Interview Protocol – STATE BEHAVIORAL HEALTH AGENCY ADMINISTRATOR**

1. Tell me a little bit about the state agency where you work. What types of services does your agency oversee or administer? (e.g., age groups, problem types, etc.)
2. What is your primary professional role(s) at your agency? (What are you responsible for overseeing?)
3. At the state agency, have you worked on any SAMHSA/CSAT-funded projects to disseminate A-CRA? Yes or No?

🞏 No **– *SKIP TO QUESTION 4***🞏 Yes – ***CONTINUE***

3A. Between what dates did you work on the project(s)? Your best guess is fine.

1. What do you think of A-CRA as a treatment for youth – does it meet the needs of the populations you serve? How so or why not? Of note, throughout this interview, by “youth” we typically mean ages 12 through 17 (or through age 24 if A-CRA training in your state included young adults).
2. What do think of A-CRA as a treatment for outpatient youth substance use treatment services – does it fit well into treatment organizations’ services and capabilities? How so or why not?
3. How has your state agency selected treatment organizations that you worked with to support A-CRA implementation?
4. Next, I’m going to list seven activities that your state agency might have engaged in its efforts to help provider organizations implement A-CRA. Please let me know whether your state agency engaged in each of these activities, and if so, any details you know about what was done and when.

7A. Providing training for organizations to implement A-CRA?

🞏 No
🞏 Yes - during the SAMHSA CSAT grant period

🞏 Yes - currently engaging in this activity

🞏 Yes - planning to continue over the coming year

🞏 Don't Know

IF YES, please describe *[PROBE AS NEEDED: What have those activities looked like in your state? Who and what have been involved? How has the activity changed since the grant funding period, if at all?]:*

7B: Directly funding organizations to implement A-CRA?

🞏 No
🞏 Yes - during the SAMHSA CSAT grant period

🞏 Yes - currently engaging in this activity

🞏 Yes - planning to continue over the coming year
🞏 Don't Know

IF YES, please describe *[PROBE AS NEEDED: What have those activities looked like in your state? Who and what have been involved? How has the activity changed since the grant funding period, if at all?]:*

7C: Developing state policies that promote use of A-CRA?

🞏 No
🞏 Yes - during the SAMHSA CSAT grant period

🞏 Yes - currently engaging in this activity

🞏 Yes - planning to continue over the coming year
🞏 Don't Know

IF YES, please describe *[PROBE AS NEEDED: What have those activities looked like in your state? Who and what have been involved? How has the activity changed since the grant funding period, if at all?]:*

7D: Supporting organizations’ sustainability planning for use of A-CRA?

🞏 No
🞏 Yes - during the SAMHSA CSAT grant period

🞏 Yes - currently engaging in this activity

🞏 Yes - planning to continue over the coming year
🞏 Don't Know

IF YES, please describe *[PROBE AS NEEDED: What have those activities looked like in your state? Who and what have been involved? How has the activity changed since the grant funding period, if at all?]:*

7E: Supporting organizations’ efforts to promote youth/family engagement?

🞏 No
🞏 Yes - during the SAMHSA CSAT grant period

🞏 Yes - currently engaging in this activity

🞏 Yes - planning to continue over the coming year
🞏 Don't Know

IF YES, please describe *[PROBE AS NEEDED: What have those activities looked like in your state? Who and what have been involved? How has the activity changed since the grant funding period, if at all?]:*

7F: Promoting organizations’ capacity for inter-organizational coordination?

🞏 No
🞏 Yes - during the SAMHSA CSAT grant period

🞏 Yes - currently engaging in this activity

🞏 Yes - planning to continue over the coming year
🞏 Don't Know

IF YES, please describe *[PROBE AS NEEDED: What have those activities looked like in your state? Who and what have been involved? How has the activity changed since the grant funding period, if at all?]:*

7G: Promoting organizations’ improving their computer systems or electronic health records?

🞏 No
🞏 Yes - during the SAMHSA CSAT grant period

🞏 Yes - currently engaging in this activity

🞏 Yes - planning to continue over the coming year
🞏 Don't Know

IF YES, please describe *[PROBE AS NEEDED: What have those activities looked like in your state? Who and what have been involved? How has the activity changed since the grant funding period, if at all?]:*

7H: Are there other activities or resources your state agency has provided to support A-CRA implementation?

🞏 No
🞏 Yes - during the SAMHSA CSAT grant period

🞏 Yes - currently engaging in this activity

🞏 Yes - planning to continue over the coming year
🞏 Don't Know

IF YES, please describe *[PROBE AS NEEDED: What have those activities looked like in your state? Who and what have been involved? How has the activity changed since the grant funding period, if at all?]:*

1. What implementation activities have you personally been involved in as part of state efforts to implement A-CRA?

8A. Providing training for organizations to implement A-CRA?

🞏 No
🞏 Yes

8B. Directly funding organizations to implement A-CRA?

🞏 No
🞏 Yes

8C. Developing state policies that promote use of A-CRA?

🞏 No
🞏 Yes

8D. Supporting organizations’ sustainability planning for use of A-CRA?

🞏 No
🞏 Yes

8E. Supporting organizations’ efforts to promote youth/family engagement?

🞏 No
🞏 Yes

8F. Promoting organizations’ capacity for inter-organizational coordination?

🞏 No
🞏 Yes

8G. Promoting organizations’ improving their computer systems or electronic health records?

🞏 No
🞏 Yes

8H. Other activities or resources your state agency currently provides to support A-CRA implementation?
🞏 No
🞏 Yes

1. How has your state agency obtained funding for continuing the A-CRA implementation activities or resources that you started during the SAMHSA CSAT grant? These are the activities and resources discussed in the previous series of questions – beyond paying for services. I’m going to read five options, please let me know which ones your agency has used.

9A. Internal funding from agency's budget?

🞏 No
🞏 Yes
🞏 Don't Know

IF YES, please describe:

9B. State budget appropriations?

🞏 No
🞏 Yes
🞏 Don't Know

IF YES, please describe:

9C. Block grant funds?
🞏 No
🞏 Yes
🞏 Don't Know

IF YES, please describe:

9D. Federal grants (like SAMHSA CSAT)?

🞏 No
🞏 Yes
🞏 Don't Know

IF YES, please describe:

9E. Payments from a special funding source (for example, a dedicated tax or bond, settlement fund, etc.)?

🞏 No
🞏 Yes
🞏 Don't Know

IF YES, please describe:

9F. Other sources of funding?

🞏 No
🞏 Yes
🞏 Don't Know

IF YES, please describe:

1. Currently, what funding options does your state agency offer provider organizations to support youth substance use treatment delivery in general?
2. What funding options, if any, does your agency currently offer to support provider organizations that deliver A-CRA specifically? By this, I mean funding for A-CRA activities or services begun during the SAMHSA CSAT grant.
3. The next few questions ask about how successful your state effort has been at implementing A-CRA. Please answer to the best of your knowledge, in the organizations/settings you would know about. I will ask you about success in three areas.

12A. How successful has your state effort been in training clinicians in A-CRA at provider organizations in your state?

12B. How successful has your state effort been in getting clinicians certified in A-CRA?

12C. How successful has your state effort been in youth receiving A-CRA in your state?

1. Tell me about any ways in which your state agency has collaborated with other entities to support A-CRA.

13A. Which of these collaborations is your agency continuing, if any? Why or why not?

1. Tell me about any efforts or planning at your state agency to obtain resources for continuing A-CRA beyond your state’s grant period. By resources, we mean things needed to support A-CRA such as money, staff, supervision, training, and A-CRA manuals.

14A. Which of these efforts or planning is your agency continuing, if any? Why or why not?

1. Can you please describe any state or federal policies that have supported A-CRA delivery? These could include funding and billing rules that support A-CRA delivery. Include policies at your state agency and other agencies.

15A. Which of these policies are currently supporting A-CRA delivery, if any?

1. What about state or federal policies that interfere with A-CRA delivery? (Including at your agency and other agencies)

16A. Which of these policies are currently impacting A-CRA delivery, if any?

1. Can you tell me about any pressure your state agency has experienced to continue delivering A-CRA or to discontinue its use?
2. Would you say provider organizations in your state are generally supportive or reluctant to use A-CRA? Can you give me an example?
3. Next, I would like to know what factors helped efforts to implement and sustain A-CRA in your state. Please let me know if these factors were important in your state, and if yes, how so.

19A. Would you say characteristics of A-CRA helped efforts to implement and sustain A-CRA in your organization? Yes or No?

🞏 No
🞏 Yes

IF YES, please describe: When was that factor most impactful? Was it during the CSAT grant funding period, after the CSAT funding ended, or throughout?

19B. Would you say characteristics of key individuals helped efforts to implement and sustain A-CRA in your organization? Yes or No?

🞏 No
🞏 Yes

IF YES, please describe: When was that factor most impactful? Was it during the CSAT grant funding period, after the CSAT funding ended, or throughout?

19C. Would you say characteristics of treatment organizations helped efforts to implement and sustain A-CRA in your organization? Yes or No?

🞏 No
🞏 Yes

IF YES, please describe: When was that factor most impactful? Was it during the CSAT grant funding period, after the CSAT funding ended, or throughout?

19D: Would you say helpful factors within your state agency helped efforts to implement and sustain A-CRA in your organization? Yes or No?

🞏 No
🞏 Yes

IF YES, please describe: When was that factor most impactful? Was it during the CSAT grant funding period, after the CSAT funding ended, or throughout?

19E: Would you say helpful factors outside your state agency helped efforts to implement and sustain A-CRA in your organization? Yes or No?

🞏 No
🞏 Yes

IF YES, please describe: When was that factor most impactful? Was it during the CSAT grant funding period, after the CSAT funding ended, or throughout?

19F: Would you say partnerships with other agencies or organizations helped efforts to implement and sustain A-CRA in your organization? Yes or No?

🞏 No
🞏 Yes

IF YES, please describe: When was that factor most impactful? Was it during the CSAT grant funding period, after the CSAT funding ended, or throughout?

19G: Were there other helpful factors for implementing and sustaining A-CRA?

🞏 No
🞏 Yes

IF YES, please describe: When was that factor most impactful? Was it during the CSAT grant funding period, after the CSAT funding ended, or throughout?

1. Now, I’m going ask how the same six categories of factors may have included barriers that hindered efforts to implement and sustain A-CRA in your state. Please let me know if these factors were important in your state, and if yes, how so.

20A. Would you say characteristics of A-CRA hindered efforts to implement and sustain A-CRA in your organization? Yes or No?

🞏 No
🞏 Yes

IF YES, please describe: When was that factor most impactful? Was it during the CSAT grant funding period, after the CSAT funding ended, or throughout?

20B. Would you say challenges with key individuals hindered efforts to implement and sustain A-CRA in your organization? Yes or No?

🞏 No
🞏 Yes

IF YES, please describe: When was that factor most impactful? Was it during the CSAT grant funding period, after the CSAT funding ended, or throughout?

20C. Would you say challenges with treatment organizations hindered efforts to implement and sustain A-CRA in your organization? Yes or No?

🞏 No
🞏 Yes

IF YES, please describe: When was that factor most impactful? Was it during the CSAT grant funding period, after the CSAT funding ended, or throughout?

20D: Would you say challenges within your state agency hindered efforts to implement and sustain A-CRA in your organization? Yes or No?

🞏 No
🞏 Yes

IF YES, please describe: When was that factor most impactful? Was it during the CSAT grant funding period, after the CSAT funding ended, or throughout?

20E: Would you say challenges outside your state agency hindered efforts to implement and sustain A-CRA in your organization? Yes or No?

🞏 No
🞏 Yes

IF YES, please describe: When was that factor most impactful? Was it during the CSAT grant funding period, after the CSAT funding ended, or throughout?

20F: Would you say issues in partnerships with other agencies or organizations hindered efforts to implement and sustain A-CRA in your organization? Yes or No?

🞏 No
🞏 Yes

IF YES, please describe: When was that factor most impactful? Was it during the CSAT grant funding period, after the CSAT funding ended, or throughout?

20G. Were there other barriers to implementing and sustaining A-CRA?

🞏 No
🞏 Yes

IF YES, please describe: When was that factor most impactful? Was it during the CSAT grant funding period, after the CSAT funding ended, or throughout?

**Next, I’m going to ask you a few questions about the COVID-19 pandemic.**

1. How has the COVID-19 pandemic affected substance use treatment services in your state?
2. When the pandemic began in March 2020, were any treatment organizations delivering A-CRA in your state?

🞏 No – ***IF NO, SKIP TO QUESTION 25***
🞏 Yes – ***CONTINUE***

🞏 Don’t know – ***SKIP TO QUESTION 25***

1. What have been the most significant changes affecting A-CRA programs in your state due to COVID-19? [PROBE AS NEEDED]
2. Have there been changes made in response to COVID-19 that have been beneficial, and will those changes continue beyond the pandemic? [PROBE AS NEEDED]

**OK, we are almost done. Next, I want to ask you about your general impressions regarding the SAMHSA CSAT-funded A-CRA project and then have a few questions about you.**

1. If you had a chance to participate in a SAMHSA CSAT project again, would you consider it?

🞏 No
🞏 Yes

1. Why/why not?
2. Is there anything you would change about the SAMHSA CSAT project in order to improve the sustainment of A-CRA in your state?
3. How many years of experience do you have in the administration of substance use treatment services?
4. How many years of experience do you have in your current position and similar positions/levels? This would include experience at other agencies, if relevant.
5. Do you have any experience as a provider of substance use treatment services?

🞏 No – ***IF NO, SKIP TO QUESTION 32***

🞏 Yes – ***CONTINUE***

1. How many years of experience do you have as a provider?
2. Is there anything else you would like to share related to the topic of sustaining A-CRA in your state?
3. Are there written reports or other documentation that you could share with us that might give us insight into your state’s A-CRA initiative? This could include anything that helps us understand the supports provided by your state agency and/or the services provided by treatment organizations that implemented A-CRA. Documents from the grant period are useful, as are documents from the time period since the grant ended. Even redacted or generic/incomplete documents could be very useful to us. I will send you an email after this call with a list, and you can let us know if they’d be available to share and any relevant considerations.

[IF TIME] Here is a list of documents that state agencies might be able to share. Would you be able to share any of the following? If so, can you describe the documents and any next steps for sharing them?

- CSAT grant progress reports and/or applications?
- Data or information related to CSAT grant progress, such as evaluation reports?
- Contracts with A-CRA treatment organizations, from during the CSAT grant?
- Contracts with A-CRA treatment organizations, from after the CSAT grant?
- Planning tools, e.g., “financial maps” of A-CRA funding, from during the CSAT grant?
- Planning tools, e.g., “financial maps” of A-CRA funding, from after the CSAT grant?
- Information disseminated about your state A-CRA initiative for a broader audience? For example:
- COVID-19 response plans relevant for A-CRA?
- Other written reports or documentation that might give us insight into your state’s A-CRA initiative?

[TURN OFF RECORDER]

That's all the interview questions I have. Thank you for providing this information. We will be sending your $25 Amazon e-gift card by e-mail within the next few days. [IF STATE ADMINISTRATOR INTERVIEW OR GROUP INTERVIEW, FIGURE OUT WHO THE GC SHOULD GO TO (ONLY ONE PER STATE). IT IS POSSIBLE THEY CANNOT ACCEPT THE GC.]

[IF PARTICIPANT WILL BE INTERVIEWED AGAIN, complete the tracking module beginning on the final page]

# A-CRA Financing Project: CLINICIAN INTERVIEW COVER SHEET

RESPONDENT ID:

RESPONDENT NAME:

TREATMENT SITE ID:

TREATMENT SITE NAME:

STATE ID:

STATE:

PHONE#:

EMAIL:

DATE OF INTERVIEW: ______ / ______ /_______

TIME OF INTERVIEW (INTERVIEWEE TIME): _______ PT/MT/CT/ET

INTERVIEWER: ______________________

A-CRA Status Expected: Sustainer Non-Sustainer Unknown

Title/Position Expected: Clinician Supervisor Clinician & Supervisor Other Unknown

CHS RECORD OF TRAINING? Yes (month/year) No Unknown

A-CRA GRANTS RECEIVED:

SAT-ED START DATE: ______ / ______ /_______ END DATE: ______ / ______ /_______

SYT START DATE: ______ / ______ /_______ END DATE: ______ / ______ /_______

SYT-I START DATE: ______ / ______ /_______ END DATE: ______ / ______ /_______

STR START DATE: ______ / ______ /_______ END DATE: ______ / ______ /_______

YT-I START DATE: ______ / ______ /_______ END DATE: ______ / ______ /_______

other START DATE: ______ / ______ /_______ END DATE: ______ / ______ /_______

(if other, describe: ___________________ )

PLAN TO INTERVIEW IN WAVE 2? Yes No

OTHER NAMES FOR THE SAMHSA/CSAT-FUNDED A-CRA IMPLEMENTATION PROJECT IN THIS STATE:

RELEVANT CASE NOTES:

***CLINICIAN INTERVIEW VERBAL CONSENT***

(READ PRIOR TO BEGINNING INTERVIEW/RECORDING)

Hi [CLINICIAN NAME],

This is [INTERVIEWER NAME] calling from RAND to speak with you about the youth substance use treatment program where you work and any experiences you have with implementing the Adolescent Community Reinforcement Approach (A-CRA) treatment model. Is this still a good time to complete your interview? To ensure confidentiality, make sure you are in a private, secure location for completing the interview.

>IF NO, RESCHEDULE.

>IF YES, great.

We would like a clinician and/or clinical supervisor for youth substance use treatment to answer these questions. Ideally, it should be someone knowledgeable about your A-CRA program. Are you the right person at your organization to participate in this opportunity?

>IF NO: May I please have the contact information of the best contact(s) for youth substance use treatment? [RECORD CONTACT INFORMATION TO FOLLOW-UP]

>IF YES, thanks for confirming.

Before we begin, let me assure you that your responses to these questions will be held in strict confidence. In collaboration with Chestnut Health Systems, we are requesting interviews and surveys from clinicians and supervisors from nearly 20 states that received A-CRA training from Chestnut Health, as part of SAMHSA Center of Substance Abuse Treatment (CSAT) grants received by state substance use services authorities. We will use information from the interviews and surveys to understand how different CSAT funding models influence the sustainability of A-CRA delivery after funding ends. We will also examine information provided by state organizations that received the CSAT grants and data already collected from prior CSAT grantee organizations that implemented A-CRA. We will not attribute comments to specific individuals or programs in any of our reports or publications. Your responses will not be shared with your organization or with SAMHSA.

Today’s interview will last up to 45 minutes. Afterwards, we will ask you to complete a 30-minute online survey on your own to give us a more complete picture. You will receive a $50 Amazon electronic gift card upon completion of the web survey as a thank you for your participation.

Your participation in this discussion and the survey is entirely voluntary. We would like to have your responses to all of the questions. However, if you’re uncomfortable with any question we can skip it, and you can stop the interview at any time. There are no right or wrong answers – we are interested in your perspectives and experiences. Finally, we would like to audio-record the interview to ensure that we capture everything that is said. We will destroy the recording once we confirm we have captured everything in our de-identified notes and transcripts. However, you can still participate in the interview even if you do not give permission to audio-record.

If you have any questions or want to discuss the project further at any time, you may always contact us at [PHONE] or at [EMAIL]. Furthermore, if you have questions about your rights as a research participant or need to report a research-related injury or concern, you can contact RAND's Human Subjects Protection Committee toll-free at (866) 697-5620 or email [hspcinfo@​rand.org](mailto:hspcinfo@​rand.org)​. If you contact the Committee, please reference Study #2020-N0887.

- **Do you have any questions?**
- **Are you willing to take part in this discussion?**

>IF NO: That is not a problem, thank you for your time.

>IF YES, great.

- **Is it ok with you if we audiotape this discussion?**

>IF NO: That is not a problem. I can take notes while we’re talking so I don’t miss anything important, though that means we might proceed through the interview more slowly than usual. I could also arrange for a colleague to take notes during the interview. [RESCHEDULE IF NEEDED]

>IF YES, perfect, let’s get started.

- [IF UNKNOWN] Based on the background information we’ve received, I am not sure whether your organization currently delivers A-CRA. Can you confirm whether or not A-CRA is delivered by clinicians at your organization?

>IF NO: BEGIN NON-SUSTAINER INTERVIEW

>IF YES: BEGIN SUSTAINER INTERVIEW

**Sustainer Interview – CLINICIAN**

1. Based on the background information we’ve received, it sounds like your agency currently delivers the Adolescent Community Reinforcement Approach or A-CRA. Is this correct?

🞏 No – ***SWITCH TO NON-SUSTAINER INTERVIEW***🞏 Yes – ***CONTINUE***

1. Tell me a little bit about the substance use services at your agency. What services are available? What types of clients do you serve? (e.g., age range, services for specific substances).
2. What youth age range do you use A-CRA with at your agency? Throughout this interview, by “youth” we typically mean ages 12 through 17 (or through age 24 if your A-CRA training included young adults).
3. What is your primary professional role(s) at your clinic/site?

🞏 Clinical Supervisor – ***SWITCH TO SUPERVISOR ONLY VERSION***

🞏 Counselor or Clinician – ***CONTINUE***
🞏 Clinical Supervisor and Counselor/Clinician ***– SWITCH TO SUPERVISOR + CLINICIAN VERSION***

🞏 Other, please describe: ____________________ (e.g., administrator, grant manager**) *– SWITCH TO SUPERVISOR VERSION***

1. Did you participate in any SAMHSA/CSAT-funded A-CRA training initiatives in your state? Yes or No? *[IF NEEDED, PROMPT AS RELEVANT: Our records do/do not indicate that you received training in A-CRA as part of a SAMHSA CSAT grant under the [initiative] in [grants years]. (IF KNOWN) In your state, I believe this initiative was called [NAME]. So would it be accurate to say you did/did not participate in your state’s A-CRA training initiative?]*

🞏 No **– *SKIP TO QUESTION 6***🞏 Yes **– *CONTINUE***

5A. Between what dates did you work on the project? Your best guess is fine.

1. What role(s) have you served in delivering A-CRA? *[MARK ALL THAT APPLY]*

🞏 A-CRA Therapist
🞏 A-CRA Clinical Supervisor
🞏 Other (Please describe: ____________________________________________________)
🞏 No Role in delivering ACRA

1. Have you received any A-CRA certifications? This means that you completed all training requirements (such as coaching and submission of recorded sessions) and received written documentation of the certification. *[MARK ALL THAT APPLY] [IF NEEDED, PROMPT: A-CRA supervisor certification is different from A-CRA certification.  It refers to being certified to provide A-CRA clinical supervision].*

🞏 First-level clinician certification (passed 9 A-CRA procedures)
🞏 Full clinician certification (passed all 19 A-CRA procedures)
🞏 Supervisor certification
🞏 A-CRA-TAY (Transitional Age Youth) certification

🞏 No, never certified in A-CRA

1. What do you think of A-CRA as a treatment for youth? Please note that throughout this interview, by “youth” we mean the population served during your SAMHSA CSAT grant periods.
2. Does your program currently offer any different types of treatment for youth with substance use disorders besides A-CRA?

🞏 No – ***SKIP TO QUESTION 11***🞏 Yes – ***CONTINUE***🞏 Don’t Know – ***SKIP TO QUESTION 11***

1. What are those treatments called? *[IF NEEDED, PROMPT: Examples of other treatments include Motivational Enhancement Therapy (MET), Cognitive Behavioral Therapy (CBT), Multidimensional Family Therapy (MDFT), 12-step facilitation, and supportive counseling as well as medication treatments like Suboxone or Naltrexone. OR you can just describe the treatment. Is it outpatient? How long does it last? # of sessions/days/months?]*
2. Tell me about any ways in which your organization collaborates with other organizations to sustain A-CRA. *[IF NEEDED, PROMPT: For example, you may have engaged in training or support activities with your state substance use services authority and/or Chestnut Health Systems or have partnered with universities or research institutions on research related to A-CRA].*
3. Tell me about any planning that has been done to ensure resources are available to continue A-CRA. By resources, we mean things needed to support A-CRA. For example, this could include things like money, staff, supervision, training, and A-CRA manuals. [*IF NEEDED, PROMPT: By strategic planning, we mean the process by which an organization defines its strategy or direction and makes decisions about how to allocate resources to pursue the strategy.]*
4. Can you please describe any policies that support A-CRA delivery? These could include external policies, such as funding source and billing rules that support A-CRA, as well as internal policies at your organization. *[IF NEEDED, PROMPT: By policies, we mean either organizational, state, national, or local policies. Here are some examples:*

*-State/county/health insurance co requires that we use an evidence-based treatment (EBT) and A-CRA is an EBT*

*-Our agency’s mission emphasizes involvement of the family, A-CRA does that]*

1. What about policies (external or internal) that interfere with A-CRA delivery? *[IF NEEDED, PROMPT: By policies, we mean either organizational, state, national, or local policies. Here are some examples:*

*-if a state decided to require that substance use clinics deliver a different treatment (other than A-CRA), this policy might interfere with the delivery of A-CRA.*

*- if certain insurance plans stopped reimbursing for A-CRA or the full number of sessions, this policy would interfere with A-CRA delivery.]*

1. Does A-CRA meet the needs of the populations you serve? How so or why not?
2. Can you tell me about any pressure your organization experienced to continue delivering A-CRA or to discontinue its use? *[IF NEEDED, PROMPT: Pressure to deliver A-CRA could come from a variety of sources. For ex., organizations might experience pressure if there is some financial incentive to deliver A-CRA. If a state department of health decides its mission is to deliver evidence-based treatment for substance use, this could also be seen as pressure.]*
3. Would you say staff are supportive or reluctant to use A-CRA? Can you give me an example of what people have said or done to indicate their supportiveness or reluctance toward A-CRA?
4. Next, I would like to know what factors helped efforts to implement and sustain A-CRA in your organization. Please let me know if these factors were important in your organization, and if yes, how so. *[PROBE AS NEEDED: What factors were most helpful in that domain? Can you give me some examples? Were there important changes in which factors were most helpful once funding ended?]*

18A. Would you say characteristics of A-CRA helped efforts to implement and sustain A-CRA in your organization? Yes or No?

*[IF NEEDED: for example, treatment content, structure of sessions, etc.]*

🞏 No
🞏 Yes

IF YES, please describe: When was that factor most impactful? Was it during the CSAT grant funding period, after the CSAT funding ended, or throughout?

18B. Would you say characteristics of key individuals helped efforts to implement and sustain A-CRA in your organization? Yes or No?

*[IF NEEDED: “key individuals” are people who had a major positive impact on A-CRA implementation; could be within or outside of your treatment organization]*

🞏 No
🞏 Yes

IF YES, please describe: When was that factor most impactful? Was it during the CSAT grant funding period, after the CSAT funding ended, or throughout

18C. Would you say client perspectives on A-CRA helped efforts to implement and sustain A-CRA in your organization?  Yes or No?

*[IF NEEDED: for example, if clients found A-CRA acceptable, appropriate, feasible, etc.]*

🞏 No
🞏 Yes

IF YES, please describe: When was that factor most impactful? Was it during the CSAT grant funding period, after the CSAT funding ended, or throughout?

18D. Would you say helpful factors within your organization helped efforts to implement and sustain A-CRA in your organization? Yes or No?

*[IF NEEDED: for example, organizational leadership, staffing patterns, scheduling appointments, etc.]*

🞏 No
🞏 Yes

IF YES, please describe: When was that factor most impactful? Was it during the CSAT grant funding period, after the CSAT funding ended, or throughout?

18E. Would you say helpful factors outside your organization helped efforts to implement and sustain A-CRA in your organization? Yes or No?

*[IF NEEDED: for example, state leadership, federal support, community factors]*

🞏 No
🞏 Yes

IF YES, please describe: When was that factor most impactful? Was it during the CSAT grant funding period, after the CSAT funding ended, or throughout?

18F. Would you say partnerships with other organizations helped efforts to implement and sustain A-CRA in your organization? Yes or No?

🞏 No
🞏 Yes

IF YES, please describe: When was that factor most impactful? Was it during the CSAT grant funding period, after the CSAT funding ended, or throughout?

18G. Would you say funding helped efforts to implement and sustain A-CRA in your organization? Yes or No?

🞏 No
🞏 Yes

IF YES, please describe: When was that factor most impactful? Was it during the CSAT grant funding period, after the CSAT funding ended, or throughout?

18H. Were there other helpful factors during or after the grant period?

🞏 No
🞏 Yes

IF YES, please describe: When was that factor most impactful? Was it during the CSAT grant funding period, after the CSAT funding ended, or throughout?

1. Now, I’m going ask how the same six categories of factors may have included barriers that hindered efforts to implement and sustain A-CRA in your organization. Please let me know if these factors were important in your organization, and if yes, how so. *[PROBE AS NEEDED: What factors were the biggest barriers in that domain? Can you give me some examples? Were there important changes in which factors were the biggest barriers once funding ended?]*

19A. Would you say characteristics of A-CRA hindered efforts to implement and sustain A-CRA in your organization? Yes or No?

*[IF NEEDED: for example, treatment content, structure of sessions, etc.]*

🞏 No
🞏 Yes

IF YES, please describe: When was that factor most impactful? Was it during the CSAT grant funding period, after the CSAT funding ended, or throughout?

19B. Would you say challenges with key individuals hindered efforts to implement and sustain A-CRA in your organization? Yes or No?

*[IF NEEDED: “key individuals” are people who presented major challenges for A-CRA implementation; could be within or outside of your treatment organization]*

🞏 No
🞏 Yes

IF YES, please describe: When was that factor most impactful? Was it during the CSAT grant funding period, after the CSAT funding ended, or throughout?

19C. Would you say client perspectives on A-CRA hindered efforts to implement and sustain A-CRA in your organization? Yes or No?

*[IF NEEDED: for example, if clients found A-CRA acceptable, appropriate, feasible, etc.]*

🞏 No
🞏 Yes

IF YES, please describe: When was that factor most impactful? Was it during the CSAT grant funding period, after the CSAT funding ended, or throughout?

19D. Would you say challenges within your organization hindered efforts to implement and sustain A-CRA in your organization? Yes or No?

*[IF NEEDED: for example, organizational leadership, staffing patterns, scheduling appointments, etc.]*

🞏 No
🞏 Yes

IF YES, please describe: When was that factor most impactful? Was it during the CSAT grant funding period, after the CSAT funding ended, or throughout?

19E. Would you say challenges outside your organization hindered efforts to implement and sustain A-CRA in your organization? Yes or No?

*[IF NEEDED: for example, state leadership, federal support, community factors]*

🞏 No
🞏 Yes

IF YES, please describe: When was that factor most impactful? Was it during the CSAT grant funding period, after the CSAT funding ended, or throughout?

19F. Would you say issues in partnerships with other organizations hindered efforts to implement and sustain A-CRA in your organization? Yes or No?

🞏 No
🞏 Yes

IF YES, please describe: When was that factor most impactful? Was it during the CSAT grant funding period, after the CSAT funding ended, or throughout?

19G. Would you say funding hindered efforts to implement and sustain A-CRA in your organization? Yes or No?

🞏 No
🞏 Yes

IF YES, please describe: When was that factor most impactful? Was it during the CSAT grant funding period, after the CSAT funding ended, or throughout?

19H. Were there other barriers during or after the grant period?

🞏 No
🞏 Yes

IF YES, please describe: When was that factor most impactful? Was it during the CSAT grant funding period, after the CSAT funding ended, or throughout?

1. Do you have a copy of the A-CRA manual? *[IF NEEDED, PROMPT: If you have access to a shared copy of the manual through your organization, electronically, etc. that counts as having a copy.]*

🞏 No – ***SKIP TO QUESTION 22***🞏 Yes – ***CONTINUE***

1. In the past six-months, how often would you say you used the manual? *[IF NEEDED, PROMPT: By use, we mean a reference to the manual. It could mean a quick review of key session content in preparation for a session, or a thorough read-through in order to master the material.]*

🞏 Never
 🞏 A few times per year, or less
 🞏 About once a month
 🞏 A few times per month
 🞏 Weekly
 🞏 Daily

1. In the past six-months, how often do you receive group clinical supervision? *[IF NEEDED, PROMPT:  This includes any supervision you received; not supervision you provided. Our focus is on A-CRA supervision but you can include any supervision where A-CRA would be discussed – it does not need to be exclusively supervision for A-CRA cases.]*

🞏 Never
 🞏 Once a week
 🞏 Every two weeks *[INTWR: THIS INCLUDES “TWICE A MONTH”]*
 🞏 Monthly
 🞏 Other, please describe how often: ___________________________________

1. In the past six-months, how often do you receive individual clinical supervision? *[IF NEEDED, PROMPT:  This includes any supervision you received; not supervision you provided. Our focus is on A-CRA supervision but you can include any supervision where A-CRA would be discussed – it does not need to be exclusively supervision for A-CRA cases.]*

🞏 Never
 🞏 Once a week
 🞏 Every two weeks *[INTWR: THIS INCLUDES “TWICE A MONTH”]*
 🞏 Monthly
 🞏 Other, please describe how often: ___________________________________

**IF QUESTION 22 = Never AND QUESTION 23 = Never, SKIP TO QUESTION 34**

**Which of the following happens during supervision sessions?**

1. My supervisor asks how my week has gone.

🞏 No
🞏 Yes

1. My supervisor discusses agency paperwork requirements.

🞏 No
🞏 Yes

1. My supervisor reviews my A-CRA case review report.

🞏 No
🞏 Yes

1. My supervisor asks me if I have any problem cases.

🞏 No
🞏 Yes

1. My supervisor reviews a recorded session with me and tells me what I have done well.

🞏 No
🞏 Yes

1. My supervisor reviews a recorded session with me and gives me suggestions about how I can improve my treatment delivery

🞏 No
🞏 Yes

1. My supervisor observes a live session and tells me what I have done well.

🞏 No
🞏 Yes

1. My supervisor observes a live session and gives me suggestions about how I can improve my treatment delivery

🞏 No
🞏 Yes

1. My supervisor asks me about my personal problems.

🞏 No
🞏 Yes

1. My supervisor role plays with me the correct way to do a procedure.

🞏 No
🞏 Yes

**Currently, when you are introducing A-CRA to a new client…**

1. How many sessions do you tell the client receiving A-CRA they will have?
2. How many weeks do you tell the client receiving A-CRA the treatment will take?
3. How has the COVID-19 pandemic affected substance use treatment services at your organization?
4. How has it affected the ability of your organization to sustain A-CRA services?
5. What have been the most significant changes affecting your A-CRA program in response to COVID-19? *[PROBE AS NEEDED: Changes could include things like…*

*…Changes in referrals to A-CRA*

*…Changes in how A-CRA assessment, treatment, or case management services are delivered*

*…Changes in staffing*

*…Remote work*

*…Telehealth service delivery*

*Were there other changes? If so, please describe:]*

1. Have there been changes made in response to COVID-19 that have been beneficial, and will those changes continue beyond the pandemic? *[PROBE AS NEEDED: These may be some of the changes you already described, or may be different. Changes could include things like…*

*…Changes in referrals to A-CRA*

*…Changes in how A-CRA assessment, treatment, or case management services are delivered*

*…Changes in staffing*

*…Remote work*

*…Telehealth service delivery*

*Were there other beneficial changes? If so, please describe:]*

**OK, we are almost done. Next, I want to ask you about your general impressions regarding the SAMHSA CSAT-funded A-CRA project and then have a few questions about you.**

1. If you had a chance to participate in a SAMHSA CSAT project again, would you consider it?

🞏 No
🞏 Yes

1. Why/why not?
2. Is there anything you would change about the SAMHSA CSAT project in order to improve the sustainment of A-CRA at your agency?

1. Is there anything else you would like to share related to the topic of sustaining A-CRA at your agency?

[TURN OFF RECORDER]

That's all the interview questions I have. Thank you for providing this information.

[IF SENDING WEB SURVEY (FULL OR ABBREVIATED) AS USUAL]

We will soon be sending you the link to the web survey in order to provide a more complete picture of the treatment offered to youth at your organization and about organizational and clinical support. We will send it within a few days, and will send your $50 Amazon e-gift card as soon as the survey is complete.

[IF PARTICIPANT WILL BE INTERVIEWED AGAIN, complete the tracking module]

**Non-Sustainer Interview – CLINICIAN**

1. Based on the background information we’ve received, it sounds like your agency currently does not deliver the Adolescent Community Reinforcement Approach or A-CRA anymore. Is this correct?

🞏 No – ***SWITCH TO SUSTAINER INTERVIEW***🞏 Yes – ***CONTINUE***

1. Tell me a little bit about the substance use services at your agency. What services are available? What types of clients do you serve? (e.g., age range, services for specific substances).
2. What youth age range do you use A-CRA with at your agency? Throughout this interview, by “youth” we typically mean ages 12 through 17 (or through age 24 if your A-CRA training included young adults).
3. What is your primary professional role(s) at your clinic/site?

🞏 Clinical Supervisor – ***SWITCH TO SUPERVISOR ONLY VERSION***

🞏 Counselor or Clinician – ***CONTINUE***
🞏 Clinical Supervisor and Counselor/Clinician *–* ***SWITCH TO SUPERVISOR + CLINICIAN VERSION***

🞏 Other, please describe: ____________________ (e.g., administrator, grant manager) *–* ***SWITCH TO SUPERVISOR VERSION***

1. Did you participate in any SAMHSA/CSAT-funded A-CRA training initiatives in your state? Yes or No? *[IF NEEDED, PROMPT AS RELEVANT: Our records do/do not indicate that you received training in A-CRA as part of a SAMHSA CSAT grant under the [INITIATIVE] in [GRANT YEARS]. (IF KNOWN) In your state, I believe this initiative was called [NAME]. So would it be accurate to say you did/did not participate in your state’s A-CRA training initiative?]*

🞏 No – ***SKIP TO QUESTION 6***
🞏 Yes – ***CONTINUE***

5A. Between what dates did you work on the project? Your best guess is fine.

1. What role(s) did you serve while A-CRA was being delivered at your agency? *[MARK ALL THAT APPLY]*

🞏 A-CRA Therapist
 🞏 A-CRA Supervisor
 🞏 Other (Please describe: ____________________________________________________)
 🞏 No Role in delivering ACRA

1. Have you received any A-CRA certifications? This means that you completed all training requirements (such as coaching and submission of recorded sessions) and received written documentation of the certification. [MARK ALL THAT APPLY]

🞏 First-level clinician certification (passed 9 A-CRA procedures)

🞏 Full clinician certification (passed all 19 A-CRA procedures)
 🞏 Supervisor certification

🞏 A-CRA-TAY (Transitional Age Youth) certification

🞏 No, never certified in A-CRA

*[IF NEEDED, PROMPT: A-CRA supervisor certification is different from A-CRA certification. It refers to being certified to provide A-CRA clinical supervision].*

1. What do you think of A-CRA as a treatment for youth?
2. When did you/your agency stop delivering A-CRA? If you do not know the exact date, please give your best estimate.
3. What were the main reasons you/your agency stopped delivering A-CRA?
4. What would have increased your desire to continue delivering A-CRA?
5. What would have increased your ability to continue delivering A-CRA?
6. Does your program currently offer any different types of treatment for youth with substance use disorders besides A-CRA?

🞏 No – ***SKIP TO QUESTION 20***🞏 Yes – ***CONTINUE***
🞏 Don’t Know – ***SKIP TO QUESTION 20***

1. What are those treatments called? *[IF NEEDED, PROMPT: Examples of other treatments include Motivational Enhancement Therapy (MET), Cognitive Behavioral Therapy (CBT), Multidimensional Family Therapy (MDFT), 12-step facilitation, and supportive counseling as well as medication treatments like Suboxone or Naltrexone. OR you can just describe the treatment. Is it outpatient? How long does it last? # of sessions/days/months?]*
2. Tell me about any ways in which your organization collaborates with other organizations – especially anything related to A-CRA. *[IF NEEDED, PROMPT: For example, you may have engaged in training or support activities with your state substance use services authority and/or Chestnut Health Systems, or have partnered with universities or research institutions on research related to A-CRA].*
3. Was there any planning done to ensure resources were available to continue A-CRA beyond the initial funding period? By resources, we mean things needed to support A-CRA. For example, this could include things like money, staff, supervision, training, and A-CRA manuals. [*IF NEEDED, PROMPT: By strategic planning, we mean the process by which an organization defines its strategy or direction and makes decisions about how to allocate resources to pursue the strategy.]*
4. Can you please describe any policies that supported A-CRA delivery? These could include external policies, such as funding source and billing rules that support A-CRA, as well as internal policies at your organization. *[IF NEEDED, PROMPT: By policies, we mean either organizational, state, national, or local policies. Here are some examples:*

*-State/county/health insurance co requires that we use an evidence-based treatment (EBT) and A-CRA is an EBT*

*-Our agency’s mission emphasizes the involvement of the family, A-CRA does that]*

1. What about policies (external or internal) that interfered with A-CRA delivery? *[IF NEEDED, PROMPT:  By policies, we mean either organizational, state, national, or local policies. Here are some examples:*

*-if a state decided to require that substance use clinics deliver a different treatment (other than A-CRA), this policy might interfere with the delivery of A-CRA.*

*- if certain insurance plans stopped reimbursing for A-CRA or the full number of sessions, this policy would interfere with A-CRA delivery.]*

1. Did A-CRA meet the needs of the populations you serve? How so or why not?
2. Can you tell me about any pressure your organization experienced to continue delivering A-CRA or to discontinue its use? *[IF NEEDED, PROMPT:  Pressure to deliver A-CRA could come from a variety of sources. For example, organizations might experience pressure if there is some financial incentive to deliver A-CRA. If a state department of health decides its mission is to deliver evidence-based treatment for substance use, this could also be seen as pressure.]*
3. Would you say staff were supportive or reluctant to use A-CRA? Can you give me an example of what people have said or done to indicate their supportiveness or reluctance toward A-CRA?
4. Next, I would like to know what factors helped efforts to implement and sustain A-CRA in your organization. Please let me know if these factors were important at your organization, and if yes, how so. *[PROBE AS NEEDED: What factors were most helpful in that domain? Can you give me some examples? Were there important changes in which factors were most helpful once funding ended?]*

27A. Would you say characteristics of A-CRA helped efforts to implement and sustain A-CRA in your organization? Yes or No?

*[IF NEEDED: for example, treatment content, structure of sessions, etc.]*

🞏 No
🞏 Yes

IF YES, please describe: When was that factor most impactful? Was it during the CSAT grant funding period, after the CSAT funding ended, or throughout?

27B. Would you say characteristics of key individuals helped efforts to implement and sustain A-CRA in your organization? Yes or No?

*[IF NEEDED: “key individuals” are people who had a major positive impact on A-CRA implementation; could be within or outside of your treatment organization]*

🞏 No
🞏 Yes

IF YES, please describe: When was that factor most impactful? Was it during the CSAT grant funding period, after the CSAT funding ended, or throughout?

27C. Would you say client perspectives on A-CRA helped efforts to implement and sustain A-CRA in your organization? Yes or No?

*[IF NEEDED: for example, if clients found A-CRA acceptable, appropriate, feasible, etc.]*

🞏 No
🞏 Yes

IF YES, please describe: When was that factor most impactful? Was it during the CSAT grant funding period, after the CSAT funding ended, or throughout?

27D. Would you say helpful factors within your organization helped efforts to implement and sustain A-CRA in your organization? Yes or No?

*[IF NEEDED: for example, organizational leadership, staffing patterns, scheduling appointments, etc.]*

🞏 No
🞏 Yes

IF YES, please describe: When was that factor most impactful? Was it during the CSAT grant funding period, after the CSAT funding ended, or throughout?

27E. Would you say helpful factors outside your organization helped efforts to implement and sustain A-CRA in your organization? Yes or No?

*[IF NEEDED: for example, state leadership, federal support, community factors]*

🞏 No
🞏 Yes

IF YES, please describe: When was that factor most impactful? Was it during the CSAT grant funding period, after the CSAT funding ended, or throughout?

27F. Would you say partnerships with other organizations helped efforts to implement and sustain A-CRA in your organization? Yes or No?

🞏 No
🞏 Yes

IF YES, please describe: When was that factor most impactful? Was it during the CSAT grant funding period, after the CSAT funding ended, or throughout?

27G. Would you say funding helped efforts to implement and sustain A-CRA in your organization? Yes or No?

🞏 No
🞏 Yes

IF YES, please describe: When was that factor most impactful? Was it during the CSAT grant funding period, after the CSAT funding ended, or throughout?

27H. Were there other helpful factors during or after the grant period?

🞏 No
🞏 Yes

IF YES, please describe: When was that factor most impactful? Was it during the CSAT grant funding period, after the CSAT funding ended, or throughout?

1. Now, I’m going ask how the same six categories of factors may have included barriers that hindered efforts to implement and sustain A-CRA in your organization. Please let me know if these factors were important at your organization, and if yes, how so. *[PROBE AS NEEDED: What factors were the biggest barriers in that domain? Can you give me some examples? Were there important changes in which factors were the biggest barriers once funding ended?]*

28A. Would you say characteristics of A-CRA hindered efforts to implement and sustain A-CRA in your organization? Yes or No?

*[IF NEEDED: for example, treatment content, structure of sessions, etc.]*

🞏 No
🞏 Yes

IF YES, please describe: When was that factor most impactful? Was it during the CSAT grant funding period, after the CSAT funding ended, or throughout?

28B. Would you say challenges with key individuals hindered efforts to implement and sustain A-CRA in your organization? Yes or No?

*[IF NEEDED: “key individuals” are people who presented major challenges for A-CRA implementation; could be within or outside of your treatment organization]*

🞏 No
🞏 Yes

IF YES, please describe: When was that factor most impactful? Was it during the CSAT grant funding period, after the CSAT funding ended, or throughout?

28C. Would you say client perspectives on A-CRA hindered efforts to implement and sustain A-CRA in your organization? Yes or No?

*[IF NEEDED: for example, if clients found A-CRA acceptable, appropriate, feasible, etc.]*

🞏 No
🞏 Yes

IF YES, please describe: When was that factor most impactful? Was it during the CSAT grant funding period, after the CSAT funding ended, or throughout?

28D. Would you say challenges within your organization hindered efforts to implement and sustain A-CRA in your organization? Yes or No?

*[IF NEEDED: for example, organizational leadership, staffing patterns, scheduling appointments, etc.]*

🞏 No
🞏 Yes

IF YES, please describe: When was that factor most impactful? Was it during the CSAT grant funding period, after the CSAT funding ended, or throughout?

28E. Would you say challenges outside your organization hindered efforts to implement and sustain A-CRA in your organization? Yes or No?

*[IF NEEDED: for example, state leadership, federal support, community factors]*

🞏 No
🞏 Yes

IF YES, please describe: When was that factor most impactful? Was it during the CSAT grant funding period, after the CSAT funding ended, or throughout?

28F. Would you say issues in partnerships with other organizations hindered efforts to implement and sustain A-CRA in your organization? Yes or No?

🞏 No
🞏 Yes

IF YES, please describe: When was that factor most impactful? Was it during the CSAT grant funding period, after the CSAT funding ended, or throughout?

28G. Would you say funding hindered efforts to implement and sustain A-CRA in your organization? Yes or No?

🞏 No
🞏 Yes

IF YES, please describe: When was that factor most impactful? Was it during the CSAT grant funding period, after the CSAT funding ended, or throughout?

28H. Were there other barriers during or after the grant period?

🞏 No
🞏 Yes

IF YES, please describe: When was that factor most impactful? Was it during the CSAT grant funding period, after the CSAT funding ended, or throughout?

1. Have you ever had a copy of the A-CRA manual? *[IF NEEDED, PROMPT: If you have access to a shared copy of the manual through your organization, electronically, etc. that counts as having a copy.]*

🞏 No – ***SKIP TO QUESTION 33***
🞏 Yes – ***CONTINUE***

1. Please think about the six-month period, right before A-CRA treatment delivery ended. How often did you use the manual? *[IF NEEDED, PROMPT: By use, we mean a reference to the manual. It could mean a quick review of key session content in preparation for a session, or a thorough read-through in order to master the material.]*

🞏 Never
🞏 A few times per year, or less
🞏 About once a month
🞏 A few times per month
🞏 Weekly
🞏 Daily

1. Do you still use your manual? *[IF NEEDED, PROMPT: By use, we mean a reference to the manual. It could mean a quick review of key session content in preparation for a session, or a thorough read-through in order to master the material.]*

🞏 No – ***SKIP TO QUESTION 33***
🞏 Yes – ***CONTINUE***

1. How often do you use your manual now? *[IF NEEDED, PROMPT: By use, we mean a reference to the manual. It could mean a quick review of key session content in preparation for a session, or a thorough read-through in order to master the material.]*

🞏 Never
🞏 A few times per year, or less
🞏 About once a month
🞏 A few times per month
🞏 Weekly
🞏 Daily

**For the next few questions, please think about the six-month period, right before A-CRA treatment delivery ended.**

1. How often did you receive group clinical supervision? *[IF NEEDED, PROMPT:  This includes any supervision you received; not supervision you provided. Our focus is on A-CRA supervision but you can include any supervision where A-CRA would be discussed – it does not need to be exclusively supervision for A-CRA cases.]*

🞏 Never
🞏 Once a week
🞏 Every two weeks
🞏 Monthly
🞏 Other, please describe how often: ___________________________________

1. Again, please think about the six-month period, right before A-CRA treatment delivery ended. How often did you receive individual clinical supervision? *[IF NEEDED, PROMPT:  This includes any supervision you received; not supervision you provided. Our focus is on A-CRA supervision but you can include any supervision where A-CRA would be discussed – it does not need to be exclusively supervision for A-CRA cases.]*

🞏 Never
🞏 Once a week
🞏 Every two weeks
🞏 Monthly
🞏 Other, please describe how often: ___________________________________

**IF QUESTION 33 = Never AND QUESTION 34 = Never, SKIP TO QUESTION 45**

**Thinking about the six-month period, right before A-CRA treatment delivery ended, which of the following happened during supervision sessions?**

1. My supervisor asked how my week had gone.

🞏 No
🞏 Yes

1. (During the six-month period, right before A-CRA treatment delivery ended), my supervisor discussed agency paperwork requirements.

🞏 No
🞏 Yes

1. (During the six-month period, right before A-CRA treatment delivery ended), my supervisor reviewed my A-CRA case review report.

🞏 No
🞏 Yes

1. (During the six-month period, right before A-CRA treatment delivery ended), my supervisor asked me if I had any problem cases.

🞏 No
🞏 Yes

1. (During the six-month period, right before A-CRA treatment delivery ended), my supervisor reviewed a recorded session with me and told me what I did well.

🞏 No
🞏 Yes

1. (During the six-month period, right before A-CRA treatment delivery ended), my supervisor reviewed a recorded session with me and gave me suggestions about how I could improve my treatment delivery

🞏 No
🞏 Yes

1. During the six-month period, right before A-CRA treatment delivery ended, my supervisor observed a live session and told me what I did well.

🞏 No
🞏 Yes

1. (During the six-month period, right before A-CRA treatment delivery ended), my supervisor observed a live session and gave me suggestions about how I could improve my treatment delivery

🞏 No
🞏 Yes

1. (During the six-month period, right before A-CRA treatment delivery ended), my supervisor asked me about my personal problems.

🞏 No
🞏 Yes

1. (During the six-month period, right before A-CRA treatment delivery ended), my supervisor role played with me the correct way to do a procedure.

🞏 No
🞏 Yes

**Now I’d like to ask you the same questions, but now please think about the current practices in the adolescent treatment program.**

1. How often do you receive group clinical supervision? *[IF NEEDED, PROMPT:  This includes any supervision you received; not supervision you provided. Our focus is on A-CRA supervision but you can include any supervision where A-CRA would be discussed – it does not need to be exclusively supervision for A-CRA cases.]*

🞏 Never
🞏 Once a week
🞏 Every two weeks
🞏 Monthly
🞏 Other, please describe how often:___________________________________

1. How often do you receive individual clinical supervision? *[IF NEEDED, PROMPT:  This includes any supervision you received; not supervision you provided. Our focus is on A-CRA supervision but you can include any supervision where A-CRA would be discussed – it does not need to be exclusively supervision for A-CRA cases.]*

🞏 Never
🞏 Once a week
🞏 Every two weeks
🞏 Monthly
🞏 Other, please describe how often:___________________________________

**IF QUESTION 45= Never AND QUESTION 46 = Never, SKIP TO QUESTION 56**

**Thinking about the current practices in your adolescent treatment program, which of the following happens during supervision sessions?**

1. My supervisor asks me how my week has gone.

🞏 No
🞏 Yes

1. My supervisor discusses agency paperwork requirements.

🞏 No
🞏 Yes

1. My supervisor asks me if I have any problem cases.

🞏 No
🞏 Yes

1. My supervisor reviews a recorded session with me and tells me what I have done well.

🞏 No
🞏 Yes

1. My supervisor reviews a recorded session with me and gives me suggestions about how I could improve my treatment delivery.

🞏 No
🞏 Yes

1. My supervisor observes a live session and tells me what I have done well.

🞏 No
🞏 Yes

1. My supervisor observes a live session and gives me suggestions about how I could improve my treatment delivery.

🞏 No
🞏 Yes

1. My supervisor asks me about my personal problems.

🞏 No
🞏 Yes

1. My supervisor role plays with me the correct way to deliver therapy.

🞏 No
🞏 Yes

1. During the six-month period, right before A-CRA treatment delivery ended, how many sessions did you tell the client receiving A-CRA they would have?
2. During the six-month period, right before A-CRA treatment delivery ended, how many weeks did you tell the client receiving A-CRA the treatment would take?
3. How has the COVID-19 pandemic affected substance use treatment services at your organization?
4. When the pandemic began in March 2020, was your organization still delivering A-CRA?

🞏 No – ***SKIP TO QUESTION 62***

🞏 Yes – ***CONTINUE***

🞏 Don’t know – ***SKIP TO QUESTION 62***

1. What have been the most significant changes affecting your A-CRA program in response to COVID-19? *[PROBE AS NEEDED: Changes could include things like…*

*…A-CRA was discontinued due to COVID-related changes*

*…Changes in referrals to A-CRA*

*…Changes in how A-CRA assessment, treatment, or case management services are delivered*

*…Changes in staffing*

*…Remote work*

*…Telehealth service delivery*

*Were there other changes? If so, please describe: ]*

1. Have there been changes made in response to COVID-19 that have been beneficial, and will those changes continue beyond the pandemic? *[PROBE AS NEEDED: These may be some of the changes you already described, or may be different. Changes could include things like…*

*…Changes in referrals to A-CRA*

*…Changes in how A-CRA assessment, treatment, or case management services are delivered*

*…Changes in staffing*

*…Remote work*

*…Telehealth service delivery*

*Were there other beneficial changes? If so, please describe: ]*

**OK, we are almost done. Next, I want to ask you about your general impressions regarding the SAMHSA CSAT-funded A-CRA project and then have a few questions about you.**

1. If you had a chance to participate in a SAMHSA CSAT project again, would you consider it?

🞏 No
🞏 Yes

1. Why/why not?
2. Is there anything you would change about the SAMHSA CSAT project in order to improve the sustainment of A-CRA at your agency?
3. Is there anything else you would like to share related to the topic of sustaining A-CRA at your agency?

[TURN OFF RECORDER]

That's all the interview questions I have. Thank you for providing this information.

[IF SENDING WEB SURVEY (FULL OR ABBREVIATED) AS USUAL]

We will soon be sending you the link to the web survey in order to provide a more complete picture of the treatment offered to youth at your organization and about organizational and clinical support. We will send it within a few days, and will send your $50 Amazon e-gift card as soon as the survey is complete.

[IF PARTICIPANT WILL BE INTERVIEWED AGAIN, complete the tracking module]

***CLINICIAN/SUPERVISOR INTERVIEW VERBAL CONSENT***

(READ PRIOR TO BEGINNING INTERVIEW/RECORDING)

Hi [CLINICIAN/SUPERVISOR NAME],

This is [INTERVIEWER NAME] calling from RAND to speak with you about the youth substance use treatment program where you work and any experiences you have with implementing the Adolescent Community Reinforcement Approach (A-CRA) treatment model. Is this still a good time to complete your interview? To ensure confidentiality, make sure you are in a private, secure location for completing the interview.

>IF NO, RESCHEDULE.

>IF YES, great.

We would like a clinician and/or clinical supervisor for youth substance use treatment to answer these questions. Ideally, it should be someone knowledgeable about your A-CRA program. Are you the right person at your organization to participate in this opportunity?

>IF NO: May I please have the contact information of the best contact(s) for youth substance use treatment? [RECORD CONTACT INFORMATION TO FOLLOW-UP]

>IF YES, thanks for confirming.

Before we begin, let me assure you that your responses to these questions will be held in strict confidence. In collaboration with Chestnut Health Systems, we are requesting interviews and surveys from clinicians and supervisors from nearly 20 states that received A-CRA training from Chestnut Health, as part of SAMHSA Center of Substance Abuse Treatment (CSAT) grants received by state substance use services authorities. We will use information from the interviews and surveys to understand how different CSAT funding models influence the sustainability of A-CRA delivery after funding ends. We will also examine information provided by state organizations that received the CSAT grants and data already collected from prior CSAT grantee organizations that implemented A-CRA. We will not attribute comments to specific individuals or programs in any of our reports or publications. Your responses will not be shared with your organization or with SAMHSA.

Today’s interview will last up to 45 minutes. Afterwards, we will ask you to complete a 30-minute online survey on your own to give us a more complete picture. You will receive a $50 Amazon electronic gift card upon completion of the web survey as a thank you for your participation.

Your participation in this discussion and the survey is entirely voluntary. We would like to have your responses to all of the questions. However, if you’re uncomfortable with any question we can skip it, and you can stop the interview at any time. There are no right or wrong answers – we are interested in your perspectives and experiences. Finally, we would like to audio-record the interview to ensure that we capture everything that is said. We will destroy the recording once we confirm we have captured everything in our de-identified notes and transcripts. However, you can still participate in the interview even if you do not give permission to audio-record.

If you have any questions or want to discuss the project further at any time, you may always contact us at [PHONE] or at [EMAIL]. Furthermore, if you have questions about your rights as a research participant or need to report a research-related injury or concern, you can contact RAND's Human Subjects Protection Committee toll-free at (866) 697-5620 or email [hspcinfo@​rand.org](mailto:hspcinfo@​rand.org)​. If you contact the Committee, please reference Study #2020-N0887.

- **Do you have any questions?**
- **Are you willing to take part in this discussion?**

>IF NO: That is not a problem, thank you for your time.

>IF YES, great.

- **Is it ok with you if we audiotape this discussion?**

>IF NO: That is not a problem. I can take notes while we’re talking so I don’t miss anything important, though that means we might proceed through the interview more slowly than usual. I could also arrange for a colleague to take notes during the interview. [RESCHEDULE IF NEEDED]

>IF YES, perfect, let’s get started.

- [IF UNKNOWN] Based on the background information we’ve received, I am not sure whether your organization currently delivers A-CRA. Can you confirm whether or not A-CRA is delivered by clinicians at your organization?

>IF NO: BEGIN NON-SUSTAINER INTERVIEW

>IF YES: BEGIN SUSTAINER INTERVIEW

**Sustainer Interview – CLINICAL SUPERVISOR & CLINICIAN**

1. Based on the background information we’ve received, it sounds like your agency currently delivers the Adolescent Community Reinforcement Approach or A-CRA. Is this correct?

🞏 No – ***SWITCH TO NON-SUSTAINER INTERVIEW***🞏 Yes – ***CONTINUE***

1. Tell me a little bit about the substance use services at your agency. What services are available? What types of clients do you serve? (e.g., age range, services for specific substances).
2. What youth age range do you use A-CRA with at your agency? Throughout this interview, by “youth” we typically mean ages 12 through 17 (or through age 24 if your A-CRA training included young adults).
3. What is your primary professional role(s) at your clinic/site?

🞏 Clinical Supervisor – ***SWITCH TO SUPERVISOR ONLY VERSION***

🞏 Counselor or Clinician – ***CONTINUE***
🞏 Clinical Supervisor and Counselor/Clinician ***– CONTINUE HERE WITH SUPERVISOR + CLINICIAN VERSION***

🞏 Other, please describe: _____________ (e.g., administrator, grant manager**) *– SWITCH TO SUPERVISOR VERSION***

1. Did you participate in any SAMHSA/CSAT-funded A-CRA training initiatives in your state? Yes or No? *[IF NEEDED, PROMPT AS RELEVANT: Our records do/do not indicate that you received training in A-CRA as part of a SAMHSA CSAT grant under the [initiative] in [grants years]. (IF KNOWN) In your state, I believe this initiative was called [NAME]. So would it be accurate to say you did/did not participate in your state’s A-CRA training initiative?]*

🞏 No **– *SKIP TO QUESTION 6***🞏 Yes **– *CONTINUE***

5A. Between what dates did you work on the project? Your best guess is fine.

1. What role(s) have you served in delivering A-CRA? *[MARK ALL THAT APPLY]*

🞏 A-CRA Therapist
🞏 A-CRA Clinical Supervisor
🞏 Other (Please describe: ____________________________________________________)
🞏 No Role in delivering ACRA

1. Have you received any A-CRA certifications? This means that you completed all training requirements (such as coaching and submission of recorded sessions) and received written documentation of the certification. *[MARK ALL THAT APPLY] [IF NEEDED, PROMPT: A-CRA supervisor certification is different from A-CRA certification.  It refers to being certified to provide A-CRA clinical supervision].*

🞏 First-level clinician certification (passed 9 A-CRA procedures)
🞏 Full clinician certification (passed all 19 A-CRA procedures)
🞏 Supervisor certification
🞏 A-CRA-TAY (Transitional Age Youth) certification

🞏 No, never certified in A-CRA

1. What do you think of A-CRA as a treatment for youth? Please note that throughout this interview, by “youth” we mean the population served during your SAMHSA CSAT grant periods.
2. Does your program currently offer any different types of treatment for youth with substance use disorders besides A-CRA?

🞏 No – ***SKIP TO QUESTION 11***🞏 Yes – ***CONTINUE***🞏 Don’t Know – ***SKIP TO QUESTION 11***

1. What are those treatments called? *[IF NEEDED, PROMPT: Examples of other treatments include Motivational Enhancement Therapy (MET), Cognitive Behavioral Therapy (CBT), Multidimensional Family Therapy (MDFT), 12-step facilitation, and supportive counseling as well as medication treatments like Suboxone or Naltrexone. OR you can just describe the treatment. Is it outpatient? How long does it last? # of sessions/days/months?]*
2. Over the past 6 months, approximately how many youth received substance use treatment at your agency?
3. Approximately how many youth received A-CRA over the past 6 months?

1. Currently, how many clinicians at your agency treat youth with substance use problems?
2. Currently, how many of the clinicians at your agency have received an A-CRA certification? *[IF NEEDED, PROMPT: By certified, we mean they completed all training requirements – such as coaching and submission of recorded sessions – and received written documentation of certification. This includes first-level, full, transitional age youth, and supervisor certifications.]*
3. How many clinicians deliver A-CRA at your agency?
4. Next, I’d like to ask you about your agency’s plan to continue A-CRA clinical supervision support. Does your agency plan to maintain a Clinical Supervisor focused on A-CRA?

🞏 No— ***SKIP TO QUESTION 17***

🞏 Yes
🞏 Don’t Know

16A. Does your agency plan to support Clinical Supervisor and Counselor time for supervision?

🞏 No
🞏 Yes
🞏 Don’t Know

16B. Does your agency plan to support the Clinical Supervisor to listen to recorded therapy sessions and provide individualized feedback to counselors?

🞏 No
🞏 Yes
🞏 Don’t Know

1. Tell me about any ways in which your organization collaborates with other organizations to sustain A-CRA. *[IF NEEDED, PROMPT: For example, you may have engaged in training or support activities with your state substance use services authority and/or Chestnut Health Systems or have partnered with universities or research institutions on research related to A-CRA].*
2. Tell me about any planning that has been done to ensure resources are available to continue A-CRA. By resources, we mean things needed to support A-CRA. For example, this could include things like money, staff, supervision, training, and A-CRA manuals. [*IF NEEDED, PROMPT: By strategic planning, we mean the process by which an organization defines its strategy or direction and makes decisions about how to allocate resources to pursue the strategy.]*
3. Can you please describe any policies that support A-CRA delivery? These could include external policies, such as funding source and billing rules that support A-CRA, as well as internal policies at your organization. *[IF NEEDED, PROMPT: By policies, we mean either organizational, state, national, or local policies. Here are some examples:*

*-State/county/health insurance co requires that we use an evidence-based treatment (EBT) and A-CRA is an EBT*

*-Our agency’s mission emphasizes involvement of the family, A-CRA does that]*

1. What about policies (external or internal) that interfere with A-CRA delivery? *[IF NEEDED, PROMPT: By policies, we mean either organizational, state, national, or local policies. Here are some examples:*

*-if a state decided to require that substance use clinics deliver a different treatment (other than A-CRA), this policy might interfere with the delivery of A-CRA.*

*- if certain insurance plans stopped reimbursing for A-CRA or the full number of sessions, this policy would interfere with A-CRA delivery.]*

1. Does A-CRA meet the needs of the populations you serve? How so or why not?
2. Can you tell me about any pressure your organization experienced to continue delivering A-CRA or to discontinue its use? *[IF NEEDED, PROMPT: Pressure to deliver A-CRA could come from a variety of sources. For ex., organizations might experience pressure if there is some financial incentive to deliver A-CRA. If a state department of health decides its mission is to deliver evidence-based treatment for substance use, this could also be seen as pressure.]*
3. Would you say staff are supportive or reluctant to use A-CRA? Can you give me an example of what people have said or done to indicate their supportiveness or reluctance toward A-CRA?
4. Next, I would like to know what factors helped efforts to implement and sustain A-CRA in your organization. Please let me know if these factors were important in your organization, and if yes, how so. *[PROBE AS NEEDED: What factors were most helpful in that domain? Can you give me some examples? Were there important changes in which factors were most helpful once funding ended?]*

24A. Would you say characteristics of A-CRA helped efforts to implement and sustain A-CRA in your organization? Yes or No?

*[IF NEEDED: for example, treatment content, structure of sessions, etc.]*

🞏 No
🞏 Yes

IF YES, please describe: When was that factor most impactful? Was it during the CSAT grant funding period, after the CSAT funding ended, or throughout?

24B. Would you say characteristics of key individuals helped efforts to implement and sustain A-CRA in your organization? Yes or No?

*[IF NEEDED: “key individuals” are people who had a major positive impact on A-CRA implementation; could be within or outside of your treatment organization]*

🞏 No
🞏 Yes

IF YES, please describe: When was that factor most impactful? Was it during the CSAT grant funding period, after the CSAT funding ended, or throughout?

24C. Would you say client perspectives on A-CRA helped efforts to implement and sustain A-CRA in your organization? Yes or No?

*[IF NEEDED: for example, if clients found A-CRA acceptable, appropriate, feasible, etc.]*

🞏 No
🞏 Yes

IF YES, please describe: When was that factor most impactful? Was it during the CSAT grant funding period, after the CSAT funding ended, or throughout?

24D. Would you say helpful factors within your organization helped efforts to implement and sustain A-CRA in your organization? Yes or No?

*[IF NEEDED: for example, organizational leadership, staffing patterns, scheduling appointments, etc.]*

🞏 No
🞏 Yes

IF YES, please describe: When was that factor most impactful? Was it during the CSAT grant funding period, after the CSAT funding ended, or throughout?

24E. Would you say helpful factors outside your organization helped efforts to implement and sustain A-CRA in your organization? Yes or No?

*[IF NEEDED: for example, state leadership, federal support, community factors]*

🞏 No
🞏 Yes

IF YES, please describe: When was that factor most impactful? Was it during the CSAT grant funding period, after the CSAT funding ended, or throughout?

24F. Would you say partnerships with other organizations helped efforts to implement and sustain A-CRA in your organization? Yes or No?

🞏 No
🞏 Yes

IF YES, please describe: When was that factor most impactful? Was it during the CSAT grant funding period, after the CSAT funding ended, or throughout?

24G. Would you say funding helped efforts to implement and sustain A-CRA in your organization?  Yes or No?

🞏 No
🞏 Yes

IF YES, please describe: When was that factor most impactful? Was it during the CSAT grant funding period, after the CSAT funding ended, or throughout?

24H. Were there other helpful factors during or after the grant period?

🞏 No
🞏 Yes

IF YES, please describe: When was that factor most impactful? Was it during the CSAT grant funding period, after the CSAT funding ended, or throughout?

1. Now, I’m going ask how the same six categories of factors may have included barriers that hindered efforts to implement and sustain A-CRA in your organization. Please let me know if these factors were important in your organization, and if yes, how so. *[PROBE AS NEEDED: What factors were the biggest barriers in that domain? Can you give me some examples? Were there important changes in which factors were the biggest barriers once funding ended?]*

25A. Would you say characteristics of A-CRA hindered efforts to implement and sustain A-CRA in your organization? Yes or No?

*[IF NEEDED: for example, treatment content, structure of sessions, etc.]*

🞏 No
🞏 Yes

IF YES, please describe: When was that factor most impactful? Was it during the CSAT grant funding period, after the CSAT funding ended, or throughout?

25B. Would you say challenges with key individuals hindered efforts to implement and sustain A-CRA in your organization? Yes or No?

*[IF NEEDED: “key individuals” are people who presented major challenges for A-CRA implementation; could be within or outside of your treatment organization]*

🞏 No
🞏 Yes

IF YES, please describe: When was that factor most impactful? Was it during the CSAT grant funding period, after the CSAT funding ended, or throughout?

25C. Would you say client perspectives on A-CRA hindered efforts to implement and sustain A-CRA in your organization? Yes or No?

*[IF NEEDED: for example, if clients found A-CRA acceptable, appropriate, feasible, etc.]*

🞏 No
🞏 Yes

IF YES, please describe: When was that factor most impactful? Was it during the CSAT grant funding period, after the CSAT funding ended, or throughout?

25D. Would you say challenges within your organization hindered efforts to implement and sustain A-CRA in your organization? Yes or No?

*[IF NEEDED: for example, organizational leadership, staffing patterns, scheduling appointments, etc.]*

🞏 No
🞏 Yes

IF YES, please describe: When was that factor most impactful? Was it during the CSAT grant funding period, after the CSAT funding ended, or throughout?

25E. Would you say challenges outside your organization hindered efforts to implement and sustain A-CRA in your organization? Yes or No?

*[IF NEEDED: for example, state leadership, federal support, community factors]*

🞏 No
🞏 Yes

IF YES, please describe: When was that factor most impactful? Was it during the CSAT grant funding period, after the CSAT funding ended, or throughout?

25F. Would you say issues in partnerships with other organizations hindered efforts to implement and sustain A-CRA in your organization? Yes or No?

🞏 No
🞏 Yes

IF YES, please describe: When was that factor most impactful? Was it during the CSAT grant funding period, after the CSAT funding ended, or throughout?

25G. Would you say funding hindered efforts to implement and sustain A-CRA in your organization? Yes or No?

🞏 No
🞏 Yes

IF YES, please describe: When was that factor most impactful? Was it during the CSAT grant funding period, after the CSAT funding ended, or throughout?

25H. Were there other barriers during or after the grant period?

🞏 No
🞏 Yes

IF YES, please describe: When was that factor most impactful? Was it during the CSAT grant funding period, after the CSAT funding ended, or throughout?

1. How many staff supervise clinicians who treat youth with substance use problems?
2. Do you supervise clinicians who deliver A-CRA?

🞏 No
🞏 Yes

🞏 Don’t Know

1. How many others at your agency supervise clinicians delivering A-CRA?
2. Have you personally certified any clinicians in A-CRA?

🞏 No— ***SKIP TO QUESTION 46***

🞏 Yes— ***CONTINUE***

*[IF NEEDED, PROMPT: By certified, we mean they completed all training requirements – such as coaching and submission of recorded sessions – and received written documentation of certification. Respond “yes” if you have personally certified anyone with the first-level, full, transitional age youth, and/or supervisor certifications.]*

1. You mentioned that you personally had certified one or more clinicians in A-CRA at your organization. Can you please describe the certification process to me?

The clinician was asked to:

🞏 Read the A-CRA manual

🞏 Take an online A-CRA research course

🞏 Pass an A-CRA quiz with a score of 80% or higher

🞏 Attend a Chestnut or Robert J. Meyers A-CRA initial training OR attend an in-house training

🞏 Participate in regular coaching calls with Chestnut or regular supervision with in-house Clinical Supervisor (respondent) during certification (regular= @ every other week)

🞏 Regularly record therapy sessions for in-house Clinical Supervisor (respondent) review/Chestnut’s review (regular= at least some sessions weekly))

🞏 Demonstrate competency in General Clinical Skills on the DSRs

🞏 Demonstrate competency in the following A-CRA procedures:

Functional Analysis of Use

Functional Analysis of Pro-social behavior

Happiness Scale

Treatment Plan/Goals of Counseling

Communication Skills

Problem Solving Skills

Adolescent-Caregiver Relationship Skills, and

Homework based of 3 or better on all components of a given procedure using the A-CRA rating manual?

If in-house training was provided, did it:

🞏 Include didactic information about A-CRA procedures?

🞏 Modeling or review of audio recordings of procedures that were well done

🞏 The opportunity to role play procedures

🞏 Other, please explain_____________________________________

**Next, I'm going to ask you a series of questions about how the certification process currently works in your agency. Please respond by saying “True," "False," or "don't know" if you are unsure.**

1. When I decide to pass a clinician on a procedure it is based on ratings of 1 or more on every component of a procedure. Remember that each component of a procedure is rated on a 1 to 5 scale.

🞏 False
🞏 True
🞏 Don’t Know

🞏 NOT APPLICABLE - DONE BY CHESTNUT HEALTH SYSTEMS

1. Communication skills is a procedure that people have to pass to attain certification.

🞏 False
🞏 True
🞏 Don’t Know

1. I review recorded sessions during the certification process.

🞏 False
🞏 True
🞏 Don’t Know

🞏 NOT APPLICABLE - DONE BY CHESTNUT HEALTH SYSTEMS

1. I am required to sit in sessions with clinicians during the certification process.

🞏 False
🞏 True
🞏 Don’t Know

1. Clinicians record one or two of their sessions.

🞏 False
🞏 True
🞏 Don’t Know

1. I refer to the A-CRA rating manual when rating session recordings.

🞏 False
🞏 True
🞏 Don’t Know

🞏 NOT APPLICABLE - DONE BY CHESTNUT HEALTH SYSTEMS

1. Clinicians are required to take a knowledge test as part of the certification process.

🞏 False
🞏 True
🞏 Don’t Know

1. Clinicians are not required to pass General Clinical Skills as part of the certification process.

🞏 False
🞏 True
🞏 Don’t Know

1. Each clinician has a certification workbook.

🞏 False
🞏 True
🞏 Don’t Know

1. I complete the A-CRA checklist when I am listening to a recorded session during or after the certification process.

🞏 False
🞏 True
🞏 Don’t Know

🞏 NOT APPLICABLE - DONE BY CHESTNUT HEALTH SYSTEMS

1. Clinicians are required to read the A-CRA manual during the training process.

🞏 False
🞏 True
🞏 Don’t Know

1. Time is set aside for training clinicians in A-CRA

🞏 False
🞏 True
🞏 Don’t Know

1. During training clinicians are required to practice procedures with role-plays.

🞏 False
 🞏 True
 🞏 Don’t Know

1. Adolescent-Caregiver Relationship Skills is one of the procedures for basic certification.

🞏 False
🞏 True
🞏 Don’t Know

1. It doesn't matter if clinicians show competency in all of the additional procedures as well.

🞏 False
🞏 True
🞏 Don’t Know

1. On average, approximately how many session recordings do clinicians at your site complete to reach first-level certification (pass first 9 A-CRA procedures)? Your best guess is fine.
2. Do you have a copy of the A-CRA manual? *[IF NEEDED, PROMPT: If you have access to a shared copy of the manual through your organization, electronically, etc. that counts as having a copy.]*

🞏 No – ***SKIP TO QUESTION 49***

🞏 Yes – ***CONTINUE***

1. In the past six-months, how often would you say you used the manual? *[IF NEEDED, PROMPT: By use, we mean a reference to the manual. It could mean a quick review of key session content in preparation for a session, or a thorough read-through in order to master the material.]*

🞏 Never
🞏 A few times per year, or less
🞏 About once a month
🞏 A few times per month
🞏 Weekly
🞏 Daily

1. In the past six-months, how often do you provide group clinical supervision? *[IF NEEDED, PROMPT:  This includes any supervision you provided; not supervision you received. Our focus is on A-CRA supervision but you can include any supervision where A-CRA would be discussed – it does not need to be exclusively supervision for A-CRA cases.]*

🞏 Never
🞏 Once a week
🞏 Every two weeks
🞏 Monthly
🞏 Other, please describe how often: ___________________________________

1. In the past six-months, how often do you provide individual clinical supervision? *[IF NEEDED, PROMPT: This includes any supervision you provided; not supervision you received. Our focus is on A-CRA supervision but you can include any supervision where A-CRA would be discussed – it does not need to be exclusively supervision for A-CRA cases.]*

🞏 Never
🞏 Once a week
🞏 Every two weeks
🞏 Monthly
🞏 Other, please describe how often: ___________________________________

**IF QUESTION 49 = Never AND QUESTION 50 = Never, SKIP TO QUESTION 61**

**Which of the following happens during supervision sessions?**

1. My supervisor asks how my week has gone.

🞏 No
🞏 Yes

1. My supervisor discusses agency paperwork requirements.

🞏 No
🞏 Yes

1. My supervisor reviews my A-CRA case review report.

🞏 No
🞏 Yes

1. My supervisor asks me if I have any problem cases.

🞏 No
🞏 Yes

1. My supervisor reviews a recorded session with me and tells me what I have done well.

🞏 No
🞏 Yes

1. My supervisor reviews a recorded session with me and gives me suggestions about how I can improve my treatment delivery

🞏 No
🞏 Yes

1. My supervisor observes a live session and tells me what I have done well.

🞏 No
🞏 Yes

1. My supervisor observes a live session and gives me suggestions about how I can improve my treatment delivery

🞏 No
🞏 Yes

1. My supervisor asks me about my personal problems.

🞏 No
🞏 Yes

1. My supervisor role plays with me the correct way to do a procedure.

🞏 No
🞏 Yes

1. How many new clinicians have been trained in A-CRA in the past six-months in the organization?
2. How many new clinicians have been trained in A-CRA in the past six-months at a Chestnut or Robert J. Meyers training?

**[IF ANSWER TO QUESITON 61 > or = to ANSWER TO QUESTION 62 CONTINUE; OTHERWISE, SKIP TO QUESTION 65]**

1. Are there training agendas for your trainings?

🞏 No – ***SKIP TO QUESTION 65***🞏 Yes – ***CONTINUE***

**[IF QUESTION 63=Yes AND ANSWER TO QUESTION 61>0 AND ANSWER TO QUESTION 61 >= ANSWER TO QUESTION 62]**

1. Will you please send me (email, mail, fax) a copy of the agenda?

🞏 No
🞏 Yes

**Currently, when you are introducing A-CRA to a new client…**

1. How many sessions do you tell the client receiving A-CRA they will have?
2. How many weeks do you tell the client receiving A-CRA the treatment will take?
3. How has the COVID-19 pandemic affected substance use treatment services at your organization?
4. How has it affected the ability of your organization to sustain A-CRA services?
5. What have been the most significant changes affecting your A-CRA program in response to COVID-19? *[PROBE AS NEEDED: Changes could include things like…*

*…Changes in referrals to A-CRA*

*…Changes in how A-CRA assessment, treatment, or case management services are delivered*

*…Changes in staffing*

*…Remote work*

*…Telehealth service delivery*

*Were there other changes? If so, please describe:]*

1. Have there been changes made in response to COVID-19 that have been beneficial, and will those changes continue beyond the pandemic? *[PROBE AS NEEDED: These may be some of the changes you already described, or may be different. Changes could include things like…*

*…Changes in referrals to A-CRA*

*…Changes in how A-CRA assessment, treatment, or case management services are delivered*

*…Changes in staffing*

*…Remote work*

*…Telehealth service delivery*

*Were there other beneficial changes? If so, please describe:]*

**OK, we are almost done. Next, I want to ask you about your general impressions regarding the SAMHSA CSAT-funded A-CRA project and then have a few questions about you.**

1. If you had a chance to participate in a SAMHSA CSAT project again, would you consider it?

🞏 No
🞏 Yes

1. Why/why not?
2. Is there anything you would change about the SAMHSA CSAT project in order to improve the sustainment of A-CRA at your agency?
3. Is there anything else you would like to share related to the topic of sustaining A-CRA at your agency?

[TURN OFF RECORDER]

That's all the interview questions I have. Thank you for providing this information.

[IF SENDING WEB SURVEY (FULL OR ABBREVIATED) AS USUAL]

We will soon be sending you the link to the web survey in order to provide a more complete picture of the treatment offered to youth at your organization and about organizational and clinical support. We will send it within a few days and will send your $50 Amazon e-gift card as soon as the survey is complete.

[IF PARTICIPANT WILL BE INTERVIEWED AGAIN, complete the tracking module]

**Non-Sustainer Interview – CLINICAL SUPERVISOR & CLINICIAN**

1. Based on the background information we’ve received, it sounds like your agency currently does not deliver the Adolescent Community Reinforcement Approach or A-CRA anymore. Is this correct?

🞏 No – ***SWITCH TO SUSTAINER INTERVIEW*** 🞏 Yes – ***CONTINUE***

1. Tell me a little bit about the substance use services at your agency. What services are available? What types of clients do you serve? (e.g., age range, services for specific substances).
2. What youth age range do you use A-CRA with at your agency? Throughout this interview, by “youth” we typically mean ages 12 through 17 (or through age 24 if your A-CRA training included young adults).
3. What is your primary professional role(s) at your clinic/site?

🞏 Clinical Supervisor – ***SWITCH TO SUPERVISOR ONLY VERSION***

🞏 Counselor or Clinician – ***SWITCH TO CLINICIAN ONLY VERSION***
🞏 Clinical Supervisor and Counselor/Clinician *–* ***CONTINUE HERE WITH SUPERVISOR + CLINICIAN VERSION***

🞏 Other, please describe: ____________________ (e.g., administrator, grant manager) *–* ***SWITCH TO SUPERVISOR VERSION***

1. Did you participate in any SAMHSA/CSAT-funded A-CRA training initiatives in your state? Yes or No? *[IF NEEDED, PROMPT AS RELEVANT: Our records do/do not indicate that you received training in A-CRA as part of a SAMHSA CSAT grant under the [initiative] in [grants years]. (IF KNOWN) In your state, I believe this initiative was called [NAME]. So would it be accurate to say you did/did not participate in your state’s A-CRA training initiative?]*

🞏 No – ***SKIP TO QUESTION 6*** 🞏 Yes – ***CONTINUE***

5A. Between what dates did you work on the project? Your best guess is fine.

1. What role(s) did you serve while A-CRA was being delivered at your agency? *[MARK ALL THAT APPLY]*

🞏 A-CRA Therapist
🞏 A-CRA Supervisor
🞏 Other (Please describe: ____________________________________________________)
🞏 No Role in delivering ACRA

1. Have you received any A-CRA certifications? This means that you completed all training requirements (such as coaching and submission of recorded sessions) and received written documentation of the certification. [MARK ALL THAT APPLY]

🞏 First-level clinician certification (passed 9 A-CRA procedures)
🞏 Full clinician certification (passed all 19 A-CRA procedures)
🞏 Supervisor certification
🞏 A-CRA-TAY (Transitional Age Youth) certification

🞏 No, never certified in A-CRA

*[IF NEEDED, PROMPT: A-CRA supervisor certification is different from A-CRA certification.  It refers to being certified to provide A-CRA clinical supervision].*

1. What do you think of A-CRA as a treatment for youth?
2. When did you/your agency stop delivering A-CRA? If you do not know the exact date, please give your best estimate.

Month/Year: ______ /______

1. What were the main reasons you/your agency stopped delivering A-CRA?
2. What would have increased your desire to continue delivering A-CRA?
3. What would have increased your ability to continue delivering A-CRA?
4. Does your program currently offer any different types of treatment for youth with substance use disorders besides A-CRA?

🞏 No – ***SKIP TO QUESTION 15***🞏 Yes – ***CONTINUE***
🞏 Don’t Know – ***SKIP TO QUESTION 15***

1. What are those treatments called? *[IF NEEDED, PROMPT: Examples of other treatments include Motivational Enhancement Therapy (MET), Cognitive Behavioral Therapy (CBT), Multidimensional Family Therapy (MDFT), 12-step facilitation, and supportive counseling as well as medication treatments like Suboxone or Naltrexone. OR you can just describe the treatment. Is it outpatient? How long does it last? # of sessions/days/months?]*
2. Over the past 6 months, approximately how many youth received substance use treatment at your agency?

1. Approximately how many youth received A-CRA over the past 6 months?

**IF QUESTION 16 = 0** 16A. Approximately how many youth received A-CRA during the six-month period, right before A-CRA treatment delivery ended?

1. Currently, how many clinicians at your agency treat youth with substance use problems?
2. Currently, how many of the clinicians at your agency have received an A-CRA certification? *[IF NEEDED, PROMPT: By certified, we mean they completed all training requirements – such as coaching and submission of recorded sessions – and received written documentation of certification. This includes first-level, full, transitional age youth, and supervisor certifications.]*

**IF QUESTION 18 = 0** 18A. Approximately how many clinicians at your agency had received an A-CRA certification during the six-month period, right before A-CRA treatment delivery ended?

1. Next, I’d like to ask you about your agency’s A-CRA clinical supervision support during the last six months it was delivered. Was your agency able to maintain a Clinical Supervisor focused on A-CRA?

🞏 No – ***SKIP TO QUESTION 20***

🞏 Yes
🞏 Don’t Know

19A. Did your agency support Clinical Supervisor and Counselor time for supervision?

🞏 No
🞏 Yes
🞏 Don’t Know

19B. Did your agency support the Clinical Supervisor to listen to recorded therapy sessions and provide individualized feedback to counselors?

🞏 No
🞏 Yes
🞏 Don’t Know

1. Tell me about any ways in which your organization collaborates with other organizations – especially anything related to A-CRA. *[IF NEEDED, PROMPT: For example, you may have engaged in training or support activities with your state substance use services authority and/or Chestnut Health Systems, or have partnered with universities or research institutions on research related to A-CRA].*
2. Was there any planning done to ensure resources were available to continue A-CRA beyond the initial funding period? By resources, we mean things needed to support A-CRA. For example, this could include things like money, staff, supervision, training, and A-CRA manuals. [*IF NEEDED, PROMPT: By strategic planning, we mean the process by which an organization defines its strategy or direction and makes decisions about how to allocate resources to pursue the strategy.]*
3. Can you please describe any policies that supported A-CRA delivery? These could include external policies, such as funding source and billing rules that support A-CRA, as well as internal policies at your organization. *[IF NEEDED, PROMPT: By policies, we mean either organizational, state, national, or local policies. Here are some examples:*

*-State/county/health insurance co requires that we use an evidence-based treatment (EBT) and A-CRA is an EBT*

*-Our agency’s mission emphasizes the involvement of the family, A-CRA does that]*

1. What about policies (external or internal) that interfered with A-CRA delivery? *[IF NEEDED, PROMPT:  By policies, we mean either organizational, state, national, or local policies. Here are some examples:*

*-if a state decided to require that substance use clinics deliver a different treatment (other than A-CRA), this policy might interfere with the delivery of A-CRA.*

*- if certain insurance plans stopped reimbursing for A-CRA or the full number of sessions, this policy would interfere with A-CRA delivery.]*

1. Did A-CRA meet the needs of the populations you serve? How so or why not?
2. Can you tell me about any pressure your organization experienced to continue delivering A-CRA or to discontinue its use? *[IF NEEDED, PROMPT:  Pressure to deliver A-CRA could come from a variety of sources. For example, organizations might experience pressure if there is some financial incentive to deliver A-CRA. If a state department of health decides its mission is to deliver evidence-based treatment for substance use, this could also be seen as pressure.]*
3. Would you say staff were supportive or reluctant to use A-CRA? Can you give me an example of what people have said or done to indicate their supportiveness or reluctance toward A-CRA?
4. Next, I would like to know what factors helped efforts to implement and sustain A-CRA in your organization. Please let me know if these factors were important in your organization, and if yes, how so. *[PROBE AS NEEDED: What factors were most helpful in that domain? Can you give me some examples? Were there important changes in which factors were most helpful once funding ended?]*

27A. Would you say characteristics of A-CRA helped efforts to implement and sustain A-CRA in your organization? Yes or No?

*[IF NEEDED: for example, treatment content, structure of sessions, etc.]*

🞏 No
🞏 Yes

IF YES, please describe: When was that factor most impactful? Was it during the CSAT grant funding period, after the CSAT funding ended, or throughout?

27B. Would you say characteristics of key individuals helped efforts to implement and sustain A-CRA in your organization? Yes or No?

*[IF NEEDED: “key individuals” are people who had a major positive impact on A-CRA implementation; could be within or outside of your treatment organization]*

🞏 No
🞏 Yes

IF YES, please describe: When was that factor most impactful? Was it during the CSAT grant funding period, after the CSAT funding ended, or throughout?

27C. Would you say client perspectives on A-CRA helped efforts to implement and sustain A-CRA in your organization? Yes or No?

*[IF NEEDED: for example, if clients found A-CRA acceptable, appropriate, feasible, etc.]*

🞏 No
🞏 Yes

IF YES, please describe: When was that factor most impactful? Was it during the CSAT grant funding period, after the CSAT funding ended, or throughout?

27D. Would you say helpful factors within your organization helped efforts to implement and sustain A-CRA in your organization? Yes or No?

*[IF NEEDED: for example, organizational leadership, staffing patterns, scheduling appointments, etc.]*

🞏 No
🞏 Yes

IF YES, please describe: When was that factor most impactful? Was it during the CSAT grant funding period, after the CSAT funding ended, or throughout?

27E. Would you say helpful factors outside your organization helped efforts to implement and sustain A-CRA in your organization? Yes or No?

*[IF NEEDED: for example, state leadership, federal support, community factors]*

🞏 No
🞏 Yes

IF YES, please describe: When was that factor most impactful? Was it during the CSAT grant funding period, after the CSAT funding ended, or throughout?

27F. Would you say partnerships with other organizations helped efforts to implement and sustain A-CRA in your organization? Yes or No?

🞏 No
🞏 Yes

IF YES, please describe: When was that factor most impactful? Was it during the CSAT grant funding period, after the CSAT funding ended, or throughout?

27G. Would you say funding helped efforts to implement and sustain A-CRA in your organization? Yes or No?

🞏 No
🞏 Yes

IF YES, please describe: When was that factor most impactful? Was it during the CSAT grant funding period, after the CSAT funding ended, or throughout?

27H. Were there other helpful factors during or after the grant period?

🞏 No
🞏 Yes

IF YES, please describe: When was that factor most impactful? Was it during the CSAT grant funding period, after the CSAT funding ended, or throughout?

1. Now, I’m going ask how the same six categories of factors may have included barriers that hindered your efforts to implement and sustain A-CRA in your organization. Please let me know if these factors were important at your organization, and if yes, how so. *[PROBE AS NEEDED: What factors were the biggest barriers in that domain? Can you give me some examples? Were there important changes in which factors were the biggest barriers once funding ended?]*

28A. Would you say characteristics of A-CRA hindered efforts to implement and sustain A-CRA in your organization? Yes or No?

*[IF NEEDED: for example, treatment content, structure of sessions, etc.]*

🞏 No
🞏 Yes

IF YES, please describe: When was that factor most impactful? Was it during the CSAT grant funding period, after the CSAT funding ended, or throughout?

28B. Would you say challenges with key individuals hindered efforts to implement and sustain A-CRA in your organization? Yes or No?

*[IF NEEDED: “key individuals” are people who presented major challenges for A-CRA implementation; could be within or outside of your treatment organization]*

🞏 No
🞏 Yes

IF YES, please describe: When was that factor most impactful? Was it during the CSAT grant funding period, after the CSAT funding ended, or throughout?

28C. Would you say client perspectives on A-CRA hindered efforts to implement and sustain A-CRA in your organization? Yes or No?

*[IF NEEDED: for example, if clients found A-CRA acceptable, appropriate, feasible, etc.]*

🞏 No
🞏 Yes

IF YES, please describe: When was that factor most impactful? Was it during the CSAT grant funding period, after the CSAT funding ended, or throughout?

28D. Would you say challenges within your organization hindered efforts to implement and sustain A-CRA in your organization? Yes or No?

*[IF NEEDED: for example, organizational leadership, staffing patterns, scheduling appointments, etc.]*

🞏 No
🞏 Yes

IF YES, please describe: When was that factor most impactful? Was it during the CSAT grant funding period, after the CSAT funding ended, or throughout?

28E. Would you say challenges outside your organization hindered efforts to implement and sustain A-CRA in your organization? Yes or No?

*[IF NEEDED: for example, state leadership, federal support, community factors]*

🞏 No
🞏 Yes

IF YES, please describe: When was that factor most impactful? Was it during the CSAT grant funding period, after the CSAT funding ended, or throughout?

28F. Would you say issues in partnerships with other organizations hindered efforts to implement and sustain A-CRA in your organization? Yes or No?

🞏 No
🞏 Yes

IF YES, please describe: When was that factor most impactful? Was it during the CSAT grant funding period, after the CSAT funding ended, or throughout?

28G. Would you say funding hindered efforts to implement and sustain A-CRA in your organization? Yes or No?

🞏 No
🞏 Yes

IF YES, please describe: When was that factor most impactful? Was it during the CSAT grant funding period, after the CSAT funding ended, or throughout?

28H. Were there other barriers during or after the grant period?

🞏 No
🞏 Yes

IF YES, please describe: When was that factor most impactful? Was it during the CSAT grant funding period, after the CSAT funding ended, or throughout?

1. How many staff supervise clinicians who treat youth with substance use problems?
2. Did you supervise clinicians delivering A-CRA at your agency?

🞏 No
🞏 Yes
🞏 Don’t Know

1. How many others at your agency supervised clinicians delivering A-CRA?

1. Have you personally certified any clinicians in A-CRA?

🞏 No – ***SKIP TO QUESTION 49***

🞏 Yes— ***CONTINUE***

*[IF NEEDED, PROMPT: By certified, we mean they completed all training requirements – such as coaching and submission of recorded sessions – and received written documentation of certification. Respond “yes” if you have personally certified anyone with the first-level, full, transitional age youth, and/or supervisor certifications.]*

1. You mentioned that you personally had certified one or more clinicians in A-CRA at your organization. Can you please describe the certification process to me?

The clinician was asked to:

🞏 Read the A-CRA manual

🞏 Take an online A-CRA research course

🞏 Pass an A-CRA quiz with a score of 80% or higher

🞏 Attend a Chestnut or Robert J. Meyers A-CRA initial training OR attend an in-house training

🞏 Participate in regular coaching calls with Chestnut or regular supervision with in-house Clinical Supervisor (respondent) during certification (regular= @ every other week)

🞏 Regularly record therapy sessions for in-house Clinical Supervisor (respondent) review/Chestnut’s review (regular= at least some sessions weekly))

🞏 Demonstrate competency in General Clinical Skills on the DSRs

🞏 Demonstrate competency in the following A-CRA procedures:

Functional Analysis of Use

Functional Analysis of Pro-social behavior

Happiness Scale

Treatment Plan/Goals of Counseling

Communication Skills

Problem Solving Skills

Adolescent-Caregiver Relationship Skills, and

Homework based of 3 or better on all components of a given procedure using the A-CRA rating manual?

If in-house training was provided, did it:

🞏 Include didactic information about A-CRA procedures?

🞏 Modeling or review of audio recordings of procedures that were well done

🞏 The opportunity to role play procedures

🞏 Other, please explain_____________________________________

**Next, I'm going to ask you a series of questions about how the certification process worked in your agency. Please respond by saying “True," "False," or "don't know" if you are unsure.**

1. When I decided to pass a clinician on a procedure it was based on ratings of 1 or more on every component of a procedure. Remember that each component of a procedure is rated on a 1 to 5 scale.

🞏 False
🞏 True
🞏 Don’t Know

🞏 NOT APPLICABLE - DONE BY CHESTNUT HEALTH SYSTEMS

1. Communication skills was a procedure that people had to pass to attain certification.

🞏 False
🞏 True
🞏 Don’t Know

1. I reviewed recorded sessions during the certification process.

🞏 False
🞏 True
🞏 Don’t Know

🞏 NOT APPLICABLE - DONE BY CHESTNUT HEALTH SYSTEMS

1. I was required to sit in on sessions with clinicians during the certification process.

🞏 False
🞏 True
🞏 Don’t Know

1. Clinicians recorded one or two of their sessions.

🞏 False
🞏 True
🞏 Don’t Know

1. I referred to the A-CRA rating manual when rating session recordings.

🞏 False
🞏 True
🞏 Don’t Know

🞏 NOT APPLICABLE - DONE BY CHESTNUT HEALTH SYSTEMS

1. Clinicians were required to take a knowledge test as part of the certification process.

🞏 False
🞏 True
🞏 Don’t Know

1. Clinicians were not required to pass General Clinical Skills as part of the certification process.

🞏 False
🞏 True
🞏 Don’t Know

1. Each clinician had a certification workbook.

🞏 False
🞏 True
🞏 Don’t Know

1. I completed the A-CRA checklist when I was listening to a recorded session during or after the certification process.

🞏 False
🞏 True
🞏 Don’t Know

🞏 NOT APPLICABLE - DONE BY CHESTNUT HEALTH SYSTEMS

1. Clinicians were required to read the A-CRA manual during the training process.

🞏 False
🞏 True
🞏 Don’t Know

1. Time was set aside for training clinicians in A-CRA

🞏 False
🞏 True
🞏 Don’t Know

1. During training clinicians were required to practice procedures with role-plays.

🞏 False
🞏 True
🞏 Don’t Know

1. Adolescent-Caregiver Relationship Skills was one of the procedures for basic certification.

🞏 False
🞏 True
🞏 Don’t Know

1. It didn’t matter if clinicians showed competency in all of the additional procedures as well.

🞏 False
🞏 True
🞏 Don’t Know

1. On average, approximately how many session recordings did clinicians at your site complete to reach first-level certification (pass first 9 A-CRA procedures)? Your best guess is fine.
2. Have you ever had a copy of the A-CRA manual? *[IF NEEDED, PROMPT: If you have access to a shared copy of the manual through your organization, electronically, etc. that counts as having a copy.]*

🞏 NO – ***SKIP TO QUESTION 54*** 🞏 Yes – ***CONTINUE***

1. Please think about the six-month period, right before A-CRA treatment delivery ended. How often did you use the manual? *[IF NEEDED, PROMPT: By use, we mean a reference to the manual. It could mean a quick review of key session content in preparation for a session, or a thorough read-through in order to master the material.]*

🞏 Never
🞏 A few times per year, or less
🞏 About once a month
🞏 A few times per month
🞏 Weekly
🞏 Daily

1. Do you still use your manual? *[IF NEEDED, PROMPT: By use, we mean a reference to the manual. It could mean a quick review of key session content in preparation for a session, or a thorough read-through in order to master the material.]*

🞏 NO – ***SKIP TO QUESTION 54***🞏 Yes – ***CONTINUE***

1. How often do you use your manual now? *[IF NEEDED, PROMPT: By use, we mean a reference to the manual. It could mean a quick review of key session content in preparation for a session, or a thorough read-through in order to master the material.]*

🞏 Never
🞏 A few times per year, or less
🞏 About once a month
🞏 A few times per month
🞏 Weekly
🞏 Daily

**For the next few questions, please think about the six-month period, right before A-CRA treatment delivery ended.**

1. How often did you provide group clinical supervision? *[IF NEEDED, PROMPT:  This includes any supervision you provided; not supervision you received. Our focus is on A-CRA supervision but you can include any supervision where A-CRA would be discussed – it does not need to be exclusively supervision for A-CRA cases.]*

🞏 Never
🞏 Once a week
🞏 Every two weeks
🞏 Monthly
🞏 Other, please describe how often: ___________________________________

1. Again, please think about the six-month period, right before A-CRA treatment delivery ended. How often did you provide individual clinical supervision? *[IF NEEDED, PROMPT:  This includes any supervision you provided; not supervision you received. Our focus is on A-CRA supervision but you can include any supervision where A-CRA would be discussed – it does not need to be exclusively supervision for A-CRA cases.]*

🞏 Never
🞏 Once a week
🞏 Every two weeks
🞏 Monthly
🞏 Other, please describe how often: ___________________________________

**IF QUESTION 54 = Never AND QUESTION 55= Never, SKIP TO QUESTION 66**

**Thinking about the six-month period, right before A-CRA treatment delivery ended, which of the following happened during supervision sessions?**

1. My supervisor asked how my week had gone.

🞏 No
🞏 Yes

1. (During the six-month period, right before A-CRA treatment delivery ended), my supervisor discussed agency paperwork requirements.

🞏 No
🞏 Yes

1. (During the six-month period, right before A-CRA treatment delivery ended), my supervisor reviewed my A-CRA case review report.

🞏 No
🞏 Yes

1. (During the six-month period, right before A-CRA treatment delivery ended), my supervisor asked me if I had any problem cases.

🞏 No
🞏 Yes

1. (During the six-month period, right before A-CRA treatment delivery ended), my supervisor reviewed a recorded session with me and told me what I did well.

🞏 No
🞏 Yes

1. (During the six-month period, right before A-CRA treatment delivery ended), my supervisor reviewed a recorded session with me and gave me suggestions about how I could improve my treatment delivery

🞏 No
🞏 Yes

1. (During the six-month period, right before A-CRA treatment delivery ended), my supervisor observed a live session and told me what I did well.

🞏 No
🞏 Yes

1. (During the six-month period, right before A-CRA treatment delivery ended), my supervisor observed a live session and gave me suggestions about how I could improve my treatment delivery

🞏 No
🞏 Yes

1. (During the six-month period, right before A-CRA treatment delivery ended), my supervisor asked me about my personal problems.

🞏 No
🞏 Yes

1. (During the six-month period, right before A-CRA treatment delivery ended), my supervisor role played with me the correct way to do a procedure.

🞏 No
🞏 Yes

**Now I’d like to ask you the same questions, but now please think about the current practices in the adolescent treatment program.**

1. How often do you provide group clinical supervision? *[IF NEEDED, PROMPT:  This includes any supervision you provided; not supervision you received. Our focus is on A-CRA supervision but you can include any supervision where A-CRA would be discussed – it does not need to be exclusively supervision for A-CRA cases.]*

🞏 Never
🞏 Once a week
🞏 Every two weeks
🞏 Monthly
🞏 Other, please describe how often: ___________________________________

1. How often do you provide individual clinical supervision?*[IF NEEDED, PROMPT:  This includes any supervision you provided; not supervision you received. Our focus is on A-CRA supervision but you can include any supervision where A-CRA would be discussed – it does not need to be exclusively supervision for A-CRA cases.]*

🞏 Never
🞏 Once a week
🞏 Every two weeks
🞏 Monthly
🞏 Other, please describe how often: ___________________________________

**IF QUESTION 66 = Never AND QUESTION 67= Never, SKIP TO QUESTION 77**

**Thinking about the current practices in your adolescent treatment program, which of the following happens during supervision sessions?**

1. My supervisor asks me how my week has gone.

🞏 No
🞏 Yes

1. My supervisor discusses agency paperwork requirements.

🞏 No
🞏 Yes

1. My supervisor asks me if I have any problem cases.

🞏 No
🞏 Yes

1. My supervisor reviews a recorded session with me and tells me what I have done well.

🞏 No
🞏 Yes

1. My supervisor reviews a recorded session with me and gives me suggestions about how I could improve my treatment delivery.

🞏 No
🞏 Yes

1. My supervisor observes a live session and tells me what I have done well.

🞏 No
🞏 Yes

1. My supervisor observes a live session and gives me suggestions about how I could improve my treatment delivery.

🞏 No
🞏 Yes

1. My supervisor asks me about my personal problems.

🞏 No
🞏 Yes

1. My supervisor role plays with me the correct way to deliver therapy.

🞏 No
🞏 Yes

1. During the six-month period, right before A-CRA treatment delivery ended, how many sessions did you tell the client receiving A-CRA they would have?
2. During the six-month period, right before A-CRA treatment delivery ended, how many weeks did you tell the client receiving A-CRA the treatment would take?
3. How has the COVID-19 pandemic affected substance use treatment services at your organization?
4. When the pandemic began in March 2020, was your organization still delivering A-CRA?

🞏 No – ***SKIP TO QUESTION 83***

🞏 Yes – ***CONTINUE***

🞏 Don’t know – ***SKIP TO QUESTION 82***

1. What have been the most significant changes affecting your A-CRA program in response to COVID-19?

*[PROBE AS NEEDED: Changes could include things like…*

*…A-CRA was discontinued due to COVID-related changes*

*…Changes in referrals to A-CRA*

*…Changes in how A-CRA assessment, treatment, or case management services are delivered*

*…Changes in staffing*

*…Remote work*

*…Telehealth service delivery*

*Were there other changes? If so, please describe:]*

1. Have there been changes made in response to COVID-19 that have been beneficial, and will those changes continue beyond the pandemic? *[PROBE AS NEEDED: These may be some of the changes you already described, or may be different. Changes could include things like…*

*…Changes in referrals to A-CRA*

*…Changes in how A-CRA assessment, treatment, or case management services are delivered*

*…Changes in staffing*

*…Remote work*

*…Telehealth service delivery*

*Were there other beneficial changes? If so, please describe: ]*

**OK, we are almost done. Next, I want to ask you about your general impressions regarding the SAMHSA CSAT-funded A-CRA project and then have a few questions about you.**

1. If you had a chance to participate in a SAMHSA CSAT project again, would you consider it?

🞏 No
🞏 Yes

1. Why/why not?
2. Is there anything you would change about the SAMHSA CSAT project in order to improve the sustainment of A-CRA at your agency?
3. Is there anything else you would like to share related to the topic of sustaining A-CRA at your agency?

[TURN OFF RECORDER]

That's all the interview questions I have. Thank you for providing this information.

[IF SENDING WEB SURVEY (FULL OR ABBREVIATED) AS USUAL]

We will soon be sending you the link to the web survey in order to provide a more complete picture of the treatment offered to youth at your organization and about organizational and clinical support. We will send it within a few days, and will send your $50 Amazon e-gift card as soon as the survey is complete.

[IF PARTICIPANT WILL BE INTERVIEWED AGAIN, complete the tracking module]

***SUPERVISOR INTERVIEW VERBAL CONSENT***

(READ PRIOR TO BEGINNING INTERVIEW/RECORDING)

Hi [SUPERVISOR NAME],

This is [INTERVIEWER NAME] calling from RAND to speak with you about the youth substance use treatment program where you work and any experiences you have with implementing the Adolescent Community Reinforcement Approach (A-CRA) treatment model. Is this still a good time to complete your interview? To ensure confidentiality, make sure you are in a private, secure location for completing the interview.

>IF NO, RESCHEDULE.

>IF YES, great.

We would like a clinician and/or clinical supervisor for youth substance use treatment to answer these questions. Ideally, it should be someone knowledgeable about your A-CRA program. Are you the right person at your organization to participate in this opportunity?

>IF NO: May I please have the contact information of the best contact(s) for youth substance use treatment? [RECORD CONTACT INFORMATION TO FOLLOW-UP]

>IF YES, thanks for confirming.

Before we begin, let me assure you that your responses to these questions will be held in strict confidence. In collaboration with Chestnut Health Systems, we are requesting interviews and surveys from clinicians and supervisors from nearly 20 states that received A-CRA training from Chestnut Health, as part of SAMHSA Center of Substance Abuse Treatment (CSAT) grants received by state substance use services authorities. We will use information from the interviews and surveys to understand how different CSAT funding models influence the sustainability of A-CRA delivery after funding ends. We will also examine information provided by state organizations that received the CSAT grants and data already collected from prior CSAT grantee organizations that implemented A-CRA. We will not attribute comments to specific individuals or programs in any of our reports or publications. Your responses will not be shared with your organization or with SAMHSA.

Today’s interview will last up to 45 minutes. Afterwards, we will ask you to complete a 30-minute online survey on your own to give us a more complete picture. You will receive a $50 Amazon electronic gift card upon completion of the web survey as a thank you for your participation.

Your participation in this discussion and the survey is entirely voluntary. We would like to have your responses to all of the questions. However, if you’re uncomfortable with any question we can skip it, and you can stop the interview at any time. There are no right or wrong answers – we are interested in your perspectives and experiences. Finally, we would like to audio-record the interview to ensure that we capture everything that is said. We will destroy the recording once we confirm we have captured everything in our de-identified notes and transcripts. However, you can still participate in the interview even if you do not give permission to audio-record.

If you have any questions or want to discuss the project further at any time, you may always contact us at [PHONE] or at [EMAIL]. Furthermore, if you have questions about your rights as a research participant or need to report a research-related injury or concern, you can contact RAND's Human Subjects Protection Committee toll-free at (866) 697-5620 or email [hspcinfo@​rand.org](mailto:hspcinfo@​rand.org)​. If you contact the Committee, please reference Study #2020-N0887.

- **Do you have any questions?**
- **Are you willing to take part in this discussion?**

>IF NO: That is not a problem, thank you for your time.

>IF YES, great.

- **Is it ok with you if we audiotape this discussion?**

>IF NO: That is not a problem. I can take notes while we’re talking so I don’t miss anything important, though that means we might proceed through the interview more slowly than usual. I could also arrange for a colleague to take notes during the interview. [RESCHEDULE IF NEEDED]

>IF YES, perfect, let’s get started.

- [IF UNKNOWN] Based on the background information we’ve received, I am not sure whether your organization currently delivers A-CRA. Can you confirm whether or not A-CRA is delivered by clinicians at your organization?

>IF NO: BEGIN NON-SUSTAINER INTERVIEW

>IF YES: BEGIN SUSTAINER INTERVIEW

**Sustainer Interview – SUPERVISOR**

1. Based on the background information we’ve received, it sounds like your agency currently delivers the Adolescent Community Reinforcement Approach or A-CRA. Is this correct?

🞏 No – ***SWITCH TO NON-SUSTAINER INTERVIEW***🞏 Yes – ***CONTINUE***

1. Tell me a little bit about the substance use services at your agency. What services are available? What types of clients do you serve? (e.g., age range, services for specific substances).
2. What youth age range do you use A-CRA with at your agency? Throughout this interview, by “youth” we typically mean ages 12 through 17 (or through age 24 if your A-CRA training included young adults).
3. What is your primary professional role(s) at your clinic/site?

🞏 Clinical Supervisor – ***CONTINUE***

🞏 Counselor or Clinician – ***SWITCH TO CLINICIAN ONLY VERSION***
🞏 Clinical Supervisor and Counselor/Clinician ***– SWITCH TO SUPERVISOR + CLINICIAN VERSION***

🞏 Other, please describe: ____________________ (e.g., administrator, grant manager**) *– CONTINUE***

1. Did you participate in any SAMHSA/CSAT-funded A-CRA training initiatives in your state? Yes or No? *[IF NEEDED, PROMPT AS RELEVANT: Our records do/do not indicate that you received training in A-CRA as part of a SAMHSA CSAT grant under the [initiative] in [grants years]. (IF KNOWN) In your state, I believe this initiative was called [NAME]. So would it be accurate to say you did/did not participate in your state’s A-CRA training initiative?]*

🞏 No **– *SKIP TO QUESTION 6***🞏 Yes **– *CONTINUE***

5A. Between what dates did you work on the project? Your best guess is fine.

1. What role(s) have you served in delivering A-CRA? *[MARK ALL THAT APPLY]*

🞏 A-CRA Therapist
🞏 A-CRA Clinical Supervisor
🞏 Other (Please describe: ____________________________________________________)
🞏 No Role in delivering ACRA

1. Have you received any A-CRA certifications? This means that you completed all training requirements (such as coaching and submission of recorded sessions) and received written documentation of the certification. *[MARK ALL THAT APPLY] [IF NEEDED, PROMPT: A-CRA supervisor certification is different from A-CRA certification.  It refers to being certified to provide A-CRA clinical supervision].*

🞏 First-level clinician certification (passed 9 A-CRA procedures)
🞏 Full clinician certification (passed all 19 A-CRA procedures)
🞏 Supervisor certification

🞏 A-CRA-TAY (Transitional Age Youth) certification

🞏 No, never certified in A-CRA

1. What do you think of A-CRA as a treatment for youth? Please note that throughout this interview, by “youth” we mean the population served during your SAMHSA CSAT grant periods.
2. Does your program currently offer any different types of treatment for youth with substance use disorders besides A-CRA?

🞏 No – ***SKIP TO QUESTION 11***🞏 Yes – ***CONTINUE***🞏 Don’t Know – ***SKIP TO QUESTION 11***

1. What are those treatments called? *[IF NEEDED, PROMPT: Examples of other treatments include Motivational Enhancement Therapy (MET), Cognitive Behavioral Therapy (CBT), Multidimensional Family Therapy (MDFT), 12-step facilitation, and supportive counseling as well as medication treatments like Suboxone or Naltrexone. OR you can just describe the treatment. Is it outpatient? How long does it last? # of sessions/days/months?]*
2. Over the past 6 months, approximately how many youth received substance use treatment at your agency?
3. Approximately how many youth received A-CRA over the past 6 months?
4. Currently, how many clinicians at your agency treat youth with substance use problems?
5. Currently, how many of the clinicians at your agency have received an A-CRA certification? *[IF NEEDED, PROMPT: By certified, we mean they completed all training requirements – such as coaching and submission of recorded sessions – and received written documentation of certification. This includes first-level, full, transitional age youth, and supervisor certifications.]*
6. How many clinicians deliver A-CRA at your agency?
7. Next, I’d like to ask you about your agency’s plan to continue A-CRA clinical supervision support. Does your agency plan to maintain a Clinical Supervisor focused on A-CRA?

🞏 No – ***SKIP TO QUESTION 17***
🞏 Yes
🞏 Don’t Know

16A. Does your agency plan to support Clinical Supervisor and Counselor time for supervision?

🞏 No
🞏 Yes
🞏 Don’t Know

16B. Does your agency plan to support the Clinical Supervisor to listen to recorded therapy sessions and provide individualized feedback to counselors?

🞏 No
🞏 Yes
🞏 Don’t Know

1. Tell me about any ways in which your organization collaborates with other organizations to sustain A-CRA. *[IF NEEDED, PROMPT: For example, you may have engaged in training or support activities with your state substance use services authority and/or Chestnut Health Systems, or have partnered with universities or research institutions on research related to A-CRA].*
2. Tell me about any planning that has been done to ensure resources are available to continue A-CRA. By resources, we mean things needed to support A-CRA. For example, this could include things like money, staff, supervision, training, and A-CRA manuals. [*IF NEEDED, PROMPT: By strategic planning, we mean the process by which an organization defines its strategy or direction and makes decisions about how to allocate resources to pursue the strategy.]*
3. Can you please describe any policies that support A-CRA delivery? These could include external policies, such as funding source and billing rules that support A-CRA, as well as internal policies at your organization. *[IF NEEDED, PROMPT: By policies, we mean either organizational, state, national, or local policies. Here are some examples:*

*-State/county/health insurance co requires that we use an evidence-based treatment (EBT) and A-CRA is an EBT*

*-Our agency’s mission emphasizes involvement of the family, A-CRA does that]*

1. What about policies (external or internal) that interfere with A-CRA delivery? *[IF NEEDED, PROMPT:  By policies, we mean either organizational, state, national, or local policies. Here are some examples:*

*-if a state decided to require that substance use clinics deliver a different treatment (other than A-CRA), this policy might interfere with the delivery of A-CRA.*

*- if certain insurance plans stopped reimbursing for A-CRA or the full number of sessions, this policy would interfere with A-CRA delivery.]*

1. Does A-CRA meet the needs of the populations you serve? How so or why not?
2. Can you tell me about any pressure your organization experienced to continue delivering A-CRA or to discontinue its use? *[IF NEEDED, PROMPT: Pressure to deliver A-CRA could come from a variety of sources. For ex., organizations might experience pressure if there is some financial incentive to deliver A-CRA. If a state department of health decides its mission is to deliver evidence-based treatment for substance use, this could also be seen as pressure.]*
3. Would you say staff are supportive or reluctant to use A-CRA? Can you give me an example of what people have said or done to indicate their supportiveness or reluctance toward A-CRA?
4. Next, I would like to know what factors helped efforts to implement and sustain A-CRA in your organization. Please let me know if these factors were important in your organization, and if yes, how so. *[PROBE AS NEEDED: What factors were most helpful in that domain? Can you give me some examples? Were there important changes in which factors were most helpful once funding ended?]*

24A. Would you say characteristics of A-CRA helped efforts to implement and sustain A-CRA in your organization? Yes or No?

*[IF NEEDED: for example, treatment content, structure of sessions, etc.]*

🞏 No
🞏 Yes

IF YES, please describe: When was that factor most impactful? Was it during the CSAT grant funding period, after the CSAT funding ended, or throughout?

24B. Would you say characteristics of key individuals helped efforts to implement and sustain A-CRA in your organization? Yes or No?

*[IF NEEDED: “key individuals” are people who had a major positive impact on A-CRA implementation; could be within or outside of your treatment organization]*

🞏 No
🞏 Yes

IF YES, please describe: When was that factor most impactful? Was it during the CSAT grant funding period, after the CSAT funding ended, or throughout?

24C. Would you say client perspectives on A-CRA helped efforts to implement and sustain A-CRA in your organization? Yes or No?

*[IF NEEDED: for example, if clients found A-CRA acceptable, appropriate, feasible, etc.]*

🞏 No
🞏 Yes

IF YES, please describe: When was that factor most impactful? Was it during the CSAT grant funding period, after the CSAT funding ended, or throughout?

24D. Would you say helpful factors within your organization helped efforts to implement and sustain A-CRA in your organization? Yes or No?

*[IF NEEDED: for example, organizational leadership, staffing patterns, scheduling appointments, etc.]*

🞏 No
🞏 Yes

IF YES, please describe: When was that factor most impactful? Was it during the CSAT grant funding period, after the CSAT funding ended, or throughout?

24E. Would you say helpful factors outside your organization helped efforts to implement and sustain A-CRA in your organization? Yes or No?

*[IF NEEDED: for example, state leadership, federal support, community factors]*

🞏 No
🞏 Yes

IF YES, please describe: When was that factor most impactful? Was it during the CSAT grant funding period, after the CSAT funding ended, or throughout?

24F. Would you say partnerships with other organizations helped efforts to implement and sustain A-CRA in your organization? Yes or No?

🞏 No
🞏 Yes

IF YES, please describe: When was that factor most impactful? Was it during the CSAT grant funding period, after the CSAT funding ended, or throughout?

24G. Would you say funding helped efforts to implement and sustain A-CRA in your organization? Yes or No?

🞏 No
🞏 Yes

IF YES, please describe: When was that factor most impactful? Was it during the CSAT grant funding period, after the CSAT funding ended, or throughout?

24H. Were there other helpful factors during or after the grant period?

🞏 No
🞏 Yes

IF YES, please describe: When was that factor most impactful? Was it during the CSAT grant funding period, after the CSAT funding ended, or throughout?

1. Now, I’m going ask how the same six categories of factors may have included barriers that hindered efforts to implement and sustain A-CRA in your organization. Please let me know if these factors were important in your organization, and if yes, how so? *[PROBE AS NEEDED: What factors were the biggest barriers in that domain? Can you give me some examples? Were there important changes in which factors were the biggest barriers once funding ended?]*

25A. Would you say characteristics of A-CRA hindered efforts to implement and sustain A-CRA in your organization? Yes or No?

*[IF NEEDED: for example, treatment content, structure of sessions, etc.]*

🞏 No
🞏 Yes

IF YES, please describe: When was that factor most impactful? Was it during the CSAT grant funding period, after the CSAT funding ended, or throughout?

25B. Would you say challenges with key individuals hindered efforts to implement and sustain A-CRA in your organization? Yes or No?

*[IF NEEDED: “key individuals” are people who presented major challenges for A-CRA implementation; could be within or outside of your treatment organization]*

🞏 No
🞏 Yes

IF YES, please describe: When was that factor most impactful? Was it during the CSAT grant funding period, after the CSAT funding ended, or throughout?

25C. Would you say client perspectives on A-CRA hindered efforts to implement and sustain A-CRA in your organization? Yes or No?

*[IF NEEDED: for example, if clients found A-CRA acceptable, appropriate, feasible, etc.]*

🞏 No
🞏 Yes

IF YES, please describe: When was that factor most impactful? Was it during the CSAT grant funding period, after the CSAT funding ended, or throughout?

25D. Would you say challenges within your organization hindered efforts to implement and sustain A-CRA in your organization? Yes or No?

*[IF NEEDED: for example, organizational leadership, staffing patterns, scheduling appointments, etc.]*

🞏 No
🞏 Yes

IF YES, please describe: When was that factor most impactful? Was it during the CSAT grant funding period, after the CSAT funding ended, or throughout?

25E. Would you say challenges outside your organization hindered efforts to implement and sustain A-CRA in your organization? Yes or No?

*[IF NEEDED: for example, state leadership, federal support, community factors]*

🞏 No
🞏 Yes

IF YES, please describe: When was that factor most impactful? Was it during the CSAT grant funding period, after the CSAT funding ended, or throughout?

25F. Would you say issues in partnerships with other organizations hindered efforts to implement and sustain A-CRA in your organization? Yes or No?

🞏 No
🞏 Yes

IF YES, please describe: When was that factor most impactful? Was it during the CSAT grant funding period, after the CSAT funding ended, or throughout?

25G. Would you say funding hindered efforts to implement and sustain A-CRA in your organization? Yes or No?

🞏 No
🞏 Yes

IF YES, please describe: When was that factor most impactful? Was it during the CSAT grant funding period, after the CSAT funding ended, or throughout?

25H. Were there other barriers during or after the grant period?

🞏 No
🞏 Yes

IF YES, please describe: When was that factor most impactful? Was it during the CSAT grant funding period, after the CSAT funding ended, or throughout?

1. How many staff supervise clinicians who treat youth with substance use problems?
2. Do you supervise clinicians who deliver A-CRA?

🞏 No
🞏 Yes
🞏 Don’t Know

1. How many others at your agency supervise clinicians delivering A-CRA?
2. Have you personally certified any clinicians in A-CRA?

🞏 No – ***SKIP TO QUESTION 46***

🞏 Yes – ***CONTINUE***

*[IF NEEDED, PROMPT: By certified, we mean they completed all training requirements – such as coaching and submission of recorded sessions – and received written documentation of certification. Respond “yes” if you have personally certified anyone with the first-level, full, transitional age youth, and/or supervisor certifications.]*

1. You mentioned that you personally had certified one or more clinicians in A-CRA at your organization. Can you please describe the certification process to me?

The clinician was asked to:

🞏 Read the A-CRA manual

🞏 Take an online A-CRA research course

🞏 Pass an A-CRA quiz with a score of 80% or higher

🞏 Attend a Chestnut or Robert J. Meyers A-CRA initial training OR attend an in-house training

🞏 Participate in regular coaching calls with Chestnut or regular supervision with in-house Clinical Supervisor (respondent) during certification (regular= @ every other week)

🞏 Regularly record therapy sessions for in-house Clinical Supervisor (respondent) review/Chestnut’s review (regular= at least some sessions weekly))

🞏 Demonstrate competency in General Clinical Skills on the DSRs

🞏 Demonstrate competency in the following A-CRA procedures:

Functional Analysis of Use

Functional Analysis of Pro-social behavior

Happiness Scale

Treatment Plan/Goals of Counseling

Communication Skills

Problem Solving Skills

Adolescent-Caregiver Relationship Skills, and

Homework based of 3 or better on all components of a given procedure using the A-CRA rating manual?

If in-house training was provided, did it:

🞏 Include didactic information about A-CRA procedures?

🞏 Modeling or review of audio recordings of procedures that were well done

🞏 The opportunity to role play procedures

🞏 Other, please explain_____________________________________

**Next, I'm going to ask you a series of questions about how the certification process currently works in your agency. Please respond by saying “True," "False," or "don't know" if you are unsure.**

1. When I decide to pass a clinician on a procedure it is based on ratings of 1 or more on every component of a procedure. Remember that each component of a procedure is rated on a 1 to 5 scale.

🞏 False
🞏 True
🞏 Don’t Know

🞏 NOT APPLICABLE - DONE BY CHESTNUT HEALTH SYSTEMS

1. Communication skills is a procedure that people have to pass to attain certification.

🞏 False
🞏 True
🞏 Don’t Know

1. I review recorded sessions during the certification process.

🞏 False
🞏 True
🞏 Don’t Know

🞏 NOT APPLICABLE - DONE BY CHESTNUT HEALTH SYSTEMS

1. I am required to sit in sessions with clinicians during the certification process.

🞏 False
🞏 True
🞏 Don’t Know

1. Clinicians record one or two of their sessions.

🞏 False
🞏 True
🞏 Don’t Know

1. I refer to the A-CRA rating manual when rating session recordings.

🞏 False
🞏 True
🞏 Don’t Know

🞏 NOT APPLICABLE - DONE BY CHESTNUT HEALTH SYSTEMS

1. Clinicians are required to take a knowledge test as part of the certification process.

🞏 False
🞏 True
🞏 Don’t Know

1. Clinicians are not required to pass General Clinical Skills as part of the certification process.

🞏 False
🞏 True
🞏 Don’t Know

1. Each clinician has a certification workbook.

🞏 False
🞏 True
🞏 Don’t Know

1. I complete the A-CRA checklist when I am listening to a recorded session during or after the certification process.

🞏 False
🞏 True
🞏 Don’t Know

🞏 NOT APPLICABLE - DONE BY CHESTNUT HEALTH SYSTEMS

1. Clinicians are required to read the A-CRA manual during the training process.

🞏 False
🞏 True
🞏 Don’t Know

1. Time is set aside for training clinicians in A-CRA

🞏 False
🞏 True
🞏 Don’t Know

1. During training clinicians are required to practice procedures with role-plays.

🞏 False
🞏 True
🞏 Don’t Know

1. Adolescent-Caregiver Relationship Skills is one of the procedures for basic certification.

🞏 False
🞏 True
🞏 Don’t Know

1. It doesn't matter if clinicians show competency in all of the additional procedures as well.

🞏 False
🞏 True
🞏 Don’t Know

1. On average, approximately how many session recordings do clinicians at your site complete to reach first-level certification (pass first 9 A-CRA procedures)? Your best guess is fine.
2. Do you have a copy of the A-CRA manual? *[IF NEEDED, PROMPT: If you have access to a shared copy of the manual through your organization, electronically, etc. that counts as having a copy.]*

🞏 No – ***SKIP TO QUESTION 51***

🞏 Yes – ***CONTINUE***

1. In the past six-months, how often would you say you used the manual? *[IF NEEDED, PROMPT: By use, we mean a reference to the manual. It could mean a quick review of key session content in preparation for a session, or a thorough read-through in order to master the material.]*

🞏 Never

🞏 A few times per year, or less

🞏 About once a month

🞏 A few times per month

🞏 Weekly

🞏 Daily

1. In the past six-months, how often do you provide group clinical supervision? *[IF NEEDED, PROMPT:  This includes any supervision you provided; not supervision you received. Our focus is on A-CRA supervision but you can include any supervision where A-CRA would be discussed – it does not need to be exclusively supervision for A-CRA cases.]*

🞏 Never
🞏 Once a week
🞏 Every two weeks
🞏 Monthly
🞏 Other, please describe how often: __________________________________

1. In the past six-months, how often do you provide individual clinical supervision? *[IF NEEDED, PROMPT:  This includes any supervision you provided; not supervision you received. Our focus is on A-CRA supervision but you can include any supervision where A-CRA would be discussed – it does not need to be exclusively supervision for A-CRA cases.]*

🞏 Never
🞏 Once a week
🞏 Every two weeks
🞏 Monthly
🞏 Other, please describe how often: __________________________________

1. How many new clinicians have been trained in A-CRA in the past six-months in the organization?
2. How many new clinicians have been trained in A-CRA in the past six-months at a Chestnut or Robert J. Meyers training?

**[IF ANSWER TO QUESTION 51 > or = to ANSWER TO QUESTION 52 CONTINUE; OTHERWISE, SKIP TO QUESTION 55]**

1. Are there training agendas for your trainings?

🞏 No – ***SKIP TO QUESTION 55*** 🞏 Yes – ***CONTINUE***

**[IF QUESTION 53=Yes AND ANSWER TO QUESTION 51>0 AND ANSWER TO QUESTION 51 >= ANSWER TO QUESTION 52]**

1. Will you please send me (email, mail, fax) a copy of the agenda?

🞏 No
 🞏 Yes

**Currently, when you are introducing A-CRA to a new client…**

1. How many sessions do you tell the client receiving A-CRA they will have?
2. How many weeks do you tell the client receiving A-CRA the treatment will take?
3. How has the COVID-19 pandemic affected substance use treatment services at your organization?
4. How has it affected the ability of your organization to sustain A-CRA services?
5. What have been the most significant changes affecting your A-CRA program in response to COVID-19? *[PROBE AS NEEDED: Changes could include things like…*

*…Changes in referrals to A-CRA*

*…Changes in how A-CRA assessment, treatment, or case management services are delivered*

*…Changes in staffing*

*…Remote work*

*…Telehealth service delivery*

*Were there other changes? If so, please describe:]*

1. Have there been changes made in response to COVID-19 that have been beneficial, and will those changes continue beyond the pandemic? *[PROBE AS NEEDED: These may be some of the changes you already described, or may be different. Changes could include things like…*

*…Changes in referrals to A-CRA*

*…Changes in how A-CRA assessment, treatment, or case management services are delivered*

*…Changes in staffing*

*…Remote work*

*…Telehealth service delivery*

*Were there other beneficial changes? If so, please describe:]*

**OK, we are almost done. Next, I want to ask you about your general impressions regarding the SAMHSA CSAT-funded A-CRA project and then have a few questions about you.**

1. If you had a chance to participate in a SAMHSA CSAT project again, would you consider it?

🞏 No
🞏 Yes

1. Why/why not?
2. Is there anything you would change about the SAMHSA CSAT project in order to improve the sustainment of A-CRA at your agency?
3. Is there anything else you would like to share related to the topic of sustaining A-CRA at your agency?

[TURN OFF RECORDER]

That's all the interview questions I have. Thank you for providing this information.

[IF SENDING WEB SURVEY (FULL OR ABBREVIATED) AS USUAL]

We will soon be sending you the link to the web survey in order to provide a more complete picture of the treatment offered to youth at your organization and about organizational and clinical support. We will send it within a few days, and will send your $50 Amazon e-gift card as soon as the survey is complete.

[IF PARTICIPANT WILL BE INTERVIEWED AGAIN, complete the tracking module]

**Non-Sustainer Interview – SUPERVISOR**

1. Based on the background information we’ve received, it sounds like your agency currently does not deliver the Adolescent Community Reinforcement Approach or A-CRA anymore. Is this correct?

🞏 No – ***SWITCH TO SUSTAINER INTERVIEW*** 🞏 Yes – ***CONTINUE***

1. Tell me a little bit about the substance use services at your agency. What services are available? What types of clients do you serve? (e.g., age range, services for specific substances).
2. What youth age range do you use A-CRA with at your agency? Throughout this interview, by “youth” we typically mean ages 12 through 17 (or through age 24 if your A-CRA training included young adults).
3. What is your primary professional role(s) at your clinic/site?

🞏 Clinical Supervisor – ***CONTINUE***

🞏 Counselor or Clinician – ***SWITCH TO CLINICIAN ONLY VERSION***
🞏 Clinical Supervisor and Counselor/Clinician ***– SWITCH TO SUPERVISOR + CLINICIAN VERSION***

🞏 Other, please describe: ____________________ (e.g., administrator, grant manager) *–* ***CONTINUE***

1. Did you participate in any SAMHSA/CSAT-funded A-CRA training initiatives in your state? Yes or No? *[IF NEEDED, PROMPT AS RELEVANT: Our records do/do not indicate that you received training in A-CRA as part of a SAMHSA CSAT grant under the [initiative] in [grants years]. (IF KNOWN) In your state, I believe this initiative was called [NAME]. So would it be accurate to say you did/did not participate in your state’s A-CRA training initiative?]*

🞏 No – ***SKIP TO QUESTION 6*** 🞏 Yes – ***CONTINUE***

5A. Between what dates did you work on the project? Your best guess is fine.

1. What role(s) did you serve while A-CRA was being delivered at your agency? *[MARK ALL THAT APPLY]*

🞏 A-CRA Therapist
 🞏 A-CRA Supervisor
 🞏 Other (Please describe: ____________________________________________________)
 🞏 No Role in delivering ACRA

1. Have you received any A-CRA certifications? This means that you completed all training requirements (such as coaching and submission of recorded sessions) and received written documentation of the certification. [MARK ALL THAT APPLY]

🞏 First-level clinician certification (passed 9 A-CRA procedures)
 🞏 Full clinician certification (passed all 19 A-CRA procedures)
 🞏 Supervisor certification
 🞏 A-CRA-TAY (Transitional Age Youth) certification

🞏 No, never certified in A-CRA

*[IF NEEDED, PROMPT:  A-CRA supervisor certification is different from A-CRA certification. It refers to being certified to provide A-CRA clinical supervision].*

1. What do you think of A-CRA as a treatment for youth?
2. When did you/your agency stop delivering A-CRA? If you do not know the exact date, please give your best estimate.

Month/Year: ______ /______

1. What were the main reasons you/your agency stopped delivering A-CRA?
2. What would have increased your desire to continue delivering A-CRA?
3. What would have increased your ability to continue delivering A-CRA?
4. Does your program currently offer any different types of treatment for youth with substance use disorders besides A-CRA?

🞏 No – ***SKIP TO QUESTION 15***

🞏 Yes – ***CONTINUE***
🞏 Don’t Know – ***SKIP TO QUESTION 15***

1. What are those treatments called? *[IF NEEDED, PROMPT: Examples of other treatments include Motivational Enhancement Therapy (MET), Cognitive Behavioral Therapy (CBT), Multidimensional Family Therapy (MDFT), 12-step facilitation, and supportive counseling as well as medication treatments like Suboxone or Naltrexone. OR you can just describe the treatment. Is it outpatient? How long does it last? # of sessions/days/months?]*
2. Over the past 6 months, approximately how many youth received substance use treatment at your agency?
3. Approximately how many youth received A-CRA over the past 6 months?

16A. Approximately how many youth received A-CRA during the six-month period, right before A-CRA treatment delivery ended?

1. Currently, how many clinicians at your agency treat youth with substance use problems?
2. Currently, how many of the clinicians at your agency have received an A-CRA certification? *[IF NEEDED, PROMPT: By certified, we mean they completed all training requirements – such as coaching and submission of recorded sessions – and received written documentation of certification. This includes first-level, full, transitional age youth, and supervisor certifications.]*

18A. Approximately how many clinicians at your agency had received an A-CRA certification during the six-month period, right before A-CRA treatment delivery ended?

1. Next, I’d like to ask you about your agency’s A-CRA clinical supervision support during the last six months it was delivered. Was your agency able to maintain a Clinical Supervisor focused on A-CRA?

🞏 No – ***SKIP TO QUESTION 20***
🞏 Yes
🞏 Don’t Know

19A. Did your agency support Clinical Supervisor and Counselor time for supervision?

🞏 No
🞏 Yes
🞏 Don’t Know

19B. Did your agency support the Clinical Supervisor to listen to recorded therapy sessions and provide individualized feedback to counselors?

🞏 No
🞏 Yes
🞏 Don’t Know

1. Tell me about any ways in which your organization collaborates with other organizations – especially anything related to A-CRA. *[IF NEEDED, PROMPT: For example, you may have engaged in training or support activities with your state substance use services authority and/or Chestnut Health Systems, or have partnered with universities or research institutions on research related to A-CRA].*
2. Was there any planning done to ensure resources were available to continue A-CRA beyond the initial funding period? By resources, we mean things needed to support A-CRA. For example, this could include things like money, staff, supervision, training, and A-CRA manuals. [*IF NEEDED, PROMPT: By strategic planning, we mean the process by which an organization defines its strategy or direction and makes decisions about how to allocate resources to pursue the strategy.]*
3. Can you please describe any policies that supported A-CRA delivery? These could include external policies, such as funding source and billing rules that support A-CRA, as well as internal policies at your organization. *[IF NEEDED, PROMPT: By policies, we mean either organizational, state, national, or local policies. Here are some examples:*

*-State/county/health insurance co requires that we use an evidence-based treatment (EBT) and A-CRA is an EBT*

*-Our agency’s mission emphasizes the involvement of the family, A-CRA does that]*

1. What about policies (external or internal) that interfered with A-CRA delivery? *[IF NEEDED, PROMPT:  By policies, we mean either organizational, state, national, or local policies. Here are some examples:*

*-if a state decided to require that substance use clinics deliver a different treatment (other than A-CRA), this policy might interfere with the delivery of A-CRA.*

*- if certain insurance plans stopped reimbursing for A-CRA or the full number of sessions, this policy would interfere with A-CRA delivery.]*

1. Did A-CRA meet the needs of the populations you serve? How so or why not?
2. Can you tell me about any pressure your organization experienced to continue delivering A-CRA or to discontinue its use? *[IF NEEDED, PROMPT:  Pressure to deliver A-CRA could come from a variety of sources. For example, organizations might experience pressure if there is some financial incentive to deliver A-CRA. If a state department of health decides its mission is to deliver evidence-based treatment for substance use, this could also be seen as pressure.]*
3. Would you say staff were supportive or reluctant to use A-CRA? Can you give me an example of what people have said or done to indicate their supportiveness or reluctance toward A-CRA?
4. Next, I would like to know what factors helped efforts to implement and sustain A-CRA in your organization. Please let me know if these factors were important in your organization, and if yes, how so. *[PROBE AS NEEDED: What factors were most helpful in that domain? Can you give me some examples? Were there important changes in which factors were most helpful once funding ended?]*

27A. Would you say characteristics of A-CRA helped efforts to implement and sustain A-CRA in your organization? Yes or No?

*[IF NEEDED: for example, treatment content, structure of sessions, etc.]*

🞏 No
🞏 Yes

IF YES, please describe: When was that factor most impactful? Was it during the CSAT grant funding period, after the CSAT funding ended, or throughout?

27B. Would you say characteristics of key individuals helped efforts to implement and sustain A-CRA in your organization? Yes or No?

*[IF NEEDED: “key individuals” are people who had a major positive impact on A-CRA implementation; could be within or outside of your treatment organization]*

🞏 No
🞏 Yes

IF YES, please describe: When was that factor most impactful? Was it during the CSAT grant funding period, after the CSAT funding ended, or throughout?

27C. Would you say client perspectives on A-CRA helped efforts to implement and sustain A-CRA in your organization? Yes or No?

*[IF NEEDED: for example, if clients found A-CRA acceptable, appropriate, feasible, etc.]*

🞏 No
🞏 Yes

IF YES, please describe: When was that factor most impactful? Was it during the CSAT grant funding period, after the CSAT funding ended, or throughout?

27D. Would you say helpful factors within your organization helped efforts to implement and sustain A-CRA in your organization? Yes or No?

*[IF NEEDED: for example, organizational leadership, staffing patterns, scheduling appointments, etc.]*

🞏 No
🞏 Yes

IF YES, please describe: When was that factor most impactful? Was it during the CSAT grant funding period, after the CSAT funding ended, or throughout?

27E. Would you say helpful factors outside your organization helped efforts to implement and sustain A-CRA in your organization? Yes or No?

*[IF NEEDED: for example, state leadership, federal support, community factors]*

🞏 No
🞏 Yes

IF YES, please describe: When was that factor most impactful? Was it during the CSAT grant funding period, after the CSAT funding ended, or throughout?

27F. Would you say partnerships with other organizations helped efforts to implement and sustain A-CRA in your organization? Yes or No?

🞏 No
🞏 Yes

IF YES, please describe: When was that factor most impactful? Was it during the CSAT grant funding period, after the CSAT funding ended, or throughout?

27G. Would you say funding helped efforts to implement and sustain A-CRA in your organization? Yes or No?

🞏 No
🞏 Yes

IF YES, please describe: When was that factor most impactful? Was it during the CSAT grant funding period, after the CSAT funding ended, or throughout?

27H. Were there other helpful factors during or after the grant period?

🞏 No
🞏 Yes

IF YES, please describe: When was that factor most impactful? Was it during the CSAT grant funding period, after the CSAT funding ended, or throughout?

1. Now, I’m going ask how the same six categories of factors may have included barriers that hindered efforts to implement and sustain A-CRA in your organization. Please let me know if these factors were important in your organization, and if yes, how so. *[PROBE AS NEEDED: What factors were the biggest barriers in that domain? Can you give me some examples? Were there important changes in which factors were the biggest barriers once funding ended?]*

28A. Would you say characteristics of A-CRA hindered efforts to implement and sustain A-CRA in your organization? Yes or No?

*[IF NEEDED: for example, treatment content, structure of sessions, etc.]*

🞏 No
🞏 Yes

IF YES, please describe: When was that factor most impactful? Was it during the CSAT grant funding period, after the CSAT funding ended, or throughout?

28B. Would you say challenges with key individuals hindered efforts to implement and sustain A-CRA in your organization? Yes or No?

*[IF NEEDED: “key individuals” are people who presented major challenges for A-CRA implementation; could be within or outside of your treatment organization]*

🞏 No
🞏 Yes

IF YES, please describe: When was that factor most impactful? Was it during the CSAT grant funding period, after the CSAT funding ended, or throughout?

28C. Would you say client perspectives on A-CRA hindered efforts to implement and sustain A-CRA in your organization? Yes or No?

*[IF NEEDED: for example, if clients found A-CRA acceptable, appropriate, feasible, etc.]*

🞏 No
🞏 Yes

IF YES, please describe: When was that factor most impactful? Was it during the CSAT grant funding period, after the CSAT funding ended, or throughout?

28D. Would you say challenges within your organization hindered efforts to implement and sustain A-CRA in your organization? Yes or No?

*[IF NEEDED: for example, organizational leadership, staffing patterns, scheduling appointments, etc.]*

🞏 No
🞏 Yes

IF YES, please describe: When was that factor most impactful? Was it during the CSAT grant funding period, after the CSAT funding ended, or throughout?

28E. Would you say challenges outside your organization hindered efforts to implement and sustain A-CRA in your organization? Yes or No?

*[IF NEEDED: for example, state leadership, federal support, community factors]*

🞏 No
🞏 Yes

IF YES, please describe: When was that factor most impactful? Was it during the CSAT grant funding period, after the CSAT funding ended, or throughout?

28F. Would you say issues in partnerships with other organizations hindered efforts to implement and sustain A-CRA in your organization? Yes or No?

🞏 No
🞏 Yes

IF YES, please describe: When was that factor most impactful? Was it during the CSAT grant funding period, after the CSAT funding ended, or throughout?

28G. Would you say funding hindered efforts to implement and sustain A-CRA in your organization? Yes or No?

🞏 No
🞏 Yes

IF YES, please describe: When was that factor most impactful? Was it during the CSAT grant funding period, after the CSAT funding ended, or throughout?

28H. Were there other barriers during or after the grant period?

🞏 No
🞏 Yes

IF YES, please describe: When was that factor most impactful? Was it during the CSAT grant funding period, after the CSAT funding ended, or throughout?

1. How many staff supervise clinicians who treat youth with substance use problems?
2. Did you supervise clinicians delivering A-CRA at your agency?

🞏 No
🞏 Yes
🞏 Don’t Know

1. How many others at your agency supervised clinicians delivering A-CRA?
2. Have you personally certified any clinicians in A-CRA?

🞏 No – ***SKIP TO QUESTION 49***🞏 Yes – ***CONTINUE***

*[IF NEEDED, PROMPT: By certified, we mean they completed all training requirements – such as coaching and submission of recorded sessions – and received written documentation of certification. Respond “yes” if you have personally certified anyone with the first-level, full, transitional age youth, and/or supervisor certifications.]*

1. You mentioned that you personally had certified one or more clinicians in A-CRA at your organization. Can you please describe the certification process to me?

The clinician was asked to:

🞏 Read the A-CRA manual

🞏 Take an online A-CRA research course

🞏 Pass an A-CRA quiz with a score of 80% or higher

🞏 Attend a Chestnut or Robert J. Meyers A-CRA initial training OR attend an in-house training

🞏 Participate in regular coaching calls with Chestnut or regular supervision with in-house Clinical Supervisor (respondent) during certification (regular= @ every other week)

🞏 Regularly record therapy sessions for in-house Clinical Supervisor (respondent) review/Chestnut’s review (regular= at least some sessions weekly))

🞏 Demonstrate competency in General Clinical Skills on the DSRs

🞏 Demonstrate competency in the following A-CRA procedures:

Functional Analysis of Use

Functional Analysis of Pro-social behavior

Happiness Scale

Treatment Plan/Goals of Counseling

Communication Skills

Problem Solving Skills

Adolescent-Caregiver Relationship Skills, and

Homework based of 3 or better on all components of a given procedure using the A-CRA rating manual?

If in-house training was provided, did it:

🞏 Include didactic information about A-CRA procedures?

🞏 Modeling or review of audio recordings of procedures that were well done

🞏 The opportunity to role play procedures

🞏 Other, please explain_____________________________________

**Next, I'm going to ask you a series of questions about how the certification process worked in your agency. Please respond by saying “True," "False," or "don't know" if you are unsure.**

1. When I decided to pass a clinician on a procedure it was based on ratings of 1 or more on every component of a procedure. Remember that each component of a procedure is rated on a 1 to 5 scale.

🞏 False
 🞏 True
 🞏 Don’t Know

🞏 NOT APPLICABLE - DONE BY CHESTNUT HEALTH SYSTEMS

1. Communication skills was a procedure that people had to pass to attain certification.

🞏 False
🞏 True
🞏 Don’t Know

1. I reviewed recorded sessions during the certification process.

🞏 False
 🞏 True
 🞏 Don’t Know

🞏 NOT APPLICABLE - DONE BY CHESTNUT HEALTH SYSTEMS

1. I was required to sit in on sessions with clinicians during the certification process.

🞏 False
🞏 True
🞏 Don’t Know

1. Clinicians recorded one or two of their sessions.

🞏 False
🞏 True
🞏 Don’t Know

1. I referred to the A-CRA rating manual when rating session recordings.

🞏 False
 🞏 True
 🞏 Don’t Know

🞏 NOT APPLICABLE - DONE BY CHESTNUT HEALTH SYSTEMS

1. Clinicians were required to take a knowledge test as part of the certification process.

🞏 False
🞏 True
🞏 Don’t Know

1. Clinicians were not required to pass General Clinical Skills as part of the certification process.

🞏 False
🞏 True
🞏 Don’t Know

1. Each clinician had a certification workbook.

🞏 False
🞏 True
🞏 Don’t Know

1. I completed the A-CRA checklist when I was listening to a recorded session during or after the certification process.

🞏 False
 🞏 True
 🞏 Don’t Know

🞏 NOT APPLICABLE - DONE BY CHESTNUT HEALTH SYSTEMS

1. Clinicians were required to read the A-CRA manual during the training process.

🞏 False
🞏 True
🞏 Don’t Know

1. Time was set aside for training clinicians in A-CRA

🞏 False
🞏 True
🞏 Don’t Know

1. During training clinicians were required to practice procedures with role-plays.

🞏 False
🞏 True
🞏 Don’t Know

1. Adolescent-Caregiver Relationship Skills was one of the procedures for basic certification.

🞏 False
🞏 True
🞏 Don’t Know

1. It didn’t matter if clinicians showed competency in all of the additional procedures as well.

🞏 False
🞏 True
🞏 Don’t Know

1. On average, approximately how many session recordings did clinicians at your site complete to reach first-level certification (pass first 9 A-CRA procedures)? Your best guess is fine.
2. Have you ever had a copy of the A-CRA manual? *[IF NEEDED, PROMPT: If you have access to a shared copy of the manual through your organization, electronically, etc. that counts as having a copy.]*

🞏 No – ***SKIP TO QUESTION 56***

🞏 Yes – ***CONTINUE***

1. Please think about the six-month period, right before A-CRA treatment delivery ended. How often did you use the manual? *[IF NEEDED, PROMPT: By use, we mean a reference to the manual. It could mean a quick review of key session content in preparation for a session, or a thorough read-through in order to master the material.]*

🞏 Never
🞏 A few times per year, or less
🞏 About once a month
🞏 A few times per month
🞏 Weekly
🞏 Daily

1. Do you still use your manual? *[IF NEEDED, PROMPT: By use, we mean a reference to the manual. It could mean a quick review of key session content in preparation for a session, or a thorough read-through in order to master the material.]*

🞏 No – ***SKIP TO QUESTION 56***

🞏 Yes – ***CONTINUE***

1. How often do you use your manual now? *[IF NEEDED, PROMPT: By use, we mean a reference to the manual. It could mean a quick review of key session content in preparation for a session, or a thorough read-through in order to master the material.]*

🞏 Never
 🞏 A few times per year, or less
 🞏 About once a month
 🞏 A few times per month
 🞏 Weekly
 🞏 Daily

**For the next few questions, please think about the six-month period, right before A-CRA treatment delivery ended.**

1. How often do you provide group clinical supervision? *[IF NEEDED, PROMPT:  This includes any supervision you provided; not supervision you received. Our focus is on A-CRA supervision but you can include any supervision where A-CRA would be discussed – it does not need to be exclusively supervision for A-CRA cases.]*

🞏 Never
🞏 Once a week
🞏 Every two weeks
🞏 Monthly
🞏 Other, please describe how often: _______________________________

1. How often do you provide individual clinical supervision? *[IF NEEDED, PROMPT:  This includes any supervision you provided; not supervision you received. Our focus is on A-CRA supervision but you can include any supervision where A-CRA would be discussed – it does not need to be exclusively supervision for A-CRA cases.]*

🞏 Never
🞏 Once a week
🞏 Every two weeks
🞏 Monthly
🞏 Other, please describe how often: _______________________________

1. During the six-month period, right before A-CRA treatment delivery ended, how many sessions did you tell the client receiving A-CRA they would have?
2. During the six-month period, right before A-CRA treatment delivery ended, how many weeks did you tell the client receiving A-CRA the treatment would take?

**Now I’d like to ask you the same questions, but now please think about the current practices in the adolescent treatment program.**

1. How often do you provide group clinical supervision? *[IF NEEDED, PROMPT: This includes any supervision you provided; not supervision you received. Our focus is on A-CRA supervision but you can include any supervision where A-CRA would be discussed – it does not need to be exclusively supervision for A-CRA cases.]*

🞏 Never
🞏 Once a week
🞏 Every two weeks
🞏 Monthly
🞏 Other, please describe how often: _______________________________

1. How often do you provide individual clinical supervision? *[IF NEEDED, PROMPT:  This includes any supervision you provided; not supervision you received. Our focus is on A-CRA supervision but you can include any supervision where A-CRA would be discussed – it does not need to be exclusively supervision for A-CRA cases.]*

🞏 Never
🞏 Once a week
🞏 Every two weeks
🞏 Monthly
🞏 Other, please describe how often: _______________________________

1. How has the COVID-19 pandemic affected substance use treatment services at your organization?
2. When the pandemic began in March 2020, was your organization still delivering A-CRA?

🞏 No – ***SKIP TO QUESTION 64***🞏 Yes – ***CONTINUE***

🞏 Don’t know – ***SKIP TO QUESTION 64***

1. What have been the most significant changes affecting your A-CRA program in response to COVID-19? *[PROBE AS NEEDED: Changes could include things like…*

*…A-CRA was discontinued due to COVID-related changes*

*…Changes in referrals to A-CRA*

*…Changes in how A-CRA assessment, treatment, or case management services are delivered*

*…Changes in staffing*

*…Remote work*

*…Telehealth service delivery*

*Were there other changes? If so, please describe:]*

1. Have there been changes made in response to COVID-19 that have been beneficial, and will those changes continue beyond the pandemic? *[PROBE AS NEEDED: These may be some of the changes you already described, or may be different. Changes could include things like…*

*…Changes in referrals to A-CRA*

*…Changes in how A-CRA assessment, treatment, or case management services are delivered*

*…Changes in staffing*

*…Remote work*

*…Telehealth service delivery*

*Were there other beneficial changes? If so, please describe:]*

**OK, we are almost done. Next, I want to ask you about your general impressions regarding the SAMHSA CSAT-funded A-CRA project and then have a few questions about you.**

1. If you had a chance to participate in a SAMHSA CSAT project again, would you consider it?

🞏 No
 🞏 Yes

1. Why/why not?
2. Is there anything you would change about the SAMHSA CSAT project in order to improve the sustainment of A-CRA at your agency?
3. Is there anything else you would like to share related to the topic of sustaining A-CRA at your agency?

[TURN OFF RECORDER]

That's all the interview questions I have. Thank you for providing this information.

[IF SENDING WEB SURVEY (FULL OR ABBREVIATED) AS USUAL]

We will soon be sending you the link to the web survey in order to provide a more complete picture of the treatment offered to youth at your organization and about organizational and clinical support. We will send it within a few days, and will send your $50 Amazon e-gift card as soon as the survey is complete.

[IF PARTICIPANT WILL BE INTERVIEWED AGAIN, complete the tracking module]

**A-CRA Financing Project Tracking Module**

Now I need to get some information to help us contact you next year. As I mentioned at the beginning, we would like to interview you every year for up to 4 years. This information, like your responses to all questions in the interview, is completely confidential. It will only be used to contact you about the project; your identity will not be linked to your interview or survey responses in any way.

IF NECESSARY: *You are very important to this study. Your experiences over the years will help us to understand how to better sustain evidence-based treatments, like A-CRA and to ultimately improve the quality of care for youth with substance use problems. When we contact you, you can decide if you want to participate in the next interview and survey.*

Q1. Just in case we are unable to reach you next year, can you confirm your work e-mail address and phone number?

Work Email:

Work Phone:

Q2. How about a personal e-mail address or cell phone number? We will only use your personal email or cell phone number if we are having trouble reaching you at your work e-mail address/phone.

Personal/Alternate Email:

Personal/Alternate Cell Phone:

Q3. Is there anyone we can contact if that may help us reach you? This could be a co-worker, a family member, a friend – whoever you think is best. What is their name and contact information? We will only contact them if we are having trouble reaching you.

Contact Name:

Contact relationship to you:

Contact Phone:

Contact Email:

Q4. Is there anything else we should keep in mind when contacting you for future interviews?

Again, thank you for your participation in this important study.

Q5. INTERVIEW NOTES FIELD:

# ORGANIZATION-FOCUSED

# A-CRA Financing Project: CLINICIAN INTERVIEW COVER SHEET

**Any Interview Notes:**

**RESPONDENT NAME: ______________________________________________________**

**SITE NAME: _______________________________________________________________**

**Phone #: ___________________________________________**

**CHESTNUT YOUTHTX.ORG WEBSITE LOG IN NAME: _____________________________**

**NOTE: Should be first name initial, last name (ex: cpham)**

**CHESTNUT YOUTHTX.ORG WEBSITE PASSCODE: ________________________________**

**DATE OF INTERVIEW (MM/DD/YY): ______________________**

**TIME OF INTERVIEW (INTERVIEWEE TIME):_______________________________**

**FUNDING NAME DATES OF AGE RANGE OF TIME SINCE**

**FUNDING POPULATION LOSS FUND**

**SERVED**

|  | **START:**   \|  \| \| --- \|   **END:**   \|  \| \| --- \| |  | \|  \| **DAYS** \| \| --- \| --- \| \|  \| **MONTHS** \| \|  \| **YEARS** \| |
| --- | --- | --- | --- | --- | --- | --- | --- | --- | --- | --- | --- |
|  | **START:**   \|  \| \| --- \|   **END:**   \|  \| \| --- \| |  | \|  \| **DAYS** \| \| --- \| --- \| \|  \| **MONTHS** \| \|  \| **YEARS** \| |
|  | **START:**   \|  \| \| --- \|   **END:**   \|  \| \| --- \| |  | \|  \| **DAYS** \| \| --- \| --- \| \|  \| **MONTHS** \| \|  \| **YEARS** \| |
|  | **START:**   \|  \| \| --- \|   **END:**   \|  \| \| --- \| |  | \|  \| **DAYS** \| \| --- \| --- \| \|  \| **MONTHS** \| \|  \| **YEARS** \| |

***CLINICIAN INTERVIEW VERBAL CONSENT***

(READ PRIOR TO BEGINNING INTERVIEW/RECORDING)

Hi [CLINICIAN NAME],

This is [INTERVIEWER NAME] calling from RAND to speak with you about the youth substance use treatment program and any experience you may have in implementing the Adolescent Community Reinforcement Approach (A-CRA). Is this still a good time to talk?

>IF NO, reschedule.

>IF YES, great.

Before we begin, let me assure you that your responses to these questions will be held in strict confidence. We are requesting interviews with staff at over 75 programs that were funded by the Centers of Substance Abuse Treatment to deliver the Adolescent Community Reinforcement Approach and we will aggregate information from the interviews to characterize programs that were funded over time. We will not attribute comments to specific individuals or programs in any of our reports or publications. Your participation will help us better understand how evidence-based treatments can be sustained in community practice settings after the initial support for delivery ends.

We would like to record the interview today to ensure that we capture everything that is said. We will destroy the recording as soon as we determine that we have captured everything in our notes.

After the interview, we will ask you to complete a 15–30-minute online survey to get a more complete picture. Upon receipt of the web survey, we will send you an honorarium check of $50. In addition, we are asking clinicians to upload audio recordings of three therapy sessions. After our discussion today, we will send you information and instructions for uploading the recordings. If you do upload three recordings, you will receive an additional honorarium check of $50, for a total of $100. ***These recordings should be of NEWLY recorded sessions, not previously recorded sessions.***

Your participation in this study is entirely voluntary. We would like to have your responses to all of the questions. However, if you’re uncomfortable with any question we can skip it. There are no right or wrong answers. We estimate that the interview will take about 45-60 minutes, depending on your answer to the questions.

- **Do you have any questions?**
- **Are you willing to take part in this discussion?**
- **Is it ok with you if we audio-record this discussion?**

**Sustainer Interview – CLINICIAN**

1. Based on an initial call, it sounded like your agency currently delivers the Adolescent Community Reinforcement Approach or A-CRA. Is this correct?

🞏 No – ***SWITCH TO NON-SUSTAINER INTERVIEW***🞏 Yes – ***CONTINUE***

1. Tell me a little bit about your agency.
2. What types of clients do you serve? (e.g., age range, gender, problem types).
3. What is your primary professional role(s) at your clinic/site?

🞏 Supervisor – ***SWITCH TO SUPERVISOR VERSION***

🞏 Counselor or Clinician – ***CONTINUE***
🞏 Supervisor and Counselor/Clinician ***– SWITCH TO SUP/CLIN VERSION***

1. Did you work on any of the following SAMHSA/CSAT A-CRA funded projects that your agency was awarded from the following dates?

|  | \| **START:** \|  \| \| --- \| --- \| \|  \|  \| \| **END:** \|  \| |
| --- | --- | --- | --- | --- | --- | --- | --- |
|  | \| **START:** \|  \| \| --- \| --- \| \|  \|  \| \| **END:** \|  \| |
|  | \| **START:** \|  \| \| --- \| --- \| \|  \|  \| \| **END:** \|  \| |
|  | \| **START:** \|  \| \| --- \| --- \| \|  \|  \| \| **END:** \|  \| |

🞏 No **– *SKIP TO QUESTION 7***🞏 Yes **– *CONTINUE***

1. Between what dates did you work on the project? Your best guess is fine.

Initiative Start Date End Date

|  | \| Month/Year: \|  \| \| --- \| --- \| \|  \|  \| | \| Month/Year: \|  \| \| --- \| --- \| |
| --- | --- | --- | --- | --- | --- | --- | --- | --- |
|  | \| Month/Year: \|  \| \| --- \| --- \| \|  \|  \| | \| Month/Year: \|  \| \| --- \| --- \| |
|  | \| Month/Year: \|  \| \| --- \| --- \| \|  \|  \| | \| Month/Year: \|  \| \| --- \| --- \| |
|  | \| Month/Year: \|  \| \| --- \| --- \| \|  \|  \| | \| Month/Year: \|  \| \| --- \| --- \| |

1. What role(s) have you served in delivering A-CRA? *[MARK ALL THAT APPLY]*

🞏 A-CRA Therapist
🞏 A-CRA Supervisor
🞏 Other (Please describe: ____________________________________________________)
🞏 No Role in delivering ACRA

1. What do you think of A-CRA as a treatment for youth? Please note that throughout this interview, by “youth” we mean the population served during your SAMHSA CSAT grant periods.
2. Does your program currently offer any different types of treatment for youth with substance use disorders besides A-CRA?

🞏 No – ***SKIP TO QUESTION 11***🞏 Yes – ***CONTINUE***🞏 Don’t Know – ***SKIP TO QUESTION 11***

1. What are those treatments called? [Or describe the treatment. Is it outpatient? How long does it last? # of sessions/days/months] *[MARK ALL THAT APPLY]*
   1. Motivational Enhancement Therapy (MET)
   2. Cognitive Behavioral Therapy (CBT)
   3. MET/CBT-5
   4. Matrix Model
   5. Multidimensional Family Therapy (MDFT)
   6. Multisystemic Therapy (MST)
   7. 12-step facilitation
   8. Psychoeducation
   9. Supportive counseling
   10. Other____________________________________________________________

**Treatment programs may have an easier time implementing a new treatment if there are a number of elements in place to support them. For this next section, we want to ask you about things that could affect A-CRA delivery at your organization.**

1. First, we want to ask you about planning. Tell me about any planning that has been done to ensure resources are available to continue A-CRA. By resources, we mean things needed to support A-CRA. For example, this could include things like money, staff, supervision, training, and A-CRA manuals.
2. Can you please describe any policies that support A-CRA delivery? These could include external policies, such as funding source and billing rules that support A-CRA.
3. What about policies that interfere with A-CRA delivery?
4. Does A-CRA meet the needs of the populations you serve? How so or why not?
5. Can you tell me about any pressure your organization experienced to continue delivering A-CRA or to discontinue its use?
6. Please describe how leaders at your organization support your use of A-CRA.
7. Would you say staff are supportive or reluctant to use A-CRA? Can you give me an example?
8. What do you think is the most important influence on A-CRA’s success at your organization?
9. In the past 6 months, how have you or others at your agency disseminated information about your A-CRA project? By disseminated, we mean things like presentations, writing articles, media coverage, teaching, blogging, tweeting, etc.
   1. Presented to a local or state-level professional audience.
   2. Presented to a national or international professional audience.
   3. Wrote article(s) that was published or accepted for publication in a peer reviewed journal.
   4. Wrote article(s) that is under review or revision for a peer reviewed journal.
   5. Wrote article(s) for a newsletter or brochure or website.
   6. Had article(s) published in a local or state newspaper or on the internet.
   7. Received local or regional media coverage -- including print, television, radio broadcast, internet.
   8. Received national media coverage -- including print (NY Times, USA Today, Time, Newsweek, etc.), television or radio or internet
   9. Used information or materials from the project in teaching a college-level course.
   10. Developed a "how-to" manual or training package.
   11. Provided technical assistance to other agencies trying to implement similar projects.
   12. Wrote a blog about the project.
   13. Presented project on a website.
   14. Tweeted about the Project.
   15. None of the above.
   16. Other (please specify).
10. **[ONLY ASK FOR THOSE WHO FUNDING HAS ENDED]** What helped your efforts to sustain your project after the CSAT funding ended?
    1. Active support from your agency's executives or administrators.
    2. Actions by your agency's Board members.
    3. Program has become essential to carrying out the mission of your agency.
    4. Actions of an internal "champion" or key leader.
    5. Agency has existing "capacity" (e.g. enough staff member, skills, resources) to continue the program.
    6. You were able to modify the program to fit within your other programming.
    7. It is low cost - did not need substantial resources to continue.
    8. You have internal support for writing new grant proposals that helped obtain new resources.
    9. You had outcome/evaluation data that helped convince potential funders of the value of this program.
    10. Current or previous clients served as advocates for the program.
    11. Your staff members believed in the program so much they would not let it die.
    12. Partnerships with other organizations helped you find new funding sources.
    13. Your grant partners provide in-kind or other resources for continuation.
    14. Technical assistance or guidance from an external agency.
    15. Other helpful influence (please specify):
11. **[ONLY ASK FOR THOSE WHO FUNDING HAS ENDED]** What barriers have you encountered in attempting to sustain the project activities or services after the end of its CSAT funding?
    1. Obtaining funding from external sources.
    2. Obtaining funding from agency's budget, or other internal sources.
    3. Obtaining support from agency administrators for continuing project activities.
    4. Project leader or other key project "champion" left the agency.
    5. Turnover among staff members delivering project services.
    6. Agency priorities changed; ACRA approach is no longer a priority.
    7. Agency priorities changed; serving youth is no longer a priority.
    8. Maintaining agreement among partners essential for continuing project activities.
    9. Lack of physical space, such as lease terminated, project offices used for a different purpose, etc.
    10. Equipment or facilities needed (such as computers) no longer available.
    11. Policy changes that were agreed on temporarily during project were not made permanent.
    12. Partner agencies did not do what they promised.
    13. Other barriers (please specify):
12. On average, approximately how many session recordings do clinicians at your site complete to reach basic certification? Your best guess is fine.
13. Do you have a copy of the A-CRA manual?

🞏 No – ***SKIP TO QUESTION 25***

🞏 Yes – ***CONTINUE***

1. In the past six-months, how often would you say you used the manual?

🞏 Never
🞏 A few times per year, or less
🞏 About once a month
🞏 A few times per month
🞏 Weekly
🞏 Daily

1. In the past six-months, how often do you provide group clinical supervision?

🞏 Never
🞏 Once a week
🞏 Every two weeks
🞏 Monthly
🞏 Other, please describe how often: ___________________________________

1. Thinking about the past six-months, please describe a typical group clinical supervision session.
2. In the past six-months, how often do you have individual clinical supervision?

🞏 Never
🞏 Once a week
🞏 Every two weeks
🞏 Monthly
🞏 Other, please describe how often: ___________________________________

1. Thinking about the past six-months, please describe a typical individual clinical supervision session. What topics do you usually discuss?
2. Thinking about all the supervision you have received over the past six-months, what does your supervisor do, and what are you expected to do?

**Thinking about the past six-months, which of the following happens during supervision sessions?**

1. My supervisor asks how my week has gone.

🞏 No
🞏 Yes

1. My supervisor discusses agency paperwork requirements.

🞏 No
🞏 Yes

1. My supervisor reviews my A-CRA case review report.

🞏 No
🞏 Yes

1. My supervisor asks me if I have any problem cases.

🞏 No
🞏 Yes

1. My supervisor reviews a recorded session with me and tells me what I have done well.

🞏 No
🞏 Yes

1. My supervisor reviews a recorded session with me and gives me suggestions about how I can improve my treatment delivery

🞏 No
🞏 Yes

1. My supervisor observes a live session and tells me what I have done well.

🞏 No
🞏 Yes

1. My supervisor observes a live session and gives me suggestions about how I can improve my treatment delivery

🞏 No
🞏 Yes

1. My supervisor asks me about my personal problems.

🞏 No
🞏 Yes

1. My supervisor role plays with me the correct way to do a procedure.

🞏 No
🞏 Yes

1. Is there anything else you want to share about a typical supervision session over the past six-months?

**Now, I’d like to ask you about trainings on treatment of use substance use.**

1. How many other trainings on youth substance use treatment have you participated in since you completed the A-CRA training?
2. What were the main topics of these trainings?
3. Do you prefer using any of the skills/tools/procedures you learned instead of A-CRA?

🞏 No
🞏 Yes

1. Why/why not?

**Currently, when you are introducing A-CRA to a new client…**

1. How many sessions do you tell the client receiving A-CRA he or she will have?

🞏 Less than 12
🞏 12 or more

1. How many weeks do you tell the client receiving A-CRA the treatment will take?

🞏 Less than 12
🞏 12 or more

**Next, I want to ask you about your general impressions regarding the SAMHSA CSAT-funded A-CRA project and then have a few questions about you.**

1. If you had a chance to participate in a SAMHSA CSAT project again, would you consider it?

🞏 No
🞏 Yes

1. Why/why not?
2. Is there anything you would change about the SAMHSA CSAT project in order to improve the sustainment of A-CRA at your agency?
3. Is there anything else you would like to share related to the topic of sustaining A-CRA at your agency?
4. How many years of experience do you have in substance use counseling as a clinician?

# of years ____________ (OR) # of months ___________

1. Please provide the full name (as opposed to only the acronym) of all Licensures and/or Certifications you currently hold.

**END SCRIPT:**

We are now done with the interview. Here’s what happens next:

- In about a week, you will receive an e-mail from RAND with the link to a web survey and it will include your personal password. You can do it any date/time convenient for you, but please try to complete it within 2-3 weeks. At the last screen of the web survey, we will ask you for your contact information for your $50 honorarium check. We will only use that to send your check and it will not be attached to your survey.
- You will also receive a separate email with instructions on how to submit your 3 sample therapy recordings, in which you will receive another $50 and should try to do within the next month. The email will have detailed instructions and a consent form that needs to be completed for each client. ***These recordings should be of NEWLY recorded sessions, not previously recorded sessions.***  One of our project staff will contact you in a [few days (if did not acknowledge)/couple of weeks (if acknowledged)] to check to see if you have any questions or need any assistance. I know you are very busy and we appreciate you participating in this ALSO very critical part of the study to help understand youth treatment program delivery.

[IF R ONLY DOES GROUP THERAPY: We prefer that the recordings be for individual therapy sessions, but if you only do group sessions, please make sure you obtain consent for everyone and place the recorder next to you].

Can you confirm your e-mail address? _____________________________________

**Non-Sustainer Interview – CLINICIAN**

1. Based on an initial call, it sounded like your agency currently does not deliver the Adolescent Community Reinforcement Approach or A-CRA anymore. Is this correct?

🞏 No – ***SWITCH TO SUSTAINER INTERVIEW***🞏 Yes – ***CONTINUE***

1. Tell me a little bit about your agency.
2. What types of clients do you serve? (e.g., age range, gender, problem types).
3. What is your primary professional role(s) at your clinic/site?

🞏 Supervisor – ***SWITCH TO SUPERVISOR VERSION***

🞏 Counselor or Clinician – ***CONTINUE***
🞏 Supervisor and Counselor/Clinician ***– SWITCH TO SUP/CLIN VERSION***

1. Did you work on any of the following SAMHSA/CSAT A-CRA funded projects that your agency was awarded from the following dates?

|  | \| **START:** \|  \| \| --- \| --- \| \|  \|  \| \| **END:** \|  \| |
| --- | --- | --- | --- | --- | --- | --- | --- |
|  | \| **START:** \|  \| \| --- \| --- \| \|  \|  \| \| **END:** \|  \| |
|  | \| **START:** \|  \| \| --- \| --- \| \|  \|  \| \| **END:** \|  \| |
|  | \| **START:** \|  \| \| --- \| --- \| \|  \|  \| \| **END:** \|  \| |

🞏 No **– *SKIP TO QUESTION 7***🞏 Yes **– *CONTINUE***

1. Between what dates did you work on the project? Your best guess is fine.

Initiative Start Date End Date

|  | \| Month/Year: \|  \| \| --- \| --- \| \|  \|  \| | \| Month/Year: \|  \| \| --- \| --- \| |
| --- | --- | --- | --- | --- | --- | --- | --- | --- |
|  | \| Month/Year: \|  \| \| --- \| --- \| \|  \|  \| | \| Month/Year: \|  \| \| --- \| --- \| |
|  | \| Month/Year: \|  \| \| --- \| --- \| \|  \|  \| | \| Month/Year: \|  \| \| --- \| --- \| |
|  | \| Month/Year: \|  \| \| --- \| --- \| \|  \|  \| | \| Month/Year: \|  \| \| --- \| --- \| |

1. What role(s) did you serve while A-CRA was being delivered at your agency? *[MARK ALL THAT APPLY]*

🞏 A-CRA Therapist
🞏 A-CRA Supervisor
🞏 Other (Please describe: ____________________________________________________)
🞏 No Role in delivering ACRA

1. What do you think of A-CRA as a treatment for youth? Please note that throughout this interview, by “youth” we mean the population served during your SAMHSA CSAT grant periods.
2. When did you/your agency stop delivering A-CRA?

**Month/Year: ___________/_____________**

1. What were the main reasons you/your agency stopped delivering A-CRA?
2. What would have increased your desire to continue delivering A-CRA?
3. What would have increased your ability to continue delivering A-CRA?
4. Does your program currently offer any different types of treatment for youth with substance use disorders besides A-CRA?

🞏 No – ***SKIP TO QUESTION 15***🞏 Yes – ***CONTINUE***🞏 Don’t Know – ***SKIP TO QUESTION 15***

1. What are those treatments called? [Or describe the treatment. Is it outpatient? How long does it last? # of sessions/days/months] *[MARK ALL THAT APPLY]*
   1. Motivational Enhancement Therapy (MET)
   2. Cognitive Behavioral Therapy (CBT)
   3. MET/CBT-5
   4. Matrix Model
   5. Multidimensional Family Therapy (MDFT)
   6. Multisystemic Therapy (MST)
   7. 12-step facilitation
   8. Psychoeducation
   9. Supportive counseling
   10. Other____________________________________________________________

**Treatment programs may have an easier time implementing a new treatment if there are a number of elements in place to support them. For this next section, we want to ask you about things that could have affected A-CRA delivery at your present organization during the A-CRA funding period.**

1. First, we want to ask you about planning. Was there any strategic planning done to ensure resources were available to continue A-CRA beyond the initial funding period? By resources, we mean things needed to support A-CRA. For example, this could include things like money, staff, supervision, training, and A-CRA manuals.
2. Can you please describe any policies that supported A-CRA delivery? These could include external policies, such as funding source and billing rules that supported A-CRA.
3. What about policies that interfered with A-CRA delivery?
4. Did A-CRA meet the needs of the populations you serve? How so or why not?
5. Can you tell me about any pressure your organization experienced to continue delivering A-CRA or to discontinue its use?
6. Please describe whether leaders at your organization supported your use of A-CRA.
7. Would you say staff were supportive or reluctant to use A-CRA? Can you give me an example?
8. What do you think is the most important influence on the discontinuation of A-CRA at your organization?
9. In the 6 months prior to ending A-CRA, how did you or others at your agency disseminate information about your A-CRA project? By disseminated, we mean things like presentations, writing articles, media coverage, teaching, blogging, tweeting, etc.
   1. Presented to a local or state-level professional audience.
   2. Presented to a national or international professional audience.
   3. Wrote article(s) that was published or accepted for publication in a peer reviewed journal.
   4. Wrote article(s) that is under review or revision for a peer reviewed journal.
   5. Wrote article(s) for a newsletter or brochure or website.
   6. Had article(s) published in a local or state newspaper or on the internet.
   7. Received local or regional media coverage -- including print, television, radio broadcast, internet.
   8. Received national media coverage -- including print (NY Times, USA Today, Time, Newsweek, etc.), television or radio or internet
   9. Used information or materials from the project in teaching a college-level course.
   10. Developed a "how-to" manual or training package.
   11. Provided technical assistance to other agencies trying to implement similar projects.
   12. Wrote a blog about the project.
   13. Presented project on a website.
   14. Tweeted about the Project.
   15. None of the above.
   16. Other (please specify).
10. Before your organization made the decision not to sustain A-CRA, can you tell me about any efforts made to sustain after the end of its CSAT funding?
    1. Active support from your agency's executives or administrators.
    2. Actions by your agency's Board members.
    3. Program has become essential to carrying out the mission of your agency.
    4. Actions of an internal "champion" or key leader.
    5. Agency has existing "capacity" (e.g. enough staff member, skills, resources) to continue the program.
    6. You were able to modify the program to fit within your other programming.
    7. It is low cost - did not need substantial resources to continue.
    8. You have internal support for writing new grant proposals that helped obtain new resources.
    9. You had outcome/evaluation data that helped convince potential funders of the value of this program.
    10. Current or previous clients served as advocates for the program.
    11. Your staff members believed in the program so much they would not let it die.
    12. Partnerships with other organizations helped you find new funding sources.
    13. Your grant partners provide in-kind or other resources for continuation.
    14. Technical assistance or guidance from an external agency.
    15. Other helpful influence (please specify):
11. What barriers were encountered in attempting to sustain the project activities or services after the end of its CSAT funding?
    1. Obtaining funding from external sources.
    2. Obtaining funding from agency's budget, or other internal sources.
    3. Obtaining support from agency administrators for continuing project activities.
    4. Project leader or other key project "champion" left the agency.
    5. Turnover among staff members delivering project services.
    6. Agency priorities changed; ACRA approach is no longer a priority.
    7. Agency priorities changed; serving youth is no longer a priority.
    8. Maintaining agreement among partners essential for continuing project activities.
    9. Lack of physical space, such as lease terminated, project offices used for a different purpose, etc.
    10. Equipment or facilities needed (such as computers) no longer available.
    11. Policy changes that were agreed on temporarily during project were not made permanent.
    12. Partner agencies did not do what they promised.
    13. Other barriers (please specify):
12. On average, approximately how many session recordings did clinicians at your site complete to reach basic certification? Your best guess is fine.
13. Have you ever had a copy of the A-CRA manual?

🞏 No – ***SKIP TO QUESTION 31***

🞏 Yes – ***CONTINUE***

1. Please think about the six-month period, right before A-CRA treatment delivery ended. How often did you use the manual?

🞏 Never
🞏 A few times per year, or less
🞏 About once a month
🞏 A few times per month
🞏 Weekly
🞏 Daily

1. Do you still use your manual?

🞏 No – ***SKIP TO QUESTION 31***

🞏 Yes – ***CONTINUE***

1. How often do you use your manual now?

🞏 Never
🞏 A few times per year, or less
🞏 About once a month
🞏 A few times per month
🞏 Weekly
🞏 Daily

**For the next few questions, please think about the six-month period, right before A-CRA treatment delivery ended.**

1. How often did you have group clinical supervision?

🞏 Never – ***SKIP TO QUESTION 33***
🞏 Once a week
🞏 Every two weeks
🞏 Monthly
🞏 Other, please describe how often: ___________________________________

1. Please describe a typical group clinical supervision session during the six-month period before A-CRA treatment delivery ended.
2. Again, please think about the six-month period, right before A-CRA treatment delivery ended. How often did you have individual clinical supervision?

🞏 Never – ***SKIP TO QUESTION 35***
🞏 Once a week
🞏 Every two weeks
🞏 Monthly
🞏 Other, please describe how often: ___________________________________

1. Thinking about the past six-months, please describe a typical individual clinical supervision session. What topics do you usually discuss?

***IF “NEVER” TO QUESTION 31 AND QUESTION 33, SKIP AND GO TO QUESTION 47; OTHERWISE CONTINUE.***

1. (During the six-month period, right before A-CRA treatment delivery ended), what did your supervisor do, and what were you expected to do?

**During the six-month period, right before A-CRA treatment delivery ended, which of the following happened during supervision sessions?**

1. My supervisor asked how my week has gone.

🞏 No
🞏 Yes

1. My supervisor discussed agency paperwork requirements.

🞏 No
🞏 Yes

1. My supervisor reviewed my A-CRA case review report.

🞏 No
🞏 Yes

1. My supervisor asked me if I had any problem cases.

🞏 No
🞏 Yes

1. My supervisor reviewed a recorded session with me and told me what I did well.

🞏 No
🞏 Yes

1. My supervisor reviewed a recorded session with me and gave me suggestions about how I could improve my treatment delivery

🞏 No
🞏 Yes

1. My supervisor observed a live session and told me what I did well.

🞏 No
🞏 Yes

1. My supervisor observed a live session and gave me suggestions about how I could improve my treatment delivery

🞏 No
🞏 Yes

1. My supervisor asked me about my personal problems.

🞏 No
🞏 Yes

1. My supervisor role played with me the correct way to do a procedure.

🞏 No
🞏 Yes

1. Is there anything else you want to share about a typical supervision session during the six-month period, right before A-CRA treatment delivery ended?

**Now, I’d like to ask you the same questions, but now please think about the current practices in the adolescent treatment program.**

1. How often do you have group clinical supervision?

🞏 Never – ***SKIP TO QUESTION 49***
🞏 Once a week
🞏 Every two weeks
🞏 Monthly
🞏 Other, please describe how often: ___________________________________

1. Please describe a typical group clinical supervision session.
2. How often do you have individual clinical supervision?

🞏 Never – ***SKIP TO QUESITON 51***
🞏 Once a week
🞏 Every two weeks
🞏 Monthly
🞏 Other, please describe how often: ___________________________________

***IF “NEVER” TO BOTH QUESTION 47 AND QUESTION 49, GO TO QUESTION 63; OTHERWISE CONTINUE.***

1. Please describe a typical individual clinical supervision session. What topics do you usually discuss?
2. What does your supervisor do, and what are you expected to do?

**Which of the following happens during supervision sessions?**

1. My supervisor asks how my week has gone.

🞏 No
🞏 Yes

1. My supervisor discusses agency paperwork requirements.

🞏 No
🞏 Yes

1. My supervisor reviews my A-CRA case review report.

🞏 No
🞏 Yes

1. My supervisor asks me if I have any problem cases.

🞏 No
🞏 Yes

1. My supervisor reviews a recorded session with me and tells me what I have done well.

🞏 No
🞏 Yes

1. My supervisor reviews a recorded session with me and gives me suggestions about how I can improve my treatment delivery

🞏 No
🞏 Yes

1. My supervisor observes a live session and tells me what I have done well.

🞏 No
🞏 Yes

1. My supervisor observes a live session and gives me suggestions about how I can improve my treatment delivery

🞏 No
🞏 Yes

1. My supervisor asks me about my personal problems.

🞏 No
🞏 Yes

1. My supervisor role plays with me the correct way to do a procedure.

🞏 No
🞏 Yes

1. Is there anything else you want to share about a typical supervision session over the past six-months?

**Now, I’d like to ask you about trainings on treatment of use substance use.**

1. How many other trainings on youth substance use treatment have you participated in since you completed the A-CRA training?
2. What were the main topics of these trainings?
3. Do you prefer using any of the skills/tools/procedures you learned instead of A-CRA?

🞏 No
🞏 Yes

1. Why/why not?
2. During the six-month period, right before A-CRA treatment delivery ended, how many sessions did you tell the client receiving A-CRA he or she would have?

🞏 Less than 12
🞏 12 or more

1. During the six-month period, right before A-CRA treatment delivery ended, how many weeks did you tell the client receiving A-CRA the treatment would take?

🞏 Less than 12
🞏 12 or more

**Currently, when you are introducing your youth treatment program to a new client…**

1. How many sessions do you tell the client he or she will have?

🞏 Less than 12
🞏 12 or more

1. Currently, how many weeks do you tell the client the treatment will take?

🞏 Less than 12
🞏 12 or more

**Next, I want to ask you about your general impressions regarding the SAMHSA CSAT-funded A-CRA project and then have a few questions about you.**

1. If you had a chance to participate in a SAMHSA CSAT project again, would you consider it?

🞏 No
🞏 Yes

1. Why/why not?
2. Is there anything you would change about the SAMHSA CSAT project in order to improve the sustainment of A-CRA at your agency?
3. Is there anything else you think would have helped to sustain the use of A-CRA at your agency?
4. How many years of experience do you have in substance use counseling as a clinician?

# of years ____________ (OR) # of months ___________

1. Please provide the full name (as opposed to only the acronym) of all Licensures and/or Certifications you currently hold.

**END SCRIPT:**

We are now done with the interview. Here’s what happens next:

- In about a week, you will receive an e-mail from RAND with the link to a web survey and it will include your personal password. You can do it any date/time convenient for you, but please try to complete it within 2-3 weeks. At the last screen of the web survey, we will ask you for your contact information for your $50 honorarium check. We will only use that to send your check and it will not be attached to your survey.
- You will also receive a separate email with instructions on how to submit your 3 sample therapy recordings, in which you will receive another $50 and should try to do within the next month. The email will have detailed instructions and a consent form that needs to be completed for each client. ***These recordings should be of NEWLY recorded sessions, not previously recorded sessions.***  One of our project staff will contact you in a [few days (if did not acknowledge)/couple of weeks (if acknowledged)] to check to see if you have any questions or need any assistance. I know you are very busy and we appreciate you participating in this ALSO very critical part of the study to help understand youth treatment program delivery.

[IF R ONLY DOES GROUP THERAPY: We prefer that the recordings be for individual therapy sessions, but if you only do group sessions, please make sure you obtain consent for everyone and place the recorder next to you].

Can you confirm your e-mail address? _____________________________________

***CLINICIAN/SUPERVISOR INTERVIEW VERBAL CONSENT***

(READ PRIOR TO BEGINNING INTERVIEW/RECORDING)

Hi [CLINICIAN/SUPERVISOR NAME],

This is [INTERVIEWER NAME] calling from RAND to speak with you about the youth substance use treatment program and any experience you may have in implementing the Adolescent Community Reinforcement Approach (A-CRA). Is this still a good time to talk?

>IF NO, reschedule.

>IF YES, great.

Before we begin, let me assure you that your responses to these questions will be held in strict confidence. We are requesting interviews with staff at over 75 programs that were funded by the Centers of Substance Abuse Treatment to deliver the Adolescent Community Reinforcement Approach and we will aggregate information from the interviews to characterize programs that were funded over time. We will not attribute comments to specific individuals or programs in any of our reports or publications. Your participation will help us better understand how evidence-based treatments can be sustained in community practice settings after the initial support for delivery ends.

We would like to record the interview today to ensure that we capture everything that is said. We will destroy the recording as soon as we determine that we have captured everything in our notes.

After the interview, we will ask you to complete a 15–30-minute online survey to get a more complete picture. Upon receipt of the web survey, we will send you an honorarium check of $50. In addition, we are asking clinicians to upload audio recordings of three therapy sessions. After our discussion today, we will send you information and instructions for uploading the recordings. If you do upload three recordings, you will receive an additional honorarium check of $50, for a total of $100. ***These recordings should be of NEWLY recorded sessions, not previously recorded sessions.***

Your participation in this study is entirely voluntary. We would like to have your responses to all of the questions. However, if you’re uncomfortable with any question we can skip it. There are no right or wrong answers. We estimate that the interview will take about 45-60 minutes, depending on your answer to the questions.

- **Do you have any questions?**
- **Are you willing to take part in this discussion?**
- **Is it ok with you if we audio-record this discussion?**

**Sustainer Interview – CLINICAL SUPERVISOR & CLINICIAN**

1. Based on an initial call, it sounded like your agency currently delivers the Adolescent Community Reinforcement Approach or A-CRA. Is this correct?

🞏 No – ***SWITCH TO NON-SUSTAINER INTERVIEW***🞏 Yes – ***CONTINUE***

1. Tell me a little bit about your agency.
2. What types of clients do you serve? (e.g., age range, gender, problem types).
3. What is your primary professional role(s) at your clinic/site?

🞏 Supervisor – ***SWITCH TO SUPERVISOR VERSION***

🞏 Counselor or Clinician – ***SWITCH TO CLINICIAN VERSION***
🞏 Supervisor and Counselor/Clinician ***– CONTINUE***

1. Did you work on any of the following SAMHSA/CSAT A-CRA funded projects that your agency was awarded from the following dates?

|  | \| **START:** \|  \| \| --- \| --- \| \|  \|  \| \| **END:** \|  \| |
| --- | --- | --- | --- | --- | --- | --- | --- |
|  | \| **START:** \|  \| \| --- \| --- \| \|  \|  \| \| **END:** \|  \| |
|  | \| **START:** \|  \| \| --- \| --- \| \|  \|  \| \| **END:** \|  \| |
|  | \| **START:** \|  \| \| --- \| --- \| \|  \|  \| \| **END:** \|  \| |

🞏 No **– *SKIP TO QUESTION 7***🞏 Yes **– *CONTINUE***

1. Between what dates did you work on the project? Your best guess is fine.

Initiative Start Date End Date

|  | \| Month/Year: \|  \| \| --- \| --- \| \|  \|  \| | \| Month/Year: \|  \| \| --- \| --- \| |
| --- | --- | --- | --- | --- | --- | --- | --- | --- |
|  | \| Month/Year: \|  \| \| --- \| --- \| \|  \|  \| | \| Month/Year: \|  \| \| --- \| --- \| |
|  | \| Month/Year: \|  \| \| --- \| --- \| \|  \|  \| | \| Month/Year: \|  \| \| --- \| --- \| |
|  | \| Month/Year: \|  \| \| --- \| --- \| \|  \|  \| | \| Month/Year: \|  \| \| --- \| --- \| |

1. What role(s) have you served in delivering A-CRA? *[MARK ALL THAT APPLY]*

🞏 A-CRA Therapist
🞏 A-CRA Clinical Supervisor
🞏 Other (Please describe: ____________________________________________________)
🞏 No Role in delivering ACRA

1. What do you think of A-CRA as a treatment for youth? Please note that throughout this interview, by “youth” we mean the population served during your SAMHSA CSAT grant periods.
2. Does your program currently offer any different types of treatment for youth with substance use disorders besides A-CRA?

🞏 No – ***SKIP TO QUESTION 11***🞏 Yes – ***CONTINUE***🞏 Don’t Know – ***SKIP TO QUESTION 11***

1. What are those treatments called? [Or describe the treatment. Is it outpatient? How long does it last? # of sessions/days/months] *[MARK ALL THAT APPLY]*
   1. Motivational Enhancement Therapy (MET)
   2. Cognitive Behavioral Therapy (CBT)
   3. MET/CBT-5
   4. Matrix Model
   5. Multidimensional Family Therapy (MDFT)
   6. Multisystemic Therapy (MST)
   7. 12-step facilitation
   8. Psychoeducation
   9. Supportive counseling
   10. Other____________________________________________________________
2. Currently, what sources of funding or other resources do you use to support youth treatment delivery?
   1. Internal funding from agency's budget
   2. Private health insurance
   3. Medicaid/Medicare
   4. State budget or block grant funds
   5. Federal compliance grant (SAMHSA)
   6. State grant funding
   7. Grant from a private foundation
   8. Local fundraising drive(s)
   9. Users pay fee for service
   10. Volunteer or in-kind efforts used
   11. Other, specify: _______________________________________________________________________
3. What sources of funding or other resources do you now use to continue the activities or services that you started during the A-CRA grant?
   1. Internal funding from agency's budget
   2. Private health insurance
   3. Medicaid/Medicare
   4. State budget or block grant funds
   5. Federal compliance grant (SAMHSA)
   6. State grant funding
   7. Grant from a private foundation
   8. Local fundraising drive(s)
   9. Users pay fee for service
   10. Volunteer or in-kind efforts used
   11. Other, specify: _______________________________________________________________________
4. What is your best estimate of the current annual budget for your outpatient youth substance use treatment program (within $25,000 or so)?
5. Over the past 6 months, approximately how many youth received substance use treatment at your agency?
6. Approximately how many youth received A-CRA over the past 6 months?
7. Currently, how many clinicians at your agency treat youth with substance use problems?

16A. Can you tell me their names?

1. Currently, how many of the clinicians at your agency have been certified in A-CRA?
2. How many clinicians deliver A-CRA at your agency?

18A. Can you tell me their names?

***IF THE R CANNOT ANSWER THESE QUESTIONS UP TO THIS POINT, THANK THEM FOR HIS/HER TIME AND TRY TO IDENTIFY ANOTHER PERSON AT THE AGENCY WHO MAY BE BETTER TO ASK THESE QUESTIONS.***

***IF CURRENTLY FUNDED, GO TO QUESTION 21; OTHERWISE CONTINUE.***

1. While you were funded by SAMHSA/CSAT, were there other youth treatment programs in your local area?

🞏 No – ***GO TO QUESTION 21***
🞏 Yes – ***CONTINUE***

🞏 Don’t know – ***GO TO QUESTION 21***

1. Do/did they serve similar youth as your program?

🞏 No – PLEASE DESCRIBE HOW DIFFERENT: __________________
🞏 Yes

🞏 Don’t know

1. Currently, are there other youth treatment programs in your local area?

🞏 No – ***GO TO QUESTION 23***
🞏 Yes – ***CONTINUE***

🞏 Don’t know – ***GO TO QUESTION 23***

1. Do/did they serve similar youth as your program?

🞏 No – PLEASE DESCRIBE HOW DIFFERENT: __________________
🞏 Yes

🞏 Don’t know

1. Tell me about any ways in which your organization collaborates with academic or research institutions (colleges, universities).

**Treatment programs may have an easier time implementing a new treatment if there are a number of elements in place to support them. For this next section, we want to ask you about things that could affect A-CRA delivery at your organization.**

1. First, we want to ask you about planning. Tell me about any planning that has been done to ensure resources are available to continue A-CRA. By resources, we mean things needed to support A-CRA. For example, this could include things like money, staff, supervision, training, and A-CRA manuals.
2. Can you please describe any policies that support A-CRA delivery? These could include external policies, such as funding source and billing rules that support A-CRA.
3. What about policies that interfere with A-CRA delivery?

***IF CURRENTLY FUNDED CONTINUE THROUGH QUESTION 29, THEN, GO TO QUESTION 30; IF NOT CURRENLTY FUNDED GO TO THE “OTHERWISE VERSION.”***

**Next, I’d like to ask you about your agency’s plan to continue A-CRA clinical supervision support.**

1. First, does your agency have the finances to support the salary of a Clinical Supervisor?

🞏 No – ***GO TO QUESTION 30***
🞏 Yes

🞏 Don’t know

1. Does your agency plan to support Clinical Supervisor and Counselor time for biweekly supervision?

🞏 No
🞏 Yes – Describe, Explain:

1. Does your agency plan to support the Clinical Supervisor to listen to recorded therapy sessions and provide individualized feedback to counselors?

🞏 No
🞏 Yes – Describe, Explain:

***“OTHERWISE” VERSION— ASK IF NOT CURRENTLY FUNDED:***

**Next, I’d like to ask you about your agency’s A-CRA clinical supervision support over the last six months.**

27A. First, did your agency have the finances to support the salary of a Clinical Supervisor?

🞏 No – ***GO TO QUESTION 30***
🞏 Yes

🞏 Don’t know

28A. Second, did your agency support Clinical Supervisor and Counselor time for biweekly supervision?

🞏 No
🞏 Yes – Describe, Explain:

29A. Third, did your agency support the Clinical Supervisor to listen to recorded therapy sessions and provide individualized feedback to counselors?

🞏 No
🞏 Yes – Describe, Explain:

1. Does A-CRA meet the needs of the populations you serve? How so or why not?
2. Can you tell me about any pressure your organization experienced to continue delivering A-CRA or to discontinue its use?
3. Would you say staff are supportive or reluctant to use A-CRA? Can you give me an example?
4. What do you think is the most important influence on A-CRA’s success at your organization?
5. In the past 6 months, how have you or others at your agency disseminated information about your A-CRA project? By disseminated, we mean things like presentations, writing articles, media coverage, teaching, blogging, tweeting, etc.
   1. Presented to a local or state-level professional audience.
   2. Presented to a national or international professional audience.
   3. Wrote article(s) that was published or accepted for publication in a peer reviewed journal.
   4. Wrote article(s) that is under review or revision for a peer reviewed journal.
   5. Wrote article(s) for a newsletter or brochure or website.
   6. Had article(s) published in a local or state newspaper or on the internet.
   7. Received local or regional media coverage -- including print, television, radio broadcast, internet.
   8. Received national media coverage -- including print (NY Times, USA Today, Time, Newsweek, etc.), television or radio or internet
   9. Used information or materials from the project in teaching a college-level course.
   10. Developed a "how-to" manual or training package.
   11. Provided technical assistance to other agencies trying to implement similar projects.
   12. Wrote a blog about the project.
   13. Presented project on a website.
   14. Tweeted about the Project.
   15. None of the above.
   16. Other (please specify).
6. **[ONLY ASK FOR THOSE WHO FUNDING HAS ENDED]** What helped your efforts to sustain your project after the CSAT funding ended?
   1. Active support from your agency's executives or administrators.
   2. Actions by your agency's Board members.
   3. Program has become essential to carrying out the mission of your agency.
   4. Actions of an internal "champion" or key leader.
   5. Agency has existing "capacity" (e.g. enough staff member, skills, resources) to continue the program.
   6. You were able to modify the program to fit within your other programming.
   7. It is low cost - did not need substantial resources to continue.
   8. You have internal support for writing new grant proposals that helped obtain new resources.
   9. You had outcome/evaluation data that helped convince potential funders of the value of this program.
   10. Current or previous clients served as advocates for the program.
   11. Your staff members believed in the program so much they would not let it die.
   12. Partnerships with other organizations helped you find new funding sources.
   13. Your grant partners provide in-kind or other resources for continuation.
   14. Technical assistance or guidance from an external agency.
   15. Other helpful influence (please specify):
7. **[ONLY ASK FOR THOSE WHO FUNDING HAS ENDED]** What barriers have you encountered in attempting to sustain the project activities or services after the end of its CSAT funding?
   1. Obtaining funding from external sources.
   2. Obtaining funding from agency's budget, or other internal sources.
   3. Obtaining support from agency administrators for continuing project activities.
   4. Project leader or other key project "champion" left the agency.
   5. Turnover among staff members delivering project services.
   6. Agency priorities changed; ACRA approach is no longer a priority.
   7. Agency priorities changed; serving youth is no longer a priority.
   8. Maintaining agreement among partners essential for continuing project activities.
   9. Lack of physical space, such as lease terminated, project offices used for a different purpose, etc.
   10. Equipment or facilities needed (such as computers) no longer available.
   11. Policy changes that were agreed on temporarily during project were not made permanent.
   12. Partner agencies did not do what they promised.
   13. Other barriers (please specify):

**I will now ask you about the clinicians at your agency.**

1. Do you supervise clinicians who treat youth for substance use problems?

🞏 No – ***GO TO QUESTION 39***
🞏 Yes – ***CONTINUE***

1. How many of these clinicians are currently delivering A-CRA?

**Next, I’ll ask you about the supervisors at your agency.**

1. How many staff supervise clinicians who treat youth with substance use problems?
2. Do you supervise clinicians who deliver A-CRA?

🞏 No
🞏 Yes

🞏 Don’t know

1. How many others at your agency supervise clinicians delivering A-CRA?

🞏 Don’t know

1. Have you personally certified any clinicians in A-CRA?

🞏 No – ***GO TO QUESTION 59***
🞏 Yes – ***CONTINUE***

1. You mentioned that you personally had certified one or more clinicians in A-CRA at your organization. Can you please describe the certification process to me?

The clinician was asked to:

🞏 Read the A-CRA manual

🞏 Take an online A-CRA research course

🞏 Pass an A-CRA quiz with a score of 80% or higher

🞏 Attend a Chestnut or RJM A-CRA initial training OR attend an in-house training

🞏 Participate in regular coaching calls with Chestnut or regular supervision with in-house Clinical Supervisor (respondent) during certification (regular= @ every other week)

🞏 Regularly record therapy sessions for in-house Clinical Supervisor (respondent) review/Chestnut’s review (regular= at least some sessions weekly))

🞏 Demonstrate competency in General Clinical Skills on the DSRs

🞏 Demonstrate competency in the following A-CRA procedures:

Functional Analysis of Use

Functional Analysis of Pro-social behavior

Happiness Scale

Treatment Plan/Goals of Counseling

Communication Skills

Problem Solving Skills

Adolescent-Caregiver Relationship Skills, and

Homework based of 3 or better on all components of a given procedure using the A-CRA rating manual?

If in-house training was provided, did it?

🞏 Include didactic information about A-CRA procedures?

🞏 Modeling or review of audio recordings of procedures that were well done

🞏 The opportunity to role play procedures

🞏 Other, please explain_____________________________________

**Next, I’m going to ask you a series of questions about how the certification process currently works in your agency. Please respond by saying “True,” “False,” or “Don’t Know” if you are unsure.**

1. When I decide to pass a clinician on a procedure it is based on ratings of 1 or more on every component of a procedure. Remember that each component of a procedure is rated on a 1 to 5 scale.

🞏 False
🞏 True
🞏 Don’t Know

1. Communication skills is a procedure that people have to pass to attain certification.

🞏 False
🞏 True
🞏 Don’t Know

1. I review recorded sessions during the certification process.

🞏 False
🞏 True
🞏 Don’t Know

1. I am required to sit in sessions with clinicians during the certification process.

🞏 False
🞏 True
🞏 Don’t Know

1. Clinicians record one or two of their sessions.

🞏 False
🞏 True
🞏 Don’t Know

1. I refer to the A-CRA rating manual when rating session recordings.

🞏 False
🞏 True
🞏 Don’t Know

1. Clinicians are required to take a knowledge test as part of the certification process.

🞏 False
🞏 True
🞏 Don’t Know

1. Clinicians are not required to pass General Clinical Skills as part of the certification process.

🞏 False
🞏 True
🞏 Don’t Know

1. Each clinician has a certification workbook.

🞏 False
🞏 True
🞏 Don’t Know

1. I complete the A-CRA checklist when I am listening to a recorded session during or after the certification process.

🞏 False
🞏 True
🞏 Don’t Know

1. Clinicians are required to read the A-CRA manual during the training process.

🞏 False
🞏 True
🞏 Don’t Know

1. Time is set aside for training clinicians in A-CRA

🞏 False
🞏 True
🞏 Don’t Know

1. During training clinicians are required to practice procedures with role-plays.

🞏 False
 🞏 True
 🞏 Don’t Know

1. Adolescent-Caregiver Relationship Skills is one of the procedures for basic certification.

🞏 False
🞏 True
🞏 Don’t Know

1. It doesn't matter if clinicians show competency in all of the additional procedures as well.

🞏 False
🞏 True
🞏 Don’t Know

1. On average, approximately how many session recordings do clinicians at your site complete to reach basic certification? Your best guess is fine.
2. Do you have a copy of the A-CRA manual?

🞏 No – ***SKIP TO QUESTION 62***

🞏 Yes – ***CONTINUE***

1. In the past six-months, how often would you say you used the manual?

🞏 Never
🞏 A few times per year, or less
🞏 About once a month
🞏 A few times per month
🞏 Weekly
🞏 Daily

1. In the past six-months, how often do you have group clinical supervision?

🞏 Never
🞏 Once a week
🞏 Every two weeks
🞏 Monthly
🞏 Other, please describe how often: ___________________________________

1. Thinking about the past six-months, please describe a typical group clinical supervision session.
2. In the past six-months, how often do you have individual clinical supervision?

🞏 Never
🞏 Once a week
🞏 Every two weeks
🞏 Monthly
🞏 Other, please describe how often: ___________________________________

1. Thinking about the past six-months, please describe a typical individual clinical supervision session. What topics do you usually discuss?

**Now, I’d like to ask you about A-CRA training.**

1. How many new clinicians have been trained in A-CRA in the past six-months in the organization?
2. How many new clinicians have been trained in A-CRA in the past six-months at a Chestnut or Robert J. Meyers training?
3. Are there training agendas for your trainings?

🞏 No – ***GO TO QUESTION 70***
🞏 Yes – ***CONTINUE***

1. Will you please send me (email, mail, fax) a copy of the agenda?

🞏 No
🞏 Yes

**Now, I’d like to ask you about trainings on treatment of use substance use.**

1. How many other trainings on youth substance use treatment have you participated in since you completed the A-CRA training?
2. What were the main topics of these trainings?
3. Do you prefer using any of the skills/tools/procedures you learned instead of A-CRA?

🞏 No
🞏 Yes

1. Why/why not?

**Currently, when you are introducing A-CRA to a new client…**

1. How many sessions do you tell the client receiving A-CRA he or she will have?

🞏 Less than 12
🞏 12 or more

1. How many weeks do you tell the client receiving A-CRA the treatment will take?

🞏 Less than 12
🞏 12 or more

**Next, I want to ask you about your general impressions regarding the SAMHSA CSAT-funded A-CRA project and then have a few questions about you.**

1. If you had a chance to participate in a SAMHSA CSAT project again, would you consider it?

🞏 No
🞏 Yes

1. Why/why not?
2. Is there anything you would change about the SAMHSA CSAT project in order to improve the sustainment of A-CRA at your agency?
3. Is there anything else you would like to share related to the topic of sustaining A-CRA at your agency?
4. How many years of experience do you have in substance use counseling as a clinician?

# of years ____________ (OR) # of months ___________

1. Do you have any experience as an administrator of youth substance use treatment programs?

🞏 No – ***GO TO QUESTION 83***
🞏 Yes – ***CONTINUE***

1. How many years of experience as an administrator do you have?

# of years ____________ (OR) # of months ___________

1. How many years of experience do you have as a clinical supervisor for substance use counselors/clinicians?

# of years ____________ (OR) # of months ___________

1. Please provide the full name (as opposed to only the acronym) of all Licensures and/or Certifications you currently hold.

**END SCRIPT:**

We are now done with the interview. Here’s what happens next:

- In about a week, you will receive an e-mail from RAND with the link to a web survey and it will include your personal password. You can do it any date/time convenient for you, but please try to complete it within 2-3 weeks. At the last screen of the web survey, we will ask you for your contact information for your $50 honorarium check. We will only use that to send your check and it will not be attached to your survey.
- You will also receive a separate email with instructions on how to submit your 3 sample therapy recordings, in which you will receive another $50 and should try to do within the next month. The email will have detailed instructions and a consent form that needs to be completed for each client. ***These recordings should be of NEWLY recorded sessions, not previously recorded sessions.***  One of our project staff will contact you in a [few days (if did not acknowledge)/couple of weeks (if acknowledged)] to check to see if you have any questions or need any assistance. I know you are very busy and we appreciate you participating in this ALSO very critical part of the study to help understand youth treatment program delivery.

[IF R ONLY DOES GROUP THERAPY: We prefer that the recordings be for individual therapy sessions, but if you only do group sessions, please make sure you obtain consent for everyone and place the recorder next to you].

Can you confirm your e-mail address? _____________________________________

**Non-Sustainer Interview – CLINICAL SUPERVISOR & CLINICIAN**

1. Based on an initial call, it sounded like your agency currently does not deliver the Adolescent Community Reinforcement Approach or A-CRA. Is this correct?

🞏 No – ***SWITCH TO SUSTAINER INTERVIEW***🞏 Yes – ***CONTINUE***

1. Tell me a little bit about your agency.
2. What types of clients do you serve? (e.g., age range, gender, problem types).
3. What is your primary professional role(s) at your clinic/site?

🞏 Supervisor – ***SWITCH TO SUPERVISOR VERSION***

🞏 Counselor or Clinician – ***SWITCH TO CLINICIAN VERSION***
🞏 Supervisor and Counselor/Clinician ***– CONTINUE***

1. Did you work on any of the following SAMHSA/CSAT A-CRA funded projects that your agency was awarded from the following dates?

|  | \| **START:** \|  \| \| --- \| --- \| \|  \|  \| \| **END:** \|  \| |
| --- | --- | --- | --- | --- | --- | --- | --- |
|  | \| **START:** \|  \| \| --- \| --- \| \|  \|  \| \| **END:** \|  \| |
|  | \| **START:** \|  \| \| --- \| --- \| \|  \|  \| \| **END:** \|  \| |
|  | \| **START:** \|  \| \| --- \| --- \| \|  \|  \| \| **END:** \|  \| |

🞏 No **– *SKIP TO QUESTION 7***🞏 Yes **– *CONTINUE***

1. Between what dates did you work on the project? Your best guess is fine.

Initiative Start Date End Date

|  | \| Month/Year: \|  \| \| --- \| --- \| \|  \|  \| | \| Month/Year: \|  \| \| --- \| --- \| |
| --- | --- | --- | --- | --- | --- | --- | --- | --- |
|  | \| Month/Year: \|  \| \| --- \| --- \| \|  \|  \| | \| Month/Year: \|  \| \| --- \| --- \| |
|  | \| Month/Year: \|  \| \| --- \| --- \| \|  \|  \| | \| Month/Year: \|  \| \| --- \| --- \| |
|  | \| Month/Year: \|  \| \| --- \| --- \| \|  \|  \| | \| Month/Year: \|  \| \| --- \| --- \| |

1. What role(s) did you serve while A-CRA was being delivered at your agency? *[MARK ALL THAT APPLY]*

🞏 A-CRA Therapist
🞏 A-CRA Supervisor
🞏 Other (Please describe: ____________________________________________________
🞏 No Role in delivering ACRA

1. What do you think of A-CRA as a treatment for youth? Please note that throughout this interview, by “youth” we mean the population served during your SAMHSA CSAT grant periods.
2. When did you/your agency stop delivering A-CRA?

**Month/Year: ___________/_____________**

1. Approximately how many youth received A-CRA during the six-month period, right before A-CRA treatment delivery ended?
2. What were the main reasons you/your agency stopped delivering A-CRA?
3. What would have increased your desire to continue delivering A-CRA?
4. What would have increased your ability to continue delivering A-CRA?
5. Does your program currently offer any different types of treatment for youth with substance use disorders besides A-CRA?

🞏 No – ***SKIP TO QUESTION 16***🞏 Yes – ***CONTINUE***🞏 Don’t Know – ***SKIP TO QUESTION 16***

1. What are those treatments called? [Or describe the treatment. Is it outpatient? How long does it last? # of sessions/days/months] *[MARK ALL THAT APPLY]*
   1. Motivational Enhancement Therapy (MET)
   2. Cognitive Behavioral Therapy (CBT)
   3. MET/CBT-5
   4. Matrix Model
   5. Multidimensional Family Therapy (MDFT)
   6. Multisystemic Therapy (MST)
   7. 12-step facilitation
   8. Psychoeducation
   9. Supportive counseling
   10. Other____________________________________________________________
2. Currently, what sources of funding or other resources do you use to support youth treatment delivery?
   1. Internal funding from agency's budget
   2. Private health insurance
   3. Medicaid/Medicare
   4. State budget or block grant funds
   5. Federal compliance grant (SAMHSA)
   6. State grant funding
   7. Grant from a private foundation
   8. Local fundraising drive(s)
   9. Users pay fee for service
   10. Volunteer or in-kind efforts used
   11. Other, specify: _______________________________________________________________________
3. What is your best estimate of the current annual budget for your outpatient youth substance use treatment program (within $25,000 or so)?
4. Over the past 6 months, approximately how many youth received substance use treatment at your agency?
5. Approximately how many youth received A-CRA over the past 6 months?
6. Currently, how many clinicians at your agency treat youth with substance use problems?

20A. Can you tell me their names?

1. Currently, how many of the clinicians at your agency have been certified in A-CRA?

21B. Can you tell me their names?

1. While you were funded by SAMHSA/CSAT, were there other youth treatment programs in your local area?

🞏 No – ***GO TO QUESTION 24***
🞏 Yes – ***CONTINUE***

🞏 Don’t know – ***GO TO QUESTION 24***

1. Do/did they serve similar youth as your program?

🞏 No – PLEASE DESCRIBE HOW DIFFERENT: __________________
🞏 Yes

🞏 Don’t know

1. Currently, are there other youth treatment programs in your local area?

🞏 No – ***GO TO QUESTION 26***
🞏 Yes – ***CONTINUE***

🞏 Don’t know – ***GO TO QUESTION 26***

1. Do/did they serve similar youth as your program?

🞏 No – PLEASE DESCRIBE HOW DIFFERENT: __________________
🞏 Yes

🞏 Don’t know

1. Tell me about any ways in which your organization collaborates with academic or research institutions (colleges, universities).

**Treatment programs may have an easier time implementing a new treatment if there are a number of elements in place to support them. For this next section, we want to ask you about things that could have affected A-CRA delivery at your present organization during the A-CRA funding period.**

1. First, we want to ask you about planning. Was there any strategic planning done to ensure resources were available to continue A-CRA beyond the initial funding period? By resources, we mean things needed to support A-CRA. For example, this could include things like money, staff, supervision, training, and A-CRA manuals.
2. Can you please describe any policies that supported A-CRA delivery? These could include external policies, such as funding source and billing rules that supported A-CRA.
3. What about policies that interfered with A-CRA delivery?

**Next, I’d like to ask you about your agency’s A-CRA clinical supervision support during the last six months it was delivered.**

1. First, did your agency have the finances to support the salary of a Clinical Supervisor?

🞏 No – ***GO TO QUESTION 33***
🞏 Yes

🞏 Don’t know

1. Second, did your agency support Clinical Supervisor and Counselor time for biweekly supervision?

🞏 No
🞏 Yes – Describe, Explain:

1. Third, did your agency support the Clinical Supervisor to listen to recorded therapy sessions and provide individualized feedback to counselors?

🞏 No
🞏 Yes – Describe, Explain:

1. Did A-CRA meet the needs of the populations you serve? How so or why not?
2. Can you tell me about any pressure your organization experienced to continue delivering A-CRA or to discontinue its use?
3. Would you say staff were supportive or reluctant to use A-CRA? Can you give me an example?
4. What do you think is the most important influence on the discontinuation of A-CRA at your organization?
5. In the 6 months prior to ending A-CRA, how did you or others at your agency disseminate information about your A-CRA project? By disseminated, we mean things like presentations, writing articles, media coverage, teaching, blogging, tweeting, etc.
   1. Presented to a local or state-level professional audience.
   2. Presented to a national or international professional audience.
   3. Wrote article(s) that was published or accepted for publication in a peer reviewed journal.
   4. Wrote article(s) that is under review or revision for a peer reviewed journal.
   5. Wrote article(s) for a newsletter or brochure or website.
   6. Had article(s) published in a local or state newspaper or on the internet.
   7. Received local or regional media coverage -- including print, television, radio broadcast, internet.
   8. Received national media coverage -- including print (NY Times, USA Today, Time, Newsweek, etc.), television or radio or internet
   9. Used information or materials from the project in teaching a college-level course.
   10. Developed a "how-to" manual or training package.
   11. Provided technical assistance to other agencies trying to implement similar projects.
   12. Wrote a blog about the project.
   13. Presented project on a website.
   14. Tweeted about the Project.
   15. None of the above.
   16. Other (please specify).
6. What barriers were encountered in attempting to sustain the project activities or services after the end of its CSAT funding?
   1. Obtaining funding from external sources.
   2. Obtaining funding from agency's budget, or other internal sources.
   3. Obtaining support from agency administrators for continuing project activities.
   4. Project leader or other key project "champion" left the agency.
   5. Turnover among staff members delivering project services.
   6. Agency priorities changed; ACRA approach is no longer a priority.
   7. Agency priorities changed; serving youth is no longer a priority.
   8. Maintaining agreement among partners essential for continuing project activities.
   9. Lack of physical space, such as lease terminated, project offices used for a different purpose, etc.
   10. Equipment or facilities needed (such as computers) no longer available.
   11. Policy changes that were agreed on temporarily during project were not made permanent.
   12. Partner agencies did not do what they promised.
   13. Other barriers (please specify):

**Now I'm going to ask you a few questions about each of the clinicians who treat youth for substance use problems at your agency.**

1. Do you currently supervise clinicians who treat youth for substance use problems?

🞏 No
🞏 Yes

**Thank you. I now have some questions about the supervisors of clinicians delivering youth treatment at your agency.**

1. How many staff supervise clinicians who treat youth with substance use problems?
2. Did you supervise clinicians who deliver A-CRA?

🞏 No
🞏 Yes

🞏 Don’t know

1. How many others at your agency supervised clinicians delivering A-CRA?

🞏 Don’t know

1. Have you personally certified any clinicians in A-CRA?

🞏 No – ***GO TO QUESTION 60***
🞏 Yes – ***CONTINUE***

1. You mentioned that you personally had certified one or more clinicians in A-CRA at your organization. Can you please describe the certification process to me?

The clinician was asked to:

🞏 Read the A-CRA manual

🞏 Take an online A-CRA research course

🞏 Pass an A-CRA quiz with a score of 80% or higher

🞏 Attend a Chestnut or RJM A-CRA initial training OR attend an in-house training

🞏 Participate in regular coaching calls with Chestnut or regular supervision with in-house Clinical Supervisor (respondent) during certification (regular= @ every other week)

🞏 Regularly record therapy sessions for in-house Clinical Supervisor (respondent) review/Chestnut’s review (regular= at least some sessions weekly))

🞏 Demonstrate competency in General Clinical Skills on the DSRs

🞏 Demonstrate competency in the following A-CRA procedures:

Functional Analysis of Use

Functional Analysis of Pro-social behavior

Happiness Scale

Treatment Plan/Goals of Counseling

Communication Skills

Problem Solving Skills

Adolescent-Caregiver Relationship Skills, and

Homework based of 3 or better on all components of a given procedure using the A-CRA rating manual?

If in-house training was provided, did it?

🞏 Include didactic information about A-CRA procedures?

🞏 Modeling or review of audio recordings of procedures that were well done

🞏 The opportunity to role play procedures

🞏 Other, please explain_____________________________________

**Next, I’m going to ask you a series of questions about how the A-CRA certification process worked in your agency. Please respond by saying “True,” “False,” or “Don’t Know” if you are unsure.**

1. When I decided to pass a clinician on a procedure it was based on ratings of 1 or more on every component of a procedure. Remember that each component of a procedure is rated on a 1 to 5 scale.

🞏 False
🞏 True
🞏 Don’t Know

1. Communication skills was a procedure that people had to pass to attain certification.

🞏 False
🞏 True
🞏 Don’t Know

1. I reviewed recorded sessions during the certification process.

🞏 False
🞏 True
🞏 Don’t Know

1. I was required to sit in sessions with clinicians during the certification process.

🞏 False
🞏 True
🞏 Don’t Know

1. Clinicians recorded one or two of their sessions.

🞏 False
🞏 True
🞏 Don’t Know

1. I referred to the A-CRA rating manual when rating session recordings.

🞏 False
🞏 True
🞏 Don’t Know

1. Clinicians were required to take a knowledge test as part of the certification process.

🞏 False
🞏 True
🞏 Don’t Know

1. Clinicians were not required to pass General Clinical Skills as part of the certification process.

🞏 False
🞏 True
🞏 Don’t Know

1. Each clinician had a certification workbook.

🞏 False
🞏 True
🞏 Don’t Know

1. I completed the A-CRA checklist when I was listening to a recorded session during or after the certification process.

🞏 False
🞏 True
🞏 Don’t Know

1. Clinicians were required to read the A-CRA manual during the training process.

🞏 False
🞏 True
🞏 Don’t Know

1. Time was set aside for training clinicians in A-CRA

🞏 False
🞏 True
🞏 Don’t Know

1. During training clinicians were required to practice procedures with role-plays.

🞏 False
 🞏 True
 🞏 Don’t Know

1. Adolescent-Caregiver Relationship Skills was one of the procedures for basic certification.

🞏 False
🞏 True
🞏 Don’t Know

1. It didn’t matter if clinicians show competency in all of the additional procedures as well.

🞏 False
🞏 True
🞏 Don’t Know

1. On average, approximately how many session recordings did clinicians at your site complete to reach basic certification? Your best guess is fine.
2. Have you ever had a copy of the A-CRA manual?

🞏 No – ***SKIP TO QUESTION 65***

🞏 Yes – ***CONTINUE***

1. Please think about the six-month period, right before A-CRA treatment delivery ended. How often did you use the manual?

🞏 Never
🞏 A few times per year, or less
🞏 About once a month
🞏 A few times per month
🞏 Weekly
🞏 Daily

1. Do you still use your manual?

🞏 No – ***SKIP TO QUESTION 65***
🞏 Yes – ***CONTINUE***

1. How often do you use your manual now?

🞏 Never
🞏 A few times per year, or less
🞏 About once a month
🞏 A few times per month
🞏 Weekly
🞏 Daily

**For the next few questions, please think about the six-month period, right before A-CRA treatment delivery ended.**

1. How often did you have group clinical supervision?

🞏 Never – ***SKIP TO QUESTION 67***
🞏 Once a week
🞏 Every two weeks
🞏 Monthly
🞏 Other, please describe how often: ___________________________________

1. Please describe a typical group clinical supervision session during the six-month period before A-CRA treatment delivery ended.
2. Again, please think about the six-month period, right before A-CRA treatment delivery ended. How often did you have individual clinical supervision?

🞏 Never – ***SKIP TO QUESTION 69***
🞏 Once a week
🞏 Every two weeks
🞏 Monthly
🞏 Other, please describe how often: ___________________________________

1. (During the six-month period, right before A-CRA treatment delivery ended), please describe a typical individual clinical supervision session. What topics did you usually discuss?

**Now I’d like to ask you the same questions, but now please think about the current practices in the adolescent treatment program.**

1. How often do you have group clinical supervision?

🞏 Never
🞏 Once a week
🞏 Every two weeks
🞏 Monthly
🞏 Other, please describe how often: ___________________________________

1. Please describe a typical group clinical supervision session.
2. How often do you have individual clinical supervision?

🞏 Never
🞏 Once a week
🞏 Every two weeks
🞏 Monthly
🞏 Other, please describe how often: ___________________________________

1. Please describe a typical individual clinical supervision session. What topics do you usually discuss?

**Now, I’d like to ask you about trainings on treatment of use substance use.**

1. How many other trainings on youth substance use treatment have you participated in since you completed the A-CRA training?
2. What were the main topics of these trainings?
3. Do you prefer using any of the skills/tools/procedures you learned instead of A-CRA?

🞏 No
🞏 Yes

1. Why/why not?
2. During the six-month period, right before A-CRA treatment delivery ended, how many sessions did you tell the client receiving A-CRA he or she would have?

🞏 Less than 12
🞏 12 or more

1. During the six-month period, right before A-CRA treatment delivery ended, how many weeks did you tell the client receiving A-CRA the treatment would take?

🞏 Less than 12
🞏 12 or more

**Currently, when you are introducing your youth treatment program to a new client…**

1. How many sessions do you tell the client he or she will have?

🞏 Less than 12
🞏 12 or more

1. How many weeks do you tell the client the treatment will take?

🞏 Less than 12
🞏 12 or more

**Next, I want to ask you about your general impressions regarding the SAMHSA CSAT-funded A-CRA project and then have a few questions about you.**

1. If you had a chance to participate in a SAMHSA CSAT project again, would you consider it?

🞏 No
🞏 Yes

1. Why/why not?
2. Is there anything you would change about the SAMHSA CSAT project in order to improve the sustainment of A-CRA at your agency?
3. Is there anything else you think would have helped to sustain the use of A-CRA at your agency?
4. How many years of experience do you have in substance use counseling as a clinician?

# of years ____________ (OR) # of months ___________

1. Do you have any experience as an administrator of youth substance use treatment programs?

🞏 No – ***GO TO QUESTION 88***
🞏 Yes – ***CONTINUE***

1. How many years of experience as an administrator do you have?

# of years ____________ (OR) # of months ___________

1. How many years of experience do you have as a clinical supervisor for substance use counselors/clinicians?

# of years ____________ (OR) # of months ___________

1. Please provide the full name (as opposed to only the acronym) of all Licensures and/or Certifications you currently hold.

**END SCRIPT:**

We are now done with the interview. Here’s what happens next:

- In about a week, you will receive an e-mail from RAND with the link to a web survey and it will include your personal password. You can do it any date/time convenient for you, but please try to complete it within 2-3 weeks. At the last screen of the web survey, we will ask you for your contact information for your $50 honorarium check. We will only use that to send your check and it will not be attached to your survey.
- You will also receive a separate email with instructions on how to submit your 3 sample therapy recordings, in which you will receive another $50 and should try to do within the next month. The email will have detailed instructions and a consent form that needs to be completed for each client. ***These recordings should be of NEWLY recorded sessions, not previously recorded sessions.***  One of our project staff will contact you in a [few days (if did not acknowledge)/couple of weeks (if acknowledged)] to check to see if you have any questions or need any assistance. I know you are very busy and we appreciate you participating in this ALSO very critical part of the study to help understand youth treatment program delivery.

[IF R ONLY DOES GROUP THERAPY: We prefer that the recordings be for individual therapy sessions, but if you only do group sessions, please make sure you obtain consent for everyone and place the recorder next to you].

Can you confirm your e-mail address? ____________________________________

***SUPERVISOR INTERVIEW VERBAL CONSENT***

(READ PRIOR TO BEGINNING INTERVIEW/RECORDING)

Hi [SUPERVISOR NAME],

This is [INTERVIEWER NAME] calling from RAND to speak with you about the youth substance use treatment program and any experience you may have in implementing the Adolescent Community Reinforcement Approach (A-CRA). Is this still a good time to talk?

>IF NO, reschedule.

>IF YES, great.

Before we begin, let me assure you that your responses to these questions will be held in strict confidence. We are requesting interviews with staff at over 75 programs that were funded by the Centers of Substance Abuse Treatment to deliver the Adolescent Community Reinforcement Approach and we will aggregate information from the interviews to characterize programs that were funded over time. We will not attribute comments to specific individuals or programs in any of our reports or publications. Your participation will help us better understand how evidence-based treatments can be sustained in community practice settings after the initial support for delivery ends.

We would like to record the interview today to ensure that we capture everything that is said. We will destroy the recording as soon as we determine that we have captured everything in our notes.

After the interview, we will ask you to complete a 15–30-minute online survey to get a more complete picture. Upon receipt of the web survey, we will send you an honorarium check of $50. In addition, we are asking clinicians to upload audio recordings of three therapy sessions. After our discussion today, we will send you information and instructions for uploading the recordings. If you do upload three recordings, you will receive an additional honorarium check of $50, for a total of $100. ***These recordings should be of NEWLY recorded sessions, not previously recorded sessions.***

Your participation in this study is entirely voluntary. We would like to have your responses to all of the questions. However, if you’re uncomfortable with any question we can skip it. There are no right or wrong answers. We estimate that the interview will take about 45-60 minutes, depending on your answer to the questions.

- **Do you have any questions?**
- **Are you willing to take part in this discussion?**
- **Is it ok with you if we audio-record this discussion?**

**Sustainer Interview – SUPERVISOR**

1. Based on an initial call, it sounded like your agency currently delivers the Adolescent Community Reinforcement Approach or A-CRA. Is this correct?

🞏 No – ***SWITCH TO NON-SUSTAINER INTERVIEW***🞏 Yes – ***CONTINUE***

1. Tell me a little bit about your agency.
2. What types of clients do you serve? (e.g., age range, gender, problem types).
3. What is your primary professional role(s) at your clinic/site?

🞏 Supervisor – ***SWITCH TO SUPERVISOR VERSION***

🞏 Counselor or Clinician – ***CONTINUE***
🞏 Supervisor and Counselor/Clinician ***– SWITCH TO SUP/CLIN VERSION***

1. Did you work on any of the following SAMHSA/CSAT A-CRA funded projects that your agency was awarded from the following dates?

|  | \| **START:** \|  \| \| --- \| --- \| \|  \|  \| \| **END:** \|  \| |
| --- | --- | --- | --- | --- | --- | --- | --- |
|  | \| **START:** \|  \| \| --- \| --- \| \|  \|  \| \| **END:** \|  \| |
|  | \| **START:** \|  \| \| --- \| --- \| \|  \|  \| \| **END:** \|  \| |
|  | \| **START:** \|  \| \| --- \| --- \| \|  \|  \| \| **END:** \|  \| |

🞏 No **– *SKIP TO QUESTION 7***🞏 Yes **– *CONTINUE***

1. Between what dates did you work on the project? Your best guess is fine.

Initiative Start Date End Date

|  | \| Month/Year: \|  \| \| --- \| --- \| \|  \|  \| | \| Month/Year: \|  \| \| --- \| --- \| |
| --- | --- | --- | --- | --- | --- | --- | --- | --- |
|  | \| Month/Year: \|  \| \| --- \| --- \| \|  \|  \| | \| Month/Year: \|  \| \| --- \| --- \| |
|  | \| Month/Year: \|  \| \| --- \| --- \| \|  \|  \| | \| Month/Year: \|  \| \| --- \| --- \| |
|  | \| Month/Year: \|  \| \| --- \| --- \| \|  \|  \| | \| Month/Year: \|  \| \| --- \| --- \| |

1. What role(s) have you served in delivering A-CRA? *[MARK ALL THAT APPLY]*

🞏 A-CRA Therapist
🞏 A-CRA Supervisor
🞏 Other (Please describe: ____________________________________________________)
🞏 No Role in delivering ACRA

1. What do you think of A-CRA as a treatment for youth? Please note that throughout this interview, by “youth” we mean the population served during your SAMHSA CSAT grant periods.
2. Does your program currently offer any different types of treatment for youth with substance use disorders besides A-CRA?

🞏 No – ***SKIP TO QUESTION 11***🞏 Yes – ***CONTINUE***🞏 Don’t Know – ***SKIP TO QUESTION 11***

1. What are those treatments called? [Or describe the treatment. Is it outpatient? How long does it last? # of sessions/days/months] *[MARK ALL THAT APPLY]*
   1. Motivational Enhancement Therapy (MET)
   2. Cognitive Behavioral Therapy (CBT)
   3. MET/CBT-5
   4. Matrix Model
   5. Multidimensional Family Therapy (MDFT)
   6. Multisystemic Therapy (MST)
   7. 12-step facilitation
   8. Psychoeducation
   9. Supportive counseling
   10. Other____________________________________________________________
2. Currently, what sources of funding or other resources do you use to support youth treatment delivery?
   1. Internal funding from agency's budget
   2. Private health insurance
   3. Medicaid/Medicare
   4. State budget or block grant funds
   5. Federal compliance grant (SAMHSA)
   6. State grant funding
   7. Grant from a private foundation
   8. Local fundraising drive(s)
   9. Users pay fee for service
   10. Volunteer or in-kind efforts used
   11. Other, specify: _______________________________________________________________________
3. What sources of funding or other resources do you now use to continue the activities or services that you started during the A-CRA grant?
   1. Internal funding from agency's budget
   2. Private health insurance
   3. Medicaid/Medicare
   4. State budget or block grant funds
   5. Federal compliance grant (SAMHSA)
   6. State grant funding
   7. Grant from a private foundation
   8. Local fundraising drive(s)
   9. Users pay fee for service
   10. Volunteer or in-kind efforts used
   11. Other, specify: _______________________________________________________________________
4. What is your best estimate of the current annual budget for your outpatient youth substance use treatment program (within $25,000 or so)?
5. Over the past 6 months, approximately how many youth received substance use treatment at your agency?
6. Approximately how many youth received A-CRA over the past 6 months?
7. Currently, how many clinicians at your agency treat youth with substance use problems?

16A. Can you tell me their names?

1. Currently, how many of the clinicians at your agency have been certified in A-CRA?
2. How many clinicians deliver A-CRA at your agency?

18A. Can you tell me their names?

***IF THE R CANNOT ANSWER THESE QUESTIONS UP TO THIS POINT, THANK THEM FOR HIS/HER TIME AND TRY TO IDENTIFY ANOTHER PERSON AT THE AGENCY WHO MAY BE BETTER TO ASK THESE QUESTIONS.***

***IF CURRENTLY FUNDED, GO TO QUESTION 21; OTHERWISE CONTINUE.***

1. While you were funded by SAMHSA/CSAT, were there other youth treatment programs in your local area?

🞏 No – ***GO TO QUESTION 21***
🞏 Yes – ***CONTINUE***

🞏 Don’t know – ***GO TO QUESTION 21***

1. Do/did they serve similar youth as your program?

🞏 No – PLEASE DESCRIBE HOW DIFFERENT: __________________
🞏 Yes

🞏 Don’t know

1. Currently, are there other youth treatment programs in your local area?

🞏 No – ***GO TO QUESTION 23***
🞏 Yes – ***CONTINUE***

🞏 Don’t know – ***GO TO QUESTION 23***

1. Do/did they serve similar youth as your program?

🞏 No – PLEASE DESCRIBE HOW DIFFERENT: __________________
🞏 Yes

🞏 Don’t know

1. Tell me about any ways in which your organization collaborates with academic or research institutions (colleges, universities).

**Treatment programs may have an easier time implementing a new treatment if there are a number of elements in place to support them. For this next section, we want to ask you about things that could affect A-CRA delivery at your organization.**

1. First, we want to ask you about planning. Tell me about any planning that has been done to ensure resources are available to continue A-CRA. By resources, we mean things needed to support A-CRA. For example, this could include things like money, staff, supervision, training, and A-CRA manuals.
2. Can you please describe any policies that support A-CRA delivery? These could include external policies, such as funding source and billing rules that support A-CRA.
3. What about policies that interfere with A-CRA delivery?

***IF CURRENTLY FUNDED CONTINUE THROUGH QUESTION 29, THEN, GO TO QUESTION 30; IF NOT CURRENLTY FUNDED GO TO THE “OTHERWISE VERSION.”***

**Next, I’d like to ask you about your agency’s plan to continue A-CRA clinical supervision support.**

1. First, does your agency have the finances to support the salary of a Clinical Supervisor?

🞏 No – ***GO TO QUESTION 30***
🞏 Yes

🞏 Don’t know

1. Does your agency plan to support Clinical Supervisor and Counselor time for biweekly supervision?

🞏 No
🞏 Yes – Describe, Explain:

1. Does your agency plan to support the Clinical Supervisor to listen to recorded therapy sessions and provide individualized feedback to counselors?

🞏 No
🞏 Yes – Describe, Explain:

***“OTHERWISE” VERSION— ASK IF NOT CURRENTLY FUNDED:***

**Next, I’d like to ask you about your agency’s A-CRA clinical supervision support over the last six months.**

27A. First, did your agency have the finances to support the salary of a Clinical Supervisor?

🞏 No – ***GO TO QUESTION 30***
🞏 Yes

🞏 Don’t know

28A. Second, did your agency support Clinical Supervisor and Counselor time for biweekly supervision?

🞏 No
🞏 Yes – Describe, Explain:

29A. Third, did your agency support the Clinical Supervisor to listen to recorded therapy sessions and provide individualized feedback to counselors?

🞏 No
🞏 Yes – Describe, Explain:

1. Does A-CRA meet the needs of the populations you serve? How so or why not?
2. Can you tell me about any pressure your organization experienced to continue delivering A-CRA or to discontinue its use?
3. Would you say staff are supportive or reluctant to use A-CRA? Can you give me an example?
4. What do you think is the most important influence on A-CRA’s success at your organization?
5. In the past 6 months, how have you or others at your agency disseminated information about your A-CRA project? By disseminated, we mean things like presentations, writing articles, media coverage, teaching, blogging, tweeting, etc.
   1. Presented to a local or state-level professional audience.
   2. Presented to a national or international professional audience.
   3. Wrote article(s) that was published or accepted for publication in a peer reviewed journal.
   4. Wrote article(s) that is under review or revision for a peer reviewed journal.
   5. Wrote article(s) for a newsletter or brochure or website.
   6. Had article(s) published in a local or state newspaper or on the internet.
   7. Received local or regional media coverage -- including print, television, radio broadcast, internet.
   8. Received national media coverage -- including print (NY Times, USA Today, Time, Newsweek, etc.), television or radio or internet
   9. Used information or materials from the project in teaching a college-level course.
   10. Developed a "how-to" manual or training package.
   11. Provided technical assistance to other agencies trying to implement similar projects.
   12. Wrote a blog about the project.
   13. Presented project on a website.
   14. Tweeted about the Project.
   15. None of the above.
   16. Other (please specify).
6. **[ONLY ASK FOR THOSE WHO FUNDING HAS ENDED]** What helped your efforts to sustain your project after the CSAT funding ended?
   1. Active support from your agency's executives or administrators.
   2. Actions by your agency's Board members.
   3. Program has become essential to carrying out the mission of your agency.
   4. Actions of an internal "champion" or key leader.
   5. Agency has existing "capacity" (e.g. enough staff member, skills, resources) to continue the program.
   6. You were able to modify the program to fit within your other programming.
   7. It is low cost - did not need substantial resources to continue.
   8. You have internal support for writing new grant proposals that helped obtain new resources.
   9. You had outcome/evaluation data that helped convince potential funders of the value of this program.
   10. Current or previous clients served as advocates for the program.
   11. Your staff members believed in the program so much they would not let it die.
   12. Partnerships with other organizations helped you find new funding sources.
   13. Your grant partners provide in-kind or other resources for continuation.
   14. Technical assistance or guidance from an external agency.
   15. Other helpful influence (please specify):
7. **[ONLY ASK FOR THOSE WHO FUNDING HAS ENDED]** What barriers have you encountered in attempting to sustain the project activities or services after the end of its CSAT funding?
   1. Obtaining funding from external sources.
   2. Obtaining funding from agency's budget, or other internal sources.
   3. Obtaining support from agency administrators for continuing project activities.
   4. Project leader or other key project "champion" left the agency.
   5. Turnover among staff members delivering project services.
   6. Agency priorities changed; ACRA approach is no longer a priority.
   7. Agency priorities changed; serving youth is no longer a priority.
   8. Maintaining agreement among partners essential for continuing project activities.
   9. Lack of physical space, such as lease terminated, project offices used for a different purpose, etc.
   10. Equipment or facilities needed (such as computers) no longer available.
   11. Policy changes that were agreed on temporarily during project were not made permanent.
   12. Partner agencies did not do what they promised.
   13. Other barriers (please specify):

**I will now ask you about the clinicians at your agency.**

1. Do you supervise clinicians who treat youth for substance use problems?

🞏 No – ***GO TO QUESTION 39***
🞏 Yes – ***CONTINUE***

1. How many of these clinicians are currently delivering A-CRA?

**Next, I’ll ask you about the supervisors at your agency.**

1. How many staff supervise clinicians who treat youth with substance use problems?
2. Do you supervise clinicians who deliver A-CRA?

🞏 No
🞏 Yes

🞏 Don’t know

1. How many others at your agency supervise clinicians delivering A-CRA?

🞏 Don’t know

1. Have you personally certified any clinicians in A-CRA?

🞏 No – ***GO TO QUESTION 59***
🞏 Yes – ***CONTINUE***

1. You mentioned that you personally had certified one or more clinicians in A-CRA at your organization. Can you please describe the certification process to me?

The clinician was asked to:

🞏 Read the A-CRA manual

🞏 Take an online A-CRA research course

🞏 Pass an A-CRA quiz with a score of 80% or higher

🞏 Attend a Chestnut or RJM A-CRA initial training OR attend an in-house training

🞏 Participate in regular coaching calls with Chestnut or regular supervision with in-house Clinical Supervisor (respondent) during certification (regular= @ every other week)

🞏 Regularly record therapy sessions for in-house Clinical Supervisor (respondent) review/Chestnut’s review (regular= at least some sessions weekly))

🞏 Demonstrate competency in General Clinical Skills on the DSRs

🞏 Demonstrate competency in the following A-CRA procedures:

Functional Analysis of Use

Functional Analysis of Pro-social behavior

Happiness Scale

Treatment Plan/Goals of Counseling

Communication Skills

Problem Solving Skills

Adolescent-Caregiver Relationship Skills, and

Homework based of 3 or better on all components of a given procedure using the A-CRA rating manual?

If in-house training was provided, did it?

🞏 Include didactic information about A-CRA procedures?

🞏 Modeling or review of audio recordings of procedures that were well done

🞏 The opportunity to role play procedures

🞏 Other, please explain_____________________________________

**Next, I’m going to ask you a series of questions about how the certification process currently works in your agency. Please respond by saying “True,” “False,” or “Don’t Know” if you are unsure.**

1. When I decide to pass a clinician on a procedure it is based on ratings of 1 or more on every component of a procedure. Remember that each component of a procedure is rated on a 1 to 5 scale.

🞏 False
🞏 True
🞏 Don’t Know

1. Communication skills is a procedure that people have to pass to attain certification.

🞏 False
🞏 True
🞏 Don’t Know

1. I review recorded sessions during the certification process.

🞏 False
🞏 True
🞏 Don’t Know

1. I am required to sit in sessions with clinicians during the certification process.

🞏 False
🞏 True
🞏 Don’t Know

1. Clinicians record one or two of their sessions.

🞏 False
🞏 True
🞏 Don’t Know

1. I refer to the A-CRA rating manual when rating session recordings.

🞏 False
🞏 True
🞏 Don’t Know

1. Clinicians are required to take a knowledge test as part of the certification process.

🞏 False
🞏 True
🞏 Don’t Know

1. Clinicians are not required to pass General Clinical Skills as part of the certification process.

🞏 False
🞏 True
🞏 Don’t Know

1. Each clinician has a certification workbook.

🞏 False
🞏 True
🞏 Don’t Know

1. I complete the A-CRA checklist when I am listening to a recorded session during or after the certification process.

🞏 False
🞏 True
🞏 Don’t Know

1. Clinicians are required to read the A-CRA manual during the training process.

🞏 False
🞏 True
🞏 Don’t Know

1. Time is set aside for training clinicians in A-CRA

🞏 False
🞏 True
🞏 Don’t Know

1. During training clinicians are required to practice procedures with role-plays.

🞏 False
 🞏 True
 🞏 Don’t Know

1. Adolescent-Caregiver Relationship Skills is one of the procedures for basic certification.

🞏 False
🞏 True
🞏 Don’t Know

1. It doesn't matter if clinicians show competency in all of the additional procedures as well.

🞏 False
🞏 True
🞏 Don’t Know

1. On average, approximately how many session recordings do clinicians at your site complete to reach basic certification? Your best guess is fine.

**Now, I’d like to ask you about A-CRA training.**

1. How many new clinicians have been trained in A-CRA in the past six-months in the organization?
2. How many new clinicians have been trained in A-CRA in the past six-months at a Chestnut or Robert J. Meyers training?
3. Are there training agendas for your trainings?

🞏 No – ***GO TO QUESTION 64***
🞏 Yes – ***CONTINUE***

1. Will you please send me (email, mail, fax) a copy of the agenda?

🞏 No
🞏 Yes

**Next, I want to ask you about your general impressions regarding the SAMHSA CSAT-funded A-CRA project and then have a few questions about you.**

1. If you had a chance to participate in a SAMHSA CSAT project again, would you consider it?

🞏 No
🞏 Yes

1. Why/why not?
2. Is there anything you would change about the SAMHSA CSAT project in order to improve the sustainment of A-CRA at your agency?
3. Is there anything else you think would have helped to sustain the use of A-CRA at your agency?
4. How many years of experience do you have as a clinical supervisor for substance use counselors/clinicians?

# of years ____________ (OR) # of months ___________

1. How many years of experience do you have in substance use counseling as a clinician?

# of years ____________ (OR) # of months ___________

1. Do you have any experience as an administrator of youth substance use treatment programs?

🞏 No – ***GO TO QUESTION 72***
🞏 Yes – ***CONTINUE***

1. How many years of experience as an administrator do you have?

# of years ____________ (OR) # of months ___________

1. Please provide the full name (as opposed to only the acronym) of all Licensures and/or Certifications you currently hold.

**END SCRIPT:**

We are now done with the interview. Here’s what happens next:

- In about a week, you will receive an e-mail from RAND with the link to a web survey and it will include your personal password. You can do it any date/time convenient for you, but please try to complete it within 2-3 weeks. At the last screen of the web survey, we will ask you for your contact information for your $50 honorarium check. We will only use that to send your check and it will not be attached to your survey.
- You will also receive a separate email with instructions on how to submit your 3 sample therapy recordings, in which you will receive another $50 and should try to do within the next month. The email will have detailed instructions and a consent form that needs to be completed for each client. ***These recordings should be of NEWLY recorded sessions, not previously recorded sessions.***  One of our project staff will contact you in a [few days (if did not acknowledge)/couple of weeks (if acknowledged)] to check to see if you have any questions or need any assistance. I know you are very busy and we appreciate you participating in this ALSO very critical part of the study to help understand youth treatment program delivery.

[IF R ONLY DOES GROUP THERAPY: We prefer that the recordings be for individual therapy sessions, but if you only do group sessions, please make sure you obtain consent for everyone and place the recorder next to you].

Can you confirm your e-mail address? _____________________________________

**Non-Sustainer Interview – SUPERVISOR**

1. Based on an initial call, it sounded like your agency currently does not deliver the Adolescent Community Reinforcement Approach or A-CRA anymore. Is this correct?

🞏 No – ***SWITCH TO SUSTAINER INTERVIEW***🞏 Yes – ***CONTINUE***

1. Tell me a little bit about your agency.
2. What types of clients do you serve? (e.g., age range, gender, problem types).
3. What is your primary professional role(s) at your clinic/site?

🞏 Supervisor – ***CONTINUE***

🞏 Counselor or Clinician – ***SWITCH TO CLINICIAN VERSION***
🞏 Supervisor and Counselor/Clinician ***– SWITCH TO SUP/CLIN VERSION***

1. Did you work on any of the following SAMHSA/CSAT A-CRA funded projects that your agency was awarded from the following dates?

|  | \| **START:** \|  \| \| --- \| --- \| \|  \|  \| \| **END:** \|  \| |
| --- | --- | --- | --- | --- | --- | --- | --- |
|  | \| **START:** \|  \| \| --- \| --- \| \|  \|  \| \| **END:** \|  \| |
|  | \| **START:** \|  \| \| --- \| --- \| \|  \|  \| \| **END:** \|  \| |
|  | \| **START:** \|  \| \| --- \| --- \| \|  \|  \| \| **END:** \|  \| |

🞏 No **– *SKIP TO QUESTION 7***🞏 Yes **– *CONTINUE***

1. Between what dates did you work on the project? Your best guess is fine.

Initiative Start Date End Date

|  | \| Month/Year: \|  \| \| --- \| --- \| \|  \|  \| | \| Month/Year: \|  \| \| --- \| --- \| |
| --- | --- | --- | --- | --- | --- | --- | --- | --- |
|  | \| Month/Year: \|  \| \| --- \| --- \| \|  \|  \| | \| Month/Year: \|  \| \| --- \| --- \| |
|  | \| Month/Year: \|  \| \| --- \| --- \| \|  \|  \| | \| Month/Year: \|  \| \| --- \| --- \| |
|  | \| Month/Year: \|  \| \| --- \| --- \| \|  \|  \| | \| Month/Year: \|  \| \| --- \| --- \| |

1. What role(s) did you serve while A-CRA was being delivered at your agency? *[MARK ALL THAT APPLY]*

🞏 A-CRA Therapist
🞏 A-CRA Supervisor
🞏 Other (Please describe: ____________________________________________________)
🞏 No Role in delivering ACRA

1. What do you think of A-CRA as a treatment for youth? Please note that throughout this interview, by “youth” we mean the population served during your SAMHSA CSAT grant periods.
2. When did you/your agency stop delivering A-CRA?

**Month/Year: ___________/_____________**

1. Approximately how many youth received A-CRA during the six-month period, right before A-CRA treatment delivery ended?
2. What were the main reasons you/your agency stopped delivering A-CRA?
3. What would have increased your desire to continue delivering A-CRA?
4. What would have increased your ability to continue delivering A-CRA?
5. Does your program currently offer any different types of treatment for youth with substance use disorders besides A-CRA?

🞏 No – ***SKIP TO QUESTION 16***🞏 Yes – ***CONTINUE***🞏 Don’t Know – ***SKIP TO QUESTION 16***

1. What are those treatments called? [Or describe the treatment. Is it outpatient? How long does it last? # of sessions/days/months] *[MARK ALL THAT APPLY]*
   1. Motivational Enhancement Therapy (MET)
   2. Cognitive Behavioral Therapy (CBT)
   3. MET/CBT-5
   4. Matrix Model
   5. Multidimensional Family Therapy (MDFT)
   6. Multisystemic Therapy (MST)
   7. 12-step facilitation
   8. Psychoeducation
   9. Supportive counseling
   10. Other____________________________________________________________
2. Currently, what sources of funding or other resources do you use to support youth treatment delivery?
   1. Internal funding from agency's budget
   2. Private health insurance
   3. Medicaid/Medicare
   4. State budget or block grant funds
   5. Federal compliance grant (SAMHSA)
   6. State grant funding
   7. Grant from a private foundation
   8. Local fundraising drive(s)
   9. Users pay fee for service
   10. Volunteer or in-kind efforts used
   11. Other, specify: _______________________________________________________________________
3. What is your best estimate of the current annual budget for your outpatient youth substance use treatment program (within $25,000 or so)?
4. Over the past 6 months, approximately how many youth received substance use treatment at your agency?

18B. Approximately how many youth received A-CRA over the past 6 months?

1. Currently, how many clinicians at your agency treat youth with substance use problems?

19A. Can you tell me their names?

1. Currently, how many of the clinicians at your agency have been certified in A-CRA?

20A. Can you tell me their names?

1. While you were funded by SAMHSA/CSAT, were there other youth treatment programs in your local area?

🞏 No – ***GO TO QUESTION 23***
🞏 Yes – ***CONTINUE***

🞏 Don’t know – ***GO TO QUESTION 23***

1. Do/did they serve similar youth as your program?

🞏 No – PLEASE DESCRIBE HOW DIFFERENT: __________________
🞏 Yes

🞏 Don’t know

1. Currently, are there other youth treatment programs in your local area?

🞏 No – ***GO TO QUESTION 25***
🞏 Yes – ***CONTINUE***

🞏 Don’t know – ***GO TO QUESTION 25***

1. Do/did they serve similar youth as your program?

🞏 No – PLEASE DESCRIBE HOW DIFFERENT: __________________
🞏 Yes

🞏 Don’t know

1. Tell me about any ways in which your organization collaborates with academic or research institutions (colleges, universities).

**Treatment programs may have an easier time implementing a new treatment if there are a number of elements in place to support them. For this next section, we want to ask you about things that could have affected A-CRA delivery at your present organization during the A-CRA funding period.**

1. First, we want to ask you about planning. Was there any strategic planning done to ensure resources were available to continue A-CRA beyond the initial funding period? By resources, we mean things needed to support A-CRA. For example, this could include things like money, staff, supervision, training, and A-CRA manuals.
2. Can you please describe any policies that supported A-CRA delivery? These could include external policies, such as funding source and billing rules that supported A-CRA.
3. What about policies that interfered with A-CRA delivery?

**Next, I’d like to ask you about your agency’s A-CRA clinical supervision support during the last six months it was delivered.**

1. First, did your agency have the finances to support the salary of a Clinical Supervisor?

🞏 No – ***GO TO QUESTION 32***
🞏 Yes

🞏 Don’t know

1. Second, did your agency support Clinical Supervisor and Counselor time for biweekly supervision?

🞏 No
🞏 Yes – Describe, Explain:

1. Third, did your agency support the Clinical Supervisor to listen to recorded therapy sessions and provide individualized feedback to counselors?

🞏 No
🞏 Yes – Describe, Explain:

1. Did A-CRA meet the needs of the populations you serve? How so or why not?
2. Can you tell me about any pressure your organization experienced to continue delivering A-CRA or to discontinue its use?
3. Would you say staff were supportive or reluctant to use A-CRA? Can you give me an example?
4. What do you think is the most important influence on the discontinuation of A-CRA at your organization?
5. In the 6 months prior to ending A-CRA, how did you or others at your agency disseminate information about your A-CRA project? By disseminated, we mean things like presentations, writing articles, media coverage, teaching, blogging, tweeting, etc.
   1. Presented to a local or state-level professional audience.
   2. Presented to a national or international professional audience.
   3. Wrote article(s) that was published or accepted for publication in a peer reviewed journal.
   4. Wrote article(s) that is under review or revision for a peer reviewed journal.
   5. Wrote article(s) for a newsletter or brochure or website.
   6. Had article(s) published in a local or state newspaper or on the internet.
   7. Received local or regional media coverage -- including print, television, radio broadcast, internet.
   8. Received national media coverage -- including print (NY Times, USA Today, Time, Newsweek, etc.), television or radio or internet
   9. Used information or materials from the project in teaching a college-level course.
   10. Developed a "how-to" manual or training package.
   11. Provided technical assistance to other agencies trying to implement similar projects.
   12. Wrote a blog about the project.
   13. Presented project on a website.
   14. Tweeted about the Project.
   15. None of the above.
   16. Other (please specify):
6. Before your organization made the decision not to sustain A-CRA, can you tell me about any efforts made to sustain after the end of its CSAT funding?
   1. Active support from your agency's executives or administrators.
   2. Actions by your agency's Board members.
   3. Program has become essential to carrying out the mission of your agency.
   4. Actions of an internal "champion" or key leader.
   5. Agency has existing "capacity" (e.g. enough staff member, skills, resources) to continue the program.
   6. You were able to modify the program to fit within your other programming.
   7. It is low cost - did not need substantial resources to continue.
   8. You have internal support for writing new grant proposals that helped obtain new resources.
   9. You had outcome/evaluation data that helped convince potential funders of the value of this program.
   10. Current or previous clients served as advocates for the program.
   11. Your staff members believed in the program so much they would not let it die.
   12. Partnerships with other organizations helped you find new funding sources.
   13. Your grant partners provide in-kind or other resources for continuation.
   14. Technical assistance or guidance from an external agency.
   15. Other helpful influence (please specify):
7. What barriers were encountered in attempting to sustain the project activities or services after the end of its CSAT funding?
   1. Obtaining funding from external sources.
   2. Obtaining funding from agency's budget, or other internal sources.
   3. Obtaining support from agency administrators for continuing project activities.
   4. Project leader or other key project "champion" left the agency.
   5. Turnover among staff members delivering project services.
   6. Agency priorities changed; ACRA approach is no longer a priority.
   7. Agency priorities changed; serving youth is no longer a priority.
   8. Maintaining agreement among partners essential for continuing project activities.
   9. Lack of physical space, such as lease terminated, project offices used for a different purpose, etc.
   10. Equipment or facilities needed (such as computers) no longer available.
   11. Policy changes that were agreed on temporarily during project were not made permanent.
   12. Partner agencies did not do what they promised.
   13. Other barriers (please specify):

**Now I’m going to ask you a few questions about each of the clinicians who treat youth for substance use problems at your agency.**

1. Do you currently supervise clinicians who treat youth for substance use problems?

🞏 No
🞏 Yes

**Thank you. I now have some questions about the supervisors of clinicians delivering youth treatment at your agency.**

1. How many staff supervise clinicians who treat youth with substance use problems?
2. Did you supervise clinicians who deliver A-CRA?

🞏 No
🞏 Yes

🞏 Don’t know

1. How many others at your agency supervised clinicians delivering A-CRA?

🞏 Don’t know

1. Have you personally certified any clinicians in A-CRA?

🞏 No – ***GO TO QUESTION 60***
🞏 Yes – ***CONTINUE***

1. You mentioned that you personally had certified one or more clinicians in A-CRA at your organization. Can you please describe the certification process to me?

The clinician was asked to:

🞏 Read the A-CRA manual

🞏 Take an online A-CRA research course

🞏 Pass an A-CRA quiz with a score of 80% or higher

🞏 Attend a Chestnut or RJM A-CRA initial training OR attend an in-house training

🞏 Participate in regular coaching calls with Chestnut or regular supervision with in-house Clinical Supervisor (respondent) during certification (regular= @ every other week)

🞏 Regularly record therapy sessions for in-house Clinical Supervisor (respondent) review/Chestnut’s review (regular= at least some sessions weekly))

🞏 Demonstrate competency in General Clinical Skills on the DSRs

🞏 Demonstrate competency in the following A-CRA procedures:

Functional Analysis of Use

Functional Analysis of Pro-social behavior

Happiness Scale

Treatment Plan/Goals of Counseling

Communication Skills

Problem Solving Skills

Adolescent-Caregiver Relationship Skills, and

Homework based of 3 or better on all components of a given procedure using the A-CRA rating manual?

If in-house training was provided, did it?

🞏 Include didactic information about A-CRA procedures?

🞏 Modeling or review of audio recordings of procedures that were well done

🞏 The opportunity to role play procedures

🞏 Other, please explain_____________________________________

**Next, I’m going to ask you a series of questions about how the A-CRA certification process worked in your agency. Please respond by saying “True,” “False,” or “Don’t Know” if you are unsure.**

1. When I decided to pass a clinician on a procedure it was based on ratings of 1 or more on every component of a procedure. Remember that each component of a procedure is rated on a 1 to 5 scale.

🞏 False
🞏 True
🞏 Don’t Know

1. Communication skills was a procedure that people had to pass to attain certification.

🞏 False
🞏 True
🞏 Don’t Know

1. I reviewed recorded sessions during the certification process.

🞏 False
🞏 True
🞏 Don’t Know

1. I was required to sit in sessions with clinicians during the certification process.

🞏 False
🞏 True
🞏 Don’t Know

1. Clinicians recorded one or two of their sessions.

🞏 False
🞏 True
🞏 Don’t Know

1. I referred to the A-CRA rating manual when rating session recordings.

🞏 False
🞏 True
🞏 Don’t Know

1. Clinicians were required to take a knowledge test as part of the certification process.

🞏 False
🞏 True
🞏 Don’t Know

1. Clinicians were not required to pass General Clinical Skills as part of the certification process.

🞏 False
🞏 True
🞏 Don’t Know

1. Each clinician had a certification workbook.

🞏 False
🞏 True
🞏 Don’t Know

1. I completed the A-CRA checklist when I was listening to a recorded session during or after the certification process.

🞏 False
🞏 True
🞏 Don’t Know

1. Clinicians were required to read the A-CRA manual during the training process.

🞏 False
🞏 True
🞏 Don’t Know

1. Time was set aside for training clinicians in A-CRA

🞏 False
🞏 True
🞏 Don’t Know

1. During training clinicians were required to practice procedures with role-plays.

🞏 False
 🞏 True
 🞏 Don’t Know

1. Adolescent-Caregiver Relationship Skills was one of the procedures for basic certification.

🞏 False
🞏 True
🞏 Don’t Know

1. It didn’t matter if clinicians show competency in all of the additional procedures as well.

🞏 False
🞏 True
🞏 Don’t Know

1. On average, approximately how many session recordings did clinicians at your site complete to reach basic certification? Your best guess is fine.

**Next, I want to ask you about your general impressions regarding the SAMHSA CSAT-funded A-CRA project and then have a few questions about you.**

1. If you had a chance to participate in a SAMHSA CSAT project again, would you consider it?

🞏 No
🞏 Yes

1. Why/why not?
2. Is there anything you would change about the SAMHSA CSAT project in order to improve the sustainment of A-CRA at your agency?
3. Is there anything else you think would have helped to sustain the use of A-CRA at your agency?
4. How many years of experience do you have as a clinical supervisor for substance use counselors/clinicians?

# of years ____________ (OR) # of months ___________

1. How many years of experience do you have in substance use counseling as a clinician?
2. Do you have any experience as an administrator of youth substance use treatment programs?

🞏 No – ***GO TO QUESTION 69***
🞏 Yes – ***CONTINUE***

1. How many years of experience as an administrator do you have?

# of years ____________ (OR) # of months ___________

1. Please provide the full name (as opposed to only the acronym) of all Licensures and/or Certifications you currently hold.

**END SCRIPT:**

We are now done with the interview. Here’s what happens next:

- In about a week, you will receive an e-mail from RAND with the link to a web survey and it will include your personal password. You can do it any date/time convenient for you, but please try to complete it within 2-3 weeks. At the last screen of the web survey, we will ask you for your contact information for your $50 honorarium check. We will only use that to send your check and it will not be attached to your survey.
- You will also receive a separate email with instructions on how to submit your 3 sample therapy recordings, in which you will receive another $50 and should try to do within the next month. The email will have detailed instructions and a consent form that needs to be completed for each client. ***These recordings should be of NEWLY recorded sessions, not previously recorded sessions.***  One of our project staff will contact you in a [few days (if did not acknowledge)/couple of weeks (if acknowledged)] to check to see if you have any questions or need any assistance. I know you are very busy and we appreciate you participating in this ALSO very critical part of the study to help understand youth treatment program delivery.

[IF R ONLY DOES GROUP THERAPY: We prefer that the recordings be for individual therapy sessions, but if you only do group sessions, please make sure you obtain consent for everyone and place the recorder next to you].

Can you confirm your e-mail address? _____________________________________
