# Supplementary material for: Comparing organization-focused and state-focused financing strategies on provider-level reach of a youth substance use treatment model: a mixed-method study
Source: Implement Sci. 2023 Oct 12;18:50. doi: 10.1186/s13012-023-01305-z (PMC10571404; doi:10.1186/s13012-023-01305-z)
Supplement: Supplementary file 3 — Additional file 3. All Wave 1 Survey Items. [file 13012_2023_1305_MOESM3_ESM.docx]

**STATE-FOCUSED**

**Welcome to the RAND Youth Treatment Study Web Survey!**

Thank you again for your participation. The survey will take you about 30 minutes to complete. The purpose of the survey is to understand how different CSAT funding models influence the sustainability of A-CRA delivery after initial funding from CSAT ends.

After you complete this survey, you will receive a $50 Amazon e-gift card for your participation in the interview and survey. We understand you are extremely busy and appreciate your time.

To participate in the survey, please click the "Start" button.

**Consent**

You are invited to complete a web survey because you are a clinician and/or supervisor (or other person with comparable knowledge) at an organization that received training in the Adolescent Community Reinforcement Approach (A-CRA), as part of a SAMHSA Center of Substance Abuse Treatment (CSAT) grant to your state. The survey is part of a National Institutes of Health (NIH)-funded research project that examines the experiences of A-CRA programs during and after CSAT funding.

***Who is conducting the research project?***

The RAND Corporation, a non-profit research institute, is conducting this research project in collaboration with Chestnut Health Systems. The research is overseen by the RAND Human Subjects Protection Committee. The research is funded by the National Institute of Drug Abuse (NIDA), which is part of the NIH.

***What is the purpose of the research?***

The purpose is to understand how different CSAT funding models influence the sustainability of A-CRA delivery after initial funding from CSAT ends. The results will inform efforts to promote sustainability of A-CRA and other evidence-based treatments, to ultimately improve the quality of care for adolescents with problematic substance use.

***What will I be asked to do?***

Your participation will involve completing a 30-minute online survey that asks questions about the leadership, staff, partnerships, and culture of your organization and its youth substance use treatment services; your implementation of A-CRA, and factors that may have affected its success; and whether you are still delivering A-CRA.

***Do I have to participate in the research project?***

No. Your participation in the survey is voluntary. If you do participate, you will be able to skip questions that you prefer not to answer.

***What will you do with the information I provide?***

The information you provide in the survey will be combined with interview and survey responses of clinicians and supervisors from nearly 20 states across the country that CSAT funded to implement A-CRA. We will also examine information provided by state organizations that administered CSAT grants like yours and data already collected from prior CSAT grantee organizations that implemented A-CRA. Results from the combined dataset will be published in journal articles that will be publicly available and will also be shared with U.S. federal agencies and centers (such as CSAT). However, we will never share your individual survey responses.

***What are the risks of participating, and how is the information I provide protected?***

No physical risks are involved in participating. The primary risk is unauthorized disclosure of your answers, but we have taken several steps to minimize this risk: All staff have been trained to understand the need for privacy. The information about your identity and all the data with your answers are kept in two separate files that are only linked through an anonymous research ID. This project’s records are also stored in locked, secure research offices on secure, encrypted computers and servers. Your individual responses will not be shared with anyone outside of the research team (this means no one at your organization or CSAT will see your responses). All data will be analyzed in aggregate form only, and no individual or program will be identifiable in any scientific report prepared by the research team. Although we request that you answer all items as honestly as you can, you may skip over items you do not wish to answer.

We will not attribute information to specific individuals or programs.

***What are the benefits of participation in the research project?***

After completion of the web survey, you will receive a $50 Amazon e-gift card for your participation in the interview and survey. In addition, your participation will help to better understand youth substance use treatment funding and delivery, which can improve the quality of care for adolescents who engage in problematic substance use.

***Who can I contact if I have questions?***

If you have any questions or want to discuss the project further at any time, you may always contact [NAME] at [PHONE] or [EMAIL].

You can also contact the lead researcher of the project directly at:

Alex R. Dopp, Ph.D.
Lead Researcher, RAND Youth Treatment Study
RAND Corporation
1776 Main Street, Santa Monica, CA 90401
Phone: (310) 393-0411 x7611
Email: [adopp@rand.org](mailto:adopp@rand.org)

Furthermore, if you have questions about your rights as a research participant or need to report a research-related injury or concern, you can contact RAND's Human Subjects Protection Committee toll-free at (866) 697-5620 or email hspcinfo@rand.org. If you contact the Committee, please reference Study #2020-N0887.

To participate in the survey, please click the "Start" button.

**Sustainer Survey**

All sustainers will have a ADMIN_ACRA = 1.

**The first set of questions are about A-CRA. Please select the correct answer for each of the following:**

1. The basic premise of CRA is that:
   1. with adequate skills training an individual can really combat any substance use problem.
   2. although will-power is ultimately still responsible for the majority of behavior change, at least a commitment to sobriety is also required.
   3. one must make a non-drinking/using lifestyle as rewarding as a drinking/using lifestyle.
   4. once an individual has a good job and the family is supportive again, the other pieces naturally fall into place.
   5. Don't know
2. A simple and easy way to monitor a youth's own report of progress in a number of areas is through:
   1. a behavioral contract.
   2. the CRA Happiness Scale.
   3. the CRA Functional Analysis for Non-Drinking Behaviors.
   4. the Self-Reminder To Be Nice form.
   5. Don't know
3. Why is it so important for a CRA therapist to always be looking for a youth's reinforcers?
   1. so that the therapist can be aware of the size of the youth's social support system at all times.
   2. because these may need to be resorted to and incorporated into the treatment plan if mild punishment does not appear to be working.
   3. because youths will be more likely to change their behavior if they feel they are being rewarded in doing so.
   4. because Community Reinforcement Approach implies that reinforcers can usually only be identified with the assistance of the youth's community.
   5. Don't know
4. Most behavioral and cognitive-behavioral treatments rely heavily on role-plays, despite the fact that it is often uncomfortable for clients (and therapists!) to do them. Role-plays are considered important because:
   1. they provide the therapist with valuable information about a youth's level of resistance to treatment.
   2. they are good for practice, because it is actually more difficult for a youth to do a role-play during a session than it is for them to try out the interaction in the natural environment.
   3. they offer valuable information about the quality of the therapeutic relationship and are early indicators of problematic transference.
   4. they give youths the opportunity to practice their new skills in a supportive environment with a person who can provide feedback.
   5. Don't know
5. The A-CRA Functional Analysis for Substance Use chart looks at the youth's short-term positive consequences for substance use. The reason for looking at these is:
   1. to be sure that the youth is fully aware from the start of what they are giving up by becoming substance free, and to be agreeable to it.
   2. to motivate the youth by reminding them of all the hardships endured (by the youth and their family) as a result of their use.
   3. to see what role substance use is serving, so that the individual can later be taught healthier ways to obtain these things.
   4. to recognize the types of obstacles that have successfully interfered with substance use in the past.
   5. Don't know
6. Why is it important to have both the youth and caregiver complete the Relationship Happiness Scale form during the Caregiver III and IV sessions?
   1. To enable the therapist to solve the families’ problems in one session.
   2. To get them to identify their weaknesses.
   3. To expose the source of unhappiness within the relationship.
   4. To have them practice communication and problem-solving skills through role-plays.
   5. Don't know
7. One CRA procedure entails: getting a client to role-play a phone call in session to an organization of interest (e.g., N.A.), and then having them actually place the call during the session, locating a contact person in the community for N.A., and calling them, and reviewing in the next session the experience of attending an N.A. meeting. The name of this procedure is:
   1. systematic encouragement
   2. reinforcer sampling
   3. cognitive restructuring
   4. communication skills training
   5. Don't know
8. Which of the following is not one of the common mistakes made by therapists who are implementing CRA? (each of the others is a common mistake):
   1. losing sight of the youth's reinforcers.
   2. not emphasizing the importance of having a satisfying job.
   3. neglecting to monitor the youth's drinking and drug use.
   4. not checking for generalizability of skills.
   5. Don't know
9. In first selecting a category from the Happiness Scale to work on with a youth, it is advisable to begin with:
   1. an area that the youth express extreme unhappiness with, thereby demonstrating that you do not intend to shy away from the really difficult problems.
   2. an area that the youth states is really the main reason for seeking treatment in the first place, and consequently is the area most important to them.
   3. an area that is broad and really includes most of the youth's drug and non-drug problems, so that many different important issues get addressed right from the start.
   4. an area that the youth has reported mild-moderate (not extreme) unhappiness in, since it will probably provide an opportunity for the youth to experience some early success in therapy.
   5. Don't know
10. Therapists often spend very little time discussing a youth's social/recreational activities. However, it is important to do so because:
    1. many youths' social lives are built around alcohol/drugs, so they'll need help developing new drug-free pleasurable activities that compete with substance use.
    2. youth will assume that if you do not discuss this part of their lives, then they can probably get away with having an occasional beer during their recreational activities.
    3. even though a youth's social life is not particularly important to discuss from a therapeutic sense, it gives out the valuable message that you care enough about the youth to do it anyway.
    4. youths' behavior during their free time is really the best indicator we have of their personal values, and consequently it is often a predictor of whether they will remain committed to changing their using behavior.
    5. Don't know

**The following questions ask about A-CRA in your organization. Please indicate the extent to which you agree with the following items:**

1. Staff use A-CRA as much as possible when appropriate
   1. Not at all
   2. To a slight extent
   3. To a moderate extent
   4. To a great extent
   5. To a very great extent
2. Staff continue to use A-CRA throughout changing circumstances
   1. Not at all
   2. To a slight extent
   3. To a moderate extent
   4. To a great extent
   5. To a very great extent
3. A-CRA is a routine part of our practice
   1. Not at all
   2. To a slight extent
   3. To a moderate extent
   4. To a great extent
   5. To a very great extent
4. Please rate your level of agreement with the following statements, ranging from strongly disagree to strongly agree.

|  | Strongly Disagree (1) | Somewhat Disagree (2) | Neither Agree nor Disagree (3) | Somewhat Agree (4) | Strongly Agree (5) |
| --- | --- | --- | --- | --- | --- |
| In general, A-CRA is more effective in creating attitudes that discourage substance use by clients than other treatment practices. |  |  |  |  |  |
| A-CRA is more effective in reducing substance use by clients than our current treatment practices. |  |  |  |  |  |
| A-CRA improved the overall quality of substance use treatment in this agency. |  |  |  |  |  |
| A-CRA is better than our previous treatment practices for substance use. |  |  |  |  |  |
| A-CRA is difficult to teach. |  |  |  |  |  |
| A-CRA is hard for therapists to understand. |  |  |  |  |  |
| A-CRA requires complex therapeutic strategies. |  |  |  |  |  |

1. Please rate your level of agreement with the following statements, ranging from strongly disagree to strongly agree.

|  | Strongly Disagree (1) | Somewhat Disagree (2) | Neither Agree nor Disagree (3) | Somewhat Agree (4) | Strongly Agree (5) |
| --- | --- | --- | --- | --- | --- |
| There is a definite need for A-CRA among my agency's clientele |  |  |  |  |  |
| There is a high level of interest for A-CRA among my agency's clientele |  |  |  |  |  |
| A-CRA is timely |  |  |  |  |  |
| A-CRA helps build partnerships |  |  |  |  |  |
| A-CRA has an impact on participants |  |  |  |  |  |

1. Please rate your level of agreement with the following statements, ranging from strongly disagree to strongly agree.

|  | Strongly Disagree (1) | Somewhat Disagree (2) | Neither Agree nor Disagree (3) | Somewhat Agree (4) | Strongly Agree (5) |
| --- | --- | --- | --- | --- | --- |
| Financing A-CRA is difficult |  |  |  |  |  |
| Recruiting participants for A-CRA is difficult |  |  |  |  |  |
| Recruiting staff to work on A-CRA is difficult |  |  |  |  |  |
| Finding time to prepare for A-CRA is difficult |  |  |  |  |  |
| Staff working on A-CRA have to learn new skills to deliver it |  |  |  |  |  |

Do you agree or disagree:

1. Management wants to continue A-CRA
   1. Strongly Disagree
   2. Somewhat Disagree
   3. Neither Agree nor Disagree
   4. Somewhat Agree
   5. Strongly Agree
2. The following items ask about financial status of your service agency. Please indicate the extent to which you agree with each statement.

|  | Not at all (1) | Slight extent (2) | Moderate extent (3) | Great extent (4) | Very great extent (5) |
| --- | --- | --- | --- | --- | --- |
| Overall, this agency is in a state of financial distress. |  |  |  |  |  |
| The financial status of this agency has improved in recent years. |  |  |  |  |  |
| The ongoing financial viability of this agency is a major concern. |  |  |  |  |  |
| This agency is in a strong financial position. |  |  |  |  |  |
| One of this agency’s main goals is to manage its finances effectively. |  |  |  |  |  |
| People in this agency think that the organization’s financial health is important. |  |  |  |  |  |
| Being financially viable is a top priority in this agency. |  |  |  |  |  |
| Evidence-based practices are expensive. |  |  |  |  |  |
| Evidence-based practices offer many financial advantages to this agency. |  |  |  |  |  |
| Our agency has done a good job of finding adequate funding for evidence-based practices. |  |  |  |  |  |
| This agency would not implement evidence-based practices if they were not externally funded. |  |  |  |  |  |

1. For each statement in the next set of questions, select the number that best indicates the extent to which the A-CRA treatment program at your organization has or does the following things.

|  | 1 To little or no extent (1) | 2 (2) | 3 (3) | 4 (4) | 5 (5) | 6 (6) | 7 To a very great extent (7) | NA Not able to answer (8) |
| --- | --- | --- | --- | --- | --- | --- | --- | --- |
| Program champions advocate for the A-CRA treatment program. |  |  |  |  |  |  |  |  |
| The A-CRA treatment program has strong champions with the ability to garner resources. |  |  |  |  |  |  |  |  |
| The A-CRA treatment program has political support within the larger organization. |  |  |  |  |  |  |  |  |
| The A-CRA treatment program has political support from outside of the organization. |  |  |  |  |  |  |  |  |
| The A-CRA treatment program has strong advocacy support. |  |  |  |  |  |  |  |  |
| The A-CRA treatment program exists in a supportive state economic climate. |  |  |  |  |  |  |  |  |
| The A-CRA treatment program implements policies to help ensure sustained funding. |  |  |  |  |  |  |  |  |
| The A-CRA treatment program is funded through a variety of sources. |  |  |  |  |  |  |  |  |
| The A-CRA treatment program has a combination of stable and flexible funding. |  |  |  |  |  |  |  |  |
| The A-CRA treatment program has sustained funding. |  |  |  |  |  |  |  |  |

1. For each statement in the next set of questions, select the number that best indicates the extent to which the A-CRA treatment program at your organization has or does the following things.

|  | 1 To little or no extent (1) | 2 (2) | 3 (3) | 4 (4) | 5 (5) | 6 (6) | 7 To a very great extent (7) | NA Not able to answer (8) |
| --- | --- | --- | --- | --- | --- | --- | --- | --- |
| The A-CRA treatment program is well integrated into the operations of the organization. |  |  |  |  |  |  |  |  |
| Organizational systems are in place to support the various A-CRA treatment program needs. |  |  |  |  |  |  |  |  |
| Leadership effectively articulates the vision of the A-CRA treatment program to external partners. |  |  |  |  |  |  |  |  |
| Leadership efficiently manages staff and other resources. |  |  |  |  |  |  |  |  |
| The A-CRA treatment program has adequate staff to complete the program’s goals. |  |  |  |  |  |  |  |  |
| The A-CRA treatment program has the capacity for quality program evaluation. |  |  |  |  |  |  |  |  |
| The A-CRA treatment program reports short term and intermediate outcomes. |  |  |  |  |  |  |  |  |
| Evaluation results inform A-CRA treatment program planning and implementation. |  |  |  |  |  |  |  |  |
| A-CRA treatment program evaluation results are used to demonstrate successes to funders and other key stakeholders. |  |  |  |  |  |  |  |  |
| The A-CRA treatment program provides strong evidence to the public that the program works. |  |  |  |  |  |  |  |  |

1. For each statement in the next set of questions, select the number that best indicates the extent to which the A-CRA treatment program at your organization has or does the following things.

|  | 1 To little or no extent (1) | 2 (2) | 3 (3) | 4 (4) | 5 (5) | 6 (6) | 7 To a very great extent (7) | NA Not able to answer (8) |
| --- | --- | --- | --- | --- | --- | --- | --- | --- |
| The A-CRA treatment program periodically reviews the evidence base. |  |  |  |  |  |  |  |  |
| The A-CRA treatment program adapts strategies as needed. |  |  |  |  |  |  |  |  |
| The A-CRA treatment program adapts to new science. |  |  |  |  |  |  |  |  |
| The A-CRA treatment program proactively adapts to changes in the environment. |  |  |  |  |  |  |  |  |
| The A-CRA treatment program makes decisions about which components are ineffective and should not continue. |  |  |  |  |  |  |  |  |
| The A-CRA treatment program has communication strategies to secure and maintain public support. |  |  |  |  |  |  |  |  |
| A-CRA treatment program staff communicate the need for the program to the public. |  |  |  |  |  |  |  |  |
| The A-CRA treatment program is marketed in a way that generates interest. |  |  |  |  |  |  |  |  |
| The A-CRA treatment program increases community awareness of the issue. |  |  |  |  |  |  |  |  |
| The A-CRA treatment program demonstrates its value to the public. |  |  |  |  |  |  |  |  |

1. For each statement in the next set of questions, select the number that best indicates the extent to which the A-CRA treatment program at your organization has or does the following things.

|  | 1 To little or no extent (1) | 2 (2) | 3 (3) | 4 (4) | 5 (5) | 6 (6) | 7 To a very great extent (7) | NA Not able to answer (8) |
| --- | --- | --- | --- | --- | --- | --- | --- | --- |
| The A-CRA treatment program plans for future resource needs. |  |  |  |  |  |  |  |  |
| The A-CRA treatment program has a long-term financial plan. |  |  |  |  |  |  |  |  |
| The A-CRA treatment program has a sustainability plan. |  |  |  |  |  |  |  |  |
| The A-CRA treatment program's goals are understood by all stakeholders. |  |  |  |  |  |  |  |  |
| The A-CRA treatment program clearly outlines roles and responsibilities for all stakeholders. |  |  |  |  |  |  |  |  |
|  |  |  |  |  |  |  |  |  |

1. For each statement in the next set of questions, select the number that best indicates the extent to which the A-CRA treatment program at your organization has or does the following things.

|  | 1 To little or no extent (1) | 2 (2) | 3 (3) | 4 (4) | 5 (5) | 6 (6) | 7 To a very great extent (7) | NA Not able to answer (8) |
| --- | --- | --- | --- | --- | --- | --- | --- | --- |
| Diverse community organizations are invested in the success of the A-CRA treatment program. |  |  |  |  |  |  |  |  |
| The A-CRA treatment program communicates with community leaders. |  |  |  |  |  |  |  |  |
| Community leaders are involved with the A-CRA treatment program. |  |  |  |  |  |  |  |  |
| Community members are passionately committed to the A-CRA treatment program. |  |  |  |  |  |  |  |  |
| The community is engaged in the development of A-CRA treatment program goals. |  |  |  |  |  |  |  |  |

**The next set of questions are about staff at your agency.**

1. Have staff been assigned to implement A-CRA treatment services?
   1. No
   2. Yes
   3. Not sure/Not applicable

IF QUESTION 24 = YES, CONTINUE. OTHERWISE, SKIP TO QUESTION 26.

1. If yes, for how many years have staff been assigned to implement A-CRA treatment services?

Less than one year:

(IF CHECKED: For how many months have staff been assigned to implement A-CRA treatment services? ___________)

1. What is your best estimate of the number of staff who implement A-CRA treatment services?
   1. None
   2. Few
   3. Most
   4. All
2. Has an administrative-level individual within your organization been actively involved in advocating for A-CRA’s continuation?
   1. No
   2. Yes
   3. Not sure/Not applicable

IF QUESTION 27 = YES, CONTINUE. OTHERWISE, SKIP TO QUESTION 29.

1. For how many years have written goals and objectives related to A-CRA actually been followed?

___________

Less than one year:

(IF CHECKED: For how many months have written goals and objectives related to A-CRA actually been followed? ___________)

1. What is your best estimate of how active this administrative level individual has been advocating for A-CRA's continuation?
   1. Not at all
   2. Minimally
   3. Moderately
   4. Very
2. Do staff in your organization, other than those actually implementing A-CRA, actively contribute to A-CRA treatment services?
   1. No
   2. Yes
   3. Not sure/Not applicable

IF QUESTION 30 = YES, CONTINUE. OTHERWISE, SKIP TO QUESTION 32.

1. For how many years have such staff in your organization actively contributed to A-CRA treatment services?

Less than one year:

(IF CHECKED: For how many months have such staff in your organization actively contributed to A-CRA treatment services? ___________)

1. Of all the staff in your organization who could contribute to the operation of A-CRA treatment services, what is your best estimate of the proportion that actually contribute to it?
   1. None
   2. Few
   3. Most
   4. All
2. Please rate your level of agreement with the following statements, ranging from strongly disagree to strongly agree:

|  | Strongly Disagree (1) | Somewhat Disagree (2) | Neither Agree nor Disagree (3) | Somewhat Agree (4) | Strongly Agree (5) |
| --- | --- | --- | --- | --- | --- |
| My supervisor has established clear standards for the implementation of evidence-based practice |  |  |  |  |  |
| My supervisor has developed a plan to facilitate implementation of evidence-based practice |  |  |  |  |  |
| My supervisor has removed obstacles to the implementation of evidence-based practice |  |  |  |  |  |
| My supervisor knows what they are talking about when it comes to evidence-based practice |  |  |  |  |  |
| My supervisor is knowledgeable about evidence-based practice |  |  |  |  |  |
| My supervisor is able to answer staff’s questions about evidence-based practice |  |  |  |  |  |
| My supervisor supports employee efforts to use evidence-based practice |  |  |  |  |  |
| My supervisor supports employee efforts to learn more about evidence-based practice |  |  |  |  |  |
| My supervisor recognizes and appreciates employee efforts toward successful implementation of evidence-based practice |  |  |  |  |  |
| My supervisor perseveres through the ups and downs of implementing evidence-based practice |  |  |  |  |  |
| My supervisor carries on through the challenges of implementing evidence-based practice |  |  |  |  |  |
| My supervisor reacts to critical issues regarding the implementation of evidence-based practice by openly and effectively addressing the problem(s) |  |  |  |  |  |

1. Please rate your level of agreement with the following statements regarding A-CRA specifically, ranging from strongly disagree to strongly agree:

|  | Strongly Disagree (1) | Somewhat Disagree (2) | Neither Agree nor Disagree (3) | Somewhat Agree (4) | Strongly Agree (5) |
| --- | --- | --- | --- | --- | --- |
| My supervisor has established clear standards for the implementation of A-CRA |  |  |  |  |  |
| My supervisor has developed a plan to facilitate implementation of A-CRA |  |  |  |  |  |
| My supervisor has removed obstacles to the implementation of A-CRA |  |  |  |  |  |
| My supervisor knows what they are talking about when it comes to A-CRA |  |  |  |  |  |
| My supervisor is knowledgeable about A-CRA |  |  |  |  |  |
| My supervisor is able to answer staff’s questions about A-CRA |  |  |  |  |  |
| My supervisor supports employee efforts to use A-CRA |  |  |  |  |  |
| My supervisor supports employee efforts to learn more about A-CRA |  |  |  |  |  |
| My supervisor recognizes and appreciates employee efforts toward successful implementation of A-CRA |  |  |  |  |  |
| My supervisor perseveres through the ups and downs of implementing A-CRA |  |  |  |  |  |
| My supervisor carries on through the challenges of implementing A-CRA |  |  |  |  |  |
| My supervisor reacts to critical issues regarding the implementation of A-CRA by openly and effectively addressing the problem(s) |  |  |  |  |  |

1. Which of the following services are provided by your agency? (MARK ALL THAT APPLY**)**
   1. Screening for substance use
   2. Screening for mental health
   3. Comprehensive substance use assessment or diagnosis
   4. Comprehensive mental health assessment or diagnosis (for example psychological or psychiatric evaluation and testing)
   5. Outreach to persons in the community who might need treatment
   6. Interim services for clients when admission is not possible
   7. Discharge planning
   8. Aftercare/continuing care
   9. Case management
   10. Social skills development
   11. Mentoring/peer support
   12. Substance use education
   13. Mental health education
   14. Self-help groups
   15. Medication Assisted Therapy (e.g., Antabuse, Naltrexone, Campral, Methadone, Buprenorphine)
   16. Smoking cessation medications (Nicotine replacement or non-nicotine)
   17. Medications for psychiatric disorders
2. What is the primary focus of this facility at this location?
   1. Substance use treatment services
   2. Mental health services
   3. Mix of mental health and substance use treatment services (neither is primary)
   4. General health care
   5. Other (Specify:) _______________
3. Is this facility operated by:
   1. A private for-profit organization
   2. A private non-profit organization
   3. State government
   4. Local, county, or community government
   5. Tribal government
   6. Federal government
4. What is the average length of stay in the treatment program? (Note: This is how long clients actually stay in the program.)

_________ (length in days)

_________ (length in weeks)

1. In total, how many youths can be in the treatment program at one time? _________
2. What percent of the youths in the substance use treatment program have at least one other mental health diagnosis, besides substance use? ____________
3. Thinking now about the design of this treatment program, how many of the following types of sessions does the average youth receive per week? Your best guess is fine.
   1. Individual therapy sessions? ____________
   2. Psychoeducational sessions? ____________
   3. Group therapy sessions? _____________
4. What is the minimum age for admission? __________
5. What is the maximum age for admission? __________
6. How many **youth treatment supervisors** *left* your program/agency in the past 6 months (e.g., quit, fired, retired)? Your best guess is fine.

IF ANSWER TO QUESTION 44>0, CONTINUE. OTHERWISE, SKIP TO QUESTION 46.

1. How many of the youth treatment supervisors who left your agency in the past 6 months had been certified to deliver A-CRA? (By certified, we mean they completed all training requirements – such as coaching and submission of recorded sessions – and received written documentation of certification. This includes first-level, full, transitional age youth (TAY), and supervisor certifications.) _____________
2. How many **youth treatment supervisors** have been *hired* by your agency in the past 6 months? Your best guess is fine. _____________

IF ANSWER TO QUESTION 46>0, CONTINUE. OTHERWISE, SKIP TO QUESTION 49.

1. How many of the youth treatment supervisors hired in the past 6 months are currently certified in A-CRA? (By certified, we mean they completed all training requirements – such as coaching and submission of recorded sessions – and received written documentation of certification. This includes first-level, full, and supervisor certifications.) ___________
2. How many of the supervisors hired in the past 6 month**s** are currently working toward A-CRA certification? ___________
3. How many clinicians *left* your program/agency in the past 6 months (e.g., quit, fired, retired)? Your best guess is fine. _____________

IF ANSWER TO QUESTION 49>0, CONTINUE. OTHERWISE, SKIP TO QUESTION 51.

1. How many of the clinicians who left your agency in the past 6 months had been certified to deliver A-CRA? (By certified, we mean they completed all training requirements – such as coaching and submission of recorded sessions – and received written documentation of certification. This includes first-level, full, and supervisor certifications.) ___________
2. How many **clinicians** have been *hired* by your agency in the past 6 months? Your best guess is fine. ___

IF ANSWER TO QUESTION 51>0, CONTINUE. OTHERWISE, SKIP TO QUESTION 54.

1. How many of these clinicians hired in the past 6 months are currently certified in A-CRA? (By certified, we mean they completed all training requirements – such as coaching and submission of recorded sessions – and received written documentation of certification. This includes first-level, full, and supervisor certifications.) __________
2. How many of the clinicians hired in the past 6 months are currently working toward A-CRA certification?

**__________**

1. Thinking about sources of reimbursement, what percentage of treatment for youth clients is paid via each of the following? Please enter 0 for any items that do not apply. Your best guess is fine.
   1. Out-of-pocket/sliding scale fees _____%
   2. Private indemnity insurance _____%
   3. HMOs/PPOs _____%
   4. Medicaid _____%
   5. Juvenile justice contracts _____%
   6. Public assistance _____%
   7. Federal grant _____%
   8. State grant _____%
   9. Other (describe): ________________
2. Now, thinking about sources of referrals, what percentages of youth clients are referred to your center by the following? Please enter 0 for any items that do not apply. Your best guess is fine.
   1. Family _____%
   2. Schools _____%
   3. Juvenile justice system _____%
   4. The state’s child welfare system _____%
   5. Other treatment & healthcare providers _____%
   6. Self-referrals _____%
   7. Other (please specify): _____________

**We know that substance use treatment programs have been affected by the COVID-19 crisis, and we are interested in hearing how you and your A-CRA program changed your processes in light of these challenges.**

1. Did your A-CRA program experience any changes in practices due to COVID-19? Changes might include limiting the services to emergency-only cases, providing virtual/telehealth options, or other changes?
   1. No
   2. Yes

IF QUESTION 56 = YES, CONTINUE. OTHERWISE, SKIP TO QUESTION 60

1. Please rate how the following areas of practice at the A-CRA program were affected, ranging from no effect to completely different:

|  | No effect (0) | Small changes (1) | Moderate changes (2) | Large changes (3) | Completely different (4) |
| --- | --- | --- | --- | --- | --- |
| Referrals to the A-CRA program |  |  |  |  |  |
| A-CRA assessment services |  |  |  |  |  |
| A-CRA treatment services |  |  |  |  |  |
| Case management for A-CRA clients |  |  |  |  |  |
| Staffing, facilities, and other operations for the A-CRA program |  |  |  |  |  |

1. Has your A-CRA program re-opened for normal operations? We understand some aspects of your practice may remain changed for many reasons. If your A-CRA program has mostly resumed pre-crisis operations, select yes. If your A-CRA program is still operating under significantly limited or altered capacity, select no.
   1. No
   2. Yes
2. How long did it take for your A-CRA program to re-open for normal operations? ___________
3. Did you work remotely due to the COVID-19 crisis?
   1. No
   2. Yes, partly remote and partly in-person
   3. Yes, fully remote
4. Given the financial impact of COVID-19, did the organization where you work have to reduce staff hours, furlough employees, or reduce salary of employees?
   1. No
   2. Yes, but this did not directly affect A-CRA providers
   3. Yes, and this directly affected A-CRA providers
5. Did your organization start providing assessment, treatment, or case management services via any telehealth method (video, telephone, etc.) due to the COVID-19 crisis?
   1. No
   2. Yes, but this did not include A-CRA services
   3. Yes, and this included A-CRA services via telehealth
6. Please rate how each of the following COVID-related changes affected your organization’s ability to sustain A-CRA services, as compared to how things were before COVID:

|  | Much harder to sustain (1) | Somewhat harder (2) | A little harder (3) | About the same (4) | A little easier (5) | Somewhat easier (6) | Much easier to sustain (7) | Not applicable (0) |
| --- | --- | --- | --- | --- | --- | --- | --- | --- |
| Changes in referrals to the A-CRA program |  |  |  |  |  |  |  |  |
| Changes in A-CRA assessment services |  |  |  |  |  |  |  |  |
| Changes in A-CRA treatment services |  |  |  |  |  |  |  |  |
| Changes in case management for A-CRA clients |  |  |  |  |  |  |  |  |
| Changes in staffing for the A-CRA program |  |  |  |  |  |  |  |  |
| Changes in remote work |  |  |  |  |  |  |  |  |
| Changes in use of telehealth or virtual delivery of services |  |  |  |  |  |  |  |  |

1. Taken together, how much did COVID-related changes affect your organization’s ability to sustain A-CRA services, as compared to how things were before March 2020:
   1. Much harder to sustain
   2. Somewhat harder
   3. A little harder
   4. About the same
   5. A little easier
   6. Somewhat easier
   7. Much easier to sustain

**We are close to the end of the survey and just have a few more background questions about you.**

1. What is your gender?
   1. Male
   2. Female
   3. Non-binary/genderqueer
   4. Prefer to self-describe: ____________
2. How old are you? __________
3. Are you of Hispanic or Latino origin or descent?
   1. No
   2. Yes
4. What is your race? Please select one or more.
   1. White
   2. Black or African American
   3. Asian
   4. Native Hawaiian or Other Pacific Islander
   5. American Indian or Alaskan Native
   6. Prefer to self-describe: ____________
5. What is your highest level of education?
   1. No high school diploma or equivalent
   2. High school diploma or equivalent (GED)
   3. Some college, but no degree
   4. Associate’s degree
   5. Bachelor's degree
   6. Master's degree
   7. Doctoral degree or equivalent
   8. Prefer to self-describe: ____________
6. How many years of experience do you have in substance use counseling as a clinician? ________
7. How many years of experience do you have as a clinical supervisor for substance use counselors/clinicians?

__________

1. What type of provider are you?
   1. Certified Drug Abuse Counselor (CADAC)
   2. Clinical Psychologist
   3. Clinical Social Worker
   4. Licensed Chemical Dependency Counselor (LCDC)
   5. Psychiatric Nurse
   6. Psychiatrist
   7. Prefer to self-describe: ____________

**CONGRATULATIONS, YOU HAVE COMPLETED THE SURVEY!**

Please indicate the e-mail address where you would like your $50 Amazon e-gift card. Your e-mail will not be linked to your survey responses.

Email: ___________________________________

Confirm email: ______________________________

[OPTION TO SELECT] Do not send me the $50 e-gift card.

**Please hit SUBMIT to send us your answers!**

**Non-Sustainer Survey**

1. We understand from our initial call that your agency stopped delivering A-CRA. When did you/your agency stop delivering A-CRA? If you do not know the exact date, please give your best estimate.

Month:

- 1. 01
  2. 02
  3. 03
  4. 04
  5. 05
  6. 06
  7. 07
  8. 08
  9. 09
  10. 10
  11. 11
  12. 12

Year:

1. 2000
2. 2001
3. 2002
4. 2003
5. 2004
6. 2005
7. 2006
8. 2007
9. 2008
10. 2009
11. 2010
12. 2011
13. 2012
14. 2013
15. 2014
16. 2015
17. 2016
18. 2017
19. 2018
20. 2019
21. 2020
22. 2021
23. 2022
24. DON'T KNOW
25. I WAS NOT EMPLOYED AT THIS AGENCY WHEN A-CRA WAS IMPLEMENTED
26. Are you familiar with Adolescent Community Reinforcement Approach (A-CRA)?
    1. No
    2. Yes

**The first set of questions are about A-CRA. Please select the correct answer for each of the following:**

1. The basic premise of CRA is that:
   1. with adequate skills training an individual can really combat any substance use problem.
   2. although will-power is ultimately still responsible for the majority of behavior change, at least a commitment to sobriety is also required.
   3. one must make a non-drinking/using lifestyle as rewarding as a drinking/using lifestyle.
   4. once an individual has a good job and the family is supportive again, the other pieces naturally fall into place.
   5. Don't know
2. A simple and easy way to monitor a youth's own report of progress in a number of areas is through:
   1. a behavioral contract.
   2. the CRA Happiness Scale.
   3. the CRA Functional Analysis for Non-Drinking Behaviors.
   4. the Self-Reminder To Be Nice form.
   5. Don't know
3. Why is it so important for a CRA therapist to always be looking for a youth's reinforcers?
   1. so that the therapist can be aware of the size of the youth's social support system at all times.
   2. because these may need to be resorted to and incorporated into the treatment plan if mild punishment does not appear to be working.
   3. because youths will be more likely to change their behavior if they feel they are being rewarded in doing so.
   4. because Community Reinforcement Approach implies that reinforcers can usually only be identified with the assistance of the youth's community.
   5. Don't know
4. Most behavioral and cognitive-behavioral treatments rely heavily on role-plays, despite the fact that it is often uncomfortable for clients (and therapists!) to do them. Role-plays are considered important because:
   1. they provide the therapist with valuable information about a youth's level of resistance to treatment.
   2. they are good for practice, because it is actually more difficult for a youth to do a role-play during a session than it is for them to try out the interaction in the natural environment.
   3. they offer valuable information about the quality of the therapeutic relationship and are early indicators of problematic transference.
   4. they give youths the opportunity to practice their new skills in a supportive environment with a person who can provide feedback.
   5. Don't know
5. The A-CRA Functional Analysis for Substance Use chart looks at the youth's short-term positive consequences for substance use. The reason for looking at these is:
   1. to be sure that the youth is fully aware from the start of what they are giving up by becoming substance free, and to be agreeable to it.
   2. to motivate the youth by reminding them of all the hardships endured (by the youth and their family) as a result of their use.
   3. to see what role substance use is serving, so that the individual can later be taught healthier ways to obtain these things.
   4. to recognize the types of obstacles that have successfully interfered with substance use in the past.
   5. Don't know
6. Why is it important to have both the youth and caregiver complete the Relationship Happiness Scale form during the Caregiver III and IV sessions?
   1. to enable the therapist to solve the families’ problems in one session.
   2. to get them to identify their weaknesses.
   3. to expose the source of unhappiness within the relationship.
   4. to have them practice communication and problem-solving skills through role-plays.
   5. Don't know
7. One CRA procedure entails: getting a client to role-play a phone call in session to an organization of interest (e.g. N.A.), and then having them actually place the call during the session, locating a contact person in the community for N.A., and calling them, and reviewing in the next session the experience of attending an N.A. meeting. The name of this procedure is:
   1. systematic encouragement
   2. reinforcer sampling
   3. cognitive restructuring
   4. communication skills training
   5. Don't know
8. Which of the following is not one of the common mistakes made by therapists who are implementing CRA (each of the others is a common mistake):
   1. losing sight of the youth's reinforcers.
   2. not emphasizing the importance of having a satisfying job.
   3. neglecting to monitor the youth's drinking and drug use.
   4. not checking for generalizability of skills.
   5. Don't know
9. In first selecting a category from the Happiness Scale to work on with a youth, it is advisable to begin with:
   1. an area that the youth express extreme unhappiness with, thereby demonstrating that you do not intend to shy away from the really difficult problems.
   2. an area that the youth states is really the main reason for seeking treatment in the first place, and consequently is the area most important to them.
   3. an area that is broad and really includes most of the youth's drug and non-drug problems, so that many different important issues get addressed right from the start.
   4. an area that the youth has reported mild-moderate (not extreme) unhappiness in, since it will probably provide an opportunity for the youth to experience some early success in therapy.
   5. Don't know
10. Therapists often spend very little time discussing a youth's social/recreational activities. However, it is important to do so because:
    1. many youths' social lives are built around alcohol/drugs, so they'll need help developing new drug-free pleasurable activities that compete with substance use.
    2. youth will assume that if you do not discuss this part of their lives, then they can probably get away with having an occasional beer during their recreational activities.
    3. even though a youth's social life is not particularly important to discuss from a therapeutic sense, it gives out the valuable message that you care enough about the youth to do it anyway.
    4. youths' behavior during their free time is really the best indicator we have of their personal values, and consequently it is often a predictor of whether they will remain committed to changing their using behavior.
    5. Don't know

**The following questions ask about A-CRA in your organization. Please indicate the extent to which you agree with the following items:**

1. Staff use A-CRA as much as possible when appropriate
   1. Not at all
   2. To a slight extent
   3. To a moderate extent
   4. To a great extent
   5. To a very great extent
2. Staff continue to use A-CRA throughout changing circumstances
   1. Not at all
   2. To a slight extent
   3. To a moderate extent
   4. To a great extent
   5. To a very great extent
3. A-CRA is a routine part of our practice
   1. Not at all
   2. To a slight extent
   3. To a moderate extent
   4. To a great extent
   5. To a very great extent
4. Please think about the six-month period, right before A-CRA treatment delivery ended. Please rate your level of agreement with the following statements, ranging from strongly disagree to strongly agree.

|  | Strongly Disagree (1) | Somewhat Disagree (2) | Neither Agree nor Disagree (3) | Somewhat Agree (4) | Strongly Agree (5) |
| --- | --- | --- | --- | --- | --- |
| In general, A-CRA was more effective in creating attitudes that discourage substance use by clients than other treatment practices. |  |  |  |  |  |
| A-CRA was more effective in reducing substance use by clients than our current treatment practices. |  |  |  |  |  |
| A-CRA improved the overall quality of substance use treatment in this agency. |  |  |  |  |  |
| A-CRA was better than our previous treatment practices for substance use. |  |  |  |  |  |
| A-CRA was difficult to teach. |  |  |  |  |  |
| A-CRA was hard for therapists to understand. |  |  |  |  |  |
| A-CRA required complex therapeutic strategies. |  |  |  |  |  |

1. Again, please think about the six-month period right before A-CRA treatment delivery ended. Please rate your level of agreement with the following statements, ranging from strongly disagree to strongly agree.

|  | Strongly Disagree (1) | Somewhat Disagree (2) | Neither Agree nor Disagree (3) | Somewhat Agree (4) | Strongly Agree (5) |
| --- | --- | --- | --- | --- | --- |
| There was a definite need for A-CRA among my agency's clientele |  |  |  |  |  |
| There was a high level of interest for A-CRA among my agency's clientele |  |  |  |  |  |
| A-CRA was timely |  |  |  |  |  |
| A-CRA helped build partnerships |  |  |  |  |  |
| A-CRA had an impact on participants |  |  |  |  |  |

1. Again, please think about the six-month period right before A-CRA treatment delivery ended. Please rate your level of agreement with the following statements, ranging from strongly disagree to strongly agree.

|  | Strongly Disagree (1) | Somewhat Disagree (2) | Neither Agree nor Disagree (3) | Somewhat Agree (4) | Strongly Agree (5) |
| --- | --- | --- | --- | --- | --- |
| Financing A-CRA was difficult |  |  |  |  |  |
| Recruiting participants for A-CRA was difficult |  |  |  |  |  |
| Recruiting staff to work on A-CRA was difficult |  |  |  |  |  |
| Finding time to prepare for A-CRA was difficult |  |  |  |  |  |
| Staff working on A-CRA had to learn new skills to deliver it |  |  |  |  |  |

Do you agree or disagree:

1. Management wanted to continue A-CRA
   1. Strongly Disagree
   2. Somewhat Disagree
   3. Neither Agree nor Disagree
   4. Somewhat Agree
   5. Strongly Agree
2. Again, please think about the six-month period right before A-CRA treatment delivery ended. The following items ask about financial status of your service agency. Please indicate the extent to which you agree with each statement.

|  | Not at all (1) | Slight extent (2) | Moderate extent (3) | Great extent (4) | Very great extent (5) |
| --- | --- | --- | --- | --- | --- |
| Overall, this agency was in a state of financial distress. |  |  |  |  |  |
| The financial status of this agency had improved in recent years. |  |  |  |  |  |
| The ongoing financial viability of this agency was a major concern. |  |  |  |  |  |
| This agency was in a strong financial position. |  |  |  |  |  |
| One of this agency’s main goals was to manage its finances effectively. |  |  |  |  |  |
| People in this agency thought that the organization’s financial health was important. |  |  |  |  |  |
| Being financially viable was a top priority in this agency. |  |  |  |  |  |
| Evidence-based practices were expensive. |  |  |  |  |  |
| Evidence-based practices offered many financial advantages to this agency. |  |  |  |  |  |
| Our agency had done a good job of finding adequate funding for evidence-based practices. |  |  |  |  |  |
| This agency would not have implemented evidence-based practices if they were not externally funded. |  |  |  |  |  |

1. Again, please think about the six-month period right before A-CRA treatment delivery ended. For each statement in the next set of questions, select the number that best indicates the extent to which the A-CRA treatment program at your organization had or did the following things during that time.

|  | 1 To little or no extent (1) | 2 (2) | 3 (3) | 4 (4) | 5 (5) | 6 (6) | 7 To a very great extent (7) | NA Not able to answer (8) |
| --- | --- | --- | --- | --- | --- | --- | --- | --- |
| Program champions advocated for the A-CRA treatment program. |  |  |  |  |  |  |  |  |
| The A-CRA treatment program had strong champions with the ability to garner resources. |  |  |  |  |  |  |  |  |
| The A-CRA treatment program had political support within the larger organization. |  |  |  |  |  |  |  |  |
| The A-CRA treatment program had political support from outside of the organization. |  |  |  |  |  |  |  |  |
| The A-CRA treatment program had strong advocacy support. |  |  |  |  |  |  |  |  |
| The A-CRA treatment program existed in a supportive state economic climate. |  |  |  |  |  |  |  |  |
| The A-CRA treatment program implemented policies to help ensure sustained funding |  |  |  |  |  |  |  |  |
| The A-CRA treatment program was funded through a variety of sources. |  |  |  |  |  |  |  |  |
| The A-CRA treatment program had a combination of stable and flexible funding. |  |  |  |  |  |  |  |  |
| The A-CRA treatment program had sustained funding. |  |  |  |  |  |  |  |  |

1. Again, please think about the six-month period right before A-CRA treatment delivery ended. For each statement in the next set of questions, select the number that best indicates the extent to which the A-CRA treatment program at your organization had or did the following things during that time.

|  | 1 To little or no extent (1) | 2 (2) | 3 (3) | 4 (4) | 5 (5) | 6 (6) | 7 To a very great extent (7) | NA Not able to answer (8) |
| --- | --- | --- | --- | --- | --- | --- | --- | --- |
| The A-CRA treatment program was well integrated into the operations of the organization. |  |  |  |  |  |  |  |  |
| Organizational systems were in place to support the various A-CRA treatment program needs. |  |  |  |  |  |  |  |  |
| Leadership effectively articulated the vision of the A-CRA treatment program to external partners. |  |  |  |  |  |  |  |  |
| Leadership efficiently managed staff and other resources. |  |  |  |  |  |  |  |  |
| The A-CRA treatment program had adequate staff to complete the program’s goals. |  |  |  |  |  |  |  |  |
| The A-CRA treatment program had the capacity for quality program evaluation. |  |  |  |  |  |  |  |  |
| The A-CRA treatment program reported short term and intermediate outcomes. |  |  |  |  |  |  |  |  |
| Evaluation results informed A-CRA treatment program planning and implementation. |  |  |  |  |  |  |  |  |
| A-CRA treatment program evaluation results were used to demonstrate successes to funders and other key stakeholders. |  |  |  |  |  |  |  |  |
| The A-CRA treatment program provided strong evidence to the public that the program works. |  |  |  |  |  |  |  |  |

1. Again, please think about the six-month period right before A-CRA treatment delivery ended. For each statement in the next set of questions, select the number that best indicates the extent to which the A-CRA treatment program at your organization had or did the following things during that time.

|  | 1 To little or no extent (1) | 2 (2) | 3 (3) | 4 (4) | 5 (5) | 6 (6) | 7 To a very great extent (7) | NA Not able to answer (8) |
| --- | --- | --- | --- | --- | --- | --- | --- | --- |
| The A-CRA treatment program periodically reviewed the evidence base. |  |  |  |  |  |  |  |  |
| The A-CRA treatment program adapted strategies as needed. |  |  |  |  |  |  |  |  |
| The A-CRA treatment program adapted to new science. |  |  |  |  |  |  |  |  |
| The A-CRA treatment program proactively adapted to changes in the environment. |  |  |  |  |  |  |  |  |
| The A-CRA treatment program made decisions about which components were ineffective and should not continue. |  |  |  |  |  |  |  |  |
| The A-CRA treatment program had communication strategies to secure and maintain public support. |  |  |  |  |  |  |  |  |
| A-CRA treatment program staff communicated the need for the program to the public. |  |  |  |  |  |  |  |  |
| The A-CRA treatment program was marketed in a way that generated interest. |  |  |  |  |  |  |  |  |
| The A-CRA treatment program increased community awareness of the issue. |  |  |  |  |  |  |  |  |
| The A-CRA treatment program demonstrated its value to the public. |  |  |  |  |  |  |  |  |

1. Again, please think about the six-month period right before A-CRA treatment delivery ended. For each statement in the next set of questions, select the number that best indicates the extent to which the A-CRA treatment program at your organization had or did the following things during that time.

|  | 1 To little or no extent (1) | 2 (2) | 3 (3) | 4 (4) | 5 (5) | 6 (6) | 7 To a very great extent (7) | NA Not able to answer (8) |
| --- | --- | --- | --- | --- | --- | --- | --- | --- |
| The A-CRA treatment program planned for future resource needs. |  |  |  |  |  |  |  |  |
| The A-CRA treatment program had a long-term financial plan. |  |  |  |  |  |  |  |  |
| The A-CRA treatment program had a sustainability plan. |  |  |  |  |  |  |  |  |
| The A-CRA treatment program's goals were understood by all stakeholders. |  |  |  |  |  |  |  |  |
| The A-CRA treatment program clearly outlined roles and responsibilities for all stakeholders. |  |  |  |  |  |  |  |  |

1. Again, please think about the six-month period right before A-CRA treatment delivery ended. For each statement in the next set of questions, select the number that best indicates the extent to which the A-CRA treatment program at your organization had or did the following things during that time.

|  | 1 To little or no extent (1) | 2 (2) | 3 (3) | 4 (4) | 5 (5) | 6 (6) | 7 To a very great extent (7) | NA Not able to answer (8) |
| --- | --- | --- | --- | --- | --- | --- | --- | --- |
| Diverse community organizations were invested in the success of the A-CRA treatment program. |  |  |  |  |  |  |  |  |
| The A-CRA treatment program communicated with community leaders. |  |  |  |  |  |  |  |  |
| Community leaders were involved with the A-CRA treatment program. |  |  |  |  |  |  |  |  |
| Community members were passionately committed to the A-CRA treatment program. |  |  |  |  |  |  |  |  |
| The community was engaged in the development of A-CRA treatment program goals. |  |  |  |  |  |  |  |  |

**The next set of questions are about staff at your agency.**

1. During the six months right before A-CRA treatment delivery ended, were staff assigned to implement A-CRA treatment services?
   1. No
   2. Yes
   3. Not sure/Not applicable

IF QUESTION 26 = YES, CONTINUE. OTHERWISE, SKIP TO QUESTION 28.

1. If yes, for how many years were staff assigned to implement A-CRA treatment services? ___________

Less than one year:

(IF CHECKED: For how many months were staff assigned to implement A-CRA treatment services? ___________)

1. During the six months right before A-CRA treatment delivery ended, what is your best estimate of the number of staff who implemented A-CRA treatment services?
   1. None
   2. Few
   3. Most
   4. All
2. During the six months right before A-CRA treatment delivery ended, was an administrative-level individual within your organization actively involved in advocating for A-CRA’s continuation?
   1. No
   2. Yes
   3. Not sure/Not applicable

IF QUESTION 29 = YES, CONTINUE. OTHERWISE, SKIP TO QUESTION 31.

1. For how many years were written goals and objectives related to A-CRA actually followed? ___________

Less than one year:

(IF CHECKED: For how many months were written goals and objectives related to A-CRA actually followed? ___________)

1. During the six months right before A-CRA treatment delivery ended, what is your best estimate of how active this administrative level individual was in advocating for A-CRA's continuation?
   1. Not at all
   2. Minimally
   3. Moderately
   4. Very
2. During the six months right before A-CRA treatment delivery ended, did staff in your organization, other than those who actually implemented A-CRA, actively contribute to A-CRA treatment services?
   1. No
   2. Yes
   3. Not sure/Not applicable

IF QUESTION 32 = YES, CONTINUE. OTHERWISE, SKIP TO QUESTION 34.

1. For how many years did such staff in your organization actively contribute to A-CRA treatment services?

___________

Less than one year:

(IF CHECKED: For how many months did such staff in your organization actively contribute to A-CRA treatment services? ___________)

1. During the six months right before A-CRA treatment delivery ended, of all the staff in your organization who could have contributed to the operation of A-CRA treatment services, what is your best estimate of the proportion that actually contributed to it?
   1. None
   2. Few
   3. Most
   4. All

**The following questions relate to your current supervisor.**

1. Please rate your level of agreement with the following statements, ranging from strongly disagree to strongly agree.

|  | Strongly Disagree (1) | Somewhat Disagree (2) | Neither Agree nor Disagree (3) | Somewhat Agree (4) | Strongly Agree (5) |
| --- | --- | --- | --- | --- | --- |
| My supervisor has established clear standards for the implementation of evidence-based practice |  |  |  |  |  |
| My supervisor has developed a plan to facilitate implementation of evidence-based practice |  |  |  |  |  |
| My supervisor has removed obstacles to the implementation of evidence-based practice |  |  |  |  |  |
| My supervisor knows what they are talking about when it comes to evidence-based practice |  |  |  |  |  |
| My supervisor is knowledgeable about evidence-based practice |  |  |  |  |  |
| My supervisor is able to answer staff’s questions about evidence-based practice |  |  |  |  |  |
| My supervisor supports employee efforts to use evidence-based practice |  |  |  |  |  |
| My supervisor supports employee efforts to learn more about evidence-based practice |  |  |  |  |  |
| My supervisor recognizes and appreciates employee efforts toward successful implementation of evidence-based practice |  |  |  |  |  |
| My supervisor perseveres through the ups and downs of implementing evidence-based practice |  |  |  |  |  |
| My supervisor carries on through the challenges of implementing evidence-based practice |  |  |  |  |  |
| My supervisor reacts to critical issues regarding the implementation of evidence-based practice by openly and effectively addressing the problem(s) |  |  |  |  |  |

1. Now, please think again about the six-month period right before A-CRA treatment delivery ended. Please rate your level of agreement with the following statements related to A-CRA regarding A-CRA your supervisor at that time, ranging from strongly disagree to strongly agree:

|  | Strongly Disagree (1) | Somewhat Disagree (2) | Neither Agree nor Disagree (3) | Somewhat Agree (4) | Strongly Agree (5) |
| --- | --- | --- | --- | --- | --- |
| My supervisor had established clear standards for the implementation of A-CRA |  |  |  |  |  |
| My supervisor had developed a plan to facilitate implementation of A-CRA |  |  |  |  |  |
| My supervisor had removed obstacles to the implementation of A-CRA |  |  |  |  |  |
| My supervisor knew what they were talking about when it came to A-CRA |  |  |  |  |  |
| My supervisor was knowledgeable about A-CRA |  |  |  |  |  |
| My supervisor was able to answer staff’s questions about A-CRA |  |  |  |  |  |
| My supervisor supported employee efforts to use A-CRA |  |  |  |  |  |
| My supervisor supported employee efforts to learn more about A-CRA |  |  |  |  |  |
| My supervisor recognized and appreciated employee efforts toward successful implementation of A-CRA |  |  |  |  |  |
| My supervisor persevered through the ups and downs of implementing A-CRA |  |  |  |  |  |
| My supervisor carried on through the challenges of implementing A-CRA |  |  |  |  |  |
| My supervisor reacted to critical issues regarding the implementation of A-CRA by openly and effectively addressing the problem(s). |  |  |  |  |  |

1. Which of the following services are provided by your agency? (MARK ALL THAT APPLY**)**
   1. Screening for substance use
   2. Screening for mental health
   3. Comprehensive substance use assessment or diagnosis
   4. Comprehensive mental health assessment or diagnosis (for example psychological or psychiatric evaluation and testing)
   5. Outreach to persons in the community who might need treatment
   6. Interim services for clients when admission is not possible
   7. Discharge planning
   8. Aftercare/continuing care
   9. Case management
   10. Social skills development
   11. Mentoring/peer support
   12. Substance use education
   13. Mental health education
   14. Self-help groups
   15. Medication Assisted Therapy (e.g., Antabuse, Naltrexone, Campral, Methadone, Buprenorphine)
   16. Smoking cessation medications (Nicotine replacement or non-nicotine)
   17. Medications for psychiatric disorders
2. What is the primary focus of this facility at this location?
   1. Substance use treatment services
   2. Mental health services
   3. Mix of mental health and substance use treatment services (neither is primary)
   4. General health care
   5. Other (Specify:) _______________
3. Is this facility operated by:
   1. A private for-profit organization
   2. A private non-profit organization
   3. State government
   4. Local, county, or community government
   5. Tribal government
   6. Federal government
4. What is the average length of stay in the treatment program? (Note: This is how long clients actually stay in the program.)

_________ (length in days)

_________ (length in weeks)

1. In total, how many youths can be in the treatment program at one time? _________
2. What percent of the youths in the substance use treatment program have at least one other mental health diagnosis, besides substance use? ____________
3. Thinking now about the design of this treatment program, how many of the following types of sessions does the average youth receive per week? Your best guess is fine.
   1. Individual therapy sessions? ___________
   2. Psychoeducational sessions? ____________
   3. Group therapy sessions? _____________
4. What is the minimum age for admission? __________
5. What is the maximum age for admission? __________
6. How many **youth treatment supervisors** *left* your program/agency in the past 6 months (e.g., quit, fired, retired)? Your best guess is fine. _________

IF ANSWER TO QUESTION 46>0, CONTINUE. OTHERWISE, SKIP TO QUESTION 48.

1. How many of the youth treatment supervisors who left your agency in the past 6 months had been certified to deliver A-CRA? (By certified, we mean they completed all training requirements – such as coaching and submission of recorded sessions – and received written documentation of certification. This includes first-level, full, transitional age youth (TAY), and supervisor certifications.) __________
2. How many **youth treatment supervisors** have been *hired* by your agency in the past 6 months? Your best guess is fine. _____________

IF ANSWER TO QUESTION 48>0, CONTINUE. OTHERWISE, SKIP TO QUESTION 51.

1. How many of the youth treatment supervisors hired in the past 6 months are currently certified in A-CRA? (By certified, we mean they completed all training requirements – such as coaching and submission of recorded sessions – and received written documentation of certification. This includes first-level, full, and supervisor certifications.)  **___________**
2. How many of the supervisors hired in the past 6 months are currently working toward A-CRA certification? ___________
3. How many **clinicians** *left* your program/agency in the past 6 months (e.g., quit, fired, retired)? Your best guess is fine. _____________

IF ANSWER TO QUESTION 51>0, CONTINUE. OTHERWISE, SKIP TO QUESTION 53.

1. How many of the clinicians who left your agency in the past 6 months had been certified to deliver A-CRA? (By certified, we mean they completed all training requirements – such as coaching and submission of recorded sessions – and received written documentation of certification. This includes first-level, full, and supervisor certifications.) **___________**
2. How many **clinicians** have been *hired* by your agency in the past 6 months? Your best guess is fine. __________

IF ANSWER TO QUESTION 53>0, CONTINUE. OTHERWISE, SKIP TO QUESTION 56.

1. How many of these clinicians hired in the past 6 months are currently certified in A-CRA? (By certified, we mean they completed all training requirements – such as coaching and submission of recorded sessions – and received written documentation of certification. This includes first-level, full, and supervisor certifications.) __________
2. How many of the clinicians hired in the past 6 months are currently working toward A-CRA certification? **__________**
3. Thinking about sources of reimbursement, what percentage of treatment for youth clients is paid via each of the following? Please enter 0 for any items that do not apply. Your best guess is fine.
   1. Out-of-pocket/sliding scale fees _____%
   2. Private indemnity insurance _____%
   3. HMOs/PPOs _____%
   4. Medicaid _____%
   5. Juvenile justice contracts _____%
   6. Public assistance _____%
   7. Federal grant _____%
   8. State grant _____%
   9. Other (describe): ________________
4. Now, thinking about sources of referrals, what percentages of youth clients are referred to your center by the following? Please enter 0 for any items that do not apply. Your best guess is fine.
   1. Family _____%
   2. Schools _____%
   3. Juvenile justice system _____%
   4. The state’s child welfare system _____%
   5. Other treatment & healthcare providers _____%
   6. Self-referrals _____%
   7. Other (please specify): ________________

**We know that substance use treatment programs have been affected by the COVID-19 crisis, and we are interested in hearing how you and your A-CRA program changed your processes in light of these challenges.**

1. Did your A-CRA program experience any changes in practices due to COVID-19? Changes might include limiting the services to emergency-only cases, providing virtual/telehealth options, or other changes?
   1. No
   2. Yes
   3. N/A— We discontinued A-CRA prior to March 2020

IF QUESTION 58 = YES, CONTINUE. OTHERWISE, SKIP TO QUESTION 62.

1. Please rate how the following areas of practice at the A-CRA program were affected, ranging from no effect to completely different:

|  | No effect (0) | Small changes (1) | Moderate changes (2) | Large changes (3) | Completely different (4) |
| --- | --- | --- | --- | --- | --- |
| Referrals to the A-CRA program |  |  |  |  |  |
| A-CRA assessment services |  |  |  |  |  |
| A-CRA treatment services |  |  |  |  |  |
| Case management for A-CRA clients |  |  |  |  |  |
| Staffing, facilities, and other operations for the A-CRA program |  |  |  |  |  |

1. Did your A-CRA program re-opened for normal operations? We understand some aspects of your practice may remain changed for many reasons. If your program has mostly resumed pre-crisis operations, select yes. If your program continued operating under significantly limited or altered capacity, select no.
   1. No
   2. Yes
2. How long did it take for your A-CRA program to re-open for normal operations? ___________
3. Did you work remotely due to the COVID-19 crisis?
   1. No
   2. Yes, partly remote and partly in-person
   3. Yes, fully remote
4. Given the financial impact of COVID-19, did the organization where you work have to reduce staff hours, furlough employees, or reduce salary of employees?
   1. No
   2. Yes, but this did not directly affect A-CRA providers
   3. Yes, and this directly affected A-CRA providers
5. Did your organization start providing assessment, treatment, or case management services via any telehealth method (video, telephone, etc.) due to the COVID-19 crisis?
   1. No
   2. Yes, but this did not include A-CRA services
   3. Yes, and this included A-CRA services via telehealth
6. Please rate how each of the following COVID-related changes affected your organization’s ability to sustain A-CRA services, as compared to how things were before COVID:

|  | Much harder to sustain (1) | Somewhat harder (2) | A little harder (3) | About the same (4) | A little easier (5) | Somewhat easier (6) | Much easier to sustain (7) | Not applicable (0) |
| --- | --- | --- | --- | --- | --- | --- | --- | --- |
| Changes in referrals to the A-CRA program |  |  |  |  |  |  |  |  |
| Changes in A-CRA assessment services |  |  |  |  |  |  |  |  |
| Changes in A-CRA treatment services |  |  |  |  |  |  |  |  |
| Changes in case management for A-CRA clients |  |  |  |  |  |  |  |  |
| Changes in staffing for the A-CRA program |  |  |  |  |  |  |  |  |
| Changes in remote work |  |  |  |  |  |  |  |  |
| Changes in use of telehealth or virtual delivery of services |  |  |  |  |  |  |  |  |

1. Taken together, how much did COVID-related changes affect your organization’s ability to sustain A-CRA services, as compared to how things were before March 2020:
   1. Much harder to sustain
   2. Somewhat harder
   3. A little harder
   4. About the same
   5. A little easier
   6. Somewhat easier
   7. Much easier to sustain

**We are close to the end of the survey and just have a few more background questions about you.**

1. What is your gender?
   1. Male
   2. Female
   3. Non-binary/genderqueer
   4. Prefer to self-describe: ____________
2. How old are you? __________
3. Are you of Hispanic or Latino origin or descent?
   1. No
   2. Yes
4. What is your race? Please select one or more.
   1. White
   2. Black or African American
   3. Asian
   4. Native Hawaiian or Other Pacific Islander
   5. American Indian or Alaskan Native
   6. Prefer to self-describe: ____________
5. What is your highest level of education?
   1. No high school diploma or equivalent
   2. High school diploma or equivalent (GED)
   3. Some college, but no degree
   4. Associate’s degree
   5. Bachelor's degree
   6. Master's degree
   7. Doctoral degree or equivalent
   8. Prefer to self-describe: ____________
6. How many years of experience do you have in substance use counseling as a clinician? __________
7. How many years of experience do you have as a clinical supervisor for substance use counselors/clinicians?

__________

1. What type of provider are you?
   1. Certified Drug Abuse Counselor (CADAC)
   2. Clinical Psychologist
   3. Clinical Social Worker
   4. Licensed Chemical Dependency Counselor (LCDC)
   5. Psychiatric Nurse
   6. Psychiatrist
   7. Prefer to self-describe: ____________

**CONGRATULATIONS, YOU HAVE COMPLETED THE SURVEY!**

Please indicate the e-mail address where you would like your $50 Amazon e-gift card. Your e-mail will not be linked to your survey responses.

Email: ___________________________________

Confirm email: ______________________________

[OPTION TO SELECT] Do not send me the $50 e-gift card.

**Please hit SUBMIT to send us your answers!**

**ORGANIZATION-FOCUSED**

**Consent - CLINICIAN**

You are invited to complete a web survey because you are a clinician at a site that received funding from Center for Substance Abuse Treatment (CSAT) to deliver the Adolescent Community Reinforcement Approach (A-CRA). The web survey is part of a National Institutes of Health (NIH)-funded study to determine what factors help or hinder the delivery of A-CRA once CSAT support has ended.

***Who is conducting the study?***

The RAND Corporation, a non-profit research institute, is conducting the study in collaboration with Chestnut Health Systems. The study is funded by the National Institute of Alcoholism and Alcohol Abuse (NIAAA), which is part of the NIH. The Institutional Review Boards (IRBs) of both the RAND Corporation and Chestnut Health Systems have approved the study.

***What is the purpose of the study?***

The study purpose is to understand factors that influence the sustainability of A-CRA delivery after initial funding from CSAT ends, which can improve the quality of care for individuals with alcohol and other drug (AOD) abuse and dependence.

***What will I be asked to do?***

Your participation will involve completing a 15–30-minute online survey that asks questions about the leadership, staff, partnerships, and culture of your organization, your implementation of the A-CRA, barriers to delivering A-CRA, things that helped you deliver A-CRA, and whether you are still delivering A-CRA.

***Do I have to participate in the study?***

No. Your participation in the survey is voluntary. If you do participate, you will be able to skip questions that you prefer not to answer.

***What will you do with the information I provide?***

The information you provide in the online survey will be combined with the responses of other staff from your program and from over 75 other sites across the country. Results from the combined dataset will be published in journal articles that will be publicly available.

***What are the risks of participating, and how is the information I provide protected?***

No physical risks are involved in this study. Your primary risk will come from unauthorized disclosure of your answers. However, we have taken several steps to reduce this risk:

- All staff have been trained to understand the need for privacy
- The information about your identity and all the data with your answers are kept in two separate files that are only linked through a research ID
- This study’s records are also stored in locked, secure research offices
- Your individual responses will not be shared with anyone outside of the research team (this means no one at your organization, CSAT, or part of the A-CRA/ACC training team will see your individual results)
- All data will be analyzed in aggregate form only and no individual or program will be identifiable in any scientific report prepared by the research team.
- Although we request that you answer all items as honestly as you can, if you are not comfortable answering certain questions, you may skip over them.

We will not attribute information to specific individuals or programs.

***What are the benefits of participation in the study?***

After completion of the web survey, you will be given a $50 honorarium. In addition, your participation will help to better understand youth substance use treatment delivery, which can improve the quality of care for individuals with alcohol and other drug (AOD) abuse and dependence.

***Who can I contact if I have questions?***

We would be happy to answer any questions you might have about the study or your participation.

Sarah Hunter, Ph.D.

Behavioral Scientist, RAND Corporation

Tel: (310) 393-0411 x7244

Email: shunter@rand.org

RAND's toll-free number is 1-800-44-RAND-1.  If you have any questions or concerns about your rights as a research subject, you may also contact the Human Subjects Protection Committee at RAND, 1776 Main Street, P.O. Box 2138, Santa Monica, CA 90407-2138, 310-393-0411, ext. 6369.

**Consent - SUPERVISOR**

You are invited to complete a web survey because you are a clinical supervisor at a site that received funding from Center for Substance Abuse Treatment (CSAT) to deliver the Adolescent Community Reinforcement Approach (A-CRA). The web survey is part of a National Institutes of Health (NIH)-funded study to determine what factors help or hinder the delivery of A-CRA once CSAT support has ended.

***Who is conducting the study?***

The RAND Corporation, a non-profit research institute, is conducting the study in collaboration with Chestnut Health Systems. The study is funded by the National Institute of Alcoholism and Alcohol Abuse (NIAAA), which is part of the NIH. The Institutional Review Boards (IRBs) of both the RAND Corporation and Chestnut Health Systems have approved the study.

***What is the purpose of the study?***

The study purpose is to understand factors that influence the sustainability of A-CRA delivery after initial funding from CSAT ends, which can improve the quality of care for individuals with alcohol and other drug (AOD) abuse and dependence.

***What will I be asked to do?***

Your participation will involve completing a 15–30-minute online survey that asks questions about the leadership, staff, partnerships, and culture of your organization, how clinicians have implemented A-CRA, and things that helped or hindered delivery of A-CRA.

***Do I have to participate in the study?***

No. Your participation in the survey is voluntary. If you do participate, you will be able to skip questions that you prefer not to answer.

***What will you do with the information I provide?***

The information you provide in the online survey will be combined with the responses of other staff from your program and from over 75 other sites across the country. Results from the combined dataset will be published in journal articles that will be publicly available.

***What are the risks of participating, and how is the information I provide protected?***

No physical risks are involved in this study. Your primary risk will come from unauthorized disclosure of your answers. However, we have taken several steps to reduce this risk:

- All staff have been trained to understand the need for privacy
- The information about your identity and all the data with your answers are kept in two separate files that are only linked through a research ID
- This study’s records are also stored in locked, secure research offices
- Your individual responses will not be shared with anyone outside of the research team (this means no one at your organization, CSAT, or part of the A-CRA/ACC training team will see your individual results)
- All data will be analyzed in aggregate form only and no individual or program will be identifiable in any scientific report prepared by the research team.
- Although we request that you answer all items as honestly as you can, if you are not comfortable answering certain questions, you may skip over them.

We will not attribute information to specific individuals or programs.

***What are the benefits of participation in the study?***

After completion of the web survey, you will be given a $50 honorarium. In addition, your participation will help to better understand youth substance use treatment delivery, which can improve the quality of care for individuals with alcohol and other drug (AOD) abuse and dependence.

***Who can I contact if I have questions?***

We would be happy to answer any questions you might have about the study or your participation.

Sarah Hunter, Ph.D.

Behavioral Scientist, RAND Corporation

Tel: (310) 393-0411 x7244

Email: shunter@rand.org

RAND's toll-free number is 1-800-44-RAND-1.  If you have any questions or concerns about your rights as a research subject, you may also contact the Human Subjects Protection Committee at RAND, 1776 Main Street, P.O. Box 2138, Santa Monica, CA 90407-2138, 310-393-0411, ext. 6369.

**Sustainer Survey**

**The first set of questions are about A-CRA. Please select the correct answer for each of the following:**

1. The basic premise of CRA is that:
   1. with adequate skills training an individual can really combat any substance use problem.
   2. although will-power is ultimately still responsible for the majority of behavior change, at least a commitment to sobriety is also required.
   3. one must make a non-drinking/using lifestyle as rewarding as a drinking/using lifestyle.
   4. once an individual has a good job and the family is supportive again, the other pieces naturally fall into place.
   5. Don't know
2. A simple and easy way to monitor a youth's own report of progress in a number of areas is through:
   1. a behavioral contract.
   2. the CRA Happiness Scale.
   3. the CRA Functional Analysis for Non-Drinking Behaviors.
   4. the Self-Reminder To Be Nice form.
   5. Don't know
3. Why is it so important for a CRA therapist to always be looking for a youth's reinforcers?
   1. so that the therapist can be aware of the size of the youth's social support system at all times.
   2. because these may need to be resorted to and incorporated into the treatment plan if mild punishment does not appear to be working.
   3. because youths will be more likely to change their behavior if they feel they are being rewarded in doing so.
   4. because Community Reinforcement Approach implies that reinforcers can usually only be identified with the assistance of the youth's community.
   5. Don't know
4. Most behavioral and cognitive-behavioral treatments rely heavily on role-plays, despite the fact that it is often uncomfortable for clients (and therapists!) to do them. Role-plays are considered important because:
   1. they provide the therapist with valuable information about a youth's level of resistance to treatment.
   2. they are good for practice, because it is actually more difficult for a youth to do a role-play during a session than it is for them to try out the interaction in the natural environment.
   3. they offer valuable information about the quality of the therapeutic relationship and are early indicators of problematic transference.
   4. they give youths the opportunity to practice their new skills in a supportive environment with a person who can provide feedback.
   5. Don't know
5. The A-CRA Functional Analysis for Substance Use chart looks at the youth's short-term positive consequences for substance use. The reason for looking at these is:
   1. to be sure that the youth is fully aware from the start of what they are giving up by becoming substance free, and to be agreeable to it.
   2. to motivate the youth by reminding them of all the hardships endured (by the youth and their family) as a result of their use.
   3. to see what role substance use is serving, so that the individual can later be taught healthier ways to obtain these things.
   4. to recognize the types of obstacles that have successfully interfered with substance use in the past.
   5. Don't know
6. Why is it important to have both the youth and caregiver complete the Relationship Happiness Scale form during the Caregiver III and IV sessions?
   1. to enable the therapist to solve the families’ problems in one session.
   2. to get them to identify their weaknesses.
   3. to expose the source of unhappiness within the relationship.
   4. to have them practice communication and problem-solving skills through role-plays.
   5. Don't know
7. One CRA procedure entails: getting a client to role-play a phone call in session to an organization of interest (e.g. N.A.), and then having them actually place the call during the session, locating a contact person in the community for N.A., and calling them, and reviewing in the next session the experience of attending an N.A. meeting. The name of this procedure is:
   1. systematic encouragement
   2. reinforcer sampling
   3. cognitive restructuring
   4. communication skills training
   5. Don't know
8. Here are some common mistakes made by therapists who are implementing CRA. Which one is not a common mistake?
   1. losing sight of the youth's reinforcers.
   2. not emphasizing the importance of having a satisfying job.
   3. neglecting to monitor the youth's drinking and drug use.
   4. not checking for generalizability of skills.
   5. Don't know
9. In first selecting a category from the Happiness Scale to work on with a youth, it is advisable to begin with:
   1. an area that the youth express extreme unhappiness with, thereby demonstrating that you do not intend to shy away from the really difficult problems.
   2. an area that the youth states is really the main reason for seeking treatment in the first place, and consequently is the area most important to them.
   3. an area that is broad and really includes most of the youth's drug and non-drug problems, so that many different important issues get addressed right from the start.
   4. an area that the youth has reported mild-moderate (not extreme) unhappiness in, since it will probably provide an opportunity for the youth to experience some early success in therapy.
   5. Don't know
10. Therapists often spend very little time discussing a youth's social/recreational activities. However, it is important to do so because:
    1. many youths' social lives are built around alcohol/drugs, so they'll need help developing new drug-free pleasurable activities that compete with substance use.
    2. youth will assume that if you do not discuss this part of their lives, then they can probably get away with having an occasional beer during their recreational activities.
    3. even though a youth's social life is not particularly important to discuss from a therapeutic sense, it gives out the valuable message that you care enough about the youth to do it anyway.
    4. youths' behavior during their free time is really the best indicator we have of their personal values, and consequently it is often a predictor of whether they will remain committed to changing their using behavior.
    5. Don't know
11. Please rate your level of agreement with the following statements, ranging from strongly disagree to strongly agree.

|  | Strongly Disagree (1) | Somewhat Disagree (2) | Neither Agree nor Disagree (3) | Somewhat Agree (4) | Strongly Agree (5) |
| --- | --- | --- | --- | --- | --- |
| In general, A-CRA is more effective in creating attitudes that discourage substance use by clients than other treatment practices. |  |  |  |  |  |
| A-CRA is more effective in reducing substance use by clients than our current treatment practices. |  |  |  |  |  |
| A-CRA improves the overall quality of substance use treatment in this agency. |  |  |  |  |  |
| A-CRA is better than our previous treatment practices for substance use. |  |  |  |  |  |
| A-CRA is difficult to teach. |  |  |  |  |  |
| A-CRA is hard for therapists to understand. |  |  |  |  |  |
| A-CRA requires complex therapeutic strategies. |  |  |  |  |  |

1. Please rate your level of agreement with the following statements, ranging from strongly disagree to strongly agree.

|  | Strongly Disagree (1) | Somewhat Disagree (2) | Neither Agree nor Disagree (3) | Somewhat Agree (4) | Strongly Agree (5) |
| --- | --- | --- | --- | --- | --- |
| There is a definite need for A-CRA among my agency's clientele |  |  |  |  |  |
| There is a high level of interest for A-CRA among my agency's clientele |  |  |  |  |  |
| A-CRA is timely |  |  |  |  |  |
| A-CRA helps build partnerships |  |  |  |  |  |
| A-CRA has an impact on participants |  |  |  |  |  |

1. Please rate your level of agreement with the following statements, ranging from strongly disagree to strongly agree.

|  | Strongly Disagree (1) | Somewhat Disagree (2) | Neither Agree nor Disagree (3) | Somewhat Agree (4) | Strongly Agree (5) |
| --- | --- | --- | --- | --- | --- |
| Financing A-CRA is difficult |  |  |  |  |  |
| Recruiting participants for A-CRA is difficult |  |  |  |  |  |
| Recruiting staff to work on A-CRA is difficult |  |  |  |  |  |
| Finding time to prepare for A-CRA is difficult |  |  |  |  |  |
| Staff working on A-CRA have to learn new skills to deliver it |  |  |  |  |  |

**Do you strongly agree, agree somewhat, or disagree:**

1. Management wants to continue A-CRA
   1. Strongly Disagree
   2. Somewhat Disagree
   3. Neither Agree nor Disagree
   4. Somewhat Agree
   5. Strongly Agree
2. For each statement in the next set of questions, select the number that best indicates the extent to which the A-CRA treatment program has or does the following things.

|  | 1 To little or no extent (1) | 2 (2) | 3 (3) | 4 (4) | 5 (5) | 6 (6) | 7 To a very great extent (7) | NA Not able to answer (8) |
| --- | --- | --- | --- | --- | --- | --- | --- | --- |
| Program champions advocate for the A-CRA treatment program. |  |  |  |  |  |  |  |  |
| The A-CRA treatment program has strong champions with the ability to garner resources. |  |  |  |  |  |  |  |  |
| The A-CRA treatment program has political support within the larger organization. |  |  |  |  |  |  |  |  |
| The A-CRA treatment program has political support from outside of the organization. |  |  |  |  |  |  |  |  |
| The A-CRA treatment program has strong advocacy support. |  |  |  |  |  |  |  |  |
| The A-CRA treatment program exists in a supportive state economic climate. |  |  |  |  |  |  |  |  |
| The A-CRA treatment program implements policies to help ensure sustained funding |  |  |  |  |  |  |  |  |
| The A-CRA treatment program is funded through a variety of sources. |  |  |  |  |  |  |  |  |
| The A-CRA treatment program has a combination of stable and flexible funding. |  |  |  |  |  |  |  |  |
| The A-CRA treatment program has sustained funding. |  |  |  |  |  |  |  |  |

1. For each statement in the next set of questions, select the number that best indicates the extent to which the A-CRA treatment program has or does the following things.

|  | 1 To little or no extent (1) | 2 (2) | 3 (3) | 4 (4) | 5 (5) | 6 (6) | 7 To a very great extent (7) | NA Not able to answer (8) |
| --- | --- | --- | --- | --- | --- | --- | --- | --- |
| The A-CRA treatment program is well integrated into the operations of the organization. |  |  |  |  |  |  |  |  |
| Organizational systems are in place to support the various A-CRA treatment program needs. |  |  |  |  |  |  |  |  |
| Leadership effectively articulates the vision of the A-CRA treatment program to external partners. |  |  |  |  |  |  |  |  |
| Leadership efficiently manages staff and other resources. |  |  |  |  |  |  |  |  |
| The A-CRA treatment program has adequate staff to complete the program’s goals. |  |  |  |  |  |  |  |  |
| The A-CRA treatment program has the capacity for quality program evaluation. |  |  |  |  |  |  |  |  |
| The A-CRA treatment program reports short term and intermediate outcomes. |  |  |  |  |  |  |  |  |
| Evaluation results inform A-CRA treatment program planning and implementation. |  |  |  |  |  |  |  |  |
| A-CRA treatment program evaluation results are used to demonstrate successes to funders and other key stakeholders. |  |  |  |  |  |  |  |  |
| The A-CRA treatment program provides strong evidence to the public that the program works. |  |  |  |  |  |  |  |  |

1. For each statement in the next set of questions, select the number that best indicates the extent to which the A-CRA treatment program has or does the following things.

|  | 1 To little or no extent (1) | 2 (2) | 3 (3) | 4 (4) | 5 (5) | 6 (6) | 7 To a very great extent (7) | NA Not able to answer (8) |
| --- | --- | --- | --- | --- | --- | --- | --- | --- |
| The A-CRA treatment program periodically reviews the evidence base. |  |  |  |  |  |  |  |  |
| The A-CRA treatment program adapts strategies as needed. |  |  |  |  |  |  |  |  |
| The A-CRA treatment program adapts to new science. |  |  |  |  |  |  |  |  |
| The A-CRA treatment program proactively adapts to changes in the environment. |  |  |  |  |  |  |  |  |
| The A-CRA treatment program makes decisions about which components are ineffective and should not continue. |  |  |  |  |  |  |  |  |
| The A-CRA treatment program has communication strategies to secure and maintain public support. |  |  |  |  |  |  |  |  |
| A-CRA treatment program staff communicate the need for the program to the public. |  |  |  |  |  |  |  |  |
| The A-CRA treatment program is marketed in a way that generates interest. |  |  |  |  |  |  |  |  |
| The A-CRA treatment program increases community awareness of the issue. |  |  |  |  |  |  |  |  |
| The A-CRA treatment program demonstrates its value to the public. |  |  |  |  |  |  |  |  |

1. For each statement in the next set of questions, select the number that best indicates the extent to which the A-CRA treatment program has or does the following things.

|  | 1 To little or no extent (1) | 2 (2) | 3 (3) | 4 (4) | 5 (5) | 6 (6) | 7 To a very great extent (7) | NA Not able to answer (8) |
| --- | --- | --- | --- | --- | --- | --- | --- | --- |
| The A-CRA treatment program staff plans for future resource needs. |  |  |  |  |  |  |  |  |
| The A-CRA treatment program has a long-term financial plan. |  |  |  |  |  |  |  |  |
| The A-CRA treatment program has a sustainability plan. |  |  |  |  |  |  |  |  |
| The A-CRA treatment program's goals are understood by all stakeholders. |  |  |  |  |  |  |  |  |
| The A-CRA treatment program clearly outlines roles and responsibilities for all stakeholders. |  |  |  |  |  |  |  |  |

1. For each statement in the next set of questions, select the number that best indicates the extent to which the A-CRA treatment program has or does the following things.

|  | 1 To little or no extent (1) | 2 (2) | 3 (3) | 4 (4) | 5 (5) | 6 (6) | 7 To a very great extent (7) | NA Not able to answer (8) |
| --- | --- | --- | --- | --- | --- | --- | --- | --- |
| Diverse community organizations are invested in the success of the A-CRA treatment program. |  |  |  |  |  |  |  |  |
| The A-CRA treatment program communicates with community leaders. |  |  |  |  |  |  |  |  |
| Community leaders are involved with the A-CRA treatment program. |  |  |  |  |  |  |  |  |
| Community members are passionately committed to the A-CRA treatment program. |  |  |  |  |  |  |  |  |
| The community is engaged in the development of A-CRA treatment program goals. |  |  |  |  |  |  |  |  |

**The next set of questions are about staff at your agency.**

1. Have staff been assigned to implement the A-CRA treatment program?
   - 1. No
     2. Yes
     3. Not sure/Not applicable

IF QUESTION 20 = YES, CONTINUE. OTHERWISE, SKIP TO QUESTION 22.

1. For how many years have staff been assigned to implement the A-CRA treatment program?

Year(s) _____________ Month(s)

1. What is your best estimate of the number of staff who implement the A-CRA treatment program?
   1. None
   2. Few
   3. Most
   4. All
2. Has an administrative-level individual within your organization been actively involved in advocating for A-CRA’s continuation?
3. No
4. Yes
5. Not sure/Not applicable

IF QUESTION 23 = YES, CONTINUE. OTHERWISE, SKIP TO QUESTION 25.

1. For how many years have written goals and objectives related to A-CRA actually been followed?

Year(s) _____________ Month(s)

1. What is your best estimate of how active this administrative level individual has been in advocating for the A-CRA treatment program’s continuation?
   1. Not at all
   2. Minimally
   3. Moderately
   4. Very
2. Do staff in your organization, other than those actually implementing A-CRA, actively contribute to the A-CRA program’s operations?
   1. No
   2. Yes
   3. Not sure/Not applicable

IF QUESTION 26 = YES, CONTINUE. OTHERWISE, SKIP TO QUESTION 28.

1. For how many years have such staff in your organization actively contributed to the A-CRA treatment program’s operations?

Year(s) _____________ Month(s)

1. Of all the staff in your organization who could contribute to the operation of the A-CRA treatment program, what is your best estimate of the proportion that actually contribute to it?
   1. None
   2. Few
   3. Most
   4. All
2. Please rate your level of agreement with the following statements, ranging from strongly disagree to strongly agree.

|  | Strongly Disagree (1) | Somewhat Disagree (2) | Neither Agree nor Disagree (3) | Somewhat Agree (4) | Strongly Agree (5) |
| --- | --- | --- | --- | --- | --- |
| My supervisor has established clear standards for the implementation of evidence-based practice |  |  |  |  |  |
| My supervisor has developed a plan to facilitate implementation of evidence-based practice |  |  |  |  |  |
| My supervisor has removed obstacles to the implementation of evidence-based practice |  |  |  |  |  |
| My supervisor knows what they are talking about when it comes to evidence-based practice |  |  |  |  |  |
| My supervisor is knowledgeable about evidence-based practice |  |  |  |  |  |
| My supervisor is able to answer staff’s questions about evidence-based practice |  |  |  |  |  |
| My supervisor supports employee efforts to use evidence-based practice |  |  |  |  |  |
| My supervisor supports employee efforts to learn more about evidence-based practice |  |  |  |  |  |
| My supervisor recognizes and appreciates employee efforts toward successful implementation of evidence-based practice |  |  |  |  |  |
| My supervisor perseveres through the ups and downs of implementing evidence-based practice |  |  |  |  |  |
| My supervisor carries on through the challenges of implementing evidence-based practice |  |  |  |  |  |
| My supervisor reacts to critical issues regarding the implementation of evidence-based practice by openly and effectively addressing the problem(s) |  |  |  |  |  |

1. How important are the following goals for your agency? Please rate on a scale ranging from 1 (not important) to 5 (extremely important).

|  | Not important (1) | Somewhat unimportant (2) | Neutral (3) | Somewhat important (4) | Extremely important (5) |
| --- | --- | --- | --- | --- | --- |
| Improving quality of care for youths with substance use. |  |  |  |  |  |
| Improving client satisfaction with their care. |  |  |  |  |  |
| Improving productivity/efficiency. |  |  |  |  |  |
| Improving client clinical outcomes. |  |  |  |  |  |
| Improving clients with their own care. |  |  |  |  |  |
| Improving continuity of care. |  |  |  |  |  |

1. How important are the following goals for you? Please rate on a scale ranging from 1 (not important) to 5 (extremely important).

|  | Not important (1) | Somewhat unimportant (2) | Neutral (3) | Somewhat important (4) | Extremely important (5) |
| --- | --- | --- | --- | --- | --- |
| Having opportunities to use your skills and abilities better. |  |  |  |  |  |
| Getting recognition (i.e., praise, promotion, etc.) from your superiors. |  |  |  |  |  |
| Feeling that you have accomplished something worthwhile. |  |  |  |  |  |
| Making changes that improve care. |  |  |  |  |  |
| Spreading changes to other parts of the organization. |  |  |  |  |  |
| Gaining support for therapy changes. |  |  |  |  |  |
| Adapting A-CRA to your organization’s needs. |  |  |  |  |  |

**The next set of questions is about your agency.**

1. Which of the following services are provided by your agency? (MARK ALL THAT APPLY**)**
   1. Screening for substance use
   2. Screening for mental health
   3. Comprehensive substance use assessment or diagnosis
   4. Comprehensive mental health assessment or diagnosis (for example psychological or psychiatric evaluation and testing)
   5. Outreach to persons in the community who might need treatment
   6. Interim services for clients when admission is not possible
   7. Discharge planning
   8. Aftercare/continuing care
   9. Case management
   10. Social skills development
   11. Mentoring/peer support
   12. Substance use education
   13. Mental health education
   14. Self-help groups
   15. Medication Assisted Therapy (e.g., Antabuse, Naltrexone, Campral, Methadone, Buprenorphine)
   16. Smoking cessation medications (Nicotine replacement or non-nicotine)
   17. Medications for psychiatric disorders
2. What is the primary focus of this facility at this location?
   1. Substance use treatment services
   2. Mental health services
   3. Mix of mental health and substance use treatment services (neither is primary)
   4. General health care
   5. Other (Specify:) _______________
3. Is this facility operated by:
   1. A private for-profit organization
   2. A private non-profit organization
   3. State government
   4. Local, county, or community government
   5. Tribal government
   6. Federal government
4. What is the average length of stay in the treatment program? (Note: This is how long clients actually stay in the program.)

_________ (days) or _________ (weeks)

1. In total, how many youths can be in the treatment program at one time? _________ youths
2. What percent of the youths in the substance use treatment program have at least one other mental health diagnosis, besides substance use? ___________%
3. Thinking now about the design of this treatment program, how many of the following types of sessions does the average youth receive per week?
   - 1. Individual therapy sessions? ___________
     2. Psychoeducational sessions? ____________
     3. Group therapy sessions? _____________
4. What is the minimum age for admission? __________ years (Range: 5-20 years)
5. What is the maximum age for admission? __________ years (Range: 5-20 years)
6. How many **youth treatment supervisors** *left* your program/agency in the past 6 months (e.g., quit, fired, retired)? _________

IF ANSWER TO QUESTION 41>0, CONTINUE. OTHERWISE, SKIP TO QUESTION 43

1. How many of the youth treatment supervisors who left your agency in the past 6 months had been certified to deliver A-CRA? __________
2. How many **youth treatment supervisors** have been *hired* by your agency in the past 6 months? _____________

IF ANSWER TO QUESTION 43>0, CONTINUE. OTHERWISE, SKIP TO QUESTION 46.

1. How many of the youth treatment supervisors hired in the past 6 months are currently certified in A-CRA? ___________
2. How many of the youth treatment supervisors hired in the past 6 months are currently working toward A-CRA certification? ___________
3. How many **youth treatment clinicians** *left* your program/agency in the past 6 months (e.g., quit, fired, retired)? _____________

IF ANSWER TO QUESTION 46>0, CONTINUE. OTHERWISE, SKIP TO QUESTION 48.

1. How many of the youth treatment clinicians who left your agency in the past 6 months had been certified to deliver A-CRA?) ___________
2. How many **youth treatment clinicians** have been *hired* by your agency in the past 6 months? __________

IF ANSWER TO QUESTION 48>0, CONTINUE. OTHERWISE, SKIP TO QUESTION 51.

1. How many of these youth treatment clinicians hired in the past 6 months are currently certified in A-CRA? __________
2. How many of the youth treatment clinicians hired in the past 6 months are currently working toward A-CRA certification? _________

**The next set of this survey asks questions about how you see yourself as a clinician and how you see your youth treatment program.**

1. Please rate your level of agreement with the following statements, ranging from strongly disagree to strongly agree:

|  | Strongly Disagree (1) | Somewhat Disagree (2) | Neither Agree nor Disagree (3) | Somewhat Agree (4) | Strongly Agree (5) |
| --- | --- | --- | --- | --- | --- |
| You have the skills needed to conduct effective group counseling. |  |  |  |  |  |
| Some staff get confused about the main goals for the youth treatment program. |  |  |  |  |  |
| Staff here all get along very well. |  |  |  |  |  |
| Program staff understand how this youth treatment program fits as part of the treatment system in your community. |  |  |  |  |  |
| Treatment planning decisions for clients here often have to be revised by a counselor supervisor. |  |  |  |  |  |
| You frequently share your knowledge of counseling with other staff. |  |  |  |  |  |
| Management here fully trusts your professional judgment. |  |  |  |  |  |
| There is too much friction among staff members. |  |  |  |  |  |
| Ideas and suggestions from staff get fair consideration by program management. |  |  |  |  |  |
| Staff generally regard you as a valuable source of information. |  |  |  |  |  |
| The staff here always work together as a team. |  |  |  |  |  |
| Your duties are clearly related to the goals of this program. |  |  |  |  |  |
| You consistently plan ahead and carry out your plans. |  |  |  |  |  |
| You are under too many pressures to do your job effectively. |  |  |  |  |  |
| Counselors here are given broad authority in treating their own clients. |  |  |  |  |  |
| This program encourages and supports professional growth. |  |  |  |  |  |
| You read about new techniques and treatment information each month. |  |  |  |  |  |
| Staff here are always quick to help one another when needed. |  |  |  |  |  |
| Novel treatment ideas by staff are discouraged. |  |  |  |  |  |
| There are enough counselors here to meet current client needs. |  |  |  |  |  |
| You have enough opportunities to keep your counseling skills up to date. |  |  |  |  |  |
| Mutual trust and cooperation among staff in this program are strong. |  |  |  |  |  |
| You are willing to try new ideas even if some staff members are reluctant. |  |  |  |  |  |
| Learning and using new procedures are easy for you. |  |  |  |  |  |
| This program operates with clear goals and objectives. |  |  |  |  |  |
| Staff members often show signs of stress and strain. |  |  |  |  |  |
| You usually accomplish whatever you set your mind on. |  |  |  |  |  |
| It is easy to change procedures here to meet new conditions. |  |  |  |  |  |
| Counselors here often try out different techniques to improve their effectiveness. |  |  |  |  |  |
| The formal and informal communication channels here work very well. |  |  |  |  |  |
| You are sometimes too cautious or slow to make changes. |  |  |  |  |  |
| Staff members are given too many rules here. |  |  |  |  |  |
| Program staff are always kept well informed. |  |  |  |  |  |
| The heavy workload here reduces program effectiveness. |  |  |  |  |  |
| You regularly read professional journal articles or books on drug abuse treatment. |  |  |  |  |  |
| Other staff often ask your advice about program procedures. |  |  |  |  |  |
| More open discussions about program issues are needed here. |  |  |  |  |  |
| You frequently hear good staff ideas for improving treatment. |  |  |  |  |  |
| Other staff often ask for your opinions about counseling and treatment issues. |  |  |  |  |  |
| You are effective and confident in doing your job. |  |  |  |  |  |
| Everybody here does their fair share of work. |  |  |  |  |  |
| A larger support staff is needed to help meet program needs. |  |  |  |  |  |
| The general attitude here is to use new and changing technology. |  |  |  |  |  |
| You do a good job of regularly updating and improving your skills. |  |  |  |  |  |
| Staff members always feel free to ask questions and express concerns in this program. |  |  |  |  |  |
| You have the skills needed to conduct effective individual counseling. |  |  |  |  |  |
| Staff frustration is common here. |  |  |  |  |  |
| Management here has a clear plan for this program. |  |  |  |  |  |
| You often influence the decisions of other staff here. |  |  |  |  |  |
| You are encouraged here to try new and different techniques. |  |  |  |  |  |
| You are able to adapt quickly when you have to shift focus. |  |  |  |  |  |
| You are viewed as a leader by other staff here. |  |  |  |  |  |
| Frequent staff turnover is a problem for this program. |  |  |  |  |  |
| Counselors here are able to spend enough time with clients. |  |  |  |  |  |
| Support staff here have the skills they need to do their jobs. |  |  |  |  |  |
| Clinical staff here are well-trained. |  |  |  |  |  |

1. Thinking about sources of reimbursement, what percentage of treatment for youth clients is paid via each of the following?
   1. Out-of-pocket/sliding scale fees _____%
   2. Private indemnity insurance _____%
   3. HMOs/PPOs _____%
   4. Medicaid _____%
   5. Juvenile justice contracts _____%
   6. Public assistance _____%
   7. Federal grant _____%
   8. State grant _____%
   9. Other (describe): ________________
2. Now, thinking about sources of referrals, what percentages of youth clients are referred to your center by the following? Please enter 0 for any items that do not apply.
   1. Family _____%
   2. Schools _____%
   3. Juvenile justice system _____%
   4. The state’s child welfare system _____%
   5. Other treatment & healthcare providers _____%
   6. Self-referrals _____%
   7. Other (please specify): ________________

**We are close to the end of the survey and just have a few more background questions about you.**

1. Are you:
   1. Male
   2. Female
2. How old are you? __________
3. Are you of Hispanic or Latino origin or descent?
   1. No
   2. Yes
4. What is your race? Please select one or more.
   1. White
   2. Black or African American
   3. Asian
   4. Native Hawaiian or Other Pacific Islander
   5. American Indian or Alaskan Native
   6. NONE OF THESE (Please Describe): ____________
5. What is your highest level of education?
   1. No high school diploma or equivalent
   2. High school diploma or equivalent (GED)
   3. Some college, but no degree
   4. Associate’s degree
   5. Bachelor's degree
   6. Master's degree
   7. Doctoral degree or equivalent
   8. Other (medical assistant, RN, post-doctorate)
6. What type of provider are you?
   1. Certified Drug Abuse Counselor (CADAC)
   2. Clinical Psychologist
   3. Clinical Social Worker
   4. Licensed Chemical Dependency Counselor (LCDC)
   5. Psychiatric Nurse
   6. Psychiatrist
   7. NONE OF THESE (Please Describe): **____________**

**We will only use your contact information here to send your honorarium check. Your contact information will not be used for any other reason and will not be linked with your survey responses.**

Remember, you will also receive an additional $50 check after you upload 3 therapy session digital recordings to the YouthTx.org website so that we can better understand the treatment being delivered at your organization. If you need your username and password, please contact [NAME] at [PHONE] or [EMAIL]. Thank you!

**Non-Sustainer Survey**

1. We understand from our initial call that your agency stopped delivering A-CRA. When did you/your agency stop delivering A-CRA? If you do not know the exact date, please give your best estimate.

Month:

- 1. 01
  2. 02
  3. 03
  4. 04
  5. 05
  6. 06
  7. 07
  8. 08
  9. 09
  10. 10
  11. 11
  12. 12

Year:

6. 2006

7. 2007

8. 2008

9. 2009

10. 2010

11. 2011

12. 2012

13. 2013

1. DON'T KNOW
2. I WAS NOT EMPLOYED AT THIS AGENCY WHEN A-CRA TREATMENT DELIVERY WAS IMPLEMENTED
3. Are you familiar with Adolescent Community Reinforcement Approach (A-CRA)?
   - 1. No
     2. Yes

**The first set of questions are about A-CRA. Please select the correct answer for each of the following:**

1. The basic premise of CRA is that:
   1. with adequate skills training an individual can really combat any substance use problem.
   2. although will-power is ultimately still responsible for the majority of behavior change, at least a commitment to sobriety is also required.
   3. one must make a non-drinking/using lifestyle as rewarding as a drinking/using lifestyle.
   4. once an individual has a good job and the family is supportive again, the other pieces naturally fall into place.
   5. Don't know
2. A simple and easy way to monitor a youth's own report of progress in a number of areas is through:
   1. a behavioral contract.
   2. the CRA Happiness Scale.
   3. the CRA Functional Analysis for Non-Drinking Behaviors.
   4. the Self-Reminder To Be Nice form.
   5. Don't know
3. Why is it so important for a CRA therapist to always be looking for a youth's reinforcers?
   1. so that the therapist can be aware of the size of the youth's social support system at all times.
   2. because these may need to be resorted to and incorporated into the treatment plan if mild punishment does not appear to be working.
   3. because youths will be more likely to change their behavior if they feel they are being rewarded in doing so.
   4. because Community Reinforcement Approach implies that reinforcers can usually only be identified with the assistance of the youth's community.
   5. Don't know
4. Most behavioral and cognitive-behavioral treatments rely heavily on role-plays, despite the fact that it is often uncomfortable for clients (and therapists!) to do them. Role-plays are considered important because:
   1. they provide the therapist with valuable information about a youth's level of resistance to treatment.
   2. they are good for practice, because it is actually more difficult for a youth to do a role-play during a session than it is for them to try out the interaction in the natural environment.
   3. they offer valuable information about the quality of the therapeutic relationship and are early indicators of problematic transference.
   4. they give youths the opportunity to practice their new skills in a supportive environment with a person who can provide feedback.
   5. Don't know
5. The A-CRA Functional Analysis for Substance Use chart looks at the youth's short-term positive consequences for substance use. The reason for looking at these is:
   1. to be sure that the youth is fully aware from the start of what they are giving up by becoming substance free, and to be agreeable to it.
   2. to motivate the youth by reminding them of all the hardships endured (by the youth and their family) as a result of their use.
   3. to see what role substance use is serving, so that the individual can later be taught healthier ways to obtain these things.
   4. to recognize the types of obstacles that have successfully interfered with substance use in the past.
   5. Don't know
6. Why is it important to have both the youth and caregiver complete the Relationship Happiness Scale form during the Caregiver III and IV sessions?
   1. to enable the therapist to solve the families’ problems in one session.
   2. to get them to identify their weaknesses.
   3. to expose the source of unhappiness within the relationship.
   4. to have them practice communication and problem-solving skills through role-plays.
   5. Don't know
7. One CRA procedure entails: getting a client to role-play a phone call in session to an organization of interest (e.g. N.A.), and then having them actually place the call during the session, locating a contact person in the community for N.A., and calling them, and reviewing in the next session the experience of attending an N.A. meeting. The name of this procedure is:
   1. systematic encouragement
   2. reinforcer sampling
   3. cognitive restructuring
   4. communication skills training
   5. Don't know
8. Here are some common mistakes made by therapists who are implementing CRA. Which one is not a common mistake?
   1. losing sight of the youth's reinforcers.
   2. not emphasizing the importance of having a satisfying job.
   3. neglecting to monitor the youth's drinking and drug use.
   4. not checking for generalizability of skills.
   5. Don't know
9. In first selecting a category from the Happiness Scale to work on with a youth, it is advisable to begin with:
   1. an area that the youth express extreme unhappiness with, thereby demonstrating that you do not intend to shy away from the really difficult problems.
   2. an area that the youth states is really the main reason for seeking treatment in the first place, and consequently is the area most important to them.
   3. an area that is broad and really includes most of the youth's drug and non-drug problems, so that many different important issues get addressed right from the start.
   4. an area that the youth has reported mild-moderate (not extreme) unhappiness in, since it will probably provide an opportunity for the youth to experience some early success in therapy.
   5. Don't know
10. Therapists often spend very little time discussing a youth's social/recreational activities. However, it is important to do so because:
    1. many youths' social lives are built around alcohol/drugs, so they'll need help developing new drug-free pleasurable activities that compete with substance use.
    2. youth will assume that if you do not discuss this part of their lives, then they can probably get away with having an occasional beer during their recreational activities.
    3. even though a youth's social life is not particularly important to discuss from a therapeutic sense, it gives out the valuable message that you care enough about the youth to do it anyway.
    4. youths' behavior during their free time is really the best indicator we have of their personal values, and consequently it is often a predictor of whether they will remain committed to changing their using behavior.
    5. Don't know
11. Please think about the six-month period, right before A-CRA treatment delivery ended. Please rate your level of agreement with the following statements, ranging from strongly disagree to strongly agree.

|  | Strongly Disagree (1) | Somewhat Disagree (2) | Neither Agree nor Disagree (3) | Somewhat Agree (4) | Strongly Agree (5) |
| --- | --- | --- | --- | --- | --- |
| In general, A-CRA was more effective in creating attitudes that discourage substance use by clients than other treatment practices. |  |  |  |  |  |
| A-CRA was more effective in reducing substance use by clients than our current treatment practices. |  |  |  |  |  |
| A-CRA improved the overall quality of substance use treatment in this agency. |  |  |  |  |  |
| A-CRA was better than our previous treatment practices for substance use. |  |  |  |  |  |
| A-CRA was difficult to teach. |  |  |  |  |  |
| A-CRA was hard for therapists to understand. |  |  |  |  |  |
| A-CRA required complex therapeutic strategies. |  |  |  |  |  |

1. Again, please think about the six-month period right before A-CRA treatment delivery ended. Please rate your level of agreement with the following statements, ranging from strongly disagree to strongly agree.

|  | Strongly Disagree (1) | Somewhat Disagree (2) | Neither Agree nor Disagree (3) | Somewhat Agree (4) | Strongly Agree (5) |
| --- | --- | --- | --- | --- | --- |
| There was a definite need for A-CRA among my agency's clientele |  |  |  |  |  |
| There was a high level of interest for A-CRA among my agency's clientele |  |  |  |  |  |
| A-CRA was timely |  |  |  |  |  |
| A-CRA helped build partnerships |  |  |  |  |  |
| A-CRA had an impact on participants |  |  |  |  |  |

1. Again, please think about the six-month period right before A-CRA treatment delivery ended. Please rate your level of agreement with the following statements, ranging from strongly disagree to strongly agree.

|  | Strongly Disagree (1) | Somewhat Disagree (2) | Neither Agree nor Disagree (3) | Somewhat Agree (4) | Strongly Agree (5) |
| --- | --- | --- | --- | --- | --- |
| Financing A-CRA was difficult |  |  |  |  |  |
| Recruiting participants for A-CRA was difficult |  |  |  |  |  |
| Recruiting staff to work on A-CRA was difficult |  |  |  |  |  |
| Finding time to prepare for A-CRA was difficult |  |  |  |  |  |
| Staff working on A-CRA had to learn new skills to deliver it |  |  |  |  |  |

**Do you strongly agree, agree somewhat, or disagree:**

1. Management wanted to continue A-CRA
   1. Strongly Disagree
   2. Somewhat Disagree
   3. Neither Agree nor Disagree
   4. Somewhat Agree
   5. Strongly Agree
2. Again, please think about the six-month period right before A-CRA treatment delivery ended. For each statement in the next set of questions, select the number that best indicates the extent to which the A-CRA treatment program had or did the following things during that time.

|  | 1 To little or no extent (1) | 2 (2) | 3 (3) | 4 (4) | 5 (5) | 6 (6) | 7 To a very great extent (7) | NA Not able to answer (8) |
| --- | --- | --- | --- | --- | --- | --- | --- | --- |
| Program champions advocated for the A-CRA treatment program. |  |  |  |  |  |  |  |  |
| The A-CRA treatment program had strong champions with the ability to garner resources. |  |  |  |  |  |  |  |  |
| The A-CRA treatment program had political support within the larger organization. |  |  |  |  |  |  |  |  |
| The A-CRA treatment program had political support from outside of the organization. |  |  |  |  |  |  |  |  |
| The A-CRA treatment program had strong advocacy support. |  |  |  |  |  |  |  |  |
| The A-CRA treatment program existed in a supportive state economic climate. |  |  |  |  |  |  |  |  |
| The A-CRA treatment program implemented policies to help ensure sustained funding |  |  |  |  |  |  |  |  |
| The A-CRA treatment program was funded through a variety of sources. |  |  |  |  |  |  |  |  |
| The A-CRA treatment program had a combination of stable and flexible funding. |  |  |  |  |  |  |  |  |
| The A-CRA treatment program had sustained funding. |  |  |  |  |  |  |  |  |

1. Again, please think about the six-month period right before A-CRA treatment delivery ended. For each statement in the next set of questions, select the number that best indicates the extent to which the A-CRA treatment program had or did the following things during that time.

|  | 1 To little or no extent (1) | 2 (2) | 3 (3) | 4 (4) | 5 (5) | 6 (6) | 7 To a very great extent (7) | NA Not able to answer (8) |
| --- | --- | --- | --- | --- | --- | --- | --- | --- |
| The A-CRA treatment program was well integrated into the operations of the organization. |  |  |  |  |  |  |  |  |
| Organizational systems were in place to support the various A-CRA treatment program needs. |  |  |  |  |  |  |  |  |
| Leadership effectively articulated the vision of the A-CRA treatment program to external partners. |  |  |  |  |  |  |  |  |
| Leadership efficiently managed staff and other resources. |  |  |  |  |  |  |  |  |
| The A-CRA treatment program had adequate staff to complete the program’s goals. |  |  |  |  |  |  |  |  |
| The A-CRA treatment program had the capacity for quality program evaluation. |  |  |  |  |  |  |  |  |
| The A-CRA treatment program reported short term and intermediate outcomes. |  |  |  |  |  |  |  |  |
| Evaluation results informed A-CRA treatment program planning and implementation. |  |  |  |  |  |  |  |  |
| A-CRA treatment program evaluation results were used to demonstrate successes to funders and other key stakeholders. |  |  |  |  |  |  |  |  |
| The A-CRA treatment program provided strong evidence to the public that the program works. |  |  |  |  |  |  |  |  |

1. Again, please think about the six-month period right before A-CRA treatment delivery ended. For each statement in the next set of questions, select the number that best indicates the extent to which the A-CRA treatment program had or did the following things during that time.

|  | 1 To little or no extent (1) | 2 (2) | 3 (3) | 4 (4) | 5 (5) | 6 (6) | 7 To a very great extent (7) | NA Not able to answer (8) |
| --- | --- | --- | --- | --- | --- | --- | --- | --- |
| The A-CRA treatment program periodically reviewed the evidence base. |  |  |  |  |  |  |  |  |
| The A-CRA treatment program adapted strategies as needed. |  |  |  |  |  |  |  |  |
| The A-CRA treatment program adapted to new science. |  |  |  |  |  |  |  |  |
| The A-CRA treatment program proactively adapted to changes in the environment. |  |  |  |  |  |  |  |  |
| The A-CRA treatment program made decisions about which components were ineffective and should not continue. |  |  |  |  |  |  |  |  |
| The A-CRA treatment program had communication strategies to secure and maintain public support. |  |  |  |  |  |  |  |  |
| A-CRA treatment program staff communicated the need for the program to the public. |  |  |  |  |  |  |  |  |
| The A-CRA treatment program was marketed in a way that generated interest. |  |  |  |  |  |  |  |  |
| The A-CRA treatment program increased community awareness of the issue. |  |  |  |  |  |  |  |  |
| The A-CRA treatment program demonstrated its value to the public. |  |  |  |  |  |  |  |  |

1. Again, please think about the six-month period right before A-CRA treatment delivery ended. For each statement in the next set of questions, select the number that best indicates the extent to which the A-CRA treatment program had or did the following things during that time.

|  | 1 To little or no extent (1) | 2 (2) | 3 (3) | 4 (4) | 5 (5) | 6 (6) | 7 To a very great extent (7) | NA Not able to answer (8) |
| --- | --- | --- | --- | --- | --- | --- | --- | --- |
| The A-CRA treatment program staff planned for future resource needs. |  |  |  |  |  |  |  |  |
| The A-CRA treatment program had a long-term financial plan. |  |  |  |  |  |  |  |  |
| The A-CRA treatment program had a sustainability plan. |  |  |  |  |  |  |  |  |
| The A-CRA treatment program's goals were understood by all stakeholders. |  |  |  |  |  |  |  |  |
| The A-CRA treatment program clearly outlined roles and responsibilities for all stakeholders. |  |  |  |  |  |  |  |  |

1. Again, please think about the six-month period right before A-CRA treatment delivery ended. For each statement in the next set of questions, select the number that best indicates the extent to which the A-CRA treatment program had or did the following things during that time.

|  | 1 To little or no extent (1) | 2 (2) | 3 (3) | 4 (4) | 5 (5) | 6 (6) | 7 To a very great extent (7) | NA Not able to answer (8) |
| --- | --- | --- | --- | --- | --- | --- | --- | --- |
| Diverse community organizations were invested in the success of the A-CRA treatment program. |  |  |  |  |  |  |  |  |
| The A-CRA treatment program communicated with community leaders. |  |  |  |  |  |  |  |  |
| Community leaders were involved with the A-CRA treatment program. |  |  |  |  |  |  |  |  |
| Community members were passionately committed to the A-CRA treatment program. |  |  |  |  |  |  |  |  |
| The community was engaged in the development of A-CRA treatment program goals. |  |  |  |  |  |  |  |  |

**The next set of questions are about staff at your agency.**

1. During the six months right before A-CRA treatment delivery ended, were staff assigned to implement the A-CRA treatment program?
   - 1. No
     2. Yes
     3. Not sure/Not applicable

IF QUESTION 22 = YES, CONTINUE. OTHERWISE, SKIP TO QUESTION 24.

1. For how many years were staff assigned to implement the A-CRA treatment program?

Year(s) _____________ Month(s)

1. During the six months right before A-CRA treatment delivery ended, what is your best estimate of the number of staff who implemented the A-CRA treatment program?
   1. None
   2. Few
   3. Most
   4. All
2. During the six months right before A-CRA treatment delivery ended, was an administrative-level individual within your organization actively involved in advocating for A-CRA’s continuation?
   - 1. No
     2. Yes
     3. Not sure/Not applicable

IF QUESTION 25 = YES, CONTINUE. OTHERWISE, SKIP TO QUESTION 27.

1. For how many years were written goals and objectives related to A-CRA actually followed?

Year(s) _____________ Month(s)

1. During the six months right before A-CRA treatment delivery ended, what is your best estimate of how active this administrative level individual was in advocating for the A-CRA treatment program's continuation?
   1. Not at all
   2. Minimally
   3. Moderately
   4. Very
2. During the six months right before A-CRA treatment delivery ended, did staff in your organization, other than those who actually implemented A-CRA, actively contribute to the program’s operations?
   - 1. No
     2. Yes
     3. Not sure/Not applicable

IF QUESTION 28 = YES, CONTINUE. OTHERWISE, SKIP TO QUESTION 30.

1. For how many years did such staff in your organization actively contribute to the A-CRA treatment program’s operations?

Year(s) _____________ Month(s)

1. During the six months right before A-CRA treatment delivery ended, of all the staff in your organization who contributed to the operation of the A-CRA treatment program, what is your best estimate of the proportion that actually contributed to it?
   1. None
   2. Few
   3. Most
   4. All
2. Please rate your level of agreement with the following statements, ranging from strongly disagree to strongly agree.

|  | Strongly Disagree (1) | Somewhat Disagree (2) | Neither Agree nor Disagree (3) | Somewhat Agree (4) | Strongly Agree (5) |
| --- | --- | --- | --- | --- | --- |
| My supervisor has established clear standards for the implementation of evidence-based practice |  |  |  |  |  |
| My supervisor has developed a plan to facilitate implementation of evidence-based practice |  |  |  |  |  |
| My supervisor has removed obstacles to the implementation of evidence-based practice |  |  |  |  |  |
| My supervisor knows what they are talking about when it comes to evidence-based practice |  |  |  |  |  |
| My supervisor is knowledgeable about evidence-based practice |  |  |  |  |  |
| My supervisor is able to answer staff’s questions about evidence-based practice |  |  |  |  |  |
| My supervisor supports employee efforts to use evidence-based practice |  |  |  |  |  |
| My supervisor supports employee efforts to learn more about evidence-based practice |  |  |  |  |  |
| My supervisor recognizes and appreciates employee efforts toward successful implementation of evidence-based practice |  |  |  |  |  |
| My supervisor perseveres through the ups and downs of implementing evidence-based practice |  |  |  |  |  |
| My supervisor carries on through the challenges of implementing evidence-based practice |  |  |  |  |  |
| My supervisor reacts to critical issues regarding the implementation of evidence-based practice by openly and effectively addressing the problem(s) |  |  |  |  |  |

1. How important are the following goals for your agency? Please rate on a scale ranging from 1 (not important) to 5 (extremely important).

|  | Not important (1) | Somewhat unimportant (2) | Neutral (3) | Somewhat important (4) | Extremely important (5) |
| --- | --- | --- | --- | --- | --- |
| Improving quality of care for youths with substance use. |  |  |  |  |  |
| Improving client satisfaction with their care. |  |  |  |  |  |
| Improving productivity/efficiency. |  |  |  |  |  |
| Improving client clinical outcomes. |  |  |  |  |  |
| Improving clients with their own care. |  |  |  |  |  |
| Improving continuity of care. |  |  |  |  |  |

1. How important are the following goals for you? Please rate on a scale ranging from 1 (not important) to 5 (extremely important)

|  | Not important (1) | Somewhat unimportant (2) | Neutral (3) | Somewhat important (4) | Extremely important (5) |
| --- | --- | --- | --- | --- | --- |
| Having opportunities to use your skills and abilities better. |  |  |  |  |  |
| Getting recognition (i.e., praise, promotion, etc.) from your superiors. |  |  |  |  |  |
| Feeling that you have accomplished something worthwhile. |  |  |  |  |  |
| Making changes that improve care. |  |  |  |  |  |
| Spreading changes to other parts of the organization. |  |  |  |  |  |
| Gaining support for therapy changes. |  |  |  |  |  |
| Adapting A-CRA to your organization’s needs. |  |  |  |  |  |

**The next set of questions is about your agency.**

1. Which of the following services are provided by your agency? (MARK ALL THAT APPLY**)**
   1. Screening for substance use
   2. Screening for mental health
   3. Comprehensive substance use assessment or diagnosis
   4. Comprehensive mental health assessment or diagnosis (for example psychological or psychiatric evaluation and testing)
   5. Outreach to persons in the community who might need treatment
   6. Interim services for clients when admission is not possible
   7. Discharge planning
   8. Aftercare/continuing care
   9. Case management
   10. Social skills development
   11. Mentoring/peer support
   12. Substance use education
   13. Mental health education
   14. Self-help groups
   15. Medication Assisted Therapy (e.g., Antabuse, Naltrexone, Campral, Methadone, Buprenorphine)
   16. Smoking cessation medications (Nicotine replacement or non-nicotine)
   17. Medications for psychiatric disorders
2. What is the primary focus of this facility at this location?
   1. Substance use treatment services
   2. Mental health services
   3. Mix of mental health and substance use treatment services (neither is primary)
   4. General health care
   5. Other (Specify:) _______________
3. Is this facility operated by:
   1. A private for-profit organization
   2. A private non-profit organization
   3. State government
   4. Local, county, or community government
   5. Tribal government
   6. Federal government
4. What is the average length of stay in the treatment program? (Note: This is how long clients actually stay in the program.)

_________ (days) or _________ (weeks)

1. In total, how many youths can be in the treatment program at one time? _________ youths
2. What percent of the youths in the substance use treatment program have at least one other mental health diagnosis, besides substance use? ___________%
3. Thinking now about the design of this treatment program, how many of the following types of sessions does the average youth receive per week?
   - 1. Individual therapy sessions? ___________
     2. Psychoeducational sessions? ____________
     3. Group therapy sessions? _____________
4. What is the minimum age for admission? __________ years (Range: 5-20 years)
5. What is the maximum age for admission? __________ years (Range: 5-20 years)
6. How many **youth treatment supervisors** *left* your program/agency in the past 6 months (e.g., quit, fired, retired)? _________

IF ANSWER TO QUESTION 43>0, CONTINUE. OTHERWISE, SKIP TO QUESTION 45.

1. How many of the youth treatment supervisors who left your agency in the past 6 months had been certified to deliver A-CRA? __________
2. How many **youth treatment supervisors** have been *hired* by your agency in the past 6 months? _____________

IF ANSWER TO QUESTION 45>0, CONTINUE. OTHERWISE, SKIP TO QUESTION 48.

1. How many of the youth treatment supervisors hired in the past 6 months are currently certified in A-CRA? ___________
2. How many of the youth treatment supervisors hired in the past 6 months are currently working toward A-CRA certification? ___________
3. How many **youth treatment clinicians** *left* your program/agency in the past 6 months (e.g., quit, fired, retired)? _____________

IF ANSWER TO QUESTION 48>0, CONTINUE. OTHERWISE, SKIP TO QUESTION 50.

1. How many of the youth treatment clinicians who left your agency in the past 6 months had been certified to deliver A-CRA?) ___________
2. How many **youth treatment clinicians** have been *hired* by your agency in the past 6 months? __________

IF ANSWER TO QUESTION 50>0, CONTINUE. OTHERWISE, SKIP TO QUESTION 53.

1. How many of these youth treatment clinicians hired in the past 6 months are currently certified in A-CRA? __________
2. How many of the youth treatment clinicians hired in the past 6 months are currently working toward A-CRA certification? _________

**The next set of this survey asks questions about how you see yourself as a clinician and how you see your youth treatment program.**

1. Please rate your level of agreement with the following statements, ranging from strongly disagree to strongly agree:

|  | Strongly Disagree (1) | Somewhat Disagree (2) | Neither Agree nor Disagree (3) | Somewhat Agree (4) | Strongly Agree (5) |
| --- | --- | --- | --- | --- | --- |
| You have the skills needed to conduct effective group counseling. |  |  |  |  |  |
| Some staff get confused about the main goals for the youth treatment program. |  |  |  |  |  |
| Staff here all get along very well. |  |  |  |  |  |
| Program staff understand how this youth treatment program fits as part of the treatment system in your community. |  |  |  |  |  |
| Treatment planning decisions for clients here often have to be revised by a counselor supervisor. |  |  |  |  |  |
| You frequently share your knowledge of counseling with other staff. |  |  |  |  |  |
| Management here fully trusts your professional judgment. |  |  |  |  |  |
| There is too much friction among staff members. |  |  |  |  |  |
| Ideas and suggestions from staff get fair consideration by program management. |  |  |  |  |  |
| Staff generally regard you as a valuable source of information. |  |  |  |  |  |
| The staff here always work together as a team. |  |  |  |  |  |
| Your duties are clearly related to the goals of this program. |  |  |  |  |  |
| You consistently plan ahead and carry out your plans. |  |  |  |  |  |
| You are under too many pressures to do your job effectively. |  |  |  |  |  |
| Counselors here are given broad authority in treating their own clients. |  |  |  |  |  |
| This program encourages and supports professional growth. |  |  |  |  |  |
| You read about new techniques and treatment information each month. |  |  |  |  |  |
| Staff here are always quick to help one another when needed. |  |  |  |  |  |
| Novel treatment ideas by staff are discouraged. |  |  |  |  |  |
| There are enough counselors here to meet current client needs. |  |  |  |  |  |
| You have enough opportunities to keep your counseling skills up to date. |  |  |  |  |  |
| Mutual trust and cooperation among staff in this program are strong. |  |  |  |  |  |
| You are willing to try new ideas even if some staff members are reluctant. |  |  |  |  |  |
| Learning and using new procedures are easy for you. |  |  |  |  |  |
| This program operates with clear goals and objectives. |  |  |  |  |  |
| Staff members often show signs of stress and strain. |  |  |  |  |  |
| You usually accomplish whatever you set your mind on. |  |  |  |  |  |
| It is easy to change procedures here to meet new conditions. |  |  |  |  |  |
| Counselors here often try out different techniques to improve their effectiveness. |  |  |  |  |  |
| The formal and informal communication channels here work very well. |  |  |  |  |  |
| You are sometimes too cautious or slow to make changes. |  |  |  |  |  |
| Staff members are given too many rules here. |  |  |  |  |  |
| Program staff are always kept well informed. |  |  |  |  |  |
| The heavy workload here reduces program effectiveness. |  |  |  |  |  |
| You regularly read professional journal articles or books on drug abuse treatment. |  |  |  |  |  |
| Other staff often ask your advice about program procedures. |  |  |  |  |  |
| More open discussions about program issues are needed here. |  |  |  |  |  |
| You frequently hear good staff ideas for improving treatment. |  |  |  |  |  |
| Other staff often ask for your opinions about counseling and treatment issues. |  |  |  |  |  |
| You are effective and confident in doing your job. |  |  |  |  |  |
| Everybody here does their fair share of work. |  |  |  |  |  |
| A larger support staff is needed to help meet program needs. |  |  |  |  |  |
| The general attitude here is to use new and changing technology. |  |  |  |  |  |
| You do a good job of regularly updating and improving your skills. |  |  |  |  |  |
| Staff members always feel free to ask questions and express concerns in this program. |  |  |  |  |  |
| You have the skills needed to conduct effective individual counseling. |  |  |  |  |  |
| Staff frustration is common here. |  |  |  |  |  |
| Management here has a clear plan for this program. |  |  |  |  |  |
| You often influence the decisions of other staff here. |  |  |  |  |  |
| You are encouraged here to try new and different techniques. |  |  |  |  |  |
| You are able to adapt quickly when you have to shift focus. |  |  |  |  |  |
| You are viewed as a leader by other staff here. |  |  |  |  |  |
| Frequent staff turnover is a problem for this program. |  |  |  |  |  |
| Counselors here are able to spend enough time with clients. |  |  |  |  |  |
| Support staff here have the skills they need to do their jobs. |  |  |  |  |  |
| Clinical staff here are well-trained. |  |  |  |  |  |

1. Thinking about sources of reimbursement, what percentage of treatment for youth clients is paid via each of the following?
   1. Out-of-pocket/sliding scale fees _____%
   2. Private indemnity insurance _____%
   3. HMOs/PPOs _____%
   4. Medicaid _____%
   5. Juvenile justice contracts _____%
   6. Public assistance _____%
   7. Federal grant _____%
   8. State grant _____%
   9. Other (describe): ________________
2. Now, thinking about sources of referrals, what percentages of youth clients are referred to your center by the following? Please enter 0 for any items that do not apply.
   1. Family _____%
   2. Schools _____%
   3. Juvenile justice system _____%
   4. The state’s child welfare system _____%
   5. Other treatment & healthcare providers _____%
   6. Self-referrals _____%
   7. Other (please specify): ________________

**We are close to the end of the survey and just have a few more background questions about you.**

1. Are you:
   - 1. Male
     2. Female
2. How old are you? __________
3. Are you of Hispanic or Latino origin or descent?
   - 1. No
     2. Yes
4. What is your race? Please select one or more.
   1. White
   2. Black or African American
   3. Asian
   4. Native Hawaiian or Other Pacific Islander
   5. American Indian or Alaskan Native
   6. NONE OF THESE (Please Describe): ____________
5. What is your highest level of education?
   1. No high school diploma or equivalent
   2. High school diploma or equivalent (GED)
   3. Some college, but no degree
   4. Associate’s degree
   5. Bachelor's degree
   6. Master's degree
   7. Doctoral degree or equivalent
   8. Other (medical assistant, RN, post-doctorate)
6. What type of provider are you?
   1. Certified Drug Abuse Counselor (CADAC)
   2. Clinical Psychologist
   3. Clinical Social Worker
   4. Licensed Chemical Dependency Counselor (LCDC)
   5. Psychiatric Nurse
   6. Psychiatrist
   7. NONE OF THESE (Please Describe): **____________**

**We will only use your contact information here to send your honorarium check. Your contact information will not be used for any other reason and will not be linked with your survey responses.**

Remember, you will also receive an additional $50 check after you upload 3 therapy session digital recordings to the YouthTx.org website so that we can better understand the treatment being delivered at your organization. If you need your username and password, please contact [NAME] at [PHONE] or [EMAIL]. Thank you!
