# Supplementary material for: Comparing organization-focused and state-focused financing strategies on provider-level reach of a youth substance use treatment model: a mixed-method study
Source: Implement Sci. 2023 Oct 12;18:50. doi: 10.1186/s13012-023-01305-z (PMC10571404; doi:10.1186/s13012-023-01305-z)
Supplement: Supplementary file 4 — Additional file 4. Supplemental Analyses for Quantitative Analysis of Provider-Level Reach Outcomes. [file 13012_2023_1305_MOESM4_ESM.docx]

**Supplemental Analyses for** **Quantitative Analysis of Provider-Level Reach Outcomes**

To compare reach rates between organization-focused and state-focused grant types, we fit a series of multivariable linear regression models with organization-level reach rates (any, first-level, full, supervisor) as the dependent variables. Each regression included grant type and several covariates (turnover rate, number of individuals trained, number of supervisors who pursued certification, grant end date, quarters of grant funding). We also tested alternate model specifications to determine if the results depended on model specification (i.e., differed from the primary analysis). Specifications tested included use of a beta regression model (appropriate for outcomes bounded between 0 and 1, like reach rates), robust standard errors, and inclusion of state-level fixed effects (with or without clustered standard errors).

Below, Table S1 reports the results of each alternate specification of the regression model. Findings changed minimally compared to the regression model in the primary analysis, so we focused on the latter results in the manuscript (see Table 5).

**Table S1: *Results from Regression Models with Different Model Specifications***

| Sensitivity Analysis | Association Between State-Focused Grant Variable^a^ and A-CRA Provider-Level Reach Outcome (*β*, *SE*) | |
| --- | --- | --- |
|  |  |  |
|  | Any Certification | First-Level Certification |
|  |  |  |
| OLS regression with robust standard errors | -0.27 (.11)  *p* = .01  *n* = 164 | -0.23 (.11)  *p* = .04  *n* = 164 |
|  |  |  |
| OLS regression with state fixed effects | -0.38 (.16)  *p* = .02  *n* = 164 | -0.32 (.16)  *p* = .05  *n* = 164 |
|  |  |  |
| OLS regression with state fixed effects and robust standard errors | -0.38 (.14)  *p* < .01^  *n* = 164 | -0.32 (.15)  *p* = .03  *n* = 164 |
|  |  |  |
| OLS regression with state fixed effects and clustered standard errors | -0.38 (.16)  *p* = .03  *n* = 164 | -0.32 (.20)  *p* = .11^  *n* = 164 |
|  |  |  |

*Note*. A-CRA = Adolescent Community Reinforcement Approach.

^a^The association between the state-focused grant (vs. organization focused grant) variable and the specified reach outcome variable in the regression model.

^indicates that the association between grant type on the specified reach outcome variable differed in statistical significance, compared to the same association in the main analysis (reported in Table 5).

**Sensitivity Analyses for Threats to Quantitative Analysis of Reach Outcomes**

We also conducted a series of sensitivity analyses that characterized threats to internal validity under our quasi-experimental design. Specifically, we examined (a) the potential influence of observed secular trends on grant outcomes and (b) patterns of findings across sub-samples that lent insight into the impacts of grant type. Note that unobserved secular trends were already controlled using grant end date as a covariate in the main analysis (see Table 5).

***Non-equivalent dependent variable***

To examine secular trends that may be confounded with grant type, we analyzed a non-equivalent dependent variable,^41,42^ which is an outcome that (1) *was not* influenced by grant type but (2) *would be* influenced by other factors that promote EBP implementation at SUD treatment organizations. Our selected dependent variable was delivery of buprenorphine, an evidence-based medication for opioid use disorder, to capture secular time trends among factors influencing EBP implementation at SUD treatment organizations. As is customary for the logic of non-equivalent dependent variables, our hypothesis was that grant type (organization-focused vs. state-focused) would not be a significant predictor of an organizations’ likelihood of delivering buprenorphine.

We measured buprenorphine delivery by matching listings in the SAMHSA National Directory of Drug and Alcohol Abuse Treatment Facilities based on responses to the previous year’s National Survey of Substance Abuse Treatment Services^52^ to participating treatment organizations for the year that organization’s grant ended (i.e., 2009-2021). If we could not match to the exact grant end year, we substituted the outcome from the most recent previous year up to four years prior. We located a match for *n*=109 organizations, a 66% match rate.

The regression model for the non-equivalent dependent variable was specified identically to the model for reach outcome analyses, but (a) with buprenorphine delivery substituted as the outcome variable and (b) using logistic regression due to the outcome being binary (Y/N). Per our published study protocol,^25^ we considered using other non-equivalent dependent variables from the SAMHSA directory data, but no other discrete EBPs were tracked in that data for the full range of years needed; and we considered including additional covariates, such as opioid overdose death rates in the treatment organization county, but determined that the model would be more interpretable if it matched the main analysis as closely as possible.

Table S2 below reports the non-equivalent dependent variable analysis, as well as descriptive statistics for buprenorphine delivery rates by grant type (15% of earlier organization-focused grants vs. 25% of later state-focused grants). In the logistic regression model, grant type was not associated with likelihood of an organization delivering buprenorphine (*OR* = 0.12, *p* = .12), as predicted. In contrast, the likelihood was significantly higher for organizations whose grants ended later (*OR* = 1.14, *p* < .01), which was not true for A-CRA reach outcomes (see Table 5). These findings suggest that this non-equivalent variable captured the intended secular time trends among factors influencing EBP use for SUDs, and that the observed differences in reach rates by grant type were unlikely to have resulted from the same trends. Because the association between grant type and the non-equivalent dependent variable was not significant, no adjustments were needed to the main analysis reported in Table 5.

**Table S2:**

***Sensitivity Analysis Results from Regression Model with Non-Equivalent Dependent Variable***

| Outcome Variable | Descriptive Statistics (*M* and *SD*)^a^  by Grant Type | |
| --- | --- | --- |
|  | Organization-focused  (*n* = 54) | State-focused  (*n* = 55) |
|  |  |  |
| Buprenorphine delivery status (Y/N) in year grant ended | 15% (36%) | 25% (44%) |
|  |  |  |

| Variable | Estimated association (*OR*, *SE*)^b^  with buprenorphine delivery status |
| --- | --- |
|  |  |
| State-focused grant (vs. organization-focused) | 0.12 (.16)  *p =* .12 |
|  |  |
| # providers trained | 0.98 (.12)  *p =* .86 |
|  |  |
| # supervisors who pursued certification | 0.96 (.26)  *p =* .87 |
|  |  |
| Turnover rate | 1.34 (1.39)  *p =* .78 |
|  |  |
| Grant end date | 1.14 (.06)  *p <* .01^**^ |
|  |  |
| Length of grant period | 0.91 (.06)  *p =* .13 |
|  |  |
| Constant | 0.00 (.00)  *p =*.01^*^ |
|  |  |
| *R^2^* | .14 |
|  |  |

*Note*. *n* = 109 for this analysis. ^*^ = significant at p < .05 level; ^**^ = significant at p < .01 level.

OR = odds ratio. ^a^We coded this variable as Yes (Y) = 1 and No (N) = 0, so descriptive statistics reflect the proportion of the sample with a 1 (Yes) value. ^b^This analysis used logistic regression, rather than simple linear regression, because the outcome was a binary variable (Y/N).

***Sensitivity analyses of sub-samples***

The remaining sensitivity analyses involved recalculating the reach outcome variables for sub-samples to help understand the impact of organization-focused versus state-focused grant type. We calculated reach for (a) treatment organizations from 11 states with both organization- and state-focused grants (*n* = 67), to permit a more direct comparison controlling for state factors; (b) demonstration sites separately from other state-focused grantees, given that demonstration sites functioned similarly to organization-focused grantees in many respects (the main difference being they worked with the state agency for A-CRA implementation rather than directly with SAMHSA); (c) including or excluding observations from organizations that trained providers under both grant types; we decided to only include the first (organization-focused) observation of outcomes from such agencies in the main model, but wanted to examine the impact of our decision, so we tested models that either dropped all 11 organizations from the sample or included state-focused observations from 6 organizations whose state-focused grant did not overlap with their organization-focused grant; and (d) excluding observations where the COVID-19 pandemic overlapped with the state-focused grant period (3 states, 14 organizations).

Table S3 details how, compared to the main analysis (see Table 5), the association between grant type and reach was (a) similar in states with both grant types; (b) only present for non-demonstration sites, with state-focused demonstration sites having equivalent outcomes to organization-focused sites (*p*s = <.01 vs. .38); (c) no longer significant if treatment organizations that implemented under both grant types were entirely excluded (*p*=.06; but note that this requires excluding valid observations), but remained similar if those state-focused observations were included; and (d) similar when states impacted by the COVID-19 pandemic during A-CRA implementation were excluded. These findings suggest that the observed differences in provider-level reach outcomes did not depend on the characteristics of states or organizations included in the sample, but instead were strongly associated with grant type (reinforced by the difference between demonstration and non- demonstration sub-samples of the state-focused grantees).

**Table S3: *Sensitivity Analysis Results from Regression Models with Sub-Samples***

| Sensitivity Analysis | Association Between State-Focused Grant Variable^a^ and A-CRA Provider-Level Reach Outcome (*β*, *SE*) | | |
| --- | --- | --- | --- |
|  |  |  | |
|  | Any Certification | | First-Level Certification |
|  |  | |  |
| States with both organization- and state-focused grants only  (11 states) | -0.34 (.19)  *p* = .09^  *n* = 67 | | -0.25 (.20)  *p* = .22^  *n* = 67 |
|  |  | |  |
| Demonstration Sites only in state-focused grantee sample | -0.10 (.12)  *p* = .38^  *n* = 125 | | -0.08 (.12)  *p* = .52^  *n* = 125 |
|  |  | |  |
| Non-Demonstration Sites only in state-focused grantee sample | -0.42 (.11)  *p* < .01  *n* = 121 | | -0.37 (.11)  *p* < .01  *n* = 121 |
|  |  | |  |
| Excluding organization-focused grantees’ outcomes if also in state-focused sample | -0.22 (.11)  *p* = .06^  *n* = 153 | | -0.17 (.11)  *p* = .13^  *n* = 153 |
|  |  | |  |
| Including organization-focused grantees’ outcomes in state-focused sample as well | -0.29 (.11)  *p* = .01  *n* = 170 | | -0.24 (.11)  *p* = .02  *n* = 170 |
|  |  | |  |
| Excluding state-focused grantees whose grant period overlapped with the COVID-19 pandemic  (3 states) | -0.36 (.13)  *p* < .01  *n* = 150 | | -0.33 (.12)  *p* < .01  *n* = 150 |
|  |  | |  |

*Note*. A-CRA = Adolescent Community Reinforcement Approach.

^a^The association between the state-focused grant (vs. organization focused grant) variable and the specified reach outcome variable in the regression model.

^ indicates that the association between grant type on the specified reach outcome variable differed in statistical significance, compared to the same association in the main analysis (reported in Table 5).
