# Supplementary material for: Comparing organization-focused and state-focused financing strategies on provider-level reach of a youth substance use treatment model: a mixed-method study
Source: Implement Sci. 2023 Oct 12;18:50. doi: 10.1186/s13012-023-01305-z (PMC10571404; doi:10.1186/s13012-023-01305-z)
Supplement: Supplementary file 5 — Additional file 5. Expanded Summary of All Barriers and Facilitators to A-CRA Provider-Level Reach Outcomes. [file 13012_2023_1305_MOESM5_ESM.docx]

Table S4

*Expanded Summary of All Barriers and Facilitators to A-CRA Provider-Level Reach Outcomes from Qualitative Interviews*

| EPIS Domain | Determinant Code (see Note for key) | Brief Description | Interview Types with Code (see Note for key) | Exemplar Quotes |
| --- | --- | --- | --- | --- |
| Innovation  Innovation, continued  Innovation, continued  Innovation, continued | Perceptions of Content/Tools (F) | Some A-CRA procedures (i.e., the happiness scale, communication skills, and problem solving) were mentioned as valuable skills that can be applied in many areas of clients’ lives, promoting buy-in and use of the model. | OF, SF; P, A | It's not all concretely substance based, but [A-CRA procedures] also incorporating other elements, like the family components and those mood components when we start to do something like the happiness scale…It's specific to substance use, but it's broad enough to touch other areas that may impact your substance use. (SF, P) |
|  | Structured Format (F/B) | Some providers expressed that the procedures were too structured and inflexible. They perceived they could not deliver A-CRA in ways that were responsive to the needs of each client, ultimately making them use it less frequently. Other respondents reported liking the structured format of the sessions since it helped them know exactly what to do and made the components easy to understand. | OF, SF; P, A | [A-CRA] provides the necessary structure to ensure that you're accomplishing goals and moving forward while also being flexible and allowing the client to dictate what issues they want to resolve first … We've used other models prior to using this one that weren't quite as flexible and that didn't seem quite as responsive to the clients' needs. (SF, P)  I think that for the population we were serving it seemed [A-CRA] was very prescriptive and rigid and sometimes I think we had to meet [teens] where they were at, even if it meant not following the steps as outlined (SF, P) |
|  | Evidence Strength and Quality (F)  (*continues*)  (*continued from previous page*) | Respondents noted that A-CRA was supported due to the fact it is an evidence-based practice. State agencies and treatment organizations tended to promote evidence-based practices, and many providers preferred using them. | OF, SF; P, A | I know through the county, [evidence-based practice] is highly recommended and used most effectively. … because [A-CRA] is evidence-based and it’s been done everywhere, and we have seen how effective it can be, so there [are] elements that we would still implement. (OF, P) |
|  | Format and Support of Training and Certification Process (F) | The format of the initial A-CRA training sessions was beneficial and well liked among providers; many respondents expressed positive attitudes regarding the content and structure of the sessions. Many providers also noted that Chestnut Health System’s support and coaching after training helped them better understand and deliver A-CRA. | OF, SF; P, A | I think we had a really positive experience working with Chestnut. The biggest thing that we experienced with this particular grant was that… we were not prepared for the level of and intensity of the training and monitoring. It was really good. We never experienced anything that was that in-depth… It's a huge time commitment, but now we can look back and say it was probably the best quality training that we could have asked for. I think it was certainly worthwhile. (OF, P) |
|  | Burden and Complexity of Training and Certification Process (B) | Many respondents noted that the certification and supervision process was frustrating in terms of time and effort. This complex process was described as one of the reasons that some providers did not complete their certification or fully implement A-CRA. | OF, SF; P, A | I never finished the [first-level] certification. The grant was too labor intensive. That's why there was so much turnover. It was too much work. … and with the [session] recording, and uploading [for fidelity monitoring] it was a nightmare… Nobody liked it because it took so much time to do anything, and the evaluations were too detailed. (OF, P) |
|  | Good Fit with Population (F) | A-CRA was perceived as a great option for communities that prioritize harm reduction and want to provide their youth with tools to reduce their substance use and become independent. | OF, SF; P, A | [A-CRA helps youth] see the patterns of their use and then helping them really focus on those pro-social activities, which I think is really what the ACRA model focuses on, is not necessarily trying to tell kids to quit, but finding out what the purpose of their use is and then replacing it with something that's more pro-social, has less consequences (SF, P) |
|  | Lack of Fit with Population (B) | Some providers noted A-CRA was not culturally appropriate for their population, often due to disparities in access to resources (e.g., low-income or minoritized racial/ethnic groups). Others noted fit problems due to the clinical population, such as not working as well with for youth in residential care or with severe mental health problems. | OF, SF; P, A | One of the difficulties with ACRA … is that there is a big component of getting kids involved in more pro-social activities. And there’s a lot of barriers to that culturally and otherwise and financially for folks…. so there aren’t a lot of resources for things for young people to do, especially in the after school hours, or the money to do a whole lot of extra things outside in the community for some of the families that we serve. (SF, P) |
|  | Positive Client Perceptions of A-CRA (F) | Families and youth often found the A-CRA model easy to understand. Clients seemed engaged in the treatment and enjoyed the procedures, specifically ones that allowed them to understand their behaviors and how to change them. | OF, SF; P, A | They loved [A-CRA]. They loved it because it was fun to them. They loved it because they learned so much from it and it put them kind of like in control of a lot of decisions for themselves. ... They were like. “Okay, I can make this decision. I can do this and I can do that.” (SF, P) |
|  | Low Client Engagement in Treatment (B) | Some youth and their families were not interested in participating in youth substance use treatment in general, making it hard to implement any model. | OF, SF; P | Some courts order treatments for youth, and these youth are resistant to work with… A-CRA needs more involvement from the clients and that makes it difficult to engage this kind of client in A-CRA…and some clients are just not interested at all, or they are against it. Some don't care. They don't want to stop doing drugs. So, it's tougher to make it work. (OF, P) |
|  | COVID Impact on A-CRA Telehealth Delivery (F) | Providing A-CRA via telehealth in response to the COVID-19 pandemic allowed for more opportunities to engage clients and maintain continuity in their care. | SF; P, A | What's been beneficial is being able to get the parents’ involvement, because sessions are being conducted [virtually] in the home. You don't have to come in ... there has been at least an increase in parent participation. (SF, P) |
|  | Technological barriers to telehealth access (B) | Client technological problems, including connection and equipment issues, made it difficult for some clients to access telehealth care during the pandemic. | SF; P, A | And [COVID-19] was an issue with the youth because some of them did not have the technology needed, especially those from indigent circumstances. And so that was a challenge at the very beginning [of the pandemic]. (SF, P) |
| Inner Context  Inner Context, continued  Inner Context, continued | Organization Leadership Support (F/B) | Champions in organizations, such as directors and administrators, helped provide resources for A-CRA implementation, expressed support for it as a beneficial model, and encouraged providers to finish their certification. In contrast, some organizations’ leadership did not support the model, and thus, did not support their staff in implementing. | OF, SF; P, A | [The team] had a good impact on the program or implementation, such as my supervisor at the time provided resources and support when needed. She was all about A-CRA and making sure that the program was successful and she was really invested in it. (SF, P)  Some challenges with the organizations would be their upper level management, not wanting to put the employees into having that time and effort to actually go through the certification process, because they weren't necessarily making money off those pieces. (SF, A) |
|  | Supervisor Support (F) | The availability of and support from A-CRA supervisors through coaching helped encourage completion of certification. | OF, SF; P, A | Just having onsite training and having our supervisors who are here, who are very knowledgeable of the [A-CRA] program, having hands on guidance is definitely helpful. If there's anything we're not sure of … there's always somebody that you can go to. (SF, P) |
|  | Clinician Support (F/B)  (*continues*)  (*continued from previous page*) | Clinicians who bought into the model’s structure, recognized the benefits of the curriculum, and felt supported in their efforts continued through their certification process and used A-CRA with clients. But some clinical staff expressed resistance to implementing the model and did not feel that the costs of the training, certification, and supervision, outweighed the benefits of A-CRA. | OF, SF; P, A | I think a lot of the components of A-CRA like the structure, the ability to understand the rationale, all these components of it make it easy to implement, and then it makes it easy to understand why we're doing it. And I think that creates a lot of buy-in for clinicians and for clients a lot of the times (SF, P)  There was some pressure, even from some of our staff to discontinue the official supervision and training component [for A-CRA], because it was too much. (SF, P) |
|  | Organization Turnover (B) | Turnover of clinical staff and leadership was a major barrier that led to loss of support and prolonged implementation activities (e.g., certification). | OF, SF; P, A | Staffing was limited, we had a bunch of turnover. And throughout [our state], there's a staffing shortage, so as far as clinicians go. That's nationwide. But it's hard to bring on clinicians and train somebody up and then have them leave. And then having put all that time into them, it's hard to justify doing it again. (SF, P) |
|  | Organization Policies Impacting A-CRA (F/B) | Organization policies made it easier or more difficult to implement A-CRA depending on the specifics of the policy. Facilitator policies included promoting the use of EBPs, providing resources useful for A-CRA delivery such as client incentives, and flexibility in provider’ schedules. Barrier policies were typically strict scheduling policies or direct time requirements, such that A-CRA-related paperwork and supervision were difficult to fit in the workday. | OF, SF; P | Our clinicians who were providing ACRA were given a lot more flexibility in their ability to schedule to try to meet the needs of those particular clients, so that was definitely an area that helped…[They] were able to adjust a little bit more freely. Keep clients longer, make more efforts to reach out to families…(SF, P)  I think one of the policies that interferes is there is a very strictly enforced direct time requirement for clinicians. They work eight hours a day, six of the eight hours they're required to be in direct client care. So, with ACRA there's time that people have to get stuff together to deliver it. (SF, P) |
|  | Strategic Planning (F) | Treatment organizations described engaging in strategic planning to promote adoption and use of A-CRA, with an emphasis on fully successful implementation and preparing for sustainment after the SAMHSA funding ended. | OF; P | Part of planning was also to work with state legislature, [Department of Children and Families], and [the] Court Services Division to let them know about the model we're doing and seek longer-term state-based funding for these services. We have also done some work with State Department of Social Service… to advocate for this model as being paid for in full. (OF, P) |
|  | COVID Impact on Turnover (B) | The COVID-19 pandemic exacerbated existing staff shortages within organizations. High turnover rates left organizations without certified providers who could deliver A-CRA. | SF; P, A | When I began, I had three other clinicians that all basically left that year between July and August…so I think all of those clinicians at that time were all trained in [A-CRA]. They had a lot more in depth knowledge. And I think with the pandemic and us trying to do that in telehealth and then with that turnover, I think that’s was what really provided the biggest barrier was with staff turnover. (SF, P) |
| Outer Context  Outer Context, continued  Outer Context, continued | State Leadership Support (F/B)  (*continues*)  (*continued from previous page*) | Having state agency staff dedicated to working with treatment organizations on A-CRA helped facilitate project administration, encouraged buy-in at the organizational level, and helped move providers through the certification process. Whereas participants in other states noted their state agency did not encourage use of A-CRA and the model was not marketed well in those states. | SF; P, A | And we kept in really good contact with the people from the state department. … I remember the guy who was from the state department that I talked to about the [A-CRA] program and stuff, we talked to each other at least, I would say every other week, at a minimum. So from the state department all the way down, I think everyone involved in the program was pretty supportive of it. (SF, P)  There were no real champions [for A-CRA within our state agency]… If there was enough mutual support, then I think that we can overcome limitations of people’s motivation to [get certified]. (SF, A) |
|  | State Policies Impacting A-CRA (F/B) | State policies supported or impeded A-CRA implementation depending on the specifics of the policy. Facilitator policies included requiring the use of EBPs or harm reduction models, or offering additional funding for the use of EBPs such as A-CRA. Barrier policies were typically strict billing requirements that did not allow funding to cover time and resources needed to deliver A-CRA  outside of client time, like training, supervision, and paperwork | SF; P, A | One of the reasons why the [state-focused] grant is being utilized so much is there's a big push in our state for making sure we use evidence-based approaches in our clinical work. And so knowing that ACRA is evidence-based, it is kind of one that we're really enjoying using because of that and it kind of checks those boxes. (SF, P)  As we started looking at sustainability, we could see that A-CRA could not be self-sustaining… [To] sustain a counselor position under third party billing, they need a sizeable caseload… We weren't getting those numbers under the A-CRA program… Also, with A-CRA we were more limited with what we could bill for. (OF, P) |
|  | Community Beliefs about Substance Use (B) | Substance use issues were seen as a less serious problem in some communities – including among caregivers, partner organizations, and providers – decreasing youth referrals and engagement. Some participants attributed attitudes to increased legalization of cannabis. | SF; P, A | There's been tremendous reduction in referrals to substance use treatment. And most of those, that reduction, is really due to the decline of children and teenagers, requesting families requesting treatment for marijuana [use]. I mean, it's really significant. It's a dramatic drop (SF, A). |
|  | Adolescents not a Priority Group (B)  (*continues*)  (*continued from previous page*) | Within some communities, adolescents were not viewed as a priority treatment group. Thus, state and federal funding was focused on adult populations instead of youth, limiting the resources available for youth treatments like A-CRA. | SF; P, A | There’s also a lot more adult resources for substance use treatment… There’s a lot more resources available to adults than there are for youth…You kind of have to make do with what you have until you get to 18. (SF, P) |
| Bridging Factors  Bridging Factors, continued  Bridging Factors, continued | State Funding (F) | The money treatment organizations received from the state for state-focused grants was integral to funding training, certification, supervision, and the resources needed to fully understand and deliver A-CRA with clients. | SF; P, A | One of the most important things that [state agency] can provide is accessibility to training and evidence-based models of treatment. My [organization] is a nonprofit. We serve an underserved community. When SAMHSA, through the states, is able to offer us access to training, consultation, and support, that’s an important resource. (SF, P) |
|  | State-led Training Activities (F) | The partnership between states and Chestnut Health Systems to provide initial A-CRA training was viewed as informative to providers and state administrators, and helped them implement the model. | SF; P, A | Talking about the state, they were able to provide the [A-CRA] training initially, which is how we were able to get it started, so that helped immensely. (SF, P) |
|  | Other State Activities (F) | Some states created advisory councils and learning collaboratives so providers using A-CRA could interact with each other, brainstorm solutions to problems, and share best practices for implementation. | SF; P, A | …Our quarterly learning collaborative…We convene all of the ACRA [provider organizations] across the state and it’s a feedback loop on all things related to infrastructure sustainability trends on ACRA so that we can always be advancing it (SF, A) |
|  | Follow Up from State and Chestnut Health Systems (F/B)  (*continues*)  (*continued from previous page*) | Follow-up from state agencies and Chestnut Health Systems regarding the status of their training and certification process was beneficial to providers since it served as helpful reminders to complete A-CRA certification. However, some providers felt that there was a lack of follow-up which made them less likely to follow through with certification | OF, SF; P, A | Chestnut was amazing. They were amazing. I mean, they sent monthly reports. They follow up and offered all kinds of support. They worked with me on a regular basis. (SF, A)  Everybody was close [to A-CRA certification], and we had a couple of people who made it all the way through, and we were doing those monthly supervision things, and then those just kind of stopped happening…. We really weren’t very incentivized to finish. (SF, P) |
|  | Intensive Support for Organization-Focused Grants (F) | Many participants found the support provided by SAMHSA organization-focused grants to be of good quality. | OF; P | I think SAMHSA provided a lot of good training with the [A-CRA provider organizations] involved… I felt like there was a lot of support. It helped us get through what we needed to do. (OF, P) |
|  | Assessment Tool was Cumbersome (B) | The GAIN (Global Assessment of Individual Needs), an assessment SAMHSA required organization-focused grantees to collect and report, was frequently seen as overly complex and burdensome. | OF; P | [GAIN] is time consuming, and [we] would prefer to have other staff besides clinicians to collect data so [they] could focus on seeing clients… Sometimes, we would switch because the client would not want to see the clinician again when they needed to collect data for the GAIN. (OF, P) |
|  | Too Much Reporting/ Paperwork (B) | Reporting and paperwork related to administration of federal grants were seen as a barrier to using organization-focused grants | OF; P | It was a very intensive-labor process, and it was monitored closely by SAMHSA … you would not be able to sustain that level of involvement per client. You would have to cut down on all the reporting. (OF, P) |
|  | Provider Organization Partnerships and Referral Sources (F/B)  (*continues*)  (*continued from previous page*) | Having connections with state mental health departments, child and family services, and court systems increased referrals of A-CRA clients to treatment organizations. Relationships with community and local organizations like schools were also important for marketing A-CRA and receiving referrals and resources. However, some participants noted a lack of commitment from their state and local partners which limited referrals for A-CRA. | OF, SF; P | Community partners, whether it be the school court services or [Department of Family Services], them being knowledgeable about just the services that we offer and being able to refer individuals to us has been very important. (SF, P)  I think [A-CRA] could help all the way around, but we just didn’t have the numbers. ... I really thought we’d have the referral, especially with juvenile court knew we were doing this and I figured that once they found out we were doing this that they would send clients our way. We got zero referrals. (SF, P) |
|  | State Partnerships with Other Agencies (F) | Working with insurance companies regarding billing and reimbursement rates was helpful in covering extra costs associated with A-CRA. Working with other agencies in the state was also beneficial for increasing referrals. | SF; P, A | We certainly look to facilitate opportunities to collaborate. We look to facilitate opportunities [to] connect with our potential referral sources, whether it be my local schools, local youth serving hospitals and medical centers, as well as other agencies that could serve as referral sources for the providers. (SF, A) |
|  | Impact of COVID on Referrals (B) | Due to changes in interactions with referrals services (e.g., school, court system) during pandemic disruptions, less youth were being identified with substance use issues and thus referred for services. | SF; P, A | The majority of services were initially transferred to virtual because our school districts were primarily virtual all during the last school year. That impacted our ability to get referrals, so adolescent services dropped significantly. (SF, A) |

*Note*. A-CRA = Adolescent Community Reinforcement Approach. EPIS = the Exploration, Preparation, Implementation, and Sustainment framework. GAIN = Global Assessment of Individual Needs. SAMHSA = U.S. Substance Abuse and Mental Health Services Administration.

Key for determinant types: B = barrier, F = facilitator, F/B = facilitator or barrier (depending on the circumstances).

Key for interview types with code, meaning that the code was present in interviews with each type of participant listed (the same abbreviations are also used for Exemplar Quote attributions): OF = Organization-focused grants, SF = State-focused grants, P = Providers (Clinicians and/or Supervisors), A = State Administrators. P interviews were completed for OF and SF grants, whereas A interviews were only completed for SF grants.
